# The Burden of Cardiovascular Diseases Among US States, 1990-2016

**DOI:** 10.1001/jamacardio.2018.0385

**Published:** 2018-04-11

**Authors:** Global Burden of Cardiovascular Diseases Collaboration, Gregory A. Roth, Catherine O. Johnson, Kalkidan Hassen Abate, Foad Abd-Allah, Muktar Ahmed, Khurshid Alam, Tahiya Alam, Nelson Alvis-Guzman, Hossein Ansari, Johan Ärnlöv, Tesfay Mehari Atey, Ashish Awasthi, Tadesse Awoke, Aleksandra Barac, Till Bärnighausen, Neeraj Bedi, Derrick Bennett, Isabela Bensenor, Sibhatu Biadgilign, Carlos Castañeda-Orjuela, Ferrán Catalá-López, Kairat Davletov, Samath Dharmaratne, Eric L. Ding, Manisha Dubey, Emerito Jose Aquino Faraon, Talha Farid, Maryam S. Farvid, Valery Feigin, João Fernandes, Joseph Frostad, Alemseged Gebru, Johanna M. Geleijnse, Philimon Nyakauru Gona, Max Griswold, Gessessew Bugssa Hailu, Graeme J. Hankey, Hamid Yimam Hassen, Rasmus Havmoeller, Simon Hay, Susan R. Heckbert, Caleb Mackay Salpeter Irvine, Spencer Lewis James, Dube Jara, Amir Kasaeian, Abdur Rahman Khan, Sahil Khera, Abdullah T. Khoja, Jagdish Khubchandani, Daniel Kim, Dhaval Kolte, Dharmesh Lal, Anders Larsson, Shai Linn, Paulo A. Lotufo, Hassan Magdy Abd El Razek, Mohsen Mazidi, Toni Meier, Walter Mendoza, George A. Mensah, Atte Meretoja, Haftay Berhane Mezgebe, Erkin Mirrakhimov, Shafiu Mohammed, Andrew Edward Moran, Grant Nguyen, Minh Nguyen, Kanyin Liane Ong, Mayowa Owolabi, Martin Pletcher, Farshad Pourmalek, Caroline A. Purcell, Mostafa Qorbani, Mahfuzar Rahman, Rajesh Kumar Rai, Usha Ram, Marissa Bettay Reitsma, Andre M. N. Renzaho, Maria Jesus Rios-Blancas, Saeid Safiri, Joshua A. Salomon, Benn Sartorius, Sadaf Ghajarieh Sepanlou, Masood Ali Shaikh, Diego Silva, Saverio Stranges, Rafael Tabarés-Seisdedos, Niguse Tadele Atnafu, J. S. Thakur, Roman Topor-Madry, Thomas Truelsen, E. Murat Tuzcu, Stefanos Tyrovolas, Kingsley Nnanna Ukwaja, Tommi Vasankari, Vasiliy Vlassov, Stein Emil Vollset, Tolassa Wakayo, Robert Weintraub, Charles Wolfe, Abdulhalik Workicho, Gelin Xu, Simon Yadgir, Yuichiro Yano, Paul Yip, Naohiro Yonemoto, Mustafa Younis, Chuanhua Yu, Zoubida Zaidi, Maysaa El Sayed Zaki, Ben Zipkin, Ashkan Afshin, Emmanuela Gakidou, Stephen S. Lim, Ali H. Mokdad, Mohsen Naghavi, Theo Vos, Christopher J. L. Murray

**Affiliations:** 1 Institute for Health Metrics and Evaluation, University of Washington, Seattle; 2 Division of Cardiology, Department of Medicine, University of Washington, Seattle; 3 Jimma University, Jimma, Ethiopia; 4 Cairo University, Giza, Egypt; 5 The University of Western Australia, Perth, Western Australia, Australia; 6 Universidad de Cartagena, Cartagena, Bolívar, Columbia; 7 Zahedan University of Medical Sciences, Zahedan, Iran; 8 Karolinska Institutet, Stockholm, Sweden; 9 Mekelle University, Mekelle, Ethiopia; 10 Indian Institute of Public Health Gandhinagar, Public Health Foundation of India, Gandhinagar, Gujarat, India; 11 University of Gondar, Gondar, Ethiopia; 12 University of Belgrade, Belgrade, Serbia; 13 Harvard University, Cambridge, Massachusetts; 14 Jazan University, Jizan, Saudi Arabia; 15 University of Oxford, Oxford, England; 16 University of São Paulo, São Paulo, São Paulo, Brazil; 17 Independent Public Health Consultant, Addis Abbaba, Ethiopia; 18 Observatorio Nacional de Salud, Instituto Nacional de Salud, Bogotá, DC, Colombia; 19 INCLIVA Health Research Institute, Centro de Investigación Biomédica en Red Salud Mental, University of Valencia, Valencia, Spain; 20 Asfendiyarov Kazakh National Medical University, Almaty, Kazakhstan; 21 University of Peradeniya, Peradeniya, Sri Lanka; 22 Harvard T. H. Chan School of Public Health, Boston, Massachusetts; 23 International Institute for Population Sciences, Mumbai, India; 24 University of the Philippines Manila, Manila, Philippines; 25 University of Louisville, Louisville, Kentucky; 26 Auckland University of Technology, Auckland, New Zealand; 27 Catholic University of Portugal, Lisbon, Portugal; 28 Wageningen University, Wageningen, the Netherlands; 29 University of Massachusetts Boston, Boston; 30 School of Medicine and Pharmacology, University of Western Australia, Perth, Western Australia, Australia; 31 Mizan Tepi University, Tepi, Ethiopia; 32 Debre Markos University, Debre Markos, Ethiopia; 33 Hematology, Oncology, and Stem Cell Transplantation Research Center, Tehran University of Medical Sciences, Tehran, Iran; 34 New York Medical College, Valhalla; 35 Al Imam Mohammad Ibn Saud Islamic University, Riyadh, Saudi Arabia; 36 Ball State University, Muncie, Indiana; 37 Northeastern University, Boston, Massachusetts; 38 Brown University, Providence, Rhode Island; 39 Public Health Foundation of India, New Delhi, India; 40 Department of Medical Sciences, Uppsala University, Uppsala, Sweden; 41 University of Haifa, Haifa, Israel; 42 Clinical Research Center, University Hospital, University of São Paulo, São Paulo, São Paulo, Brazil; 43 Faculty of Medicine, Mansoura University, Mansoura, Egypt; 44 State Key Laboratory of Molecular Developmental Biology, Institute of Genetics and Developmental Biology, Chinese Academy of Sciences, Chaoyang, Beijing; 45 Martin Luther University of Halle-Wittenberg, Halle, Germany; 46 United Nations Population Fund, New York, New York; 47 National Institutes of Health, Bethesda, Maryland; 48 University of Melbourne, Melbourne, Victoria, Australia; 49 Kyrgyz State Medical Academy, Bishkek, Kyrgyzstan; 50 Ahmadu Bello University, Zaria, Kaduna State, Nigeria; 51 Columbia University, New York, New York; 52 Department of Medicine, University of Ibadan, Ibadan, Oyo State, Nigeria; 53 University of British Columbia, Vancouver, British Columbia, Canada; 54 Noncommunicable Diseases Research Center, Alborz University of Medical Sciences, Hassan Abad, Karaj, Iran; 55 BRAC, Dhaka, Bangladesh; 56 Society for Health and Demographic Surveillance, West Bengal, India; 57 Western Sydney University, Penrith, New South Wales, Australia; 58 National Institute of Public Health, Wako, Japan; 59 Maragheh University of Medical Sciences, East Azerbaijan Province, Iran; 60 University of KwaZulu-Natal, Durban, South Africa; 61 Tehran University of Medical Sciences, Tehran, Iran; 62 Independent Consultant, Karachi, Pakistan; 63 Federal University of Santa Catarina, Florianópolis, Santa Catarina, Brazil; 64 Department of Epidemiology and Biostatistics, Schulich School of Medicine & Dentistry, Western University, London, Ontario, Canada; 65 Addis Ababa University, Addis Ababa, Ethiopia; 66 Postgraduate Institute of Medical Education and Research, Chandigarh, India; 67 Jagiellonian University Medical College, Krakow, Poland; 68 University of Copenhagen, Copenhagen, Denmark; 69 Cleveland Clinic, Cleveland, Ohio; 70 Hospital Sant Joan de Déu Barcelona, Sant Joan de Déu Research Foundation, Centro de Investigación Biomédica en Red Salud Mental, Universitat de Barcelona, Barcelona, Spain; 71 Department of Internal Medicine, Federal Teaching Hospital, Abakaliki, Ebonyi State, Nigeria; 72 UKK Institute for Health Promotion Research, Tampere, Finland; 73 National Research University Higher School of Economics, Moscow, Russia; 74 Norwegian Institute of Public Health, Oslo, Norway; 75 Royal Children’s Hospital, Melbourne, Victoria, Australia; 76 King’s College London, London, England; 77 Nanjing University School of Medicine, Nanjing, China; 78 The University of Mississippi Medical Center, Jackson; 79 University of Hong Kong, Pokfulam, Hong Kong; 80 Kyoto University, Kyoto, Japan; 81 Jackson State University, Jackson, Mississippi; 82 Wuhan University, Wuhan, China; 83 University Hospital of Setif, Setif, Algeria

## Abstract

**Importance:**

Cardiovascular disease (CVD) is the leading cause of death in the United States, but regional variation within the United States is large. Comparable and consistent state-level measures of total CVD burden and risk factors have not been produced previously.

**Objective:**

To quantify and describe levels and trends of lost health due to CVD within the United States from 1990 to 2016 as well as risk factors driving these changes.

**Design, Setting, and Participants:**

Using the Global Burden of Disease methodology, cardiovascular disease mortality, nonfatal health outcomes, and associated risk factors were analyzed by age group, sex, and year from 1990 to 2016 for all residents in the United States using standardized approaches for data processing and statistical modeling. Burden of disease was estimated for 10 groupings of CVD, and comparative risk analysis was performed. Data were analyzed from August 2016 to July 2017.

**Exposures:**

Residing in the United States.

**Main Outcomes and Measures:**

Cardiovascular disease disability-adjusted life-years (DALYs).

**Results:**

Between 1990 and 2016, age-standardized CVD DALYs for all states decreased. Several states had large rises in their relative rank ordering for total CVD DALYs among states, including Arkansas, Oklahoma, Alabama, Kentucky, Missouri, Indiana, Kansas, Alaska, and Iowa. The rate of decline varied widely across states, and CVD burden increased for a small number of states in the most recent years. Cardiovascular disease DALYs remained twice as large among men compared with women. Ischemic heart disease was the leading cause of CVD DALYs in all states, but the second most common varied by state. Trends were driven by 12 groups of risk factors, with the largest attributable CVD burden due to dietary risk exposures followed by high systolic blood pressure, high body mass index, high total cholesterol level, high fasting plasma glucose level, tobacco smoking, and low levels of physical activity. Increases in risk-deleted CVD DALY rates between 2006 and 2016 in 16 states suggest additional unmeasured risks beyond these traditional factors.

**Conclusions and Relevance:**

Large disparities in total burden of CVD persist between US states despite marked improvements in CVD burden. Differences in CVD burden are largely attributable to modifiable risk exposures.

## Key Points

**Question:**

How does the total burden of cardiovascular diseases vary across US states?

**Findings:**

In this study using the Global Burden of Disease methodology, large disparities in total burden of CVD were found between US states despite marked improvements in CVD burden.

**Meaning:**

These estimates can provide a benchmark for states working to focus on key risk factors, improve health care quality, and lower health care costs.

## Teaser

This study using the Global Burden of Disease methodology quantifies and describes levels and trends of lost health due to cardiovascular disease within the United States from 1990 to 2016 as well as risk factors driving these changes.

## Introduction

Cardiovascular disease (CVD) was the leading cause of death in the United States in 2016, accounting for more than 900 000 deaths.^1^ Despite large declines in CVD mortality in the late 20th century attributed to advances in public health and health care, improvements in US life expectancy have slowed for some groups, and CVD mortality is no longer improving.^2,3,4,5^ The strongest signal for this alarming trend in US health is identified subnationally at the state and county level, where levels of risk exposure and health vary widely.^6,7,8^

Geographic variation in CVD has many determinants, but these are not usually evaluated in a consistent and comparable manner across all states. Rapid changes in average risk at the national level, such as large declines in plasma cholesterol levels over a relatively short period due to increased use of 3-hydroxy-3-methylglutaryl coenzyme A reductase inhibitors, suggest that subnational evaluation of cardiovascular risk is needed to understand persistent health disparities.^9,10^ Geographic variation in the quality of primary, prehospital, acute, and long-term cardiovascular care also requires a comprehensive, subnational assessment.^11,12,13^

The Global Burden of Disease (GBD) Study 2016^1^ was a study of global health across 332 causes of disease and injury and 84 risk factors in 195 countries and territories. In this article, we report the study’s US state-level results for CVD and its modifiable risk factors.

## Methods

### Overview

The methods of the GBD Study 2016 have been reported in detail previously.^1,14,15,16^ The study used data on incidence, prevalence, mortality, and risk exposure to produce comparable estimates of disease burden. All analyses were done separately by sex and aggregated by 5-year age categories. A detailed discussion of data sources and methods are provided in eMethods 1 through 5 in the Supplement, with a brief overview below. This study was reviewed and approved by the University of Washington institutional review board, and informed consent was waived because deidentified data were used.

### Causes of CVD

Cardiovascular disease was estimated for the 10 most common global causes of CVD-related death and an additional category that combined all other CVD and circulatory conditions. These causes were ischemic heart disease (IHD), ischemic stroke, hemorrhagic and other stroke, atrial fibrillation, peripheral artery disease, aortic aneurysm, cardiomyopathy and myocarditis, hypertensive heart disease, endocarditis, and rheumatic heart disease. Death due to each underlying CVD cause was defined by categorization of *International Classification of Diseases* (*ICD*) codes.^1^ Disease incidence and prevalence were defined according to a set of standard case definitions mapped to these codes based on expert guidance.^14^ These included the third universal definition of myocardial infarction, the World Health Organization definition for stroke, electrocardiographic identification of atrial fibrillation, diagnosis of peripheral arterial disease by ankle-brachial index, the World Heart Federation criteria for definite rheumatic heart disease, and the Framingham Heart Study definition of congestive heart failure. Stroke deaths assigned to a non–subtype-specific code (*ICD *code I64) were reassigned to subtypes using the proportion of ischemic to hemorrhagic strokes.

### Data

Data sources and methods for estimation of CVD have been previously described.^17^ In brief, population counts were obtained from the US Census Bureau for each state.^18^ Death certificate data were obtained from the National Center for Health Statistics for each state. *ICD*-*9* and *ICD*-*10* codes were aggregated for each cause of CVD. Structured reviews of published literature were performed to identify published and unpublished data on incidence, prevalence, case fatality, and mortality related to CVD causes. State-level inpatient and outpatient claims data were obtained from a database of private and public insurance schemes for 2000, 2010, and 2012.^19^ *ICD*-*9* codes were aggregated for each CVD case definition and used to calculate the annual incidence (using inpatient data) or prevalence rate (using inpatient and outpatient data combined) for selected health conditions, stratified by age, sex, year, and state. A correction factor was applied to account for changes in coding of administrative claims data over time. Data on risk factor exposure were obtained from multiple sources, including the National Health and Nutrition Examination surveys, the Behavioral Risk Factor Surveillance surveys, satellite data and air sampling data for estimation of particulate matter less than 2.5 µm in diameter, and a systematic review of published scientific literature. Surveys with complex sampling design, including National Health and Nutrition Examination surveys and Behavioral Risk Factor Surveillance surveys, were analyzed using appropriate sample weights to accurately estimate variance. Risk factor definitions and attribution methods have been previously reported.^15^ Definitions of metabolic exposures included fasting plasma glucose level measured in millimoles per liter, total cholesterol level measured in millimoles per liter, systolic blood pressure measured in millimeters of mercury, and body mass index (calculated as weight in kilograms divided by height in meters squared).^15^

### Estimation of CVD Burden

All-cause, all-cardiovascular, and cause-specific mortality were estimated using the Cause of Death Ensemble Model, which produces cause-specific smoothed trends over time by age, sex, and state. Atrial fibrillation mortality was estimated with a separate natural history model described below. DisMod-MR, a Bayesian meta-regression tool developed for the GBD Study,^1^ was used to estimate prevalence and incidence for each cause. This software produced estimates for 6 estimation years (1990, 1995, 2000, 2005, 2010, and 2016), including data from a selected number of years before and after each estimation year when estimating for these time points. Interpolation was performed to produce a continuous series of annual results. Analysis was performed at the level of specific disease sequelae (for example, IHD due to acute coronary syndrome, chronic stable angina, chronic ischemic heart disease, and ischemic cardiomyopathy) by age, sex, year, and state. Adjustments were made to data that did not follow the selected case definition (eg, electronic claims to clinical diagnosis) by a regression model that crosswalked values in the direction of case definition–based data.^17^ Heart failure prevalence was estimated and then attributed proportionally to its underlying causes, including IHD, nonischemic cardiomyopathy, and myocarditis. We include a separate analysis of total heart failure prevalence, given its importance to clinical care and public health. For atrial fibrillation, both prevalence and cause-specific mortality were estimated using DisMod-MR because mortality based on vital registration data alone provides an implausibly steep increase over time believed to represent changes in ascertainment rather than the disease’s epidemiology. Prevalence was estimated across a range of severities for each condition as well as an asymptomatic state. Severity levels for each disease were estimated using data from the Medical Expenditure Panel Survey except for stroke, which was estimated from a model of Rankin scores collected within stroke registries, as described previously.^17^ Disability weights were developed to represent functional capacity for each severity level and multiplied by prevalence to calculate years lived with disability (YLDs), a summary measure of health among those living with a condition. Years lived with disability for sequelae are summed for their parent cause. Disability weights for the GBD Study 2016,^1^ including data collection and methods, have been previously described.^14,20,21^ Adjustments were made for comorbidity using a microsimulation process in which persons had an independent probability of having each sequela, and the probability was derived from the prevalence estimates. Years of life lost (YLLs) prematurely due to a cause was calculated by multiplying observed deaths for a specific age in the year of interest by a global age-specific reference life expectancy estimated using life table methods.

### Disability-Adjusted Life-Years, Attributable Risks, and Sociodemographic Index

The disability-adjusted life-year (DALY) is a summary measure of health that was calculated for each age-sex-year-state-cause strata by summing the fatal (YLL) and nonfatal (YLD) components.^16^ For example, age-sex-state-year–specific numbers of YLLs due to IHD were added to the matching YLDs due to IHD to produce DALYs due to IHD. By dividing by population for that same strata, a DALY rate per 100 000 individuals was calculated. In the absence of health examination data from states, we predicted mean systolic blood pressure and total cholesterol levels for each state with a regression model combining covariates from the Behavioral Risk Factor Surveillance surveys and National Health and Nutrition Examination surveys. For risk factors estimated as continuous variables, we developed an ensemble distribution for each risk modeled using a family of probability density functions, a fitting method, a model selection criteria, and the method of moments.^15^ Population-attributable fractions of disease by cause were modeled based on estimates of exposure level, relative risk, and theoretical (eg, counterfactual) minimum risk levels using methods previously described.^15^ We accounted for joint effects of combinations of risk factors when sufficient evidence existed for a causal relationship. We modeled mediation pathways using individual-level data from prospective cohort studies and estimated the proportion of cardiometabolic effect from each metabolic and behavioral risk factor.

We performed a decomposition analysis of the change in DALYs from 2006 to 2016, estimating the change in CVD DALYs that would be observed after removal of the effects of population aging, population growth, and GBD Study 2016 CVD–associated risks.^1^ The decomposition analysis was undertaken at the all-risk level, taking into account risk mediation at the most detailed cause level. This was repeated at the most detailed risk-outcome level. The contribution of risk exposures over longer periods, eg, 2006 to 2016, or at higher cause aggregates, eg, all-CVD mortality, were calculated as the linear aggregate of the effect of individual risks at the most detailed cause level and period.

To provide a consistent comparison by socioeconomic status, a sociodemographic index (SDI) was estimated by state using equally weighted age-sex-state-year–specific geometric means of income per capita, educational attainment, and total fertility rate. The metric of SDI was used for consistency across all global locations included in the GBD 2016 Study.

The 95% uncertainty intervals (UIs) reported for each estimate used 1000 samples from the posterior distribution from the respective step in the modeling process, reported as the 2.5th and 97.5th values of the distribution. Age standardization was calculated via the direct method, applying a global age structure. Differences in estimates were considered significant if 95% UIs did not overlap.

## Results

### SDI and Change in Total CVD Burden

**Figure 1.**
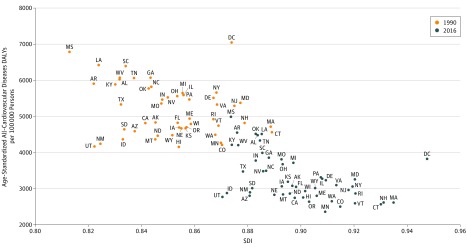
Scatterplot of Age-Standardized Cardiovascular Disease Disability-Adjusted Life-Years (DALYs) per 100 000 Persons and Sociodemographic Index (SDI) in 1990 and 2016 To provide a consistent comparison by socioeconomic status, an SDI was estimated by state using equally weighted age-sex-state-year–specific geometric means of income per capita, educational attainment, and total fertility rate.

Several states had large rises in their relative rank ordering for total CVD DALYs among states, including Arkansas, Oklahoma, Alabama, Kentucky, Missouri, Indiana, Kansas, Alaska, and Iowa (Figure 1). A notable outlier was the (nonstate) District of Columbia, which achieved the highest SDI in the United States from 1990 to 2016 while decreasing its age-standardized CVD DALY rate from the highest in the United States in 1990 (7044 DALYs per 100 000; 95% UI, 6194-7482) to the 11th highest in 2016 (3821 DALYs per 100 000 persons; 95% UI, 3424-4209).

### Change in Total CVD Burden, 1990-2016

**Figure 2.**
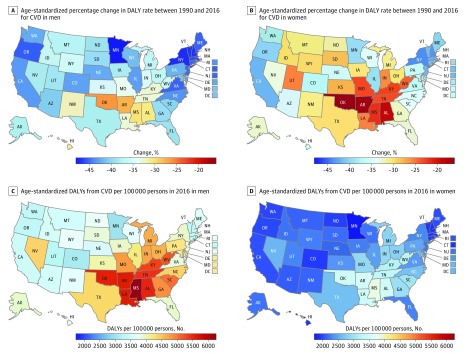
Maps of Age-Standardized Disability-Adjusted Life-Year (DALY) Rate and Percentage Change in DALY Rate for All Cardiovascular Diseases (CVDs) by Sex A, Percentage change in age-standardized DALY rate of CVD for men between 1990 and 2016. B, Percentage change in age-standardized DALY rate of CVD for women between 1990 and 2016. C, Age-standardized DALYs from CVD per 100 000 persons in 2016 for men. D, Age-standardized DALYs from CVD per 100 000 persons in 2016 for women.

The age-standardized rate of CVD DALYs decreased significantly in all states between 1990 and 2016, but there was wide regional variation in the amount of this decline (Table; eTable 1 in the Supplement). The largest percentage change occurred in the District of Columbia, New Hampshire, and New York. The rate of decline varied by sex, with a slower decline for women than men in all states (Figure 2A and B). The slowest decline was observed for women in Oklahoma, Arkansas, and Alabama. Total CVD burden increased for both men and women from 2010 to 2016 in Indiana, Kentucky, Michigan, Mississippi, Missouri, New Mexico, and South Dakota.

**Table.** Total and Age-Standardized Rate of All-Age, All–Cardiovascular Disease Disability-Adjusted Life-Years (DALYs) and Percentage Change of DALYs by US State in 1990, 2006, and 2016

State	No. of DALYs (95% UI)			Change in DALYs, % (95% UI)		Age-Standardized DALY Rates per 100 000 Persons (95% UI)			Change in DALY Rates, % (95% UI)	
	1990	2006	2016	1990-2016	2006-2016	1990	2006	2016	1990-2016	2006-2016
United States	14 942 504 (14 528 977 to 15 366 692)	13 795 265 (13 256 320 to 14 337 878)	14 824 304 (14 204 082 to 15 498 029)	−0.01 (−0.03 to 0.02)	0.07 (0.05 to 0.09)	5282 (5138 to 5430)	3687 (3543 to 3831)	3269 (3134 to 3418)	−0.38 (−0.40 to −0.37)	−0.11 (−0.13 to −0.10)
Alabama	283 159 (270 172 to 296 435)	285 841 (272 385 to 300 018)	307 235 (275 629 to 338 582)	0.09 (−0.03 to 0.20)	0.08 (−0.03 to 0.19)	6029 (5751 to 6321)	4842 (4609 to 5084)	4482 (4008 to 4944)	−0.26 (−0.33 to −0.18)	−0.07 (−0.17 to 0.02)
Alaska	14 428 (13 585 to 15 329)	19 462 (18 242 to 20 682)	24 105 (21 474 to 26 865)	0.67 (0.48 to 0.88)	0.24 (0.10 to 0.40)	4833 (4576 to 5117)	3279 (3080 to 3476)	3074 (2751 to 3415)	−0.36 (−0.43 to −0.29)	−0.06 (−0.16 to 0.05)
Arizona	190 297 (181 234 to 199 224)	243 045 (231 017 to 255 859)	280 232 (256 911 to 304 431)	0.47 (0.36 to 0.60)	0.15 (0.06 to 0.24)	4588 (4362 to 4808)	3262 (3099 to 3432)	2803 (2568 to 3056)	−0.39 (−0.44 to −0.34)	−0.14 (−0.21 to −0.08)
Arkansas	177 086 (168 998 to 184 754)	176 402 (168 535 to 184 723)	190 918 (176 514 to 205 755)	0.08 (−0.01 to 0.17)	0.08 (0 to 0.17)	5905 (5639 to 6169)	4791 (4575 to 5020)	4549 (4190 to 4911)	−0.23 (−0.29 to −0.16)	−0.05 (−0.13 to 0.03)
California	1 419 533 (1 355 252 to 1 481 102)	1 348 314 (1 277 129 to 1 415 359)	1 426 136 (1 295 290 to 1 561 249)	0 (−0.09 to 0.10)	0.06 (−0.03 to 0.16)	4816 (4594 to 5020)	3299 (3123 to 3467)	2745 (2491 to 3009)	−0.43 (−0.49 to −0.37)	−0.17 (−0.24 to −0.09)
Colorado	135 217 (128 875 to 142 051)	151 218 (143 317 to 159 496)	178 207 (164 511 to 193 087)	0.32 (0.22 to 0.43)	0.18 (0.10 to 0.27)	4212 (4015 to 4418)	2882 (2730 to 3040)	2502 (2308 to 2711)	−0.41 (−0.45 to −0.36)	−0.13 (−0.19 to −0.06)
Connecticut	184 329 (174 788 to 193 820)	144 635 (136 219 to 153 144)	142 014 (128 004 to 157 153)	−0.23 (−0.30 to −0.15)	−0.02 (−0.10 to 0.08)	4562 (4324 to 4799)	2938 (2761 to 3115)	2568 (2310 to 2846)	−0.44 (−0.49 to −0.37)	−0.13 (−0.20 to −0.04)
Delaware	40 801 (39 035 to 42 631)	42 605 (40 462 to 44 657)	46 634 (43 197 to 50 034)	0.14 (0.07 to 0.23)	0.09 (0.02 to 0.17)	5515 (5274 to 5766)	3788 (3597 to 3972)	3230 (2985 to 3466)	−0.41 (−0.46 to −0.37)	−0.15 (−0.21 to −0.09)
District of Columbia	48 023 (45 139 to 50 981)	33 900 (32 207 to 35 887)	29 434 (26 436 to 32 338)	−0.39 (−0.45 to −0.32)	−0.13 (−0.22 to −0.04)	7045 (6619 to 7482)	5070 (4814 to 5371)	3821 (3423 to 4209)	−0.46 (−0.51 to −0.39)	−0.25 (−0.32 to −0.17)
Florida	912 597 (871 318 to 955 778)	938 757 (890 152 to 990 403)	1 036 581 (945 964 to 1 127 932)	0.14 (0.05 to 0.23)	0.10 (0.02 to 0.19)	4825 (4605 to 5056)	3468 (3290 to 3658)	3046 (2773 to 3322)	−0.37 (−0.42 to −0.32)	−0.12 (−0.19 to −0.05)
Georgia	383 333 (364 736 to 404 195)	422 915 (402 599 to 443 265)	495 512 (448 477 to 547 458)	0.29 (0.16 to 0.43)	0.17 (0.07 to 0.30)	6069 (5782 to 6395)	4314 (4106 to 4515)	3855 (3486 to 4261)	−0.36 (−0.43 to −0.30)	−0.11 (−0.18 to −0.01)
Hawaii	47 905 (45 557 to 50 368)	52 621 (49 933 to 55 671)	58 809 (54 592 to 63 122)	0.23 (0.15 to 0.32)	0.12 (0.05 to 0.19)	4155 (3950 to 4366)	3004 (2850 to 3181)	2751 (2546 to 2963)	−0.34 (−0.38 to −0.29)	−0.08 (−0.15 to −0.02)
Idaho	48 004 (45 277 to 50 595)	55 036 (51 882 to 58 352)	65 778 (59 608 to 72 816)	0.37 (0.24 to 0.51)	0.20 (0.08 to 0.32)	4362 (4111 to 4599)	3157 (2975 to 3349)	2878 (2599 to 3193)	−0.34 (−0.41 to −0.27)	−0.09 (−0.18 to 0.01)
Illinois	729 700 (700 447 to 758 951)	586 576 (558 164 to 615 650)	583 809 (542 582 to 625 055)	−0.20 (−0.25 to −0.14)	0 (−0.07 to 0.06)	5593 (5365 to 5820)	3776 (3592 to 3960)	3276 (3045 to 3510)	−0.41 (−0.45 to −0.37)	−0.13 (−0.19 to −0.07)
Indiana	348 952 (332 367 to 364 783)	319 455 (302 691 to 338 262)	345 111 (311 560 to 382 080)	−0.01 (−0.11 to 0.09)	0.08 (−0.02 to 0.19)	5466 (5207 to 5716)	4057 (3842 to 4291)	3776 (3398 to 4190)	−0.31 (−0.38 to −0.23)	−0.07 (−0.16 to 0.03)
Iowa	177 351 (169 847 to 185 704)	141 801 (134 563 to 149 347)	145 688 (134 198 to 158 065)	−0.18 (−0.24 to −0.11)	0.03 (−0.05 to 0.11)	4695 (4487 to 4918)	3308 (3132 to 3485)	3062 (2813 to 3334)	−0.35 (−0.40 to −0.29)	−0.07 (−0.14 to 0)
Kansas	142 966 (135 734 to 150 353)	125 502 (118 490 to 132 286)	130 275 (117 197 to 143 618)	−0.09 (−0.18 to 0.01)	0.04 (−0.07 to 0.15)	4670 (4429 to 4909)	3475 (3281 to 3662)	3188 (2859 to 3529)	−0.32 (−0.39 to −0.24)	−0.08 (−0.18 to 0.02)
Kentucky	249 584 (239 516 to 260 047)	240 203 (229 017 to 250 977)	261 141 (241 901 to 281 046)	0.05 (−0.02 to 0.13)	0.09 (0.01 to 0.17)	5885 (5645 to 6132)	4510 (4300 to 4714)	4211 (3904 to 4536)	−0.28 (−0.33 to −0.23)	−0.07 (−0.13 to 0.01)
Louisiana	278 530 (267 499 to 290 522)	253 031 (242 116 to 265 036)	278 149 (259 576 to 297 612)	0 (−0.06 to 0.08)	0.10 (0.03 to 0.18)	6416 (6161 to 6685)	4893 (4675 to 5121)	4511 (4209 to 4841)	−0.30 (−0.34 to −0.24)	−0.08 (−0.14 to −0.01)
Maine	73 664 (70 512 to 76 954)	60 831 (57 568 to 64 137)	63 895 (59 206 to 68 758)	−0.13 (−0.19 to −0.06)	0.05 (−0.02 to 0.13)	4935 (4720 to 5162)	3139 (2970 to 3314)	2801 (2589 to 3018)	−0.43 (−0.47 to −0.39)	−0.11 (−0.17 to −0.04)
Maryland	266 272 (255 178 to 277 361)	257 224 (244 706 to 269 859)	270 454 (252 064 to 290 154)	0.02 (−0.06 to 0.09)	0.05 (−0.01 to 0.13)	5377 (5158 to 5598)	3777 (3589 to 3965)	3255 (3027 to 3499)	−0.39 (−0.44 to −0.35)	−0.14 (−0.19 to −0.08)
Massachusetts	344 926 (329 811 to 360 472)	263 564 (248 612 to 279 250)	265 984 (245 243 to 288 132)	−0.23 (−0.29 to −0.17)	0.01 (−0.06 to 0.08)	4713 (4501 to 4930)	3007 (2836 to 3191)	2613 (2398 to 2834)	−0.45 (−0.49 to −0.40)	−0.13 (−0.19 to −0.07)
Michigan	576 478 (553 769 to 599 201)	513 054 (490 607 to 536 292)	545 986 (510 193 to 584 379)	−0.05 (−0.11 to 0.02)	0.06 (0 to 0.14)	5640 (5420 to 5861)	3988 (3812 to 4173)	3714 (3461 to 3985)	−0.34 (−0.38 to −0.29)	−0.07 (−0.13 to 0)
Minnesota	216 269 (206 145 to 226 792)	172 124 (162 363 to 182 206)	187 696 (171 699 to 203 292)	−0.13 (−0.2 to −0.06)	0.09 (0.01 to 0.18)	4266 (4063 to 4478)	2603 (2454 to 2755)	2352 (2148 to 2552)	−0.45 (−0.49 to −0.40)	−0.10 (−0.16 to −0.02)
Mississippi	193 562 (185 083 to 202 896)	189 046 (180 264 to 198 433)	200 049 (179 924 to 220 022)	0.03 (−0.07 to 0.15)	0.06 (−0.05 to 0.17)	6785 (6480 to 7126)	5408 (5155 to 5678)	4982 (4475 to 5487)	−0.27 (−0.34 to −0.18)	−0.08 (−0.17 to 0.02)
Missouri	341 469 (326 761 to 355 657)	312 547 (296 795 to 328 007)	335 603 (312 125 to 358 105)	−0.02 (−0.08 to 0.05)	0.07 (0 to 0.15)	5356 (5129 to 5583)	4079 (3878 to 4283)	3806 (3538 to 4067)	−0.29 (−0.34 to −0.24)	−0.07 (−0.14 to 0)
Montana	41 761 (39 342 to 44 092)	42 137 (39 596 to 44 661)	46 011 (41 234 to 51 236)	0.10 (−0.01 to 0.22)	0.09 (−0.01 to 0.21)	4360 (4110 to 4605)	3177 (2987 to 3373)	2838 (2532 to 3170)	−0.35 (−0.42 to −0.28)	−0.11 (−0.20 to 0)
Nebraska	93 521 (89 492 to 97 875)	73 099 (69 187 to 76 979)	76 222 (70 668 to 81 841)	−0.18 (−0.24 to −0.13)	0.04 (−0.02 to 0.11)	4670 (4464 to 4897)	3095 (2924 to 3261)	2826 (2614 to 3039)	−0.39 (−0.44 to −0.35)	−0.09 (−0.15 to −0.02)
Nevada	67 463 (64 458 to 70 831)	116 195 (111 238 to 121 992)	138 242 (127 224 to 149 411)	1.05 (0.88 to 1.21)	0.19 (0.09 to 0.29)	5524 (5283 to 5792)	4057 (3882 to 4258)	3485 (3212 to 3764)	−0.37 (−0.42 to −0.32)	−0.14 (−0.21 to −0.07)
New Hampshire	57 141 (54 760 to 59 763)	50 784 (48 060 to 53 883)	55 515 (51 279 to 60 208)	−0.03 (−0.09 to 0.05)	0.09 (0.02 to 0.18)	4819 (4617 to 5041)	2964 (2805 to 3147)	2612 (2403 to 2840)	−0.46 (−0.50 to −0.41)	−0.12 (−0.18 to −0.05)
New Jersey	493 824 (472 733 to 516 372)	387 544 (366 588 to 409 963)	389 537 (357 117 to 422 716)	−0.21 (−0.27 to −0.15)	0.01 (−0.07 to 0.09)	5290 (5066 to 5532)	3383 (3196 to 3582)	2960 (2703 to 3217)	−0.44 (−0.49 to −0.39)	−0.12 (−0.19 to −0.05)
New Mexico	64 891 (61 707 to 68 460)	75 758 (71 560 to 80 242)	86 133 (77 073 to 95 519)	0.33 (0.18 to 0.47)	0.14 (0.03 to 0.26)	4248 (4039 to 4481)	3128 (2955 to 3318)	2897 (2585 to 3222)	−0.32 (−0.39 to −0.24)	−0.07 (−0.17 to 0.03)
New York	1 211 900 (1 159 815 to 1 268 126)	888 732 (841 668 to 937 980)	874 203 (788 838 to 964 687)	−0.28 (−0.35 to −0.20)	−0.02 (−0.11 to 0.08)	5658 (5410 to 5925)	3543 (3354 to 3742)	3056 (2749 to 3383)	−0.46 (−0.51 to −0.40)	−0.14 (−0.22 to −0.05)
North Carolina	430 072 (411 436 to 447 770)	434 895 (413 181 to 456 048)	493 351 (461 381 to 529 097)	0.15 (0.07 to 0.22)	0.13 (0.06 to 0.21)	5819 (5567 to 6055)	3962 (3765 to 4154)	3493 (3267 to 3748)	−0.40 (−0.44 to −0.36)	−0.12 (−0.18 to −0.06)
North Dakota	35 950 (34 121 to 37 825)	28 372 (26 900 to 29 915)	29 767 (27 010 to 32 684)	−0.17 (−0.24 to −0.09)	0.05 (−0.05 to 0.15)	4465 (4237 to 4709)	3060 (2896 to 3225)	2861 (2583 to 3152)	−0.36 (−0.42 to −0.29)	−0.06 (−0.16 to 0.03)
Ohio	708 366 (681 145 to 739 364)	611 429 (581 107 to 640 071)	628 546 (587 335 to 671 386)	−0.11 (−0.17 to −0.05)	0.03 (−0.04 to 0.10)	5558 (5345 to 5802)	4019 (3821 to 4210)	3674 (3426 to 3929)	−0.34 (−0.38 to −0.29)	−0.09 (−0.15 to −0.02)
Oklahoma	218 465 (209 890 to 227 515)	219 457 (209 424 to 229 268)	237 754 (221 868 to 253 051)	0.09 (0.02 to 0.16)	0.08 (0.01 to 0.15)	5774 (5541 to 6020)	4790 (4572 to 5012)	4512 (4207 to 4816)	−0.22 (−0.27 to −0.16)	−0.06 (−0.12 to 0.01)
Oregon	163 349 (155 956 to 170 501)	152 113 (144 369 to 159 935)	162 611 (150 597 to 173 855)	0 (−0.07 to 0.07)	0.07 (0 to 0.14)	4694 (4481 to 4901)	3067 (2907 to 3229)	2638 (2441 to 2825)	−0.44 (−0.48 to −0.40)	−0.14 (−0.20 to −0.08)
Pennsylvania	863 452 (825 830 to 901 277)	684 109 (652 518 to 718 402)	669 427 (622 590 to 716 637)	−0.22 (−0.27 to −0.17)	−0.02 (−0.08 to 0.04)	5468 (5234 to 5708)	3748 (3568 to 3945)	3307 (3068 to 3549)	−0.40 (−0.43 to −0.35)	−0.12 (−0.18 to −0.06)
Rhode Island	63 951 (60 728 to 67 202)	50 118 (47 462 to 52 770)	47 060 (42 729 to 52 039)	−0.26 (−0.33 to −0.19)	−0.06 (−0.15 to 0.04)	4922 (4670 to 5178)	3318 (3136 to 3498)	2864 (2590 to 3176)	−0.42 (−0.47 to −0.36)	−0.14 (−0.22 to −0.04)
South Carolina	233 955 (223 544 to 245 019)	239 304 (227 372 to 252 251)	281 201 (255 863 to 308 170)	0.2 (0.09 to 0.33)	0.18 (0.07 to 0.29)	6394 (6108 to 6694)	4386 (4166 to 4622)	3988 (3631 to 4374)	−0.38 (−0.44 to −0.31)	−0.09 (−0.17 to 0)
South Dakota	41 571 (39 490 to 43 736)	35 253 (33 193 to 37 208)	38 171 (34 862 to 41 689)	−0.08 (−0.16 to 0.01)	0.08 (−0.01 to 0.18)	4643 (4406 to 4891)	3247 (3058 to 3430)	3011 (2739 to 3304)	−0.35 (−0.41 to −0.28)	−0.07 (−0.16 to 0.01)
Tennessee	342 436 (327 680 to 356 040)	353 579 (337 242 to 370 112)	401 757 (373 136 to 429 060)	0.17 (0.09 to 0.25)	0.14 (0.05 to 0.21)	6062 (5802 to 6306)	4618 (4403 to 4833)	4330 (4011 to 4627)	−0.29 (−0.34 to −0.24)	−0.06 (−0.13 to 0)
Texas	874 588 (836 443 to 917 058)	959 876 (912 337 to 1 006 742)	1 138 668 (1 048 585 to 1 225 207)	0.30 (0.20 to 0.41)	0.19 (0.10 to 0.27)	5330 (5099 to 5590)	3893 (3701 to 4084)	3473 (3199 to 3736)	−0.35 (−0.40 to −0.30)	−0.11 (−0.18 to −0.05)
Utah	59 956 (57 274 to 62 829)	70 601 (66 680 to 74 999)	86 921 (80 925 to 93 731)	0.45 (0.35 to 0.55)	0.23 (0.16 to 0.32)	4169 (3986 to 4366)	3006 (2839 to 3198)	2770 (2579 to 2991)	−0.34 (−0.38 to −0.29)	−0.08 (−0.13 to −0.01)
Vermont	29 418 (28 062 to 30 855)	24 546 (23 171 to 25 952)	26 669 (24 653 to 28 926)	−0.09 (−0.16 to −0.02)	0.09 (0.01 to 0.17)	4741 (4524 to 4974)	2852 (2688 to 3021)	2595 (2399 to 2823)	−0.45 (−0.49 to −0.41)	−0.09 (−0.15 to −0.02)
Virginia	338 350 (324 960 to 352 462)	328 352 (312 057 to 344 310)	355 048 (330 606 to 379 379)	0.05 (−0.02 to 0.12)	0.08 (0.01 to 0.15)	5329 (5120 to 5551)	3572 (3395 to 3746)	3096 (2876 to 3311)	−0.42 (−0.46 to −0.38)	−0.13 (−0.19 to −0.07)
Washington	238 207 (226 974 to 249 651)	240 521 (227 557 to 252 774)	266 486 (246 955 to 286 257)	0.12 (0.04 to 0.20)	0.11 (0.04 to 0.19)	4491 (4279 to 4709)	3072 (2903 to 3228)	2647 (2450 to 2849)	−0.41 (−0.45 to −0.37)	−0.14 (−0.19 to −0.08)
West Virginia	142 310 (136 548 to 148 703)	117 927 (112 414 to 123 650)	120 928 (113 164 to 129 817)	−0.15 (−0.21 to −0.08)	0.03 (−0.04 to 0.10)	6067 (5812 to 6347)	4442 (4232 to 4654)	4204 (3923 to 4521)	−0.31 (−0.35 to −0.25)	−0.05 (−0.12 to 0.02)
Wisconsin	282 970 (271 112 to 294 586)	238 667 (227 253 to 250 715)	254 635 (237 018 to 272 402)	−0.1 (−0.16 to −0.04)	0.07 (0 to 0.14)	4791 (4594 to 4989)	3213 (3055 to 3376)	2932 (2726 to 3144)	−0.39 (−0.43 to −0.34)	−0.09 (−0.15 to −0.03)
Wyoming	20 201 (19 177 to 21 342)	22 184 (20 904 to 23 352)	24 008 (21 572 to 26 595)	0.19 (0.07 to 0.32)	0.08 (−0.02 to 0.20)	4479 (4252 to 4735)	3398 (3202 to 3578)	3011 (2698 to 3343)	−0.33 (−0.40 to −0.26)	−0.11 (−0.20 to −0.02)

Abbreviation: UI, uncertainty interval.

### Geographic Variation in Total and Cause-Specific CVD Burden in 2016

There was wide geographic variation in the age-standardized CVD burden among US states in 2016, with the greatest burden concentrated in a band of states extending from the Gulf Coast to West Virginia. The highest rate of CVD DALYs was in Mississippi (4982 age-standardized DALYs per 100 000 persons; 95% UI, 4475-5487), followed by Arkansas, Oklahoma, Louisiana, Alabama, Tennessee, Kentucky, West Virginia, South Carolina, and Georgia (Table). Notably, several states outside this region had levels of CVD DALYs nearly as high, including Indiana, Missouri, Ohio, Michigan, North Carolina, Nevada, and Texas. The lowest rate of CVD DALYs was in Minnesota (2352 age-standardized DALYs per 100 000 persons; 95% UI, 2148-2552), followed by Colorado and areas of New England and the Pacific Northwest, including Massachusetts, New Hampshire, Washington, Connecticut, Vermont, and Oregon. Ischemic heart disease was the leading cause of age-standardized CVD DALYs in all states and the District of Columbia (eFigure 1 in the Supplement). The second-leading CVD cause was ischemic stroke in all states. The proportion of DALYs due to YLD (as opposed to YLL) ranged from 10% (in Mississippi) to 18% (in Connecticut) (eFigure 2 in the Supplement).

### Age and Sex Disparities in Total and Cause-Specific CVD Burden in 2016

**Figure 3.**
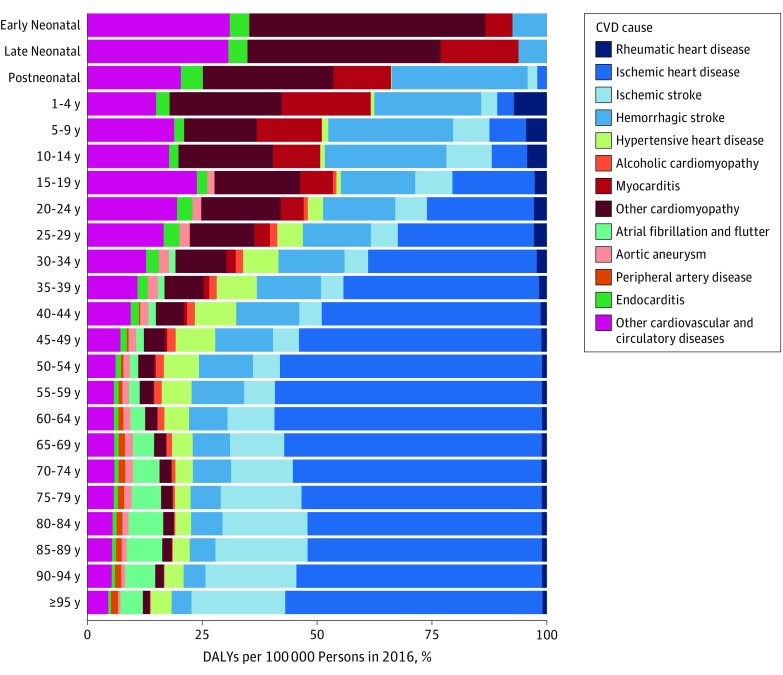
Percentage of Disability-Adjusted Life-Years (DALYs) per 100 000 Persons for Cardiovascular Disease (CVD) Causes by Age in 2016

In 2016 in the United States, CVD as a proportion of all DALYs increased with age rapidly after age 40 years, rising to account for 20% of all DALY burden by age 65 years. The largest cause of CVD in the first year of life was cardiomyopathy (Figure 3). Hemorrhagic stroke accounted for an increasingly larger amount of CVD DALYs from age 1 to 14 years and then decreased slowly with increasing age, while ischemic stroke increased rapidly as a cause of CVD after age 60 years. Ischemic heart disease was the dominant source of CVD DALYs after age 40 years. Atrial fibrillation became an increasingly common cause of CVD burden for those 65 years and older. As noted above, estimates of heart failure have been disaggregated into their underlying cause in this analysis.

**Figure 4.**
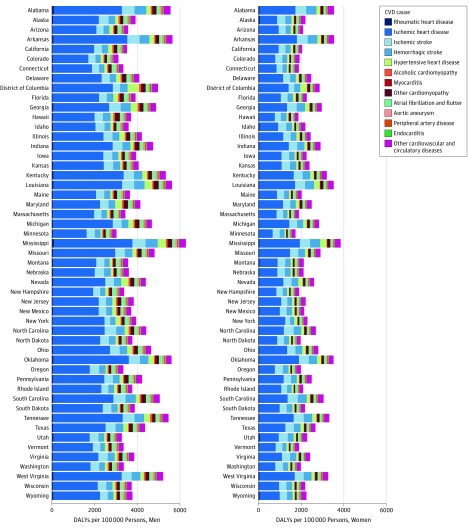
Age-Standardized Cardiovascular Disease (CVD) Disability-Adjusted Life-Years (DALYs) per 100 000 Persons by US State in 2016

Large disparities between men and women existed for total CVD burden in 2016 (Figure 2C and D). Cardiovascular disease burden was generally twice as great for men compared with women in all states for ischemic heart disease, cardiomyopathy and myocarditis, and aortic aneurysm (Figure 4). While the patterns of states with higher and lower rates of age-standardized CVD DALYs are similar, the age-standardized rate for women is lower for every state. For example, the rate of CVD DALYs in Mississippi among women (3581 age-standardized DALYs per 100 000 persons; 95% UI, 3285-4399), the highest rate experienced by women in any state, is similar to the rate of CVD DALYs among men in Florida, Virginia, and South Dakota. The largest absolute difference in CVD DALY rates was observed between Minnesota and Mississippi for both men and women; however, this gap between the lowest and highest rates of CVD DALYs was much larger for men than women (3249 vs 2115 age-standardized DALYs per 100 000 persons). As noted above, estimates of heart failure have been disaggregated to their underlying cause in this analysis.

### Geographic Variation in Heart Failure Prevalence

**Figure 5.**
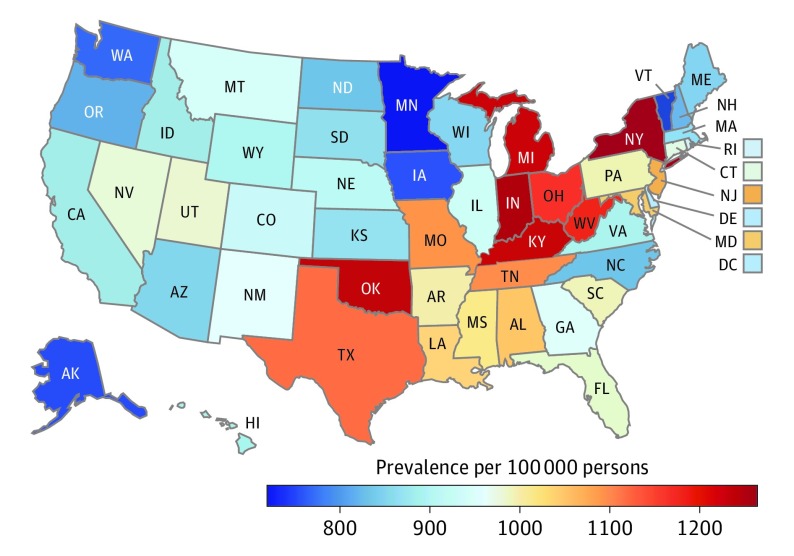
Age-Standardized Prevalence of Heart Failure per 100 000 Persons in 2016 in Both Sexes

Age-standardized heart failure prevalence was greatest in many Midwestern and Eastern states and was least across the northern Great Plains and Western states (Figure 5; eTable 2 in the Supplement). New York had the greatest age-standardized prevalence rate for heart failure in 2016 (1319 cases per 100 000 persons; 95% UI, 1277-1350), followed by Indiana, Oklahoma, Kentucky, Michigan, West Virginia, and Ohio. Heart failure was least prevalent in Minnesota (760 cases per 100 000 persons; 95% UI, 702-827), with similarly low rates in Washington, Vermont, and Iowa.

### Attribution of Total CVD to Risk Factors for Each State

**Figure 6.**
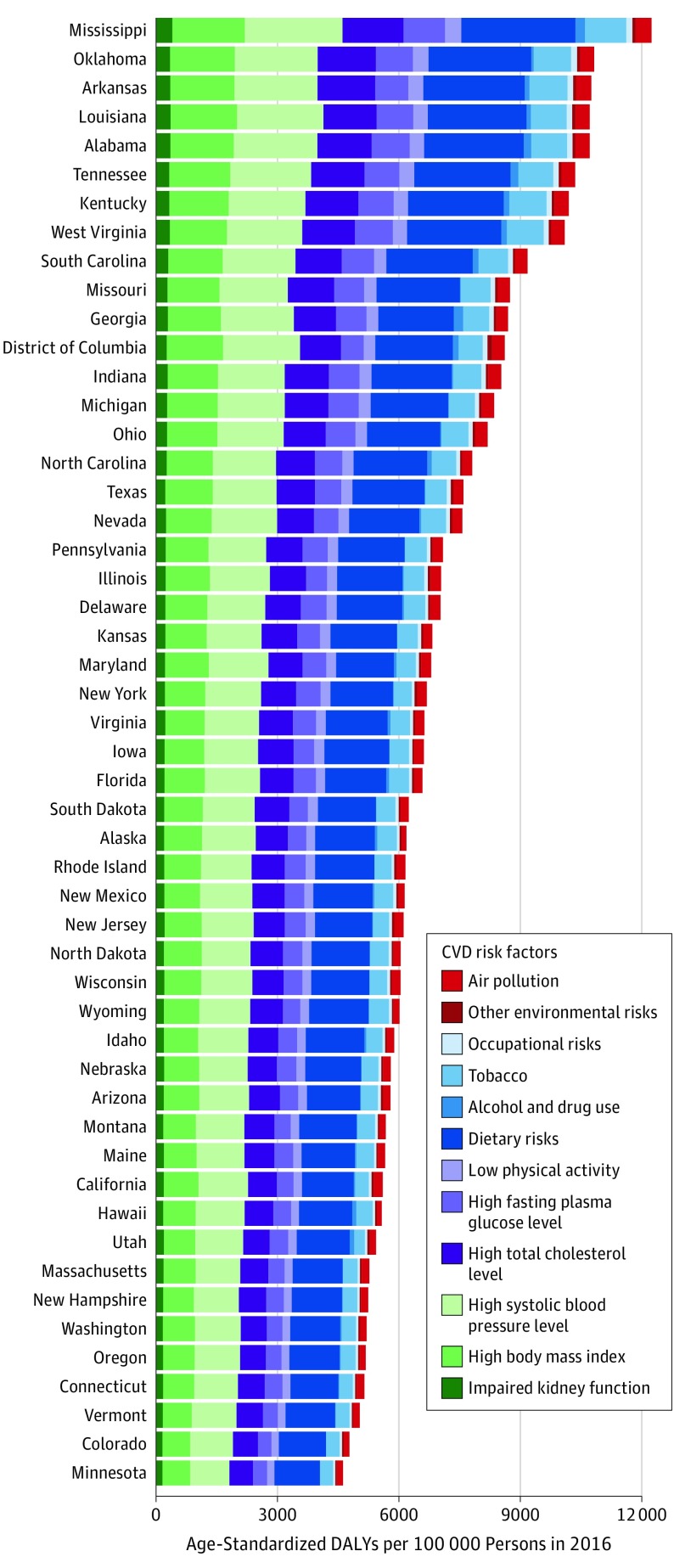
Age-Standardized Cardiovascular Disease (CVD) Disability-Adjusted Life-Years (DALYs) per 100 000 Persons Attributable to Risk Factors in 2016

For almost all states, the greatest proportion of age-standardized CVD DALYs was attributable to dietary risk factors, followed by high systolic blood pressure, high body mass index, high total cholesterol level, high fasting plasma glucose level, tobacco smoking, and low levels of physical activity (Figure 6). Notable risks that made up smaller proportions of CVD DALYs were ambient air particulate matter, impaired kidney function, and alcohol use. As an example, eFigure 3 in the Supplement shows the relative change in rank position for magnitude of the attributable age-standardized CVD DALY rate for risk factors in Mississippi and Minnesota. While dietary risks and elevated systolic blood pressure were leading risk factors for CVD in both Mississippi and Minnesota in both 1990 and 2016, high body mass index became a greater contributor and tobacco smoking became a lesser contributor to CVD burden over time.

### Drivers of Changes in Risk-Attributable DALYs

**Figure 7.**
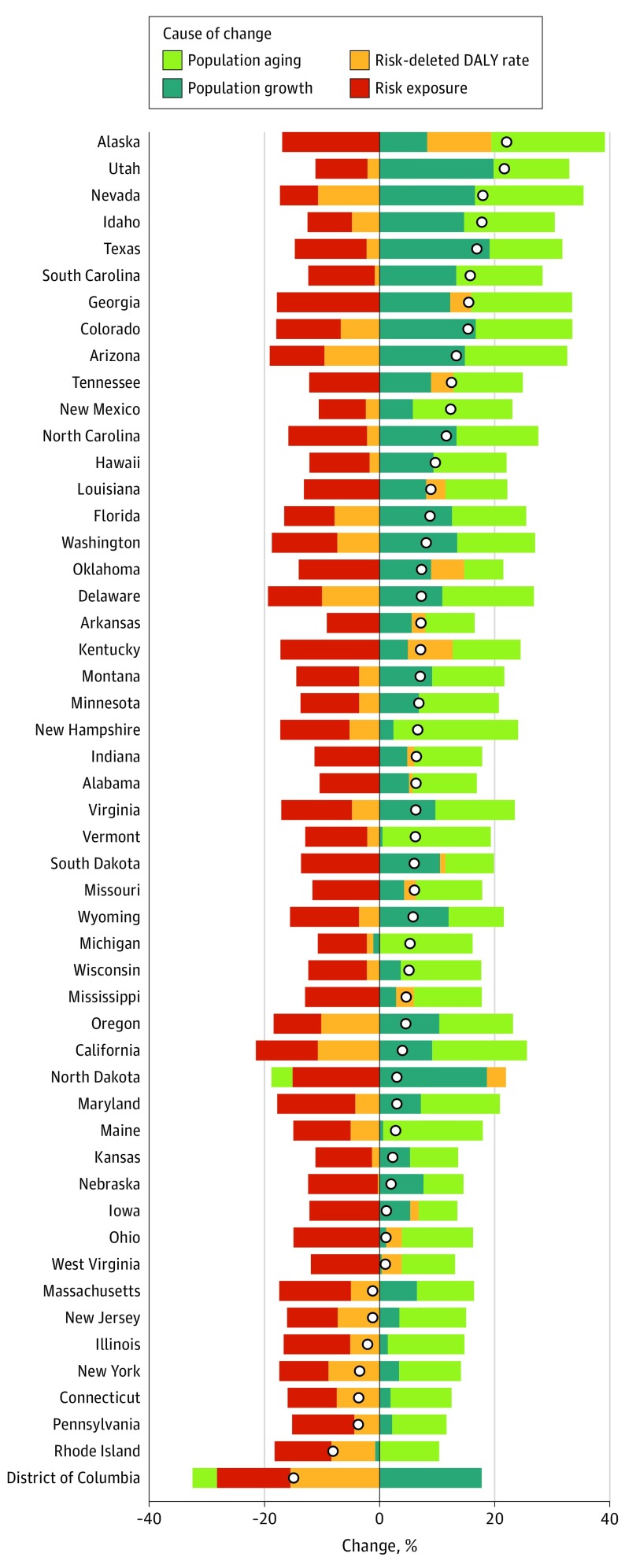
US State Drivers of Change in Cardiovascular Disease (CVD) Disability-Adjusted Life-Years (DALYs) From 2006 to 2016 Drivers explored in this analysis include population growth, population aging, trends in exposure to all risks included in the Global Burden of Disease 2016 Study, and all other unmeasured factors combined. Results are shown for all CVD DALYs by state. The circle on the bar graph indicates the total percentage change.

Figure 7 shows the relative contributions of 4 mutually exclusive drivers of the observed change in CVD DALYs from 2006 to 2016 for each state: population growth, population aging, trends in exposure to all CVD risk factors measured in the GBD Study 2016,^1^ and all other unmeasured factors combined. The change from 1990 to 2016 is shown in eFigure 4 in the Supplement. Most states had an increase in CVD DALYs during this time despite all states experiencing a decrease in CVD-related risk exposures. Population aging and population growth accounted for most of this increase. Notably, the residual category of unmeasured factors, which would account for health care–related treatment and any other exposures not included in the GDB Study 2016 evaluation of traditional CVD risk factors, explained increases in many states, suggesting that unmeasured risk exposures are increasing the burden of CVD in many parts of the United States.

## Discussion

Large disparities in total burden of CVD persist between US states despite marked improvements in CVD burden. We found that it took 25 years for states with the largest burden of CVD to achieve levels observed among the healthiest states in 1990. States with the highest burden of CVD in 1990, such as Kentucky, West Virginia, Alabama, Arkansas, Louisiana, Tennessee, and Oklahoma, are only now achieving the 1990 levels of CVD burden in Massachusetts, Connecticut, and New Jersey. Mississippi continues to lag as the state with the largest CVD burden in the United States. These findings support the idea that tremendous gains in cardiovascular health are possible even in states with lower socioeconomic levels but that relative disparities between states have changed very little.^22^ These relative disparities may be of particular concern for Alabama, Mississippi, Oklahoma, and Tennessee, given their recent decision to not expand their respective Medicaid systems.^23^

We found increases in the total burden of CVD in 12 states from 2010 to 2016 (eFigure 5 in the Supplement). Several studies have noted increasing all-cause mortality for selected subgroups or regions of the United States. Life expectancy has been decreasing among women in some counties.^6^ It has been suggested that increasing body mass index will result in decreasing life expectancy in the United States.^24^ Our finding of increasing CVD burden is concerning and suggests that long-term decline in CVD may be ending. New clinical or public health interventions delivered earlier in the life course may be required to alter this alarming trajectory.

The District of Columbia, a small urban area tracked separately, is a notable outlier that demonstrates the potential for improvements in the burden of CVD for cities. This region experienced marked improvements in socioeconomic status since 1990, as reflected by our summary measure of SDI. The District of Columbia also experienced particularly rapid declines in CVD. The causal relationship between socioeconomic status and health has been well described.^25^ Also, wide variation in the rate of change for cardiovascular mortality has been shown for small geographic regions, such as counties.^26^ Migration of healthier individuals into the District of Columbia and states or migration of sicker individuals out of these locations may have also contributed to changes in CVD burden.

An intriguing finding of our study was that socioeconomic status did not fully explain a population’s level of CVD burden or risk factors. States with lower rates of CVD burden were found across the full range of SDI. Prior research has suggested a causal association between higher altitude and lower CVD mortality, which could explain lower CVD burden in some Mountain and Southwestern states.^27^ Variation in health care quality between states, another possible explanation, has been less well documented than variation between specific hospitals or health care referral regions but may be substantial. Finally, some aspects of socioeconomic status may not be well accounted for by our index, such as wealth (as opposed to income per capita) or attained level of maternal education.

Research and policy have focused extensively on race and ethnicity as independent risk factors for CVD in the United States.^28^ The GBD 2016 Study did not stratify health by these categories, and a full discussion of this important topic is beyond the scope of this article. For example, disparities attributed to race may in fact reflect differences in access to high-quality health care or genetic factors.^29^ The concentration of CVD burden in states with higher proportions of individuals that identify their race as black/African American or American Indian/Alaska Native is a well-known observation.^30,31,32^ The association of race/ethnicity and risk are complicated by the observation that self-reported race/ethnicity differs from genetic background. Furthermore, reported risk associations have differed for various regions of the country, suggesting effect modification by local factors.^33,34^ The addition of race-specific and ethnicity-specific state-level estimates is an important goal for future iterations of the GBD Study and will allow for further exploration of these issues.

Diseases caused wholly or in part by atherosclerotic vascular disease (IHD, stroke, peripheral artery disease, or aortic aneurysm) accounted for the largest portion of CVD in all states. Most of this burden was due to IHD. As noted above, estimates of heart failure were disaggregated to their underlying cause in this analysis, including IHD; burden due only to heart failure is not reported. More than 80% of CVD burden could be attributed to known modifiable risk factors. The prevention of CVD through the reduction of these well-known risk factors remains a major public health goal for the United States.^35^ Clinical trials have shown that medications should target lower levels of systolic blood pressure and plasma low-density lipoprotein cholesterol for many patients.^36,37,38,39^ Tobacco cessation also remains a major target for health systems and local governments.^40^ In addition to these clinical and public health goals, our analysis shows that a large proportion of CVD can also be attributed to dietary risks, high body mass index, and low physical activity. Notably, air pollution has continued to decrease in terms of its relative contribution to CVD in the United States.^41^ Both rheumatic heart disease and endocarditis account for a small but persistent proportion of CVD.

### Limitations

Our study has limitations. All estimates have been reported as a mean value with an estimate of uncertainty. Given the combination of diverse data sources used to produce these results, the 95% uncertainty range is an important feature of our analysis that should be considered whenever interpreting a particular point estimate. Our nonfatal modeling process has improved significantly over the lifetime of the GBD Study, yet several challenges remain, including incorporation of uncertainty because of using multiple nonreference case definitions (such as cohort and claims data), quantifying the generalizability of claims data, identifying additional data on disease severity, accounting for the interdependence of comorbidities, and moving from cross-sectional estimation to a method that accounts for birth cohort effects.

Our analysis has several specific limitations. First, estimates at the level of US states represent an aggregate across a range of substate geographies, such as counties and urban vs rural areas. State-level estimates remain important given that many policy decisions continue to be made uniformly at the state level; however, further analysis is needed to examine differences between other geographic categories, such as urban and rural regions. Bias in death certification related to CVD has been demonstrated.^42^ Our results correct for some of this bias by adjusting for the use of nonspecific and intermediate *ICD* codes. Second, for this analysis, we applied a method to account for the variable use between states of nonspecific or intermediate causes on death certificates. Other biases in death certification are more difficult to correct in a state-specific manner, such as the common coding of death to *ICD *code I64 (stroke, unspecified type), which were reassigned to stroke subtypes using the same ratio of subtypes for each state.^43^ Third, our source for administrative health care data was limited to a database of mostly commercial payers, which may underestimate incidence and prevalence of disease if those with employer-based health plans are healthier than the general population. Incidence data for CVD is particularly limited in the United States. These claims data are available at the individual level for inpatient and ambulatory care and for all ages in all states across multiple years but do not include care covered solely by Medicare. Prevalence of CVD may be lower among those with private, employee-based insurance than those using Medicare or without health insurance. Administrative data were used only for conditions where it was felt to be reliable based on validation studies and expert opinion; however, some degree of misclassification bias, with both overestimation and underestimation, is likely from these sources. Expanding the GBD Study to include Medicare data is an important goal for future versions of the study. Fourth, our attribution of CVD burden to modifiable risk factors assumes a theoretical minimum level of exposure rather than the treatment goal of any set of existing intervention. For example, we rely on data from prospective cohort studies and clinical trials to set the minimum level of systolic blood pressure at 110 to 115 mm Hg. This level was selected because it is at this level that the lowest association with CVD outcomes is observed.^44^ We do not make any assumptions (or provide any estimates of health gains) regarding a specific level to be targeted by antihypertensive medication. Furthermore, while there is good evidence for causality for each risk and paired CVD outcome, the precise causes in any given population cannot be known with certainty. Fifth, estimates of prevalent heart failure are based on administrative claims data and carry the assumption that all cases presented to hospitals. Subclinical heart failure is therefore not included in these estimates. Sixth, our study is, by design, cross-sectional and therefore descriptive and hypothesis-generating rather than the basis for causal claims. Finally, this analysis does not report CVD due to 2 cardiovascular conditions, Chagas cardiomyopathy and congenital heart disease. Several hundred thousand cases of Chagas cardiomyopathy may be present in the United States.^45^ Estimates for disease burden due to congenital heart disease in the United States have been reported previously.^46^

## Conclusions

Cardiovascular disease is a major cause of lost health in the United States but varies widely in level among states. Most CVD burden in the United States is from atherosclerotic vascular disease, and 80% can be attributed to known causal risk factors. We found that CVD burden has improved for all states, but the rate of decline varies widely and is strongly associated with an index of socioeconomic level. For 12 states, CVD burden has increased since 2010. These estimates can provide a benchmark for states working to focus on key risk factors, improve health care quality, and lower health care costs.

## Notes

Supplement. **eMethods 1.** Global Burden of Disease overview. **eMethods 2.** Outcomes estimations. **eMethods 3.** Fatal cause-specific estimation process. **eMethods 4.** Nonfatal modeling methods. **eMethods 5.** Risk factor cause-specific estimation process. **eTable 1.** Disability-adjusted life-years (DALYs) and percentage change of DALYs for all cardiovascular causes by US state, total number and age-standardized rate for 1990, 2006, and 2016 for both sexes. **eTable 2.** Age-standardized heart failure prevalence per 100 000 persons for 2016. **eFigure 1.** US State rankings for age-standardized cardiovascular disease disability-adjusted life-year rates per 100 000 persons for both sexes combined in 2016. **eFigure 2.** Proportion of cardiovascular disease disability-adjusted life-years due to years lived with disability in 2016. **eFigure 3.** Leading level 2 cardiovascular risk factors for both sexes for Minnesota and Mississippi. **eFigure 4.** US State drivers of change in cardiovascular disease from 1990 to 2016. **eFigure 5.** Age-standardized percentage change in disability-adjusted life-year rate between 2010 and 2016 for all cardiovascular diseases.
