# Supplementary material for: The Burden of Cardiovascular Diseases Among US States, 1990-2016
Source: JAMA Cardiol. 2018 Apr 11;3(5):375–89. doi: 10.1001/jamacardio.2018.0385 (PMC6145754; doi:10.1001/jamacardio.2018.0385)
Supplement: Supplement. — eMethods 1. Global Burden of Disease overview. eMethods 2. Outcomes estimations. eMethods 3. Fatal cause-specific estimation process. eMethods 4. Nonfatal modeling methods. eMethods 5. Risk factor cause-specific estimation process. eTable 1. Disability-adjusted life-years (DALYs) and percentage change of DALYs for all cardiovascular causes by US state, total number and age-standardized rate for 1990, 2006, and 2016 for both sexes. eTable 2. Age-standardized heart failure prevalence per 100 000 persons for 2016. eFigure 1. US State rankings for age-standardized cardiovascular disease disability-adjusted life-year rates per 100 000 persons for both sexes combined in 2016. eFigure 2. Proportion of cardiovascular disease disability-adjusted life-years due to years lived with disability in 2016. eFigure 3. Leading level 2 cardiovascular risk factors for both sexes for Minnesota and Mississippi. eFigure 4. US State drivers of change in cardiovascular disease from 1990 to 2016. eFigure 5. Age-standardized percentage change in disability-adjusted life-year rate between 2010 and 2016 for all cardiovascular diseases. [file jamacardiol-3-375-s001.pdf]

Supplementary Online Content

Global Burden of Cardiovascular Diseases Collaboration. The burden of cardiovascular diseases among US states, 1990-2016. *JAMA Cardiol*. Published online April 11, 2018. doi:10.1001/jamacardio.2018.0385

eMethods 1. Global Burden of Disease overview

eMethods 2. Outcomes estimations

eMethods 3. Fatal cause-specific estimation process

eMethods 4. Nonfatal modeling methods

eMethods 5. Risk factor cause-specific estimation process

eTable 1. Disability-adjusted life-years (DALYs) and percentage change of DALYs for all cardiovascular causes by US state, total number and age-standardized rate for 1990, 2006, and 2016 for both sexes

eTable 2. Age-standardized heart failure prevalence per 100 000 persons for 2016

eFigure 1. US State rankings for age-standardized cardiovascular disease disability-adjusted life-year rates per 100 000 persons for both sexes combined in 2016

eFigure 2. Proportion of cardiovascular disease disability-adjusted life-years due to years lived with disability in 2016

eFigure 3. Leading level 2 cardiovascular risk factors for both sexes for Minnesota and Mississippi

eFigure 4. US State drivers of change in cardiovascular disease from 1990 to 2016

eFigure 5. Age-standardized percentage change in disability-adjusted life-year rate between 2010 and 2016 for all cardiovascular diseases

This supplementary material has been provided by the authors to give readers additional information about their work.

© 2018 Global Burden of Cardiovascular Diseases Collaboration. *JAMA Cardiology*.

eMethods 1. Global Burden of Disease Overview

a. GATHER statement

This study is in compliance with the Guidelines for Accurate and Transparent Health Estimates Reporting (GATHER) recommendations. The GBD 2016 capstone papers and their respective supplementary documents contain the general methods, data sources, model selection information, performance and limitation information for the GBD 2016 analyses including detailed GATHER documentation[1,2,3]. Appendix Table 1 contains GATHER compliance information for this publication.

b. GBD Cause List

The GBD Cause List is organized into six levels, consisting of a hierarchy that is mutually exclusive and collectively exhaustive. Details on the overall GBD Cause list have been documented elsewhere[1,2]. Appendix Table 2 contains the cause and sequelae list for Cardiovascular Diseases.

c. Socio-Demographic Index (SDI)

The Socio-demographic Index (SDI) is a composite indicator of development status constructed for GBD 2015 whose components are strongly correlated with health outcomes. SDI was calculated using the Human Development Index (HDI) methodology, wherein an index value was determined for each of the covariate inputs (log LDI, mean educational attainment over age 15, and TFR). Detailed methodology and analysis information for SDI have been described elsewhere[1,2].

d. Data Sources

A complete list of sources used in the GBD 2016 analyses is available from the GBD 2016 Data Input Sources Tool (http://ghdx.healthdata.org/gbd-2016/data-input-sources).

© 2018 Global Burden of Cardiovascular Diseases Collaboration. *JAMA Cardiology*.

eMethods 2. Outcomes estimations

Hospital and Claims Data

Hospital data plays a key role in nonfatal estimation for many CVD causes. GBD 2016 used both inpatient and outpatient administrative claims data. Detailed methods for claims data analysis from the United States were described previously[2]. Briefly, aggregate data was derived from claims information in the Truvan Marketscan database of US private and public health insurance and were incorporated for the years of 2000, 2010, and 2012. Populations covered in each year were 3.3 million, 40.4 million and 40.8 million respectively. all ICD-9 four- or five-digit-coded diagnoses were mapped to GBD Causes. GBD conditions were categorized as “long-term” or “short-term” depending on cause duration. In a given year, for each individual in the claims data, a long-term case was defined as any mention in any diagnostic field associated with any claim, including inpatient and outpatient encounters. A short-term case was defined the same way, but assumed that claims within a condition-specific duration were the same case. A correction factor was applied to account for bias in health service encounter data over time, with the assumption that data from 2012 was most representative of the entire population.

© 2018 Global Burden of Cardiovascular Diseases Collaboration. *JAMA Cardiology*.

eMethods 3. Fatal cause-specific estimation process

Fatal estimates for cardiovascular diseases were generated using CODEm. The CODEm methods approach has been described elsewhere[1]. A list of covariates used in CODEm modeling for each CVD cause can be found in Appendix Table 3a.

ICD8, 9, and 10 codes were mapped to GBD causes. Nonspecific or intermediate causes of death inappropriately assigned as underlying causes of death were redistributed to appropriate underlying causes using an algorithm developed for the GBD study. After identifying nonspecific or intermediate codes (for example generalized atherosclerosis or left-sided heart failure), a regression model was used to reassign these codes to biologically plausible targets. All-cause, all-cardiovascular, and cause-specific mortality was estimated using the Cause of Death Ensemble Model (CODEm) which produces cause-specific smoothed trends over time by age, sex, and state. Atrial fibrillation mortality was estimated with a separate natural history model described below. The CODCorrect algorithm was applied to ensure that cause-specific, cardiovascular, and all-cause deaths were consistent. Years of life lost (YLLs) were computed by multiplying the number of deaths from each cause in each age group by a global reference life expectancy at the average of age of death among those who died in the age group.

© 2018 Global Burden of Cardiovascular Diseases Collaboration. *JAMA Cardiology*.

eMethods 4. Nonfatal modeling methods

Nonfatal estimates for cardiovascular diseases were modeled using the DisMod-MR 2.1 platform. Morbidity modeling methods have been documented elsewhere[2]. A list of covariates used in DisMod modeling for each CVD cause can be found in Appendix Table 3b. Appendix Table 4 includes a list of International Classification of Diseases (ICD) codes used in the extraction of hospital and claims data, mapped to specific cardiovascular diseases.

© 2018 Global Burden of Cardiovascular Diseases Collaboration. *JAMA Cardiology*.

eMethods 5. Risk factor cause-specific estimation process

A set of behavioral, environmental and occupational, and metabolic risks that contribute to health outcomes were evaluated in GBD 2016. The Comparative Risk Assessment framework included 84 behavioral, environmental and occupational, and metabolic risks or risk clusters. Risk-outcome pairs were defined using the World Cancer Research Fund-defined criteria for convincing or probable evidence. Relative risk estimates were derived from published and unpublished data, including randomized trials and pooling of longitudinal cohort studies. Both Bayesian meta-regression and Gaussian spatiotemporal process regression models were used to produce consistent estimates of risk exposure.

Risks were organized in four hierarchical levels, each level being evaluated to determine whether risk combinations were additive, multiplicative, or shared common pathways for intervention. Through this method, we are able to quantify the proportion of risk attributable burden shared by risks or combination of risks. Additionally, this methodology allows for the measurement of potential overlaps between behavioral, environmental and occupational, and metabolic risks. The full risk factor estimation and evaluation methodology has been described elsewhere[3].

© 2018 Global Burden of Cardiovascular Diseases Collaboration. *JAMA Cardiology*.

eReferences.

1. GBD 2016 Causes of Death Collaborators. Global, regional, and national age-sex specific mortality for 264 causes of death, 1980–2016: a systematic analysis for the Global Burden of Disease Study 2016. The Lancet. 2017: 390;1151–210.

2. GBD 2016 Disease and Injury Incidence and Prevalence Collaborators. Global, regional, and national incidence, prevalence, and years lived with disability for 328 diseases and injuries for 195 countries, 1990–2016: a systematic analysis for the Global Burden of Disease Study 2016. The Lancet. 2017: 390; 1211–59.

3. GBD 2016 Risk Factors Collaborators. Global, regional, and national comparative risk assessment of 84 behavioural, environmental and occupational, and metabolic risks or clusters of risks, 1990–2016: a systematic analysis for the Global Burden of Disease Study 2016. The Lancet. 2017: 390; 1345-1422.

© 2018 Global Burden of Cardiovascular Diseases Collaboration. *JAMA Cardiology*.

Appendix Table 1. GATHER checklist of information that should be included in reports of global health estimates, with description of compliance and location of information for "The burden of cardiovascular diseases among US states, 1990–2016".

#	GATHER checklist item	Description of compliance	Reference
**Objectives and funding**			
1	Define the indicators, populations, and time periods for which estimates were made.	Narrative provided in paper and appendix describing indicators, definitions, and populations.	Manuscript; Methods Appendix, Section 1. GBD Overview
2	List the funding sources for the work.	Funding sources listed at end of paper.	Funding Sources
**Data Inputs**			
For all data inputs from multiple sources that are synthesized as part of the study:			
3	Describe how the data were identified and how the data were accessed.	Narrative description of data seeking methodology provided in previously published appendices.	1. GBD 2016 Causes of Death Collaborators. Global, regional, and national age-sex specific mortality for 264 causes of death, 1980–2016: a systematic analysis for the Global Burden of Disease Study 2016. The Lancet. 2017: 390;1151–210.<br>2. GBD 2016 Disease and Injury Incidence and Prevalence Collaborators. Global, regional, and national incidence, prevalence, and years lived with disability for 328 diseases and injuries for 195 countries, 1990–2016: a systematic analysis for the Global Burden of Disease Study 2016. The Lancet. 2017: 390; 1211–59.<br>3. GBD 2016 Risk Factors Collaborators. Global, regional, and national comparative risk assessment of 84 behavioural, environmental and occupational, and metabolic risks or clusters of risks, 1990–2016: a systematic analysis for the Global Burden of Disease Study 2016. The Lancet. 2017: 390;1345-1422.
4	Specify the inclusion and exclusion criteria. Identify all ad-hoc exclusions.	Narrative about inclusion and exclusion criteria by data type provided in previously published appendices.	1) GBD 2016 Causes of Death Collaborators. Global, regional, and national age-sex specific mortality for 264 causes of death, 1980–2016: a systematic analysis for the Global Burden of Disease Study 2016. The Lancet. 2017: 390;1151–210.<br>2) GBD 2016 Disease and Injury Incidence and

© 2018 Global Burden of Cardiovascular Diseases Collaboration. *JAMA Cardiology*.

			Prevalence Collaborators. Global, regional, and national incidence, prevalence, and years lived with disability for 328 diseases and injuries for 195 countries, 1990–2016: a systematic analysis for the Global Burden of Disease Study 2016. The Lancet. 2017: 390; 1211–59.<br>3) GBD 2016 Risk Factors Collaborators. Global, regional, and national comparative risk assessment of 84 behavioural, environmental and occupational, and metabolic risks or clusters of risks, 1990–2016: a systematic analysis for the Global Burden of Disease Study 2016. The Lancet. 2017: 390;1345-1422.
5	Provide information on all included data sources and their main characteristics. For each data source used, report reference information or contact name/institution, population represented, data collection method, year(s) of data collection, sex and age range, diagnostic criteria or measurement method, and sample size, as relevant.	Interactive, online data source tool that provides metadata for data sources by component, geography, cause, risk, or impairment has been developed.	Online data tools: http://ghdx.healthdata.org/gbd-2016/data-input-sources
6	Identify and describe any categories of input data that have potentially important biases (e.g., based on characteristics listed in item 5).	Summary of known biases by cause included in methodological approaches sections of previously published appendices.	1) GBD 2016 Causes of Death Collaborators. Global, regional, and national age-sex specific mortality for 264 causes of death, 1980–2016: a systematic analysis for the Global Burden of Disease Study 2016. The Lancet. 2017: 390;1151–210.<br>2) GBD 2016 Disease and Injury Incidence and Prevalence Collaborators. Global, regional, and national incidence, prevalence, and years lived with disability for 328 diseases and injuries for 195 countries, 1990–2016: a systematic analysis for the Global Burden of Disease Study 2016. The Lancet. 2017: 390; 1211–59.<br>3) GBD 2016 Risk Factors Collaborators. Global, regional, and national comparative risk assessment of 84 behavioural, environmental and occupational, and metabolic risks or clusters of risks, 1990–2016: a systematic analysis for the Global Burden of Disease Study 2016. The Lancet.

© 2018 Global Burden of Cardiovascular Diseases Collaboration. *JAMA Cardiology*.

			2017: 390;1345-1422.
For data inputs that contribute to the analysis but were not synthesized as part of the study:			
7	Describe and give sources for any other data inputs.	Included in list of all data sources provided on online data source tool.	Online data tools: http://ghdx.healthdata.org/gbd-2016/data-input-sources
For all data inputs:			
8	Provide all data inputs in a file format from which data can be efficiently extracted (e.g., a spreadsheet as opposed to a PDF), including all relevant meta-data listed in item 5. For any data inputs that cannot be shared due to ethical or legal reasons, such as third-party ownership, provide a contact name or the name of the institution that retains the right to the data.	Downloads of input data will be available through online tools, including data visualization tools and data query tools. Input data not available in tools will be made available upon request.	Online data tools http://www.healthdata.org/results/data-visualizations; http://ghdx.healthdata.org/; http://ghdx.healthdata.org/gbd-data-tool
**Data analysis**			
9	Provide a conceptual overview of the data analysis method. A diagram may be helpful.	Flow diagrams of the overall methodological processes, as well as cause-specific modelling processes have been provided in previously published appendicies.	1) GBD 2016 Causes of Death Collaborators. Global, regional, and national age-sex specific mortality for 264 causes of death, 1980–2016: a systematic analysis for the Global Burden of Disease Study 2016. The Lancet. 2017: 390;1151–210.<br>2) GBD 2016 Disease and Injury Incidence and Prevalence Collaborators. Global, regional, and national incidence, prevalence, and years lived with disability for 328 diseases and injuries for 195 countries, 1990–2016: a systematic analysis for the Global Burden of Disease Study 2016. The Lancet. 2017: 390; 1211–59.<br>3) GBD 2016 Risk Factors Collaborators. Global, regional, and national comparative risk assessment of 84 behavioural, environmental and occupational, and metabolic risks or clusters of risks, 1990–2016: a systematic analysis for the Global Burden of Disease Study 2016. The Lancet. 2017: 390;1345-1422.
10	Provide a detailed description of all steps of the analysis, including mathematical formulae. This description should cover, as relevant, data cleaning, data pre-processing, data adjustments and weighting of data	Detailed descriptions of all steps of the analysis were included in the methodological approaches sections of previously published	1) GBD 2016 Causes of Death Collaborators. Global, regional, and national age-sex specific mortality for 264 causes of death,

© 2018 Global Burden of Cardiovascular Diseases Collaboration. *JAMA Cardiology*.

	sources, and mathematical or statistical model(s).	appendices.	1980–2016: a systematic analysis for the Global Burden of Disease Study 2016. The Lancet. 2017: 390;1151–210.<br>2) GBD 2016 Disease and Injury Incidence and Prevalence Collaborators. Global, regional, and national incidence, prevalence, and years lived with disability for 328 diseases and injuries for 195 countries, 1990–2016: a systematic analysis for the Global Burden of Disease Study 2016. The Lancet. 2017: 390; 1211–59.<br>3) GBD 2016 Risk Factors Collaborators. Global, regional, and national comparative risk assessment of 84 behavioural, environmental and occupational, and metabolic risks or clusters of risks, 1990–2016: a systematic analysis for the Global Burden of Disease Study 2016. The Lancet. 2017: 390;1345-1422.
11	Describe how candidate models were evaluated and how the final model(s) were selected.	Provided in the methodological write-ups of previously published appendices.	1) GBD 2016 Causes of Death Collaborators. Global, regional, and national age-sex specific mortality for 264 causes of death, 1980–2016: a systematic analysis for the Global Burden of Disease Study 2016. The Lancet. 2017: 390;1151–210.<br>2) GBD 2016 Disease and Injury Incidence and Prevalence Collaborators. Global, regional, and national incidence, prevalence, and years lived with disability for 328 diseases and injuries for 195 countries, 1990–2016: a systematic analysis for the Global Burden of Disease Study 2016. The Lancet. 2017: 390; 1211–59.<br>3) GBD 2016 Risk Factors Collaborators. Global, regional, and national comparative risk assessment of 84 behavioural, environmental and occupational, and metabolic risks or clusters of risks, 1990–2016: a systematic analysis for the Global Burden of Disease Study 2016. The Lancet. 2017: 390;1345-1422.

© 2018 Global Burden of Cardiovascular Diseases Collaboration. *JAMA Cardiology*.

12	Provide the results of an evaluation of model performance, if done, as well as the results of any relevant sensitivity analysis.	Provided in the methodological write-ups of previously published appendices.	1) GBD 2016 Causes of Death Collaborators. Global, regional, and national age-sex specific mortality for 264 causes of death, 1980–2016: a systematic analysis for the Global Burden of Disease Study 2016. The Lancet. 2017: 390;1151–210.<br>2) GBD 2016 Disease and Injury Incidence and Prevalence Collaborators. Global, regional, and national incidence, prevalence, and years lived with disability for 328 diseases and injuries for 195 countries, 1990–2016: a systematic analysis for the Global Burden of Disease Study 2016. The Lancet. 2017: 390; 1211–59.<br>3) GBD 2016 Risk Factors Collaborators. Global, regional, and national comparative risk assessment of 84 behavioural, environmental and occupational, and metabolic risks or clusters of risks, 1990–2016: a systematic analysis for the Global Burden of Disease Study 2016. The Lancet. 2017: 390;1345-1422.
13	Describe methods for calculating uncertainty of the estimates. State which sources of uncertainty were, and were not, accounted for in the uncertainty analysis.	Provided in the methodological write-ups of previously published appendices.	1) GBD 2016 Causes of Death Collaborators. Global, regional, and national age-sex specific mortality for 264 causes of death, 1980–2016: a systematic analysis for the Global Burden of Disease Study 2016. The Lancet. 2017: 390;1151–210.<br>2) GBD 2016 Disease and Injury Incidence and Prevalence Collaborators. Global, regional, and national incidence, prevalence, and years lived with disability for 328 diseases and injuries for 195 countries, 1990–2016: a systematic analysis for the Global Burden of Disease Study 2016. The Lancet. 2017: 390; 1211–59.<br>3) GBD 2016 Risk Factors Collaborators. Global, regional, and national comparative risk assessment of 84 behavioural, environmental and occupational, and

© 2018 Global Burden of Cardiovascular Diseases Collaboration. *JAMA Cardiology*.

			metabolic risks or clusters of risks, 1990–2016: a systematic analysis for the Global Burden of Disease Study 2016. The Lancet. 2017: 390;1345-1422.
14	State how analytic or statistical source code used to generate estimates can be accessed.	Access statement provided.	http://ghdx.healthdata.org/global-burden-disease-study-2016
**Results and Discussion**			
15	Provide published estimates in a file format from which data can be efficiently extracted.	GBD 2016 results are available through online data visualization tools, the Global Health Data Exchange, and the online data query tool (these tools are already available for GBD 2013 results).	Online data tools http://www.healthdata.org/results/data-visualizations; http://ghdx.healthdata.org/; http://ghdx.healthdata.org/gbd-data-tool
16	Report a quantitative measure of the uncertainty of the estimates (e.g. uncertainty intervals).	Uncertainty intervals are provided with all results.	Main text; Online data tools http://www.healthdata.org/results/data-visualizations; http://ghdx.healthdata.org/; http://ghdx.healthdata.org/gbd-data-tool
17	Interpret results in light of existing evidence. If updating a previous set of estimates, describe the reasons for changes in estimates.	Discussion of methodological changes between GBD rounds provided in the appendices of previously published papers.	1) GBD 2016 Causes of Death Collaborators. Global, regional, and national age-sex specific mortality for 264 causes of death, 1980–2016: a systematic analysis for the Global Burden of Disease Study 2016. The Lancet. 2017: 390;1151–210.<br>2) GBD 2016 Disease and Injury Incidence and Prevalence Collaborators. Global, regional, and national incidence, prevalence, and years lived with disability for 328 diseases and injuries for 195 countries, 1990–2016: a systematic analysis for the Global Burden of Disease Study 2016. The Lancet. 2017: 390; 1211–59.<br>3) GBD 2016 Risk Factors Collaborators. Global, regional, and national comparative risk assessment of 84 behavioural, environmental and occupational, and metabolic risks or clusters of risks, 1990–2016: a systematic analysis for the Global Burden of Disease Study 2016. The Lancet. 2017: 390;1345-1422.
18	Discuss limitations of the estimates. Include a discussion of any modelling assumptions or data limitations that affect interpretation of the estimates.	Discussion of limitations provided in the narrative of the main paper as well as in the methodological write-ups of previously published appendices	1) Main text, Limitations<br>2) GBD 2016 Causes of Death Collaborators. Global, regional, and national age-sex specific mortality for 264 causes of death, 1980–2016: a systematic analysis for the

© 2018 Global Burden of Cardiovascular Diseases Collaboration. *JAMA Cardiology*.

			Global Burden of Disease Study 2016. The Lancet. 2017: 390;1151–210.<br>3) GBD 2016 Disease and Injury Incidence and Prevalence Collaborators. Global, regional, and national incidence, prevalence, and years lived with disability for 328 diseases and injuries for 195 countries, 1990–2016: a systematic analysis for the Global Burden of Disease Study 2016. The Lancet. 2017: 390; 1211–59.<br>4) GBD 2016 Risk Factors Collaborators. Global, regional, and national comparative risk assessment of 84 behavioural, environmental and occupational, and metabolic risks or clusters of risks, 1990–2016: a systematic analysis for the Global Burden of Disease Study 2016. The Lancet. 2017: 390;1345-1422.

© 2018 Global Burden of Cardiovascular Diseases Collaboration. *JAMA Cardiology*.

Appendix Table 2. GBD 2016 Cause and Sequela Hierarchy for Cardiovascular Diseases

Causes and sequelae	Level
Cardiovascular diseases	2
Rheumatic heart disease	3
Asymptomatic and mild heart failure due to rheumatic heart disease	5
Moderate heart failure due to rheumatic heart disease	5
Rheumatic heart disease, without heart failure	5
Severe heart failure due to rheumatic heart disease	5
Ischemic heart disease	3
Acute myocardial infarction 3 to 28 days	5
Acute myocardial infarction first 2 days	5
Asymptomatic and mild heart failure due to ischemic heart disease	5
Asymptomatic angina due to ischemic heart disease	5
Asymptomatic ischemic heart disease following myocardial infarction	5
Mild angina due to ischemic heart disease	5
Moderate angina due to ischemic heart disease	5
Moderate heart failure due to ischemic heart disease	5
Severe angina due to ischemic heart disease	5
Severe heart failure due to ischemic heart disease	5
Cerebrovascular disease	3
Ischemic stroke	4
Acute ischemic stroke severity level 1	5
Acute ischemic stroke severity level 2	5
Acute ischemic stroke severity level 3	5
Acute ischemic stroke severity level 4	5
Acute ischemic stroke severity level 5	5
Asymptomatic chronic ischemic stroke	5
Chronic ischemic stroke severity level 1	5
Chronic ischemic stroke severity level 2	5
Chronic ischemic stroke severity level 3	5

© 2018 Global Burden of Cardiovascular Diseases Collaboration. *JAMA Cardiology*.

Chronic ischemic stroke severity level 4	5
Chronic ischemic stroke severity level 5	5
Hemorrhagic stroke	4
Acute hemorrhagic stroke severity level 1	5
Acute hemorrhagic stroke severity level 2	5
Acute hemorrhagic stroke severity level 3	5
Acute hemorrhagic stroke severity level 4	5
Acute hemorrhagic stroke severity level 5	5
Asymptomatic chronic hemorrhagic stroke	5
Chronic hemorrhagic stroke severity level 1	5
Chronic hemorrhagic stroke severity level 2	5
Chronic hemorrhagic stroke severity level 3	5
Chronic hemorrhagic stroke severity level 4	5
Chronic hemorrhagic stroke severity level 5	5
Hypertensive heart disease	3
Asymptomatic and mild heart failure due to hypertensive heart disease	5
Moderate heart failure due to hypertensive heart disease	5
Severe heart failure due to hypertensive heart disease	5
Cardiomyopathy and myocarditis	3
Myocarditis	4
Acute myocarditis	5
Asymptomatic and mild heart failure due to myocarditis	5
Moderate heart failure due to myocarditis	5
Severe heart failure due to myocarditis	5
Alcoholic cardiomyopathy	4
Asymptomatic and mild heart failure due to alcoholic cardiomyopathy	5
Moderate heart failure due to alcoholic cardiomyopathy	5
Severe heart failure due to alcoholic cardiomyopathy	5
Other cardiomyopathy	4
Asymptomatic and mild heart failure due to other cardiomyopathy	5

© 2018 Global Burden of Cardiovascular Diseases Collaboration. *JAMA Cardiology*.

Moderate heart failure due to other cardiomyopathy	5
Severe heart failure due to other cardiomyopathy	5
Atrial fibrillation and flutter	3
Asymptomatic atrial fibrillation and flutter	5
Symptomatic atrial fibrillation and flutter	5
Aortic aneurysm	3
Peripheral artery disease	3
Asymptomatic peripheral vascular disease	5
Symptomatic claudication due to peripheral vascular disease	5
Endocarditis	3
Asymptomatic and mild heart failure due to endocarditis	5
Moderate endocarditis	5
Moderate heart failure due to endocarditis	5
Severe endocarditis	5
Severe heart failure due to endocarditis	5
Other cardiovascular and circulatory diseases	3
Asymptomatic and mild heart failure due to other cardiovascular diseases	5
Asymptomatic other cardiovascular diseases	5
Mild other cardiovascular diseases	5
Moderate heart failure due to other cardiovascular diseases	5
Moderate other cardiovascular diseases	5
Severe heart failure due to other cardiovascular diseases	5
Severe other cardiovascular diseases	5

© 2018 Global Burden of Cardiovascular Diseases Collaboration. *JAMA Cardiology*.

Appendix Table 3a. GBD 2016 CODem model covariates by CVD Cause				
**Cause Name**	**Covariate**	**Transformation**	**Level**	**Direction**
Cardiovascular diseases	Summary exposure variable	None	1	1
Cardiovascular diseases	Cholesterol (total, mean per capita)	None	1	1
Cardiovascular diseases	Smoking prevalence	None	1	1
Cardiovascular diseases	Systolic blood pressure (mmHg)	None	1	1
Cardiovascular diseases	Trans fatty acid	None	1	1
Cardiovascular diseases	Mean BMI	None	2	1
Cardiovascular diseases	Elevation over 1500m (proportion)	None	2	-1
Cardiovascular diseases	Fasting plasma glucose (mmol/L)	None	2	1
Cardiovascular diseases	Outdoor pollution ($PM_{2.5}$)	None	2	1
Cardiovascular diseases	Indoor air pollution (all fuel types)	None	2	1
Cardiovascular diseases	Healthcare access and quality index	None	2	-1
Cardiovascular diseases	Lag distributed income per capita (I$)	Log	3	-1
Cardiovascular diseases	Socio-demographic Index	None	3	0
Cardiovascular diseases	Omega-3 (kcal/capita, adjusted)	Log	3	-1
Cardiovascular diseases	Fruits (kcal/capita, adjusted)	None	3	-1
Cardiovascular diseases	Vegetables (kcal/capita, adjusted)	None	3	-1
Cardiovascular diseases	Nuts and seeds (kcal/capita, adjusted)	None	3	-1
Cardiovascular diseases	Whole grains (kcal/capita, adjusted)	None	3	-1
Cardiovascular diseases	Pulses/legumes (kcal/capita, adjusted)	None	3	-1
Cardiovascular diseases	PUFA adjusted (percent)	None	3	-1
Cardiovascular diseases	Alcohol (litres per capita)	None	3	0
Rheumatic heart disease	SEV	None	1	1
Rheumatic heart disease	Improved water (proportion)	None	1	-1
Rheumatic heart disease	Malnutrition	None	1	1
Rheumatic heart disease	Sanitation (proportion with access)	None	1	-1
Rheumatic heart disease	Healthcare access and quality index	None	2	-1
Rheumatic heart disease	LDI	Log	3	-1
Rheumatic heart disease	SDI	None	3	-1
Rheumatic heart disease	Education (years per capita)	None	3	-1
Ischemic heart disease	Summary exposure variable	None	1	1
Ischemic heart disease	Cholesterol (total, mean per capita)	None	1	1
Ischemic heart disease	Smoking prevalence	None	1	1
Ischemic heart disease	Systolic blood pressure (mmHg)	None	1	1
Ischemic heart disease	Trans fatty acid	None	1	1
Ischemic heart disease	Mean BMI	None	2	1
Ischemic heart disease	Elevation over 1500m (proportion)	None	2	-1
Ischemic heart disease	Fasting plasma glucose	None	2	1
Ischemic heart disease	Outdoor pollution ($PM_{2.5}$)	None	2	1
Ischemic heart disease	Indoor air pollution	None	2	1
Ischemic heart disease	Healthcare access and quality index	None	2	-1

© 2018 Global Burden of Cardiovascular Diseases Collaboration. *JAMA Cardiology*.

Ischemic heart disease	Lag distributed income per capita (I$)	Log	3	-1
Ischemic heart disease	Socio-demographic Index	None	3	0
Ischemic heart disease	Omega-3 (kcal/capita, adjusted)	Log	3	-1
Ischemic heart disease	Fruits (kcal/capita, adjusted)	None	3	-1
Ischemic heart disease	Vegetables (kcal/capita, adjusted)	None	3	-1
Ischemic heart disease	Nuts and seeds (kcal/capita, adjusted)	None	3	-1
Ischemic heart disease	Whole grains (kcal/capita, adjusted)	None	3	-1
Ischemic heart disease	Pulses/legumes (kcal/capita, adjusted)	None	3	-1
Ischemic heart disease	PUFA adjusted (percent)	None	3	-1
Ischemic heart disease	Alcohol (litres per capita)	None	3	0
Cerebrovascular disease	Summary exposure variable	None	1	1
Cerebrovascular disease	Cholesterol (total, mean per capita)	None	1	1
Cerebrovascular disease	Smoking prevalence	None	1	1
Cerebrovascular disease	Systolic blood pressure (mmHg)	None	1	1
Cerebrovascular disease	Trans fatty acid	None	1	1
Cerebrovascular disease	Mean BMI	None	2	1
Cerebrovascular disease	Elevation over 1500m (proportion)	None	2	-1
Cerebrovascular disease	Fasting plasma glucose	None	2	1
Cerebrovascular disease	Outdoor pollution ($PM_{2.5}$)	None	2	1
Cerebrovascular disease	Indoor air pollution	None	2	1
Cerebrovascular disease	Healthcare access and quality index	None	2	-1
Cerebrovascular disease	Lag distributed income per capita (I$)	Log	3	-1
Cerebrovascular disease	Socio-demographic Index	None	3	0
Cerebrovascular disease	Omega-3 (kcal/capita, adjusted)	Log	3	-1
Cerebrovascular disease	Fruits (kcal/capita, adjusted)	None	3	-1
Cerebrovascular disease	Vegetables (kcal/capita, adjusted)	None	3	-1
Cerebrovascular disease	Nuts and seeds (kcal/capita, adjusted)	None	3	-1
Cerebrovascular disease	Whole grains (kcal/capita, adjusted)	None	3	-1
Cerebrovascular disease	Pulses/legumes (kcal/capita, adjusted)	None	3	-1
Cerebrovascular disease	PUFA adjusted (percent)	None	3	-1
Cerebrovascular disease	Alcohol (litres per capita)	None	3	0
Ischemic stroke	Summary exposure variable	None	1	1
Ischemic stroke	Cholesterol (total, mean per capita)	None	1	1
Ischemic stroke	Smoking prevalence	None	1	1
Ischemic stroke	Systolic blood pressure (mmHg)	None	1	1
Ischemic stroke	Trans fatty acid	None	1	1
Ischemic stroke	Mean BMI	None	2	1
Ischemic stroke	Elevation over 1500m (proportion)	None	2	-1
Ischemic stroke	Fasting plasma glucose	None	2	1
Ischemic stroke	Outdoor pollution ($PM_{2.5}$)	None	2	1
Ischemic stroke	Indoor air pollution	None	2	1
Ischemic stroke	Healthcare access and quality index	None	2	-1

© 2018 Global Burden of Cardiovascular Diseases Collaboration. *JAMA Cardiology*.

Ischemic stroke	Lag distributed income per capita (I$)	Log	3	-1
Ischemic stroke	Socio-demographic Index	None	3	0
Ischemic stroke	Omega-3 (kcal/capita, adjusted)	Log	3	-1
Ischemic stroke	Fruits (kcal/capita, adjusted)	None	3	-1
Ischemic stroke	Vegetables (kcal/capita, adjusted)	None	3	-1
Ischemic stroke	Nuts and seeds (kcal/capita, adjusted)	None	3	-1
Ischemic stroke	Whole grains (kcal/capita, adjusted)	None	3	-1
Ischemic stroke	Pulses/legumes (kcal/capita, adjusted)	None	3	-1
Ischemic stroke	PUFA adjusted (percent)	None	3	-1
Ischemic stroke	Alcohol (litres per capita)	None	3	0
Hemorrhagic stroke	Summary exposure variable	None	1	1
Hemorrhagic stroke	Cholesterol (total, mean per capita)	None	1	0
Hemorrhagic stroke	Smoking prevalence	None	1	1
Hemorrhagic stroke	Systolic blood pressure (mmHg)	None	1	1
Hemorrhagic stroke	Trans fatty acid	None	1	1
Hemorrhagic stroke	Mean BMI	None	2	1
Hemorrhagic stroke	Elevation over 1500m (proportion)	None	2	-1
Hemorrhagic stroke	Fasting plasma glucose	None	2	1
Hemorrhagic stroke	Outdoor pollution ($PM_{2.5}$)	None	2	1
Hemorrhagic stroke	Indoor air pollution	None	2	1
Hemorrhagic stroke	Healthcare access and quality index	None	2	-1
Hemorrhagic stroke	Lag distributed income per capita (I$)	Log	3	-1
Hemorrhagic stroke	Socio-demographic index	None	3	0
Hemorrhagic stroke	Omega-3 (kcal/capita, adjusted)	Log	3	-1
Hemorrhagic stroke	Fruits (kcal/capita, adjusted)	None	3	-1
Hemorrhagic stroke	Vegetables (kcal/capita, adjusted)	None	3	-1
Hemorrhagic stroke	Nuts and seeds (kcal/capita, adjusted)	None	3	-1
Hemorrhagic stroke	Whole grains (kcal/capita, adjusted)	None	3	-1
Hemorrhagic stroke	Pulses/legumes (kcal/capita, adjusted)	None	3	-1
Hemorrhagic stroke	PUFA adjusted (percent)	None	3	-1
Hemorrhagic stroke	Alcohol (litres per capita)	None	3	0
Hypertensive heart disease	Cholesterol (total, mean per capita)	None	1	1
Hypertensive heart disease	Smoking prevalence	None	1	1
Hypertensive heart disease	Systolic blood pressure (mmHg)	None	1	1
Hypertensive heart disease	Mean BMI	None	2	1
Hypertensive heart disease	Healthcare access and quality index	None	2	-1
Hypertensive heart disease	Lag distributed income per capita (I$)	Log	3	-1
Hypertensive heart disease	Socio-demographic index	None	3	0
Alcoholic Cardiomyopathy	Summary exposure variable, CMP	none	1	1
Alcoholic Cardiomyopathy	Smoking prevalence	none	1	1
Alcoholic Cardiomyopathy	Alcohol (litres per capita)	none	1	1
Alcoholic Cardiomyopathy	Healthcare access and quality index	none	2	-1
Alcoholic Cardiomyopathy	Lag distributed income per capita (I$)	log	3	0

© 2018 Global Burden of Cardiovascular Diseases Collaboration. *JAMA Cardiology*.

Alcoholic Cardiomyopathy	Socio-demographic Index	none	3	0
Myocarditis	Summary exposure variable, CMP	none	1	1
Myocarditis	Systolic blood pressure (mm Hg)	none	1	1
Myocarditis	Healthcare access and quality index	none	2	-1
Myocarditis	Lag distributed income per capita (I$)	log	3	0
Myocarditis	Socio-demographic Index	none	3	0
Other Cardiomyopathy	Summary exposure variable, CMP	none	1	1
Other Cardiomyopathy	Systolic blood pressure (mmHg)	none	1	1
Other Cardiomyopathy	Smoking prevalence	none	1	1
Other Cardiomyopathy	Body mass index (kg/m^2)	none	2	1
Other Cardiomyopathy	Healthcare access and quality index	none	2	-1
Other Cardiomyopathy	Lag distributed income per capita (I$)	log	3	0
Other Cardiomyopathy	Socio-demographic Index	none	3	0
Atrial fibrillation and flutter	Summary exposure variable	None	1	1
Atrial fibrillation and flutter	Cholesterol (total, mean per capita)	None	1	1
Atrial fibrillation and flutter	Smoking prevalence	None	1	1
Atrial fibrillation and flutter	Systolic blood pressure (mmHg)	None	1	1
Atrial fibrillation and flutter	Mean BMI	None	2	1
Atrial fibrillation and flutter	Elevation over 1500m (proportion)	None	2	-1
Atrial fibrillation and flutter	Fasting plasma glucose	None	2	1
Atrial fibrillation and flutter	Outdoor pollution ($PM_{2.5}$)	None	2	1
Atrial fibrillation and flutter	Indoor air pollution	None	2	1
Atrial fibrillation and flutter	Healthcare Access and Quality Index	None	2	-1
Atrial fibrillation and flutter	Lag distributed income per capita (I$)	Log	3	-1
Atrial fibrillation and flutter	Socio-demographic Index	None	3	0
Atrial fibrillation and flutter	Omega-3 (kcal/capita, adjusted)	Log	3	-1
Atrial fibrillation and flutter	Fruits (kcal/capita, adjusted)	None	3	-1
Atrial fibrillation and flutter	Vegetables (kcal/capita, adjusted)	None	3	-1
Atrial fibrillation and flutter	Nuts and seeds (kcal/capita, adjusted)	None	3	-1
Atrial fibrillation and flutter	Whole grains (kcal/capita, adjusted)	None	3	-1
Atrial fibrillation and flutter	Pulses/legumes (kcal/capita, adjusted)	None	3	-1
Atrial fibrillation and flutter	PUFA adjusted (percent)	None	3	-1
Atrial fibrillation and flutter	Alcohol (litres per capita)	None	3	0
Atrial fibrillation and flutter	Trans fatty acid	None	1	1
Aortic aneurysm	Summary exposure variable	None	1	1
Aortic aneurysm	Cholesterol (total, mean per capita)	None	1	1
Aortic aneurysm	Cumulative cigarettes (10 yrs)	None	1	1
Aortic aneurysm	Systolic blood pressure (mmHg)	None	1	1
Aortic aneurysm	Trans fatty acid (percent)	None	1	1
Aortic aneurysm	Mean BMI	None	2	1
Aortic aneurysm	Healthcare access and quality index	None	2	-1
Aortic aneurysm	Lag distributed income per capita (I$)	Log	3	-1

© 2018 Global Burden of Cardiovascular Diseases Collaboration. *JAMA Cardiology*.

Aortic aneurysm	Socio-demographic Index	None	3	0
Aortic aneurysm	Omega-3 (kcal/capita, adjusted)	Log	3	-1
Aortic aneurysm	Fruits (kcal/capita, adjusted)	None	3	-1
Aortic aneurysm	Vegetables (kcal/capita, adjusted)	None	3	-1
Aortic aneurysm	Nuts and seeds (kcal/capita, adjusted)	None	3	-1
Aortic aneurysm	Whole grains (kcal/capita, adjusted)	None	3	-1
Aortic aneurysm	Pulses/legumes (kcal/capita, adjusted)	None	3	-1
Aortic aneurysm	PUFA adjusted (percent)	None	3	-1
Aortic aneurysm	Alcohol (litres per capita)	None	3	0
Peripheral artery disease	Summary exposure variable	None	1	1
Peripheral artery disease	Systolic blood pressure (mmHg)	None	1	1
Peripheral artery disease	Cholesterol (total, mean per capita)	None	1	1
Peripheral artery disease	Smoking prevalence	None	1	1
Peripheral artery disease	Mean body mass index (kg/m^2)	None	2	1
Peripheral artery disease	Healthcare access and quality index	None	2	-1
Peripheral artery disease	Lag distributed income per capita (I$)	Log	3	-1
Peripheral artery disease	Socio-demographic Index	None	3	0
Peripheral artery disease	Omega-3 (kcal/capita, adjusted)	Log	3	-1
Peripheral artery disease	Fruits (kcal/capita, adjusted)	None	3	-1
Peripheral artery disease	Vegetables (kcal/capita, adjusted)	None	3	-1
Peripheral artery disease	Nuts and seeds (kcal/capita, adjusted)	None	3	-1
Peripheral artery disease	Whole grains (kcal/capita, adjusted)	None	3	-1
Peripheral artery disease	Pulses/legumes (kcal/capita, adjusted)	None	3	-1
Peripheral artery disease	PUFA adjusted (percent)	None	3	-1
Peripheral artery disease	Trans fatty acid (percent)	None	3	1
Peripheral artery disease	Alcohol (litres per capita)	None	3	0
Endocarditis	Summary exposure variable	None	1	1
Endocarditis	Improved water (proportion)	None	1	-1
Endocarditis	Sanitation (proportion with access)	None	1	-1
Endocarditis	Healthcare access and quality index	None	1	-1
Endocarditis	Lag distributed income per capita (I$)	Log	3	-1
Endocarditis	Socio-demographic Index	None	3	0
Other cardiovascular and circulatory diseases	Summary exposure variable	None	1	1
Other cardiovascular and circulatory diseases	Cholesterol (total, mean per capita)	None	1	1
Other cardiovascular and circulatory diseases	Smoking prevalence	None	1	1
Other cardiovascular and circulatory diseases	Systolic blood pressure (mmHg)	None	1	1
Other cardiovascular and circulatory diseases	Trans fatty acid (percent)	None	1	1
Other cardiovascular and circulatory diseases	Mean BMI	None	2	1
Other cardiovascular and circulatory diseases	Elevation over 1500m (proportion)	None	2	-1
Other cardiovascular and circulatory diseases	Fasting plasma glucose (mmol/L)	None	2	1
Other cardiovascular and circulatory diseases	Indoor air pollution (all fuel types)	None	2	1
Other cardiovascular and circulatory diseases	Outdoor air pollution ($PM_{2.5}$)	None	2	1

© 2018 Global Burden of Cardiovascular Diseases Collaboration. *JAMA Cardiology*.

Other cardiovascular and circulatory diseases	Healthcare access and quality index	None	2	-1
Other cardiovascular and circulatory diseases	Lag distributed income per capita (I$)	Log	3	-1
Other cardiovascular and circulatory diseases	Socio-demographic Index	None	3	0
Other cardiovascular and circulatory diseases	Omega-3 (kcal/capita, adjusted)	Log	3	-1
Other cardiovascular and circulatory diseases	Fruits (kcal/capita, adjusted)	None	3	-1
Other cardiovascular and circulatory diseases	Vegetables (kcal/capita, adjusted)	None	3	-1
Other cardiovascular and circulatory diseases	Nuts and seeds (kcal/capita, adjusted)	None	3	-1
Other cardiovascular and circulatory diseases	Whole grains (kcal/capita, adjusted)	None	3	-1
Other cardiovascular and circulatory diseases	Pulses/legumes (kcal/capita, adjusted)	None	3	-1
Other cardiovascular and circulatory diseases	PUFA adjusted (percent)	None	3	-1
Other cardiovascular and circulatory diseases	Alcohol (litres per capita)	None	3	0

© 2018 Global Burden of Cardiovascular Diseases Collaboration. *JAMA Cardiology*.

Appendix Table 3b. GBD 2016 Dismod model covariates by CVD Cause		
**Cause**	**Covariate Name**	**Measure**
Acute Myocardial Infarction	Diagnostic blood sample (troponin)	Incidence
Acute Myocardial Infarction	First ever MI	Incidence
Acute Myocardial Infarction	LDI (I$ per capita)	Excess mortality rate
Acute Myocardial Infarction	Log-transformed age-standardized SEV scalar: IHD	Incidence
Acute Myocardial Infarction	Non-fatal MI	Incidence
Acute Myocarditis	All MarketScan, year 2000	Incidence
Acute Myocarditis	All MarketScan, year 2010	Incidence
Acute Myocarditis	LDI (I$ per capita)	Excess mortality rate
Acute Myocarditis	Log-transformed age-standardized SEV scalar: CMP	Incidence
Angina	LDI (I$ per capita)	Excess mortality rate
Angina	Log-transformed age-standardized SEV scalar: IHD	Prevalence
Angina	RAQ, female, 50 to 64	Prevalence
Angina	RAQ, female, 65 plus	Prevalence
Angina	RAQ, female, less than 50	Prevalence
Angina	RAQ, male, 50 to 64	Prevalence
Angina	RAQ, male, 65 plus	Prevalence
Angina	RAQ, male, less than 50	Prevalence
Atrial Fibrillation	All MarketScan, year 2000	Prevalence
Atrial Fibrillation	All MarketScan, year 2010	Prevalence
Atrial Fibrillation	Hospital data	Prevalence
Atrial Fibrillation	LDI (I$ per capita)	Excess mortality rate
Atrial Fibrillation	Log-transformed age-standardized SEV scalar: A Fib	Prevalence
Chronic hemorrhagic stroke	LDI (I$ per capita)	Excess mortality rate
Chronic hemorrhagic stroke	Log-transformed SEV scalar: Hem Stroke	Prevalence
Chronic ischemic stroke	LDI (I$ per capita)	Excess mortality rate
Chronic ischemic stroke	Log-transformed SEV scalar: Isch Stroke	Prevalence
Endocarditis	Hospital data	Incidence
Endocarditis	Inpatient-only Marketscan, year 2000	Incidence
Endocarditis	Inpatient-only Marketscan, year 2010	Incidence
Endocarditis	LDI (I$per capita)	Excess mortality rate
Endocarditis	Log-transformed age-standardized SEV scalar: endocarditis	Incidence
First ever acute hemorrhagic stroke	Any stroke	Incidence
First ever acute hemorrhagic stroke	Any stroke	Excess mortality rate
First ever acute hemorrhagic stroke	First-ever acute stroke, ischemic or hemorrhagic	Incidence
First ever acute hemorrhagic stroke	First-ever acute stroke, ischemic or hemorrhagic	Excess mortality rate
First ever acute hemorrhagic stroke	Hospital data	Incidence

© 2018 Global Burden of Cardiovascular Diseases Collaboration. *JAMA Cardiology*.

First ever acute hemorrhagic stroke	Log-transformed age-standardized SEV scalar: hemorrhagic stroke	Incidence
First ever acute ischemic stroke	Any stroke	Incidence
First ever acute ischemic stroke	First-ever acute stroke, ischemic or hemorrhagic	Incidence
First ever acute ischemic stroke	Hospital data	Incidence
First ever acute ischemic stroke	Log-transformed age-standardized SEV scalar: ischemic stroke	Incidence
HF Envelope	All MarketScan, year 2000	Prevalence
HF Envelope	LDI (I$ per capita)	Excess mortality rate
HF Envelope	Log-transformed age-standardised SEV scalar: CVD	Prevalence
Peripheral Arterial Disease	Healthcare access and quality index	Proportion
Peripheral Arterial Disease	LDI (I$ per capita)	Excess mortality rate
Peripheral Arterial Disease	Log-transformed age-standardized SEV scalar: PVD	Prevalence
Rhuematic Heart Disease - Endemic	LDI (I$ per capita)	Excess mortality rate
Rhuematic Heart Disease - Endemic	Log-transformed age-standardized SEV scalar: RHD	Prevalence
Rhuematic Heart Disease - Nonendemic	All Marketscan, year 2000	Study-level
Rhuematic Heart Disease - Nonendemic	All Marketscan, year 2010	Study-level`
Rhuematic Heart Disease - Nonendemic	LDI (I$ per capita)	Excess mortality rate
Rhuematic Heart Disease - Nonendemic	Log-transformed age-standardized SEV scalar: RHD	Prevalence

© 2018 Global Burden of Cardiovascular Diseases Collaboration. *JAMA Cardiology*.

Appendix Table 4: List of International Classification of Diseases (ICD) codes mapped to the Global Burden of Disease cause list for Cardiovascular Diseases				
**Cause**	**ICD10**	**ICD10 Used in Hospital/Claims Analyses**	**ICD9**	**ICD9 Used in Hospital/Claims Analyses**
Rheumatic heart disease	I01-I01.9, I02.0, I05-I09.9	I01-I09.9	391-391.9, 392.0, 393-398.99	391-398.99
Ischemic heart disease	I20-I21.6, I21.9-I25.9, Z82.4-Z82.49	I20-I25.9	410-414.9, V17.3	410-414.9
Cerebrovascular disease	G45-G46.8, I60-I64, I64.1, I65-I69.998, Z82.3		430-439.6, V12.54, V17.1	
Ischemic stroke	G45-G46.8, I63-I63.9, I65-I66.9, I67.2-I67.848, I69.3-I69.4	I63-I63.9, I67.2-I67.848, I69.3-I69.4	433-435.9, 437.0-437.2, 437.4-437.9	434-434.91, 437.0-437.2, 437.4-437.9
Hemorrhagic stroke	I60-I62.9, I67.0-I67.1, I69.0-I69.298	I60-I62.9, I67.0-I67.1, I69.0-I69.298	430-432.9, 437.3	430-432.9
Hypertensive heart disease	I11-I11.2, I11.9		402-402.91	
Cardiomyopathy and myocarditis	B33.2-B33.20, B33.22-B33.24, D86.85, I40-I41.8, I42-I43.8, I51.4-I51.6		074.2, 074.23, 422-422.99, 425-425.5, 425.7-425.9, 429.0-429.1	
Myocarditis	B33.2-B33.20, B33.22-B33.24, D86.85, I40-I41.8, I51.4-I51.6	B33.2-B33.20, B33.22-B33.24, D86.85, I40-I41.8, I51.4-I51.6	074.2, 074.23, 422-422.99, 429.0-429.1	074.2, 074.23, 422-422.99, 429.0-429.1
Alcoholic cardiomyopathy	I42.6		425.5	
Other cardiomyopathy	I42.0-I42.5, I42.7-I43.8		425.0-425.4, 425.7-425.9	
Atrial fibrillation and flutter	I48-I48.92	I48-I48.92	427.3-427.32	427.3-427.32
Peripheral artery disease	I70.2-I70.92, I73-I73.9	I70.2-I73.9	440.2-440.29, 440.4-440.9, 443-443.9	440.2-440.29, 440.4-443.9
Endocarditis	B33.21, I33-I33.9, I38-I38.0, I39-I39.9	B33.21, I33-I39.9	074.22, 421-421.9, 424, 424.4-424.99	074.22, 421-421.9, 424-424.99
Other cardiovascular and circulatory diseases	I30-I32.8, I34-I37.9, I51-I51.3, I51.7-I52.8, I72-I72.9, I77-I83.93, I86-I89.0, I89.9, I95.0-I95.1, I98, I98.8-I99.9, K75.1		074.21, 417-417.9, 420-420.99, 423-423.9, 424.0-424.3, 429, 429.2-429.9, 442-442.9, 447-454.9, 456, 456.3-457, 457.1, 457.8-458.1, 459-459.9	

© 2018 Global Burden of Cardiovascular Diseases Collaboration. *JAMA Cardiology*.

Appendix Table 5. GBD 2016 Citations Sorted by CVD Cause	
**GBD cause name**	**Citation**
Atrial fibrillation and flutter	Healthcare Cost and Utilization Project (HCUP), Agency for Healthcare Research and Quality (AHRQ). United States State Inpatient Databases 2009. Rockville, United States: Healthcare Cost and Utilization Project (HCUP), Agency for Healthcare Research and Quality (AHRQ)
Atrial fibrillation and flutter	Healthcare Cost and Utilization Project (HCUP), Agency for Healthcare Research and Quality (AHRQ). United States State Inpatient Databases 2005. Rockville, United States: Healthcare Cost and Utilization Project (HCUP), Agency for Healthcare Research and Quality (AHRQ)
Atrial fibrillation and flutter	Healthcare Cost and Utilization Project (HCUP), Agency for Healthcare Research and Quality (AHRQ). United States State Inpatient Databases 2013. Rockville, United States: Healthcare Cost and Utilization Project (HCUP), Agency for Healthcare Research and Quality (AHRQ)
Atrial fibrillation and flutter	Healthcare Cost and Utilization Project (HCUP), Agency for Healthcare Research and Quality (AHRQ). United States State Inpatient Databases 2011. Rockville, United States: Healthcare Cost and Utilization Project (HCUP), Agency for Healthcare Research and Quality (AHRQ)
Atrial fibrillation and flutter	Truven Health Analytics. United States MarketScan Claims and Medicare Data - 2012. Ann Arbor, United States: Truven Health Analytics
Atrial fibrillation and flutter	Healthcare Cost and Utilization Project (HCUP), Agency for Healthcare Research and Quality (AHRQ). United States State Inpatient Databases 2012. Rockville, United States: Healthcare Cost and Utilization Project (HCUP), Agency for Healthcare Research and Quality (AHRQ)
Atrial fibrillation and flutter	National Center for Health Statistics (NCHS), Centers for Disease Control and Prevention (CDC), United States Census Bureau. United States National Hospital Discharge Survey 1991. Hyattsville, United States: National Center for Health Statistics (NCHS), Centers for Disease Control and Prevention (CDC)
Atrial fibrillation and flutter	Healthcare Cost and Utilization Project (HCUP), Agency for Healthcare Research and Quality (AHRQ). United States State Inpatient Databases 2014. Rockville, United States: Healthcare Cost and Utilization Project (HCUP), Agency for Healthcare Research and Quality (AHRQ)
Atrial fibrillation and flutter	National Center for Health Statistics (NCHS), Centers for Disease Control and Prevention (CDC), United States Census Bureau. United States National Hospital Discharge Survey 1987. Hyattsville, United States: National Center for Health Statistics (NCHS), Centers for Disease Control and Prevention (CDC)
Atrial fibrillation and flutter	Healthcare Cost and Utilization Project (HCUP), Agency for Healthcare Research and Quality (AHRQ). United States State Inpatient Databases 2003. Rockville, United States: Healthcare Cost and Utilization Project (HCUP), Agency for Healthcare Research and Quality (AHRQ)
Atrial fibrillation and flutter	Truven Health Analytics. United States MarketScan Claims and Medicare Data - 2010. Ann Arbor, United States: Truven Health Analytics
Atrial fibrillation and flutter	Healthcare Cost and Utilization Project (HCUP), Agency for Healthcare Research and Quality (AHRQ). United States State Inpatient Databases 2004. Rockville, United States: Healthcare Cost and Utilization Project (HCUP), Agency for Healthcare Research and Quality (AHRQ)
Atrial fibrillation and flutter	Healthcare Cost and Utilization Project (HCUP), Agency for Healthcare Research and Quality (AHRQ). United States State Inpatient Databases 2007. Rockville, United States: Healthcare Cost and Utilization Project (HCUP), Agency for Healthcare Research and Quality (AHRQ)
Atrial fibrillation and flutter	National Center for Health Statistics (NCHS), Centers for Disease Control and Prevention (CDC), United States Census Bureau. United States National Hospital Discharge Survey 2005. Hyattsville, United States: National Center for Health Statistics (NCHS), Centers for Disease Control and Prevention (CDC)
Atrial fibrillation and flutter	National Center for Health Statistics (NCHS), Centers for Disease Control and Prevention (CDC), United States Census Bureau. United States National Hospital Discharge Survey 1990. Hyattsville, United States: National Center for Health Statistics (NCHS), Centers for Disease Control and Prevention (CDC)
Atrial fibrillation and flutter	Healthcare Cost and Utilization Project (HCUP), Agency for Healthcare Research and Quality (AHRQ). United States State Inpatient Databases 2006. Rockville, United States: Healthcare Cost and Utilization Project (HCUP), Agency for Healthcare Research and Quality (AHRQ)
Atrial fibrillation and flutter	National Center for Health Statistics (NCHS), Centers for Disease Control and Prevention (CDC), United States Census Bureau. United States National Hospital Discharge Survey 1999. Hyattsville, United States: National Center for Health Statistics (NCHS), Centers for Disease Control and Prevention (CDC)
Atrial fibrillation and flutter	National Center for Health Statistics (NCHS), Centers for Disease Control and Prevention (CDC), United States Census Bureau. United States National Hospital Discharge Survey 1981. Hyattsville, United States: National Center for Health Statistics (NCHS), Centers for Disease Control and Prevention (CDC)
Atrial fibrillation and flutter	National Center for Health Statistics (NCHS), Centers for Disease Control and Prevention (CDC), United States Census Bureau. United States National Hospital Discharge Survey 2007. Hyattsville, United States: National Center for Health Statistics (NCHS), Centers for Disease Control and Prevention (CDC)
Atrial fibrillation and flutter	Truven Health Analytics. United States MarketScan Claims and Medicare Data - 2000. Ann Arbor, United States: Truven Health Analytics
Atrial fibrillation and flutter	National Center for Health Statistics (NCHS), Centers for Disease Control and Prevention (CDC), United States Census Bureau. United States National Hospital Discharge Survey 1993. Hyattsville, United States: National Center for Health Statistics (NCHS), Centers for Disease Control and Prevention (CDC)
Atrial fibrillation and flutter	National Center for Health Statistics (NCHS), Centers for Disease Control and Prevention (CDC), US Census Bureau. United States National Health Interview Survey 1992. Hyattsville, United States: National Center for Health Statistics (NCHS), Centers for Disease Control and Prevention (CDC)
Atrial fibrillation and flutter	National Center for Health Statistics (NCHS), Centers for Disease Control and Prevention (CDC), United States Census Bureau. United States National Hospital Discharge Survey 1988. Hyattsville, United States: National Center for Health

© 2018 Global Burden of Cardiovascular Diseases Collaboration. *JAMA Cardiology*.

	Statistics (NCHS), Centers for Disease Control and Prevention (CDC)
Atrial fibrillation and flutter	National Center for Health Statistics (NCHS), Centers for Disease Control and Prevention (CDC), United States Census Bureau. United States National Hospital Discharge Survey 1989. Hyattsville, United States: National Center for Health Statistics (NCHS), Centers for Disease Control and Prevention (CDC)
Atrial fibrillation and flutter	National Center for Health Statistics (NCHS), Centers for Disease Control and Prevention (CDC), US Census Bureau. United States National Health Interview Survey 2004. Hyattsville, United States: National Center for Health Statistics (NCHS), Centers for Disease Control and Prevention (CDC)
Atrial fibrillation and flutter	Healthcare Cost and Utilization Project (HCUP), Agency for Healthcare Research and Quality (AHRQ). United States Nationwide Inpatient Sample 2013. Rockville, United States: Healthcare Cost and Utilization Project (HCUP), Agency for Healthcare Research and Quality (AHRQ)
Atrial fibrillation and flutter	National Center for Health Statistics (NCHS), Centers for Disease Control and Prevention (CDC), United States Census Bureau. United States National Hospital Discharge Survey 2010. Hyattsville, United States: National Center for Health Statistics (NCHS), Centers for Disease Control and Prevention (CDC)
Atrial fibrillation and flutter	Healthcare Cost and Utilization Project (HCUP), Agency for Healthcare Research and Quality (AHRQ). United States State Inpatient Databases 2008. Rockville, United States: Healthcare Cost and Utilization Project (HCUP), Agency for Healthcare Research and Quality (AHRQ)
Atrial fibrillation and flutter	National Center for Health Statistics (NCHS), Centers for Disease Control and Prevention (CDC). United States National Health and Nutrition Examination Survey 2005-2006. Hyattsville, United States: National Center for Health Statistics (NCHS), Centers for Disease Control and Prevention (CDC), 2007
Atrial fibrillation and flutter	National Center for Health Statistics (NCHS), Centers for Disease Control and Prevention (CDC), United States Census Bureau. United States National Hospital Discharge Survey 2000. Hyattsville, United States: National Center for Health Statistics (NCHS), Centers for Disease Control and Prevention (CDC)
Atrial fibrillation and flutter	National Center for Health Statistics (NCHS), Centers for Disease Control and Prevention (CDC), United States Census Bureau. United States National Hospital Discharge Survey 1982. Hyattsville, United States: National Center for Health Statistics (NCHS), Centers for Disease Control and Prevention (CDC)
Atrial fibrillation and flutter	Washington State University, World Health Organization (WHO). United States WHO Multi-country Survey Study on Health and Health System Responsiveness 2000-2001. Geneva, Switzerland: World Health Organization (WHO)
Atrial fibrillation and flutter	National Center for Health Statistics (NCHS), Centers for Disease Control and Prevention (CDC), United States Census Bureau. United States National Hospital Discharge Survey 2003. Hyattsville, United States: National Center for Health Statistics (NCHS), Centers for Disease Control and Prevention (CDC)
Atrial fibrillation and flutter	National Center for Health Statistics (NCHS), Centers for Disease Control and Prevention (CDC), United States Census Bureau. United States National Hospital Discharge Survey 1994. Hyattsville, United States: National Center for Health Statistics (NCHS), Centers for Disease Control and Prevention (CDC)
Atrial fibrillation and flutter	National Center for Health Statistics (NCHS), Centers for Disease Control and Prevention (CDC), United States Census Bureau. United States National Hospital Discharge Survey 2009. Hyattsville, United States: National Center for Health Statistics (NCHS), Centers for Disease Control and Prevention (CDC)
Atrial fibrillation and flutter	Healthcare Cost and Utilization Project (HCUP), Agency for Healthcare Research and Quality (AHRQ). United States Nationwide Inpatient Sample 2014. Rockville, United States: Healthcare Cost and Utilization Project (HCUP), Agency for Healthcare Research and Quality (AHRQ)
Atrial fibrillation and flutter	National Center for Health Statistics (NCHS), Centers for Disease Control and Prevention (CDC), United States Census Bureau. United States National Hospital Discharge Survey 2006. Hyattsville, United States: National Center for Health Statistics (NCHS), Centers for Disease Control and Prevention (CDC)
Atrial fibrillation and flutter	National Center for Health Statistics (NCHS), Centers for Disease Control and Prevention (CDC), US Census Bureau. United States National Health Interview Survey 1991. Hyattsville, United States: National Center for Health Statistics (NCHS), Centers for Disease Control and Prevention (CDC)
Atrial fibrillation and flutter	National Center for Health Statistics (NCHS), Centers for Disease Control and Prevention (CDC). United States National Health and Nutrition Examination Survey 2007-2008. Hyattsville, United States: National Center for Health Statistics (NCHS), Centers for Disease Control and Prevention (CDC), 2009
Atrial fibrillation and flutter	National Center for Health Statistics (NCHS), Centers for Disease Control and Prevention (CDC), United States Census Bureau. United States National Hospital Discharge Survey 1980. Hyattsville, United States: National Center for Health Statistics (NCHS), Centers for Disease Control and Prevention (CDC)
Atrial fibrillation and flutter	National Center for Health Statistics (NCHS), Centers for Disease Control and Prevention (CDC), United States Census Bureau. United States National Hospital Discharge Survey 1992. Hyattsville, United States: National Center for Health Statistics (NCHS), Centers for Disease Control and Prevention (CDC)
Atrial fibrillation and flutter	National Center for Health Statistics (NCHS), Centers for Disease Control and Prevention (CDC), US Census Bureau. United States National Health Interview Survey 1998. Hyattsville, United States: National Center for Health Statistics (NCHS), Centers for Disease Control and Prevention (CDC)
Atrial fibrillation and flutter	National Center for Health Statistics (NCHS), Centers for Disease Control and Prevention (CDC), United States Census Bureau. United States National Hospital Discharge Survey 1983. Hyattsville, United States: National Center for Health Statistics (NCHS), Centers for Disease Control and Prevention (CDC)
Atrial fibrillation and flutter	National Center for Health Statistics (NCHS), Centers for Disease Control and Prevention (CDC), US Census Bureau. United States National Health Interview Survey 2010. Hyattsville, United States: National Center for Health Statistics (NCHS), Centers for Disease Control and Prevention (CDC)
Atrial fibrillation and flutter	National Center for Health Statistics (NCHS), Centers for Disease Control and Prevention (CDC), US Census Bureau. United States National Health Interview Survey 2006. Hyattsville, United States: National Center for Health Statistics (NCHS), Centers for Disease Control and Prevention (CDC)

© 2018 Global Burden of Cardiovascular Diseases Collaboration. *JAMA Cardiology*.

Atrial fibrillation and flutter	National Center for Health Statistics (NCHS), Centers for Disease Control and Prevention (CDC), US Census Bureau. United States National Health Interview Survey 2000. Hyattsville, United States: National Center for Health Statistics (NCHS), Centers for Disease Control and Prevention (CDC)
Atrial fibrillation and flutter	National Center for Health Statistics (NCHS), Centers for Disease Control and Prevention (CDC), United States Census Bureau. United States National Health Interview Survey 2012. Hyattsville, United States: National Center for Health Statistics (NCHS), Centers for Disease Control and Prevention (CDC), 2013
Atrial fibrillation and flutter	National Center for Health Statistics (NCHS) Centers for Disease Control and Prevention (CDC). United States National Health and Nutrition Examination Survey 2011-2012. Hyattsville, United States: National Center for Health Statistics (NCHS) Centers for Disease Control and Prevention (CDC), 2013
Atrial fibrillation and flutter	National Center for Health Statistics (NCHS), Centers for Disease Control and Prevention (CDC), United States Census Bureau. United States National Hospital Discharge Survey 1979. Hyattsville, United States: National Center for Health Statistics (NCHS), Centers for Disease Control and Prevention (CDC)
Atrial fibrillation and flutter	National Center for Health Statistics (NCHS), Centers for Disease Control and Prevention (CDC), United States Census Bureau. United States National Hospital Discharge Survey 2004. Hyattsville, United States: National Center for Health Statistics (NCHS), Centers for Disease Control and Prevention (CDC)
Atrial fibrillation and flutter	National Center for Health Statistics (NCHS), Centers for Disease Control and Prevention (CDC). United States National Health and Nutrition Examination Survey 2001-2002. Hyattsville, United States: National Center for Health Statistics (NCHS), Centers for Disease Control and Prevention (CDC)
Atrial fibrillation and flutter	Healthcare Cost and Utilization Project (HCUP), Agency for Healthcare Research and Quality (AHRQ). United States Nationwide Inpatient Sample 2015. Rockville, United States: Healthcare Cost and Utilization Project (HCUP), Agency for Healthcare Research and Quality (AHRQ)
Atrial fibrillation and flutter	National Center for Health Statistics (NCHS), Centers for Disease Control and Prevention (CDC), US Census Bureau. United States National Health Interview Survey 2011. Hyattsville, United States: National Center for Health Statistics (NCHS), Centers for Disease Control and Prevention (CDC)
Atrial fibrillation and flutter	National Center for Health Statistics (NCHS), Centers for Disease Control and Prevention (CDC), United States Census Bureau. United States National Hospital Discharge Survey 1998. Hyattsville, United States: National Center for Health Statistics (NCHS), Centers for Disease Control and Prevention (CDC)
Atrial fibrillation and flutter	Miyasaka Y, Barnes ME, Gersh BJ, Cha SS, Bailey KR, Abhayaratna WP, Seward JB, Tsang TSM. Secular Trends in Incidence of Atrial Fibrillation in Olmsted County, Minnesota, 1980 to 2000, and Implications on the Projections for Future Prevalence. Circulation. 2006; 114(2): 119 -125
Atrial fibrillation and flutter	National Center for Health Statistics (NCHS), Centers for Disease Control and Prevention (CDC), United States Census Bureau. United States National Hospital Discharge Survey 1997. Hyattsville, United States: National Center for Health Statistics (NCHS), Centers for Disease Control and Prevention (CDC)
Atrial fibrillation and flutter	National Center for Health Statistics (NCHS), Centers for Disease Control and Prevention (CDC), United States Census Bureau. United States National Hospital Discharge Survey 2002. Hyattsville, United States: National Center for Health Statistics (NCHS), Centers for Disease Control and Prevention (CDC)
Atrial fibrillation and flutter	National Center for Health Statistics (NCHS), Centers for Disease Control and Prevention (CDC), United States Census Bureau. United States National Hospital Discharge Survey 1986. Hyattsville, United States: National Center for Health Statistics (NCHS), Centers for Disease Control and Prevention (CDC)
Atrial fibrillation and flutter	National Center for Health Statistics (NCHS), Centers for Disease Control and Prevention (CDC), US Census Bureau. United States National Health Interview Survey 1999. Hyattsville, United States: National Center for Health Statistics (NCHS), Centers for Disease Control and Prevention (CDC)
Atrial fibrillation and flutter	National Center for Health Statistics (NCHS), Centers for Disease Control and Prevention (CDC), United States Census Bureau. United States National Hospital Discharge Survey 1985. Hyattsville, United States: National Center for Health Statistics (NCHS), Centers for Disease Control and Prevention (CDC)
Atrial fibrillation and flutter	National Center for Health Statistics (NCHS), Centers for Disease Control and Prevention (CDC), US Census Bureau. United States National Health Interview Survey 1993. Hyattville, United States: National Center for Health Statistics (NCHS), Centers for Disease Control and Prevention (CDC)
Atrial fibrillation and flutter	National Center for Health Statistics (NCHS), Centers for Disease Control and Prevention (CDC), United States Census Bureau. United States National Hospital Discharge Survey 2001. Hyattsville, United States: National Center for Health Statistics (NCHS), Centers for Disease Control and Prevention (CDC)
Atrial fibrillation and flutter	National Center for Health Statistics (NCHS), Centers for Disease Control and Prevention (CDC), United States Census Bureau. United States National Hospital Discharge Survey 1984. Hyattsville, United States: National Center for Health Statistics (NCHS), Centers for Disease Control and Prevention (CDC)
Atrial fibrillation and flutter	National Center for Health Statistics (NCHS), Centers for Disease Control and Prevention (CDC), United States Census Bureau. United States National Hospital Discharge Survey 1995. Hyattsville, United States: National Center for Health Statistics (NCHS), Centers for Disease Control and Prevention (CDC)
Atrial fibrillation and flutter	Panel Study of Income Dynamics, 2007 public use dataset. Produced and distributed by the University of Michigan with primary funding from the National Science Foundation, the National Institute of Aging, and the National Institute of Child Health and Human Development. Ann Arbor, MI, (2011)
Atrial fibrillation and flutter	National Center for Health Statistics (NCHS), Centers for Disease Control and Prevention (CDC), United States Census Bureau. United States National Hospital Discharge Survey 1996. Hyattsville, United States: National Center for Health Statistics (NCHS), Centers for Disease Control and Prevention (CDC)
Atrial fibrillation and flutter	National Center for Health Statistics (NCHS), Centers for Disease Control and Prevention (CDC), US Census Bureau. United States National Health Interview Survey 2002. Hyattsville, United States: National Center for Health Statistics (NCHS), Centers for Disease Control and Prevention (CDC)
Atrial fibrillation and flutter	National Center for Health Statistics (NCHS), Centers for Disease Control and Prevention (CDC). United States National Health and Nutrition Examination Survey 2013-2014. Hyattsville, United States: National Center for Health Statistics (NCHS), Centers for Disease Control and Prevention (CDC)

© 2018 Global Burden of Cardiovascular Diseases Collaboration. *JAMA Cardiology*.

Atrial fibrillation and flutter	National Center for Health Statistics (NCHS), Centers for Disease Control and Prevention (CDC). United States National Health and Nutrition Examination Survey 2003-2004. Hyattsville, United States: National Center for Health Statistics (NCHS), Centers for Disease Control and Prevention (CDC)
Atrial fibrillation and flutter	National Center for Health Statistics (NCHS), Centers for Disease Control and Prevention (CDC), United States Census Bureau. United States National Hospital Discharge Survey 2008. Hyattsville, United States: National Center for Health Statistics (NCHS), Centers for Disease Control and Prevention (CDC)
Atrial fibrillation and flutter	Furberg CD, Psaty BM, Manolio TA, Gardin JM, Smith VE, Rautaharju PM. Prevalence of atrial fibrillation in elderly subjects (the Cardiovascular Health Study). Am J Cardiol. 1994; 74(3): 236-41
Atrial fibrillation and flutter	National Center for Health Statistics (NCHS), Centers for Disease Control and Prevention (CDC), United States Census Bureau. United States National Health Interview Survey 2013. Hyattsville, United States: National Center for Health Statistics (NCHS), Centers for Disease Control and Prevention (CDC), 2014
Atrial fibrillation and flutter	National Center for Health Statistics (NCHS), Centers for Disease Control and Prevention (CDC). United States National Health and Nutrition Examination Survey 1988-1994. Hyattsville, United States: National Center for Health Statistics (NCHS), Centers for Disease Control and Prevention (CDC)
Atrial fibrillation and flutter	National Center for Health Statistics (NCHS), Centers for Disease Control and Prevention (CDC), US Census Bureau. United States National Health Interview Survey 2007. Hyattsville, United States: National Center for Health Statistics (NCHS), Centers for Disease Control and Prevention (CDC)
Atrial fibrillation and flutter	Prineas RJ, Le A, Soliman EZ, Zhang Z-M, Howard VJ, Ostchega Y, Howard G, Reasons for Geographic and Racial Differences in Stroke (REGARDS) Investigators. United States national prevalence of electrocardiographic abnormalities in black and white middle-age (45- to 64-Year) and older (?65-Year) adults (from the Reasons for Geographic and Racial Differences in Stroke Study). Am J Cardiol. 2012; 109(8): 1223Â–8
Atrial fibrillation and flutter	National Center for Health Statistics (NCHS), Centers for Disease Control and Prevention (CDC), US Census Bureau. United States National Health Interview Survey 2003. Hyattsville, United States: National Center for Health Statistics (NCHS), Centers for Disease Control and Prevention (CDC)
Atrial fibrillation and flutter	National Center for Health Statistics (NCHS), Centers for Disease Control and Prevention (CDC), US Census Bureau. United States National Health Interview Survey 2009. Hyattsville, United States: National Center for Health Statistics (NCHS), Centers for Disease Control and Prevention (CDC)
Atrial fibrillation and flutter	Vaziri S, Larson M, Benjamin E, Levy D. Echocardiographic predictors of nonrheumatic atrial fibrillation. The Framingham Heart Study. Circulation. 1994; 89(2): 724 -730
Atrial fibrillation and flutter	Benjamin EJ, Wolf PA, D'Agostino RB, Silbershatz H, Kannel WB, Levy D. Impact of atrial fibrillation on the risk of death: the Framingham Heart Study. Circulation. 1998; 98(10): 946-52
Atrial fibrillation and flutter	Go AS, Hylek EM, Phillips KA, Chang Y, Henault LE, Selby JV, Singer DE. Prevalence of diagnosed atrial fibrillation in adults: National implications for rhythm management and stroke prevention. JAMA. 2001; 285(18): 2370 -2375
Atrial fibrillation and flutter	National Center for Health Statistics (NCHS), Centers for Disease Control and Prevention (CDC), US Census Bureau. United States National Health Interview Survey 1997. Hyattsville, United States: National Center for Health Statistics (NCHS), Centers for Disease Control and Prevention (CDC)
Atrial fibrillation and flutter	Upshaw CB. Reduced prevalence of atrial fibrillation in black patients compared with white patients attending an urban hospital: an electrocardiographic study. J Natl Med Assoc. 2002; 94(4): 204-8
Atrial fibrillation and flutter	National Center for Health Statistics (NCHS), Centers for Disease Control and Prevention (CDC). United States National Health and Nutrition Examination Survey 2009-2010. Hyattsville, United States: National Center for Health Statistics (NCHS), Centers for Disease Control and Prevention (CDC), 2011
Atrial fibrillation and flutter	Go AS, Hylek EM, Phillips KA, Chang Y, Henault LE, Selby JV, Singer DE. Prevalence of diagnosed atrial fibrillation in adults: national implications for rhythm management and stroke prevention: the AnTicoagulation and Risk Factors in Atrial Fibrillation (ATRIA) Study. JAMA. 2001; 285(18): 2370Â–5
Atrial fibrillation and flutter	National Center for Health Statistics (NCHS), Centers for Disease Control and Prevention (CDC), US Census Bureau. United States National Health Interview Survey 2008. Hyattsville, United States: National Center for Health Statistics (NCHS), Centers for Disease Control and Prevention (CDC)
Atrial fibrillation and flutter	United States Department of Health and Human Services. Centers for Disease Control and Prevention. National Center for Health Statistics. National Health Interview Survey, 1994: Second Supplement on Aging. ICPSR02563-v3. Ann Arbor, MI: Inter-university Consortium for Political and Social Research [distributor], 2007-02-12. http://doi.org/10.3886/ICPSR02563.v3
Atrial fibrillation and flutter	National Center for Health Statistics (NCHS), Centers for Disease Control and Prevention (CDC). United States National Health and Nutrition Examination Survey 1999-2000. Hyattsville, United States: National Center for Health Statistics (NCHS), Centers for Disease Control and Prevention (CDC)
Atrial fibrillation and flutter	National Center for Health Statistics (NCHS), Centers for Disease Control and Prevention (CDC), US Census Bureau. United States National Health Interview Survey 2001. Hyattsville, United States: National Center for Health Statistics (NCHS), Centers for Disease Control and Prevention (CDC)
Atrial fibrillation and flutter	Lakshminarayan K, Solid CA, Collins AJ, Anderson DC, Herzog CA. Atrial fibrillation and stroke in the general Medicare population: a 10-year perspective (1992 to 2002). Stroke. 2006; 37(8): 1969-74
Atrial fibrillation and flutter	United States Department of Health and Human Services. Centers for Disease Control and Prevention. National Center for Health Statistics. National Health Interview Survey, 1994: Second Longitudinal Study on Aging, Wave 2, 1997. ICPSR03526-v2. Ann Arbor, MI: Inter-university Consortium for Political and Social Research [distributor], 2007-03-01. http://doi.org/10.3886/ICPSR03526.v2
Atrial fibrillation and flutter	National Center for Health Statistics (NCHS), Centers for Disease Control and Prevention (CDC), US Census Bureau. United States National Health Interview Survey 1990. Hyattsville, United States: National Center for Health Statistics (NCHS), Centers for Disease Control and Prevention (CDC)
Atrial fibrillation and flutter	Schnabel RB, Yin X, Gona P, Larson MG, Beiser AS, McManus DD, Newton-Cheh C, Lubitz SA, Magnani JW, Ellinor PT, Seshadri S, Wolf PA, Vasan RS, Benjamin EJ, Levy D. 50 year trends in atrial fibrillation prevalence, incidence, risk factors, and mortality in the Framingham Heart Study: a cohort study. Lancet. 2015; 386(9989): 154Â–62

© 2018 Global Burden of Cardiovascular Diseases Collaboration. *JAMA Cardiology*.

Atrial fibrillation and flutter	National Center for Health Statistics (NCHS), Centers for Disease Control and Prevention (CDC). United States National Health Interview Survey 2014. Hyattsville, United States: National Center for Health Statistics (NCHS), Centers for Disease Control and Prevention (CDC), 2015
Atrial fibrillation and flutter	Baber U, Howard VJ, Halperin JL, Soliman EZ, Zhang X, McClellan W, Warnock DG, Muntner P. Association of chronic kidney disease with atrial fibrillation among adults in the United States: REasons for Geographic and Racial Differences in Stroke (REGARDS) Study. Circ Arrhythm Electrophysiol. 2011; 4(1): 26Â–32
Atrial fibrillation and flutter	Panel Study of Income Dynamics, 2005 public use dataset. Produced and distributed by the University of Michigan with primary funding from the National Science Foundation, the National Institute of Aging, and the National Institute of Child Health and Human Development. Ann Arbor, MI, (2011)
Atrial fibrillation and flutter	Misialek JR, Rose KM, Everson-Rose SA, Soliman EZ, Clark CJ, Lopez FL, Alonso A. Socioeconomic status and the incidence of atrial fibrillation in whites and blacks: the Atherosclerosis Risk in Communities (ARIC) study. J Am Heart Assoc. 2014; 3(4)
Atrial fibrillation and flutter	Fretts AM, Mozaffarian D, Siscovick DS, Heckbert SR, McKnight B, King IB, Rimm EB, Psaty BM, Sacks FM, Song X, Spiegelman D, Lemaitre RN. Associations of plasma phospholipid and dietary alpha linolenic acid with incident atrial fibrillation in older adults: the Cardiovascular Health Study. J Am Heart Assoc. 2013; 2(1): e003814
Endocarditis	Healthcare Cost and Utilization Project (HCUP), Agency for Healthcare Research and Quality (AHRQ). United States State Inpatient Databases 2014. Rockville, United States: Healthcare Cost and Utilization Project (HCUP), Agency for Healthcare Research and Quality (AHRQ)
Endocarditis	Healthcare Cost and Utilization Project (HCUP), Agency for Healthcare Research and Quality (AHRQ). United States State Inpatient Databases 2012. Rockville, United States: Healthcare Cost and Utilization Project (HCUP), Agency for Healthcare Research and Quality (AHRQ)
Endocarditis	Healthcare Cost and Utilization Project (HCUP), Agency for Healthcare Research and Quality (AHRQ). United States State Inpatient Databases 2013. Rockville, United States: Healthcare Cost and Utilization Project (HCUP), Agency for Healthcare Research and Quality (AHRQ)
Endocarditis	Healthcare Cost and Utilization Project (HCUP), Agency for Healthcare Research and Quality (AHRQ). United States State Inpatient Databases 2011. Rockville, United States: Healthcare Cost and Utilization Project (HCUP), Agency for Healthcare Research and Quality (AHRQ)
Endocarditis	Healthcare Cost and Utilization Project (HCUP), Agency for Healthcare Research and Quality (AHRQ). United States State Inpatient Databases 2009. Rockville, United States: Healthcare Cost and Utilization Project (HCUP), Agency for Healthcare Research and Quality (AHRQ)
Endocarditis	Healthcare Cost and Utilization Project (HCUP), Agency for Healthcare Research and Quality (AHRQ). United States State Inpatient Databases 2005. Rockville, United States: Healthcare Cost and Utilization Project (HCUP), Agency for Healthcare Research and Quality (AHRQ)
Endocarditis	Truven Health Analytics. United States MarketScan Claims and Medicare Data - 2012. Ann Arbor, United States: Truven Health Analytics
Endocarditis	National Center for Health Statistics (NCHS), Centers for Disease Control and Prevention (CDC), United States Census Bureau. United States National Hospital Discharge Survey 1981. Hyattsville, United States: National Center for Health Statistics (NCHS), Centers for Disease Control and Prevention (CDC)
Endocarditis	Healthcare Cost and Utilization Project (HCUP), Agency for Healthcare Research and Quality (AHRQ). United States Nationwide Inpatient Sample 2014. Rockville, United States: Healthcare Cost and Utilization Project (HCUP), Agency for Healthcare Research and Quality (AHRQ)
Endocarditis	Healthcare Cost and Utilization Project (HCUP), Agency for Healthcare Research and Quality (AHRQ). United States State Inpatient Databases 2008. Rockville, United States: Healthcare Cost and Utilization Project (HCUP), Agency for Healthcare Research and Quality (AHRQ)
Endocarditis	National Center for Health Statistics (NCHS), Centers for Disease Control and Prevention (CDC), United States Census Bureau. United States National Hospital Discharge Survey 2006. Hyattsville, United States: National Center for Health Statistics (NCHS), Centers for Disease Control and Prevention (CDC)
Endocarditis	Healthcare Cost and Utilization Project (HCUP), Agency for Healthcare Research and Quality (AHRQ). United States State Inpatient Databases 2003. Rockville, United States: Healthcare Cost and Utilization Project (HCUP), Agency for Healthcare Research and Quality (AHRQ)
Endocarditis	Healthcare Cost and Utilization Project (HCUP), Agency for Healthcare Research and Quality (AHRQ). United States State Inpatient Databases 2006. Rockville, United States: Healthcare Cost and Utilization Project (HCUP), Agency for Healthcare Research and Quality (AHRQ)
Endocarditis	National Center for Health Statistics (NCHS), Centers for Disease Control and Prevention (CDC), US Census Bureau. United States National Health Interview Survey 1997. Hyattsville, United States: National Center for Health Statistics (NCHS), Centers for Disease Control and Prevention (CDC)
Endocarditis	Healthcare Cost and Utilization Project (HCUP), Agency for Healthcare Research and Quality (AHRQ). United States Nationwide Inpatient Sample 2015. Rockville, United States: Healthcare Cost and Utilization Project (HCUP), Agency for Healthcare Research and Quality (AHRQ)
Endocarditis	Truven Health Analytics. United States MarketScan Claims and Medicare Data - 2000. Ann Arbor, United States: Truven Health Analytics
Endocarditis	Healthcare Cost and Utilization Project (HCUP), Agency for Healthcare Research and Quality (AHRQ). United States State Inpatient Databases 2004. Rockville, United States: Healthcare Cost and Utilization Project (HCUP), Agency for Healthcare Research and Quality (AHRQ)
Endocarditis	National Center for Health Statistics (NCHS), Centers for Disease Control and Prevention (CDC), United States Census Bureau. United States National Hospital Discharge Survey 2007. Hyattsville, United States: National Center for Health Statistics (NCHS), Centers for Disease Control and Prevention (CDC)

© 2018 Global Burden of Cardiovascular Diseases Collaboration. *JAMA Cardiology*.

Endocarditis	National Center for Health Statistics (NCHS), Centers for Disease Control and Prevention (CDC), United States Census Bureau. United States National Hospital Discharge Survey 2003. Hyattsville, United States: National Center for Health Statistics (NCHS), Centers for Disease Control and Prevention (CDC)
Endocarditis	National Center for Health Statistics (NCHS), Centers for Disease Control and Prevention (CDC), United States Census Bureau. United States National Hospital Discharge Survey 1991. Hyattsville, United States: National Center for Health Statistics (NCHS), Centers for Disease Control and Prevention (CDC)
Endocarditis	Healthcare Cost and Utilization Project (HCUP), Agency for Healthcare Research and Quality (AHRQ). United States State Inpatient Databases 2007. Rockville, United States: Healthcare Cost and Utilization Project (HCUP), Agency for Healthcare Research and Quality (AHRQ)
Endocarditis	National Center for Health Statistics (NCHS), Centers for Disease Control and Prevention (CDC), United States Census Bureau. United States National Hospital Discharge Survey 1984. Hyattsville, United States: National Center for Health Statistics (NCHS), Centers for Disease Control and Prevention (CDC)
Endocarditis	National Center for Health Statistics (NCHS), Centers for Disease Control and Prevention (CDC), United States Census Bureau. United States National Hospital Discharge Survey 2009. Hyattsville, United States: National Center for Health Statistics (NCHS), Centers for Disease Control and Prevention (CDC)
Endocarditis	National Center for Health Statistics (NCHS), Centers for Disease Control and Prevention (CDC), United States Census Bureau. United States National Hospital Discharge Survey 1999. Hyattsville, United States: National Center for Health Statistics (NCHS), Centers for Disease Control and Prevention (CDC)
Endocarditis	National Center for Health Statistics (NCHS), Centers for Disease Control and Prevention (CDC), United States Census Bureau. United States National Hospital Discharge Survey 1993. Hyattsville, United States: National Center for Health Statistics (NCHS), Centers for Disease Control and Prevention (CDC)
Endocarditis	National Center for Health Statistics (NCHS), Centers for Disease Control and Prevention (CDC), United States Census Bureau. United States National Hospital Discharge Survey 1987. Hyattsville, United States: National Center for Health Statistics (NCHS), Centers for Disease Control and Prevention (CDC)
Endocarditis	Truven Health Analytics. United States MarketScan Claims and Medicare Data - 2010. Ann Arbor, United States: Truven Health Analytics
Endocarditis	National Center for Health Statistics (NCHS), Centers for Disease Control and Prevention (CDC), United States Census Bureau. United States National Hospital Discharge Survey 1979. Hyattsville, United States: National Center for Health Statistics (NCHS), Centers for Disease Control and Prevention (CDC)
Endocarditis	National Center for Health Statistics (NCHS), Centers for Disease Control and Prevention (CDC), United States Census Bureau. United States National Hospital Discharge Survey 1986. Hyattsville, United States: National Center for Health Statistics (NCHS), Centers for Disease Control and Prevention (CDC)
Endocarditis	National Center for Health Statistics (NCHS), Centers for Disease Control and Prevention (CDC), United States Census Bureau. United States National Hospital Discharge Survey 2010. Hyattsville, United States: National Center for Health Statistics (NCHS), Centers for Disease Control and Prevention (CDC)
Endocarditis	Healthcare Cost and Utilization Project (HCUP), Agency for Healthcare Research and Quality (AHRQ). United States State Inpatient Databases 2008-2009
Endocarditis	National Center for Health Statistics (NCHS), Centers for Disease Control and Prevention (CDC), United States Census Bureau. United States National Hospital Discharge Survey 1992. Hyattsville, United States: National Center for Health Statistics (NCHS), Centers for Disease Control and Prevention (CDC)
Endocarditis	National Center for Health Statistics (NCHS), Centers for Disease Control and Prevention (CDC), US Census Bureau. United States National Health Interview Survey 2004. Hyattsville, United States: National Center for Health Statistics (NCHS), Centers for Disease Control and Prevention (CDC)
Endocarditis	Healthcare Cost and Utilization Project (HCUP), Agency for Healthcare Research and Quality (AHRQ). United States State Inpatient Databases 2003-2007
Endocarditis	National Center for Health Statistics (NCHS), Centers for Disease Control and Prevention (CDC), US Census Bureau. United States National Health Interview Survey 1991. Hyattsville, United States: National Center for Health Statistics (NCHS), Centers for Disease Control and Prevention (CDC)
Endocarditis	National Center for Health Statistics (NCHS), Centers for Disease Control and Prevention (CDC). United States National Health Interview Survey 2014. Hyattsville, United States: National Center for Health Statistics (NCHS), Centers for Disease Control and Prevention (CDC), 2015
Endocarditis	National Center for Health Statistics (NCHS), Centers for Disease Control and Prevention (CDC), United States Census Bureau. United States National Hospital Discharge Survey 1994. Hyattsville, United States: National Center for Health Statistics (NCHS), Centers for Disease Control and Prevention (CDC)
Endocarditis	National Center for Health Statistics (NCHS), Centers for Disease Control and Prevention (CDC), United States Census Bureau. United States National Hospital Discharge Survey 1988. Hyattsville, United States: National Center for Health Statistics (NCHS), Centers for Disease Control and Prevention (CDC)
Endocarditis	National Center for Health Statistics (NCHS), Centers for Disease Control and Prevention (CDC). United States National Health and Nutrition Examination Survey 2005-2006. Hyattsville, United States: National Center for Health Statistics (NCHS), Centers for Disease Control and Prevention (CDC), 2007
Endocarditis	National Center for Health Statistics (NCHS), Centers for Disease Control and Prevention (CDC), United States Census Bureau. United States National Hospital Discharge Survey 1985. Hyattsville, United States: National Center for Health Statistics (NCHS), Centers for Disease Control and Prevention (CDC)
Endocarditis	National Center for Health Statistics (NCHS), Centers for Disease Control and Prevention (CDC), United States Census Bureau. United States National Hospital Discharge Survey 2004. Hyattsville, United States: National Center for Health Statistics (NCHS), Centers for Disease Control and Prevention (CDC)
Endocarditis	National Center for Health Statistics (NCHS), Centers for Disease Control and Prevention (CDC). United States National Health and Nutrition Examination Survey 2001-2002. Hyattsville, United States: National Center for Health Statistics (NCHS), Centers for Disease Control and Prevention (CDC)

© 2018 Global Burden of Cardiovascular Diseases Collaboration. *JAMA Cardiology*.

Endocarditis	National Center for Health Statistics (NCHS), Centers for Disease Control and Prevention (CDC), US Census Bureau. United States National Health Interview Survey 1992. Hyattsville, United States: National Center for Health Statistics (NCHS), Centers for Disease Control and Prevention (CDC)
Endocarditis	Healthcare Cost and Utilization Project (HCUP), Agency for Healthcare Research and Quality (AHRQ). United States Nationwide Inpatient Sample 2013. Rockville, United States: Healthcare Cost and Utilization Project (HCUP), Agency for Healthcare Research and Quality (AHRQ)
Endocarditis	National Center for Health Statistics (NCHS), Centers for Disease Control and Prevention (CDC), United States Census Bureau. United States National Hospital Discharge Survey 1990. Hyattsville, United States: National Center for Health Statistics (NCHS), Centers for Disease Control and Prevention (CDC)
Endocarditis	Panel Study of Income Dynamics, 2007 public use dataset. Produced and distributed by the University of Michigan with primary funding from the National Science Foundation, the National Institute of Aging, and the National Institute of Child Health and Human Development. Ann Arbor, MI, (2011)
Endocarditis	National Center for Health Statistics (NCHS), Centers for Disease Control and Prevention (CDC). United States National Hospital Discharge Survey 2008-2010
Endocarditis	Panel Study of Income Dynamics, 2005 public use dataset. Produced and distributed by the University of Michigan with primary funding from the National Science Foundation, the National Institute of Aging, and the National Institute of Child Health and Human Development. Ann Arbor, MI, (2011)
Endocarditis	National Center for Health Statistics (NCHS), Centers for Disease Control and Prevention (CDC), United States Census Bureau. United States National Hospital Discharge Survey 2008. Hyattsville, United States: National Center for Health Statistics (NCHS), Centers for Disease Control and Prevention (CDC)
Endocarditis	National Center for Health Statistics (NCHS), Centers for Disease Control and Prevention (CDC), United States Census Bureau. United States National Hospital Discharge Survey 1980. Hyattsville, United States: National Center for Health Statistics (NCHS), Centers for Disease Control and Prevention (CDC)
Endocarditis	National Center for Health Statistics (NCHS), Centers for Disease Control and Prevention (CDC), United States Census Bureau. United States National Health Interview Survey 2012. Hyattsville, United States: National Center for Health Statistics (NCHS), Centers for Disease Control and Prevention (CDC), 2013
Endocarditis	National Center for Health Statistics (NCHS), Centers for Disease Control and Prevention (CDC), United States Census Bureau. United States National Hospital Discharge Survey 1998. Hyattsville, United States: National Center for Health Statistics (NCHS), Centers for Disease Control and Prevention (CDC)
Endocarditis	National Center for Health Statistics (NCHS), Centers for Disease Control and Prevention (CDC), United States Census Bureau. United States National Hospital Discharge Survey 1983. Hyattsville, United States: National Center for Health Statistics (NCHS), Centers for Disease Control and Prevention (CDC)
Endocarditis	National Center for Health Statistics (NCHS), Centers for Disease Control and Prevention (CDC), United States Census Bureau. United States National Hospital Discharge Survey 1982. Hyattsville, United States: National Center for Health Statistics (NCHS), Centers for Disease Control and Prevention (CDC)
Endocarditis	National Center for Health Statistics (NCHS) Centers for Disease Control and Prevention (CDC). United States National Health and Nutrition Examination Survey 2011-2012. Hyattsville, United States: National Center for Health Statistics (NCHS) Centers for Disease Control and Prevention (CDC), 2013
Endocarditis	National Center for Health Statistics (NCHS), Centers for Disease Control and Prevention (CDC), US Census Bureau. United States National Health Interview Survey 2003. Hyattsville, United States: National Center for Health Statistics (NCHS), Centers for Disease Control and Prevention (CDC)
Endocarditis	National Center for Health Statistics (NCHS), Centers for Disease Control and Prevention (CDC), United States Census Bureau. United States National Hospital Discharge Survey 1989. Hyattsville, United States: National Center for Health Statistics (NCHS), Centers for Disease Control and Prevention (CDC)
Endocarditis	National Center for Health Statistics (NCHS), Centers for Disease Control and Prevention (CDC), United States Census Bureau. United States National Hospital Discharge Survey 2001. Hyattsville, United States: National Center for Health Statistics (NCHS), Centers for Disease Control and Prevention (CDC)
Endocarditis	National Center for Health Statistics (NCHS), Centers for Disease Control and Prevention (CDC), US Census Bureau. United States National Health Interview Survey 1993. Hyattville, United States: National Center for Health Statistics (NCHS), Centers for Disease Control and Prevention (CDC)
Endocarditis	National Center for Health Statistics (NCHS), Centers for Disease Control and Prevention (CDC), US Census Bureau. United States National Health Interview Survey 2001. Hyattsville, United States: National Center for Health Statistics (NCHS), Centers for Disease Control and Prevention (CDC)
Endocarditis	National Center for Health Statistics (NCHS), Centers for Disease Control and Prevention (CDC), United States Census Bureau. United States National Hospital Discharge Survey 2005. Hyattsville, United States: National Center for Health Statistics (NCHS), Centers for Disease Control and Prevention (CDC)
Endocarditis	National Center for Health Statistics (NCHS), Centers for Disease Control and Prevention (CDC), US Census Bureau. United States National Health Interview Survey 2011. Hyattsville, United States: National Center for Health Statistics (NCHS), Centers for Disease Control and Prevention (CDC)
Endocarditis	National Center for Health Statistics (NCHS), Centers for Disease Control and Prevention (CDC), United States Census Bureau. United States National Hospital Discharge Survey 1997. Hyattsville, United States: National Center for Health Statistics (NCHS), Centers for Disease Control and Prevention (CDC)
Endocarditis	National Center for Health Statistics (NCHS), Centers for Disease Control and Prevention (CDC), US Census Bureau. United States National Health Interview Survey 1999. Hyattsville, United States: National Center for Health Statistics (NCHS), Centers for Disease Control and Prevention (CDC)
Endocarditis	National Center for Health Statistics (NCHS), Centers for Disease Control and Prevention (CDC), United States Census Bureau. United States National Hospital Discharge Survey 2000. Hyattsville, United States: National Center for Health Statistics (NCHS), Centers for Disease Control and Prevention (CDC)

© 2018 Global Burden of Cardiovascular Diseases Collaboration. *JAMA Cardiology*.

Endocarditis	Washington State University, World Health Organization (WHO). United States WHO Multi-country Survey Study on Health and Health System Responsiveness 2000-2001. Geneva, Switzerland: World Health Organization (WHO)
Endocarditis	National Center for Health Statistics (NCHS), Centers for Disease Control and Prevention (CDC), United States Census Bureau. United States National Hospital Discharge Survey 1996. Hyattsville, United States: National Center for Health Statistics (NCHS), Centers for Disease Control and Prevention (CDC)
Endocarditis	National Center for Health Statistics (NCHS), Centers for Disease Control and Prevention (CDC), United States Census Bureau. United States National Hospital Discharge Survey 2002. Hyattsville, United States: National Center for Health Statistics (NCHS), Centers for Disease Control and Prevention (CDC)
Endocarditis	National Center for Health Statistics (NCHS), Centers for Disease Control and Prevention (CDC), United States Census Bureau. United States National Hospital Discharge Survey 1995. Hyattsville, United States: National Center for Health Statistics (NCHS), Centers for Disease Control and Prevention (CDC)
Endocarditis	National Center for Health Statistics (NCHS), Centers for Disease Control and Prevention (CDC). United States National Health and Nutrition Examination Survey 2007-2008. Hyattsville, United States: National Center for Health Statistics (NCHS), Centers for Disease Control and Prevention (CDC), 2009
Endocarditis	National Center for Health Statistics (NCHS), Centers for Disease Control and Prevention (CDC). United States National Health and Nutrition Examination Survey 2013-2014. Hyattsville, United States: National Center for Health Statistics (NCHS), Centers for Disease Control and Prevention (CDC)
Endocarditis	National Center for Health Statistics (NCHS), Centers for Disease Control and Prevention (CDC). United States National Health and Nutrition Examination Survey 2003-2004. Hyattsville, United States: National Center for Health Statistics (NCHS), Centers for Disease Control and Prevention (CDC)
Endocarditis	National Center for Health Statistics (NCHS), Centers for Disease Control and Prevention (CDC), US Census Bureau. United States National Health Interview Survey 2010. Hyattsville, United States: National Center for Health Statistics (NCHS), Centers for Disease Control and Prevention (CDC)
Endocarditis	National Center for Health Statistics (NCHS), Centers for Disease Control and Prevention (CDC). United States National Health and Nutrition Examination Survey 1988-1994. Hyattsville, United States: National Center for Health Statistics (NCHS), Centers for Disease Control and Prevention (CDC)
Endocarditis	National Center for Health Statistics (NCHS), Centers for Disease Control and Prevention (CDC), United States Census Bureau. United States National Health Interview Survey 2013. Hyattsville, United States: National Center for Health Statistics (NCHS), Centers for Disease Control and Prevention (CDC), 2014
Endocarditis	United States Department of Health and Human Services. Centers for Disease Control and Prevention. National Center for Health Statistics. National Health Interview Survey, 1994: Second Supplement on Aging. ICPSR02563-v3. Ann Arbor, MI: Inter-university Consortium for Political and Social Research [distributor], 2007-02-12. http://doi.org/10.3886/ICPSR02563.v3
Endocarditis	National Center for Health Statistics (NCHS), Centers for Disease Control and Prevention (CDC), US Census Bureau. United States National Health Interview Survey 2006. Hyattsville, United States: National Center for Health Statistics (NCHS), Centers for Disease Control and Prevention (CDC)
Endocarditis	National Center for Health Statistics (NCHS), Centers for Disease Control and Prevention (CDC). United States National Health and Nutrition Examination Survey 2009-2010. Hyattsville, United States: National Center for Health Statistics (NCHS), Centers for Disease Control and Prevention (CDC), 2011
Endocarditis	National Center for Health Statistics (NCHS), Centers for Disease Control and Prevention (CDC). United States National Health and Nutrition Examination Survey 1999-2000. Hyattsville, United States: National Center for Health Statistics (NCHS), Centers for Disease Control and Prevention (CDC)
Endocarditis	National Center for Health Statistics (NCHS), Centers for Disease Control and Prevention (CDC), US Census Bureau. United States National Health Interview Survey 2007. Hyattsville, United States: National Center for Health Statistics (NCHS), Centers for Disease Control and Prevention (CDC)
Endocarditis	National Center for Health Statistics (NCHS), Centers for Disease Control and Prevention (CDC), US Census Bureau. United States National Health Interview Survey 2008. Hyattsville, United States: National Center for Health Statistics (NCHS), Centers for Disease Control and Prevention (CDC)
Endocarditis	National Center for Health Statistics (NCHS), Centers for Disease Control and Prevention (CDC), US Census Bureau. United States National Health Interview Survey 1990. Hyattsville, United States: National Center for Health Statistics (NCHS), Centers for Disease Control and Prevention (CDC)
Endocarditis	United States Department of Health and Human Services. Centers for Disease Control and Prevention. National Center for Health Statistics. National Health Interview Survey, 1994: Second Longitudinal Study on Aging, Wave 2, 1997. ICPSR03526-v2. Ann Arbor, MI: Inter-university Consortium for Political and Social Research [distributor], 2007-03-01. http://doi.org/10.3886/ICPSR03526.v2
Endocarditis	National Center for Health Statistics (NCHS), Centers for Disease Control and Prevention (CDC). United States National Hospital Discharge Survey 1993-1997
Endocarditis	National Center for Health Statistics (NCHS), Centers for Disease Control and Prevention (CDC), US Census Bureau. United States National Health Interview Survey 2009. Hyattsville, United States: National Center for Health Statistics (NCHS), Centers for Disease Control and Prevention (CDC)
Endocarditis	National Center for Health Statistics (NCHS), Centers for Disease Control and Prevention (CDC), US Census Bureau. United States National Health Interview Survey 2002. Hyattsville, United States: National Center for Health Statistics (NCHS), Centers for Disease Control and Prevention (CDC)
Endocarditis	National Center for Health Statistics (NCHS), Centers for Disease Control and Prevention (CDC), US Census Bureau. United States National Health Interview Survey 2000. Hyattsville, United States: National Center for Health Statistics (NCHS), Centers for Disease Control and Prevention (CDC)
Endocarditis	National Center for Health Statistics (NCHS), Centers for Disease Control and Prevention (CDC), US Census Bureau. United States National Health Interview Survey 1998. Hyattsville, United States: National Center for Health Statistics (NCHS), Centers for Disease Control and Prevention (CDC)

© 2018 Global Burden of Cardiovascular Diseases Collaboration. *JAMA Cardiology*.

Endocarditis	National Center for Health Statistics (NCHS), Centers for Disease Control and Prevention (CDC). United States National Hospital Discharge Survey 2003-2007
Endocarditis	National Center for Health Statistics (NCHS), Centers for Disease Control and Prevention (CDC). United States National Hospital Discharge Survey 1988-1992
Endocarditis	National Center for Health Statistics (NCHS), Centers for Disease Control and Prevention (CDC). United States National Hospital Discharge Survey 1998-2002
Heart failure	Truven Health Analytics. United States MarketScan Claims and Medicare Data - 2000. Ann Arbor, United States: Truven Health Analytics
Heart failure	Truven Health Analytics. United States MarketScan Claims and Medicare Data - 2010. Ann Arbor, United States: Truven Health Analytics
Heart failure	Truven Health Analytics. United States MarketScan Claims and Medicare Data - 2012. Ann Arbor, United States: Truven Health Analytics
Hemorrhagic Stroke	Healthcare Cost and Utilization Project (HCUP), Agency for Healthcare Research and Quality (AHRQ). United States State Inpatient Databases 2011. Rockville, United States: Healthcare Cost and Utilization Project (HCUP), Agency for Healthcare Research and Quality (AHRQ)
Hemorrhagic Stroke	Healthcare Cost and Utilization Project (HCUP), Agency for Healthcare Research and Quality (AHRQ). United States State Inpatient Databases 2013. Rockville, United States: Healthcare Cost and Utilization Project (HCUP), Agency for Healthcare Research and Quality (AHRQ)
Hemorrhagic Stroke	Healthcare Cost and Utilization Project (HCUP), Agency for Healthcare Research and Quality (AHRQ). United States State Inpatient Databases 2012. Rockville, United States: Healthcare Cost and Utilization Project (HCUP), Agency for Healthcare Research and Quality (AHRQ)
Hemorrhagic Stroke	Healthcare Cost and Utilization Project (HCUP), Agency for Healthcare Research and Quality (AHRQ). United States State Inpatient Databases 2014. Rockville, United States: Healthcare Cost and Utilization Project (HCUP), Agency for Healthcare Research and Quality (AHRQ)
Hemorrhagic Stroke	Healthcare Cost and Utilization Project (HCUP), Agency for Healthcare Research and Quality (AHRQ). United States State Inpatient Databases 2006. Rockville, United States: Healthcare Cost and Utilization Project (HCUP), Agency for Healthcare Research and Quality (AHRQ)
Hemorrhagic Stroke	Healthcare Cost and Utilization Project (HCUP), Agency for Healthcare Research and Quality (AHRQ). United States State Inpatient Databases 2009. Rockville, United States: Healthcare Cost and Utilization Project (HCUP), Agency for Healthcare Research and Quality (AHRQ)
Hemorrhagic Stroke	Healthcare Cost and Utilization Project (HCUP), Agency for Healthcare Research and Quality (AHRQ). United States State Inpatient Databases 2005. Rockville, United States: Healthcare Cost and Utilization Project (HCUP), Agency for Healthcare Research and Quality (AHRQ)
Hemorrhagic Stroke	Healthcare Cost and Utilization Project (HCUP), Agency for Healthcare Research and Quality (AHRQ). United States State Inpatient Databases 2008. Rockville, United States: Healthcare Cost and Utilization Project (HCUP), Agency for Healthcare Research and Quality (AHRQ)
Hemorrhagic Stroke	National Center for Health Statistics (NCHS), Centers for Disease Control and Prevention (CDC), United States Census Bureau. United States National Hospital Discharge Survey 1993. Hyattsville, United States: National Center for Health Statistics (NCHS), Centers for Disease Control and Prevention (CDC)
Hemorrhagic Stroke	National Center for Health Statistics (NCHS), Centers for Disease Control and Prevention (CDC), United States Census Bureau. United States National Hospital Discharge Survey 2009. Hyattsville, United States: National Center for Health Statistics (NCHS), Centers for Disease Control and Prevention (CDC)
Hemorrhagic Stroke	National Center for Health Statistics (NCHS), Centers for Disease Control and Prevention (CDC), US Census Bureau. United States National Health Interview Survey 2000. Hyattsville, United States: National Center for Health Statistics (NCHS), Centers for Disease Control and Prevention (CDC)
Hemorrhagic Stroke	National Center for Health Statistics (NCHS), Centers for Disease Control and Prevention (CDC). United States National Health and Nutrition Examination Survey 2007-2008. Hyattsville, United States: National Center for Health Statistics (NCHS), Centers for Disease Control and Prevention (CDC), 2009
Hemorrhagic Stroke	National Center for Health Statistics (NCHS), Centers for Disease Control and Prevention (CDC), United States Census Bureau. United States National Health Interview Survey 2012. Hyattsville, United States: National Center for Health Statistics (NCHS), Centers for Disease Control and Prevention (CDC), 2013
Hemorrhagic Stroke	Healthcare Cost and Utilization Project (HCUP), Agency for Healthcare Research and Quality (AHRQ). United States State Inpatient Databases 2003. Rockville, United States: Healthcare Cost and Utilization Project (HCUP), Agency for Healthcare Research and Quality (AHRQ)
Hemorrhagic Stroke	Healthcare Cost and Utilization Project (HCUP), Agency for Healthcare Research and Quality (AHRQ). United States Nationwide Inpatient Sample 2015. Rockville, United States: Healthcare Cost and Utilization Project (HCUP), Agency for Healthcare Research and Quality (AHRQ)
Hemorrhagic Stroke	National Center for Health Statistics (NCHS), Centers for Disease Control and Prevention (CDC), United States Census Bureau. United States National Hospital Discharge Survey 2002. Hyattsville, United States: National Center for Health Statistics (NCHS), Centers for Disease Control and Prevention (CDC)
Hemorrhagic Stroke	Healthcare Cost and Utilization Project (HCUP), Agency for Healthcare Research and Quality (AHRQ). United States State Inpatient Databases 2007. Rockville, United States: Healthcare Cost and Utilization Project (HCUP), Agency for Healthcare Research and Quality (AHRQ)
Hemorrhagic Stroke	National Center for Health Statistics (NCHS), Centers for Disease Control and Prevention (CDC), United States Census Bureau. United States National Hospital Discharge Survey 1994. Hyattsville, United States: National Center for Health Statistics (NCHS), Centers for Disease Control and Prevention (CDC)
Hemorrhagic Stroke	Healthcare Cost and Utilization Project (HCUP), Agency for Healthcare Research and Quality (AHRQ). United States Nationwide Inpatient Sample 2013. Rockville, United States: Healthcare Cost and Utilization Project (HCUP), Agency for Healthcare Research and Quality (AHRQ)

© 2018 Global Burden of Cardiovascular Diseases Collaboration. *JAMA Cardiology*.

Hemorrhagic Stroke	National Center for Health Statistics (NCHS), Centers for Disease Control and Prevention (CDC), United States Census Bureau. United States National Hospital Discharge Survey 1999. Hyattsville, United States: National Center for Health Statistics (NCHS), Centers for Disease Control and Prevention (CDC)
Hemorrhagic Stroke	National Center for Health Statistics (NCHS), Centers for Disease Control and Prevention (CDC), United States Census Bureau. United States National Hospital Discharge Survey 1981. Hyattsville, United States: National Center for Health Statistics (NCHS), Centers for Disease Control and Prevention (CDC)
Hemorrhagic Stroke	National Center for Health Statistics (NCHS), Centers for Disease Control and Prevention (CDC). United States National Health Interview Survey 2014. Hyattsville, United States: National Center for Health Statistics (NCHS), Centers for Disease Control and Prevention (CDC), 2015
Hemorrhagic Stroke	Healthcare Cost and Utilization Project (HCUP), Agency for Healthcare Research and Quality (AHRQ). United States State Inpatient Databases 2004. Rockville, United States: Healthcare Cost and Utilization Project (HCUP), Agency for Healthcare Research and Quality (AHRQ)
Hemorrhagic Stroke	National Center for Health Statistics (NCHS), Centers for Disease Control and Prevention (CDC), United States Census Bureau. United States National Hospital Discharge Survey 1979. Hyattsville, United States: National Center for Health Statistics (NCHS), Centers for Disease Control and Prevention (CDC)
Hemorrhagic Stroke	National Center for Health Statistics (NCHS), Centers for Disease Control and Prevention (CDC), United States Census Bureau. United States National Hospital Discharge Survey 1996. Hyattsville, United States: National Center for Health Statistics (NCHS), Centers for Disease Control and Prevention (CDC)
Hemorrhagic Stroke	Healthcare Cost and Utilization Project (HCUP), Agency for Healthcare Research and Quality (AHRQ). United States State Inpatient Databases 2003-2007
Hemorrhagic Stroke	National Center for Health Statistics (NCHS), Centers for Disease Control and Prevention (CDC). United States National Health Interview Survey 2015. Hyattsville, United States: National Center for Health Statistics (NCHS), Centers for Disease Control and Prevention (CDC), 2016
Hemorrhagic Stroke	National Center for Health Statistics (NCHS), Centers for Disease Control and Prevention (CDC), US Census Bureau. United States National Health Interview Survey 2011. Hyattsville, United States: National Center for Health Statistics (NCHS), Centers for Disease Control and Prevention (CDC)
Hemorrhagic Stroke	National Center for Health Statistics (NCHS), Centers for Disease Control and Prevention (CDC), US Census Bureau. United States National Health Interview Survey 2008. Hyattsville, United States: National Center for Health Statistics (NCHS), Centers for Disease Control and Prevention (CDC)
Hemorrhagic Stroke	National Center for Health Statistics (NCHS), Centers for Disease Control and Prevention (CDC), US Census Bureau. United States National Health Interview Survey 2010. Hyattsville, United States: National Center for Health Statistics (NCHS), Centers for Disease Control and Prevention (CDC)
Hemorrhagic Stroke	National Center for Health Statistics (NCHS), Centers for Disease Control and Prevention (CDC), US Census Bureau. United States National Health Interview Survey 2002. Hyattsville, United States: National Center for Health Statistics (NCHS), Centers for Disease Control and Prevention (CDC)
Hemorrhagic Stroke	National Center for Health Statistics (NCHS), Centers for Disease Control and Prevention (CDC), United States Census Bureau. United States National Hospital Discharge Survey 1980. Hyattsville, United States: National Center for Health Statistics (NCHS), Centers for Disease Control and Prevention (CDC)
Hemorrhagic Stroke	National Center for Health Statistics (NCHS), Centers for Disease Control and Prevention (CDC), United States Census Bureau. United States National Hospital Discharge Survey 2000. Hyattsville, United States: National Center for Health Statistics (NCHS), Centers for Disease Control and Prevention (CDC)
Hemorrhagic Stroke	National Center for Health Statistics (NCHS), Centers for Disease Control and Prevention (CDC), US Census Bureau. United States National Health Interview Survey 2004. Hyattsville, United States: National Center for Health Statistics (NCHS), Centers for Disease Control and Prevention (CDC)
Hemorrhagic Stroke	Health Measurement Research Group, University of Wisconsin. United States National Health Measurement Study 2005-2006. Data and Information Sciences Center, University of Wisconsin-Madison [distributor]
Hemorrhagic Stroke	Healthcare Cost and Utilization Project (HCUP), Agency for Healthcare Research and Quality (AHRQ). United States State Inpatient Databases 2008-2009
Hemorrhagic Stroke	National Center for Health Statistics (NCHS), Centers for Disease Control and Prevention (CDC), United States Census Bureau. United States National Hospital Discharge Survey 1997. Hyattsville, United States: National Center for Health Statistics (NCHS), Centers for Disease Control and Prevention (CDC)
Hemorrhagic Stroke	National Center for Health Statistics (NCHS), Centers for Disease Control and Prevention (CDC), United States Census Bureau. United States National Hospital Discharge Survey 1984. Hyattsville, United States: National Center for Health Statistics (NCHS), Centers for Disease Control and Prevention (CDC)
Hemorrhagic Stroke	National Center for Health Statistics (NCHS), Centers for Disease Control and Prevention (CDC). United States National Health and Nutrition Examination Survey 2005-2006. Hyattsville, United States: National Center for Health Statistics (NCHS), Centers for Disease Control and Prevention (CDC), 2007
Hemorrhagic Stroke	National Center for Health Statistics (NCHS), Centers for Disease Control and Prevention (CDC), United States Census Bureau. United States National Hospital Discharge Survey 1987. Hyattsville, United States: National Center for Health Statistics (NCHS), Centers for Disease Control and Prevention (CDC)
Hemorrhagic Stroke	National Center for Health Statistics (NCHS), Centers for Disease Control and Prevention (CDC), United States Census Bureau. United States National Hospital Discharge Survey 2001. Hyattsville, United States: National Center for Health Statistics (NCHS), Centers for Disease Control and Prevention (CDC)
Hemorrhagic Stroke	National Center for Health Statistics (NCHS), Centers for Disease Control and Prevention (CDC). United States National Health and Nutrition Examination Survey 1999-2000. Hyattsville, United States: National Center for Health Statistics (NCHS), Centers for Disease Control and Prevention (CDC)
Hemorrhagic Stroke	Washington State University, World Health Organization (WHO). United States WHO Multi-country Survey Study on Health and Health System Responsiveness 2000-2001. Geneva, Switzerland: World Health Organization (WHO)

© 2018 Global Burden of Cardiovascular Diseases Collaboration. *JAMA Cardiology*.

Hemorrhagic Stroke	National Center for Health Statistics (NCHS), Centers for Disease Control and Prevention (CDC), United States Census Bureau. United States National Hospital Discharge Survey 1995. Hyattsville, United States: National Center for Health Statistics (NCHS), Centers for Disease Control and Prevention (CDC)
Hemorrhagic Stroke	National Center for Health Statistics (NCHS), Centers for Disease Control and Prevention (CDC), United States Census Bureau. United States National Hospital Discharge Survey 1988. Hyattsville, United States: National Center for Health Statistics (NCHS), Centers for Disease Control and Prevention (CDC)
Hemorrhagic Stroke	National Center for Health Statistics (NCHS), Centers for Disease Control and Prevention (CDC), US Census Bureau. United States National Health Interview Survey 2006. Hyattsville, United States: National Center for Health Statistics (NCHS), Centers for Disease Control and Prevention (CDC)
Hemorrhagic Stroke	National Center for Health Statistics (NCHS), Centers for Disease Control and Prevention (CDC), United States Census Bureau. United States National Hospital Discharge Survey 1982. Hyattsville, United States: National Center for Health Statistics (NCHS), Centers for Disease Control and Prevention (CDC)
Hemorrhagic Stroke	National Center for Health Statistics (NCHS), Centers for Disease Control and Prevention (CDC), United States Census Bureau. United States National Hospital Discharge Survey 1990. Hyattsville, United States: National Center for Health Statistics (NCHS), Centers for Disease Control and Prevention (CDC)
Hemorrhagic Stroke	National Center for Health Statistics (NCHS) Centers for Disease Control and Prevention (CDC). United States National Health and Nutrition Examination Survey 2011-2012. Hyattsville, United States: National Center for Health Statistics (NCHS) Centers for Disease Control and Prevention (CDC), 2013
Hemorrhagic Stroke	National Center for Health Statistics (NCHS), Centers for Disease Control and Prevention (CDC), United States Census Bureau. United States National Hospital Discharge Survey 2003. Hyattsville, United States: National Center for Health Statistics (NCHS), Centers for Disease Control and Prevention (CDC)
Hemorrhagic Stroke	National Center for Health Statistics (NCHS), Centers for Disease Control and Prevention (CDC), US Census Bureau. United States National Health Interview Survey 2007. Hyattsville, United States: National Center for Health Statistics (NCHS), Centers for Disease Control and Prevention (CDC)
Hemorrhagic Stroke	National Center for Health Statistics (NCHS), Centers for Disease Control and Prevention (CDC), US Census Bureau. United States National Health Interview Survey 2003. Hyattsville, United States: National Center for Health Statistics (NCHS), Centers for Disease Control and Prevention (CDC)
Hemorrhagic Stroke	National Center for Health Statistics (NCHS), Centers for Disease Control and Prevention (CDC), United States Census Bureau. United States National Hospital Discharge Survey 1991. Hyattsville, United States: National Center for Health Statistics (NCHS), Centers for Disease Control and Prevention (CDC)
Hemorrhagic Stroke	National Center for Health Statistics (NCHS), Centers for Disease Control and Prevention (CDC), US Census Bureau. United States National Health Interview Survey 1997. Hyattsville, United States: National Center for Health Statistics (NCHS), Centers for Disease Control and Prevention (CDC)
Hemorrhagic Stroke	National Center for Health Statistics (NCHS), Centers for Disease Control and Prevention (CDC), United States Census Bureau. United States National Hospital Discharge Survey 2006. Hyattsville, United States: National Center for Health Statistics (NCHS), Centers for Disease Control and Prevention (CDC)
Hemorrhagic Stroke	National Center for Health Statistics (NCHS), Centers for Disease Control and Prevention (CDC), United States Census Bureau. United States National Hospital Discharge Survey 2007. Hyattsville, United States: National Center for Health Statistics (NCHS), Centers for Disease Control and Prevention (CDC)
Hemorrhagic Stroke	National Center for Health Statistics (NCHS), Centers for Disease Control and Prevention (CDC), United States Census Bureau. United States National Hospital Discharge Survey 1992. Hyattsville, United States: National Center for Health Statistics (NCHS), Centers for Disease Control and Prevention (CDC)
Hemorrhagic Stroke	National Center for Health Statistics (NCHS), Centers for Disease Control and Prevention (CDC), US Census Bureau. United States National Health Interview Survey 2001. Hyattsville, United States: National Center for Health Statistics (NCHS), Centers for Disease Control and Prevention (CDC)
Hemorrhagic Stroke	United States Department of Health and Human Services. Centers for Disease Control and Prevention. National Center for Health Statistics. National Health Interview Survey, 1994: Second Supplement on Aging. ICPSR02563-v3. Ann Arbor, MI: Inter-university Consortium for Political and Social Research [distributor], 2007-02-12. http://doi.org/10.3886/ICPSR02563.v3
Hemorrhagic Stroke	National Center for Health Statistics (NCHS), Centers for Disease Control and Prevention (CDC), US Census Bureau. United States National Health Interview Survey 1998. Hyattsville, United States: National Center for Health Statistics (NCHS), Centers for Disease Control and Prevention (CDC)
Hemorrhagic Stroke	Healthcare Cost and Utilization Project (HCUP), Agency for Healthcare Research and Quality (AHRQ). United States Nationwide Inpatient Sample 2014. Rockville, United States: Healthcare Cost and Utilization Project (HCUP), Agency for Healthcare Research and Quality (AHRQ)
Hemorrhagic Stroke	United States Department of Health and Human Services. Centers for Disease Control and Prevention. National Center for Health Statistics. National Health Interview Survey, 1994: Second Longitudinal Study on Aging, Wave 2, 1997. ICPSR03526-v2. Ann Arbor, MI: Inter-university Consortium for Political and Social Research [distributor], 2007-03-01. http://doi.org/10.3886/ICPSR03526.v2
Hemorrhagic Stroke	National Center for Health Statistics (NCHS), Centers for Disease Control and Prevention (CDC), US Census Bureau. United States National Health Interview Survey 1990. Hyattsville, United States: National Center for Health Statistics (NCHS), Centers for Disease Control and Prevention (CDC)
Hemorrhagic Stroke	National Center for Health Statistics (NCHS), Centers for Disease Control and Prevention (CDC), US Census Bureau. United States National Health Interview Survey 1999. Hyattsville, United States: National Center for Health Statistics (NCHS), Centers for Disease Control and Prevention (CDC)
Hemorrhagic Stroke	Truven Health Analytics. United States MarketScan Commercial Claims and Encounters Database 2010. Ann Arbor, United States: Truven Health Analytics
Hemorrhagic Stroke	National Center for Health Statistics (NCHS), Centers for Disease Control and Prevention (CDC). United States National Hospital Discharge Survey 2003-2007

© 2018 Global Burden of Cardiovascular Diseases Collaboration. *JAMA Cardiology*.

Hemorrhagic Stroke	National Center for Health Statistics (NCHS), Centers for Disease Control and Prevention (CDC), United States Census Bureau. United States National Hospital Discharge Survey 1998. Hyattsville, United States: National Center for Health Statistics (NCHS), Centers for Disease Control and Prevention (CDC)
Hemorrhagic Stroke	National Center for Health Statistics (NCHS), Centers for Disease Control and Prevention (CDC), United States Census Bureau. United States National Hospital Discharge Survey 2004. Hyattsville, United States: National Center for Health Statistics (NCHS), Centers for Disease Control and Prevention (CDC)
Hemorrhagic Stroke	National Center for Health Statistics (NCHS), Centers for Disease Control and Prevention (CDC), US Census Bureau. United States National Health Interview Survey 2009. Hyattsville, United States: National Center for Health Statistics (NCHS), Centers for Disease Control and Prevention (CDC)
Hemorrhagic Stroke	National Center for Health Statistics (NCHS), Centers for Disease Control and Prevention (CDC), United States Census Bureau. United States National Hospital Discharge Survey 1985. Hyattsville, United States: National Center for Health Statistics (NCHS), Centers for Disease Control and Prevention (CDC)
Hemorrhagic Stroke	Health and Retirement Study, (Biennial 2000) public use dataset. Produced and distributed by the University of Michigan with funding from the National Institute on Aging (grant number NIA U01AG009740). Ann Arbor, MI, (2011)
Hemorrhagic Stroke	National Center for Health Statistics (NCHS), Centers for Disease Control and Prevention (CDC). United States National Health and Nutrition Examination Survey 2001-2002. Hyattsville, United States: National Center for Health Statistics (NCHS), Centers for Disease Control and Prevention (CDC)
Hemorrhagic Stroke	National Center for Health Statistics (NCHS), Centers for Disease Control and Prevention (CDC), United States Census Bureau. United States National Hospital Discharge Survey 2010. Hyattsville, United States: National Center for Health Statistics (NCHS), Centers for Disease Control and Prevention (CDC)
Hemorrhagic Stroke	Panel Study of Income Dynamics, 2005 public use dataset. Produced and distributed by the University of Michigan with primary funding from the National Science Foundation, the National Institute of Aging, and the National Institute of Child Health and Human Development. Ann Arbor, MI, (2011)
Hemorrhagic Stroke	Panel Study of Income Dynamics, 2007 public use dataset. Produced and distributed by the University of Michigan with primary funding from the National Science Foundation, the National Institute of Aging, and the National Institute of Child Health and Human Development. Ann Arbor, MI, (2011)
Hemorrhagic Stroke	National Center for Health Statistics (NCHS), Centers for Disease Control and Prevention (CDC). United States National Health and Nutrition Examination Survey 1988-1994. Hyattsville, United States: National Center for Health Statistics (NCHS), Centers for Disease Control and Prevention (CDC)
Hemorrhagic Stroke	National Center for Health Statistics (NCHS), Centers for Disease Control and Prevention (CDC). United States National Health and Nutrition Examination Survey 2009-2010. Hyattsville, United States: National Center for Health Statistics (NCHS), Centers for Disease Control and Prevention (CDC), 2011
Hemorrhagic Stroke	Health and Retirement Study, (Biennial 2006) public use dataset. Produced and distributed by the University of Michigan with funding from the National Institute on Aging (grant number NIA U01AG009740). Ann Arbor, MI, (2014)
Hemorrhagic Stroke	National Center for Health Statistics (NCHS), Centers for Disease Control and Prevention (CDC). United States National Hospital Discharge Survey 1988-1992
Hemorrhagic Stroke	National Center for Health Statistics (NCHS), Centers for Disease Control and Prevention (CDC). United States National Hospital Discharge Survey 1993-1997
Hemorrhagic Stroke	National Center for Health Statistics (NCHS), Centers for Disease Control and Prevention (CDC). United States National Hospital Discharge Survey 1998-2002
Hemorrhagic Stroke	National Center for Health Statistics (NCHS), Centers for Disease Control and Prevention (CDC), United States Census Bureau. United States National Hospital Discharge Survey 1983. Hyattsville, United States: National Center for Health Statistics (NCHS), Centers for Disease Control and Prevention (CDC)
Hemorrhagic Stroke	National Center for Health Statistics (NCHS), Centers for Disease Control and Prevention (CDC). United States National Hospital Discharge Survey 2008-2010
Hemorrhagic Stroke	National Center for Health Statistics (NCHS), Centers for Disease Control and Prevention (CDC), United States Census Bureau. United States National Hospital Discharge Survey 1989. Hyattsville, United States: National Center for Health Statistics (NCHS), Centers for Disease Control and Prevention (CDC)
Hemorrhagic Stroke	National Center for Health Statistics (NCHS), Centers for Disease Control and Prevention (CDC), United States Census Bureau. United States National Hospital Discharge Survey 2008. Hyattsville, United States: National Center for Health Statistics (NCHS), Centers for Disease Control and Prevention (CDC)
Hemorrhagic Stroke	National Center for Health Statistics (NCHS), Centers for Disease Control and Prevention (CDC), United States Census Bureau. United States National Hospital Discharge Survey 1986. Hyattsville, United States: National Center for Health Statistics (NCHS), Centers for Disease Control and Prevention (CDC)
Hemorrhagic Stroke	National Center for Health Statistics (NCHS), Centers for Disease Control and Prevention (CDC). United States National Health and Nutrition Examination Survey 2013-2014. Hyattsville, United States: National Center for Health Statistics (NCHS), Centers for Disease Control and Prevention (CDC)
Hemorrhagic Stroke	Health and Retirement Study, (Biennial 2010) public use dataset. Produced and distributed by the University of Michigan with funding from the National Institute on Aging (grant number NIA U01AG009740). Ann Arbor, MI, (2014)
Hemorrhagic Stroke	National Center for Health Statistics (NCHS), Centers for Disease Control and Prevention (CDC). United States National Health and Nutrition Examination Survey 2003-2004. Hyattsville, United States: National Center for Health Statistics (NCHS), Centers for Disease Control and Prevention (CDC)
Hemorrhagic Stroke	National Center for Health Statistics (NCHS), Centers for Disease Control and Prevention (CDC), US Census Bureau. United States National Health Interview Survey 1991. Hyattsville, United States: National Center for Health Statistics (NCHS), Centers for Disease Control and Prevention (CDC)

© 2018 Global Burden of Cardiovascular Diseases Collaboration. *JAMA Cardiology*.

Hemorrhagic Stroke	National Center for Health Statistics (NCHS), Centers for Disease Control and Prevention (CDC), United States Census Bureau. United States National Health Interview Survey 2013. Hyattsville, United States: National Center for Health Statistics (NCHS), Centers for Disease Control and Prevention (CDC), 2014
Hemorrhagic Stroke	Health and Retirement Study, (Biennial 1998) public use dataset. Produced and distributed by the University of Michigan with funding from the National Institute on Aging (grant number NIA U01AG009740). Ann Arbor, MI, (2014)
Hemorrhagic Stroke	National Center for Health Statistics (NCHS), Centers for Disease Control and Prevention (CDC), United States Census Bureau. United States National Hospital Discharge Survey 2005. Hyattsville, United States: National Center for Health Statistics (NCHS), Centers for Disease Control and Prevention (CDC)
Hemorrhagic Stroke	Health and Retirement Study, (Biennial 2002) public use dataset. Produced and distributed by the University of Michigan with funding from the National Institute on Aging (grant number NIA U01AG009740). Ann Arbor, MI, (2011)
Hemorrhagic Stroke	Health and Retirement Study, (Biennial 2004) public use dataset. Produced and distributed by the University of Michigan with funding from the National Institute on Aging (grant number NIA U01AG009740). Ann Arbor, MI, (2013)
Hemorrhagic Stroke	National Center for Health Statistics (NCHS), Centers for Disease Control and Prevention (CDC), US Census Bureau. United States National Health Interview Survey 1992. Hyattsville, United States: National Center for Health Statistics (NCHS), Centers for Disease Control and Prevention (CDC)
Hemorrhagic Stroke	Health and Retirement Study, (Biennial 2012) public use dataset. Produced and distributed by the University of Michigan with funding from the National Institute on Aging (grant number NIA U01AG009740). Ann Arbor, MI, (2015)
Hemorrhagic Stroke	National Center for Health Statistics (NCHS), Centers for Disease Control and Prevention (CDC), US Census Bureau. United States National Health Interview Survey 1993. Hyattville, United States: National Center for Health Statistics (NCHS), Centers for Disease Control and Prevention (CDC)
Hemorrhagic Stroke	National Center for Health Statistics (NCHS), Centers for Disease Control and Prevention (CDC), US Census Bureau. United States National Health Interview Survey 2005. Hyattsville, United States: National Center for Health Statistics (NCHS), Centers for Disease Control and Prevention (CDC)
Hemorrhagic Stroke	Agency for Healthcare Research and Quality. United States Medical Expenditure Panel Survey 2002-2009. Rockville, United States: Agency for Healthcare Research and Quality
Hemorrhagic Stroke	Health and Retirement Study, (Biennial 1992) public use dataset. Produced and distributed by the University of Michigan with funding from the National Institute on Aging (grant number NIA U01AG009740). Ann Arbor, MI, (2011)
Hemorrhagic Stroke	Health and Retirement Study, (Biennial 1996) public use dataset. Produced and distributed by the University of Michigan with funding from the National Institute on Aging (grant number NIA U01AG009740). Ann Arbor, MI, (2011)
Hemorrhagic Stroke	Howard G, Cushman M, Howard VJ, Kissela BM, Kleindorfer DO, Moy CS, Switzer J, Woo D. Risk factors for Hemorrhagic Stroke: the REasons for geographic and racial differences in stroke (REGARDS) study. Stroke. 2013; 44(5): 1282Â–7
Hemorrhagic Stroke	Zahuranec DB, Brown DL, Lisabeth LD, Morgenstern LB. Is it time for a large, collaborative study of pediatric stroke?. Stroke. 2005; 36(9): 1825-9
Hemorrhagic Stroke	Earley CJ, Kittner SJ, Feeser BR, Gardner J, Epstein A, Wozniak MA, Wityk R, Stern BJ, Price TR, Macko RF, Johnson C, Sloan MA, Buchholz D. Stroke in children and sickle-cell disease: Baltimore-Washington Cooperative Young Stroke Study. Neurology. 1998; 51(1): 169-76
Ischemic heart disease	Healthcare Cost and Utilization Project (HCUP), Agency for Healthcare Research and Quality (AHRQ). United States State Inpatient Databases 2013. Rockville, United States: Healthcare Cost and Utilization Project (HCUP), Agency for Healthcare Research and Quality (AHRQ)
Ischemic heart disease	Healthcare Cost and Utilization Project (HCUP), Agency for Healthcare Research and Quality (AHRQ). United States State Inpatient Databases 2014. Rockville, United States: Healthcare Cost and Utilization Project (HCUP), Agency for Healthcare Research and Quality (AHRQ)
Ischemic heart disease	National Center for Health Statistics (NCHS), Centers for Disease Control and Prevention (CDC), United States Census Bureau. United States National Hospital Discharge Survey 1996. Hyattsville, United States: National Center for Health Statistics (NCHS), Centers for Disease Control and Prevention (CDC)
Ischemic heart disease	Healthcare Cost and Utilization Project (HCUP), Agency for Healthcare Research and Quality (AHRQ). United States State Inpatient Databases 2011. Rockville, United States: Healthcare Cost and Utilization Project (HCUP), Agency for Healthcare Research and Quality (AHRQ)
Ischemic heart disease	Healthcare Cost and Utilization Project (HCUP), Agency for Healthcare Research and Quality (AHRQ). United States State Inpatient Databases 2012. Rockville, United States: Healthcare Cost and Utilization Project (HCUP), Agency for Healthcare Research and Quality (AHRQ)
Ischemic heart disease	Truven Health Analytics. United States MarketScan Claims and Medicare Data - 2010. Ann Arbor, United States: Truven Health Analytics
Ischemic heart disease	Healthcare Cost and Utilization Project (HCUP), Agency for Healthcare Research and Quality (AHRQ). United States State Inpatient Databases 2009. Rockville, United States: Healthcare Cost and Utilization Project (HCUP), Agency for Healthcare Research and Quality (AHRQ)
Ischemic heart disease	Healthcare Cost and Utilization Project (HCUP), Agency for Healthcare Research and Quality (AHRQ). United States State Inpatient Databases 2006. Rockville, United States: Healthcare Cost and Utilization Project (HCUP), Agency for Healthcare Research and Quality (AHRQ)
Ischemic heart disease	Healthcare Cost and Utilization Project (HCUP), Agency for Healthcare Research and Quality (AHRQ). United States State Inpatient Databases 2008. Rockville, United States: Healthcare Cost and Utilization Project (HCUP), Agency for Healthcare Research and Quality (AHRQ)

© 2018 Global Burden of Cardiovascular Diseases Collaboration. *JAMA Cardiology*.

Ischemic heart disease	National Center for Health Statistics (NCHS), Centers for Disease Control and Prevention (CDC), United States Census Bureau. United States National Health Interview Survey 2012. Hyattsville, United States: National Center for Health Statistics (NCHS), Centers for Disease Control and Prevention (CDC), 2013
Ischemic heart disease	National Center for Health Statistics (NCHS), Centers for Disease Control and Prevention (CDC), US Census Bureau. United States National Health Interview Survey 2002. Hyattsville, United States: National Center for Health Statistics (NCHS), Centers for Disease Control and Prevention (CDC)
Ischemic heart disease	Panel Study of Income Dynamics, 2007 public use dataset. Produced and distributed by the University of Michigan with primary funding from the National Science Foundation, the National Institute of Aging, and the National Institute of Child Health and Human Development. Ann Arbor, MI, (2011)
Ischemic heart disease	National Center for Health Statistics (NCHS), Centers for Disease Control and Prevention (CDC), US Census Bureau. United States National Health Interview Survey 1992. Hyattsville, United States: National Center for Health Statistics (NCHS), Centers for Disease Control and Prevention (CDC)
Ischemic heart disease	Healthcare Cost and Utilization Project (HCUP), Agency for Healthcare Research and Quality (AHRQ). United States State Inpatient Databases 2004. Rockville, United States: Healthcare Cost and Utilization Project (HCUP), Agency for Healthcare Research and Quality (AHRQ)
Ischemic heart disease	Healthcare Cost and Utilization Project (HCUP), Agency for Healthcare Research and Quality (AHRQ). United States State Inpatient Databases 2005. Rockville, United States: Healthcare Cost and Utilization Project (HCUP), Agency for Healthcare Research and Quality (AHRQ)
Ischemic heart disease	National Center for Health Statistics (NCHS), Centers for Disease Control and Prevention (CDC), US Census Bureau. United States National Health Interview Survey 2000. Hyattsville, United States: National Center for Health Statistics (NCHS), Centers for Disease Control and Prevention (CDC)
Ischemic heart disease	Truven Health Analytics. United States MarketScan Claims and Medicare Data - 2012. Ann Arbor, United States: Truven Health Analytics
Ischemic heart disease	Healthcare Cost and Utilization Project (HCUP), Agency for Healthcare Research and Quality (AHRQ). United States State Inpatient Databases 2003. Rockville, United States: Healthcare Cost and Utilization Project (HCUP), Agency for Healthcare Research and Quality (AHRQ)
Ischemic heart disease	National Center for Health Statistics (NCHS) Centers for Disease Control and Prevention (CDC). United States National Health and Nutrition Examination Survey 2011-2012. Hyattsville, United States: National Center for Health Statistics (NCHS) Centers for Disease Control and Prevention (CDC), 2013
Ischemic heart disease	National Center for Health Statistics (NCHS), Centers for Disease Control and Prevention (CDC). United States National Health and Nutrition Examination Survey 2009-2010. Hyattsville, United States: National Center for Health Statistics (NCHS), Centers for Disease Control and Prevention (CDC), 2011
Ischemic heart disease	Healthcare Cost and Utilization Project (HCUP), Agency for Healthcare Research and Quality (AHRQ). United States State Inpatient Databases 2007. Rockville, United States: Healthcare Cost and Utilization Project (HCUP), Agency for Healthcare Research and Quality (AHRQ)
Ischemic heart disease	Truven Health Analytics. United States MarketScan Claims and Medicare Data - 2000. Ann Arbor, United States: Truven Health Analytics
Ischemic heart disease	National Center for Health Statistics (NCHS), Centers for Disease Control and Prevention (CDC), United States Census Bureau. United States National Hospital Discharge Survey 1990. Hyattsville, United States: National Center for Health Statistics (NCHS), Centers for Disease Control and Prevention (CDC)
Ischemic heart disease	National Center for Health Statistics (NCHS), Centers for Disease Control and Prevention (CDC), US Census Bureau. United States National Health Interview Survey 1999. Hyattsville, United States: National Center for Health Statistics (NCHS), Centers for Disease Control and Prevention (CDC)
Ischemic heart disease	National Center for Health Statistics (NCHS), Centers for Disease Control and Prevention (CDC), United States Census Bureau. United States National Hospital Discharge Survey 1987. Hyattsville, United States: National Center for Health Statistics (NCHS), Centers for Disease Control and Prevention (CDC)
Ischemic heart disease	National Center for Health Statistics (NCHS), Centers for Disease Control and Prevention (CDC), United States Census Bureau. United States National Hospital Discharge Survey 1981. Hyattsville, United States: National Center for Health Statistics (NCHS), Centers for Disease Control and Prevention (CDC)
Ischemic heart disease	Healthcare Cost and Utilization Project (HCUP), Agency for Healthcare Research and Quality (AHRQ). United States State Inpatient Databases 2003-2007
Ischemic heart disease	National Center for Health Statistics (NCHS), Centers for Disease Control and Prevention (CDC), US Census Bureau. United States National Health Interview Survey 2008. Hyattsville, United States: National Center for Health Statistics (NCHS), Centers for Disease Control and Prevention (CDC)
Ischemic heart disease	National Center for Health Statistics (NCHS), Centers for Disease Control and Prevention (CDC), US Census Bureau. United States National Health Interview Survey 2010. Hyattsville, United States: National Center for Health Statistics (NCHS), Centers for Disease Control and Prevention (CDC)
Ischemic heart disease	National Center for Health Statistics (NCHS), Centers for Disease Control and Prevention (CDC), United States Census Bureau. United States National Hospital Discharge Survey 2002. Hyattsville, United States: National Center for Health Statistics (NCHS), Centers for Disease Control and Prevention (CDC)
Ischemic heart disease	National Center for Health Statistics (NCHS), Centers for Disease Control and Prevention (CDC), United States Census Bureau. United States National Hospital Discharge Survey 1994. Hyattsville, United States: National Center for Health Statistics (NCHS), Centers for Disease Control and Prevention (CDC)
Ischemic heart disease	National Center for Health Statistics (NCHS), Centers for Disease Control and Prevention (CDC), United States Census Bureau. United States National Hospital Discharge Survey 1989. Hyattsville, United States: National Center for Health Statistics (NCHS), Centers for Disease Control and Prevention (CDC)
Ischemic heart disease	Panel Study of Income Dynamics, 2005 public use dataset. Produced and distributed by the University of Michigan with primary funding from the National Science Foundation, the National Institute of Aging, and the National Institute of Child Health and Human Development. Ann Arbor, MI, (2011)

© 2018 Global Burden of Cardiovascular Diseases Collaboration. *JAMA Cardiology*.

Ischemic heart disease	National Center for Health Statistics (NCHS), Centers for Disease Control and Prevention (CDC), United States Census Bureau. United States National Hospital Discharge Survey 2004. Hyattsville, United States: National Center for Health Statistics (NCHS), Centers for Disease Control and Prevention (CDC)
Ischemic heart disease	National Center for Health Statistics (NCHS), Centers for Disease Control and Prevention (CDC), United States Census Bureau. United States National Hospital Discharge Survey 2007. Hyattsville, United States: National Center for Health Statistics (NCHS), Centers for Disease Control and Prevention (CDC)
Ischemic heart disease	National Center for Health Statistics (NCHS), Centers for Disease Control and Prevention (CDC), United States Census Bureau. United States National Hospital Discharge Survey 2006. Hyattsville, United States: National Center for Health Statistics (NCHS), Centers for Disease Control and Prevention (CDC)
Ischemic heart disease	National Center for Health Statistics (NCHS), Centers for Disease Control and Prevention (CDC), United States Census Bureau. United States National Hospital Discharge Survey 2000. Hyattsville, United States: National Center for Health Statistics (NCHS), Centers for Disease Control and Prevention (CDC)
Ischemic heart disease	National Center for Health Statistics (NCHS), Centers for Disease Control and Prevention (CDC). United States National Health Interview Survey 2014. Hyattsville, United States: National Center for Health Statistics (NCHS), Centers for Disease Control and Prevention (CDC), 2015
Ischemic heart disease	National Center for Health Statistics (NCHS), Centers for Disease Control and Prevention (CDC), United States Census Bureau. United States National Hospital Discharge Survey 1993. Hyattsville, United States: National Center for Health Statistics (NCHS), Centers for Disease Control and Prevention (CDC)
Ischemic heart disease	National Center for Health Statistics (NCHS), Centers for Disease Control and Prevention (CDC), US Census Bureau. United States National Health Interview Survey 2004. Hyattsville, United States: National Center for Health Statistics (NCHS), Centers for Disease Control and Prevention (CDC)
Ischemic heart disease	National Center for Health Statistics (NCHS), Centers for Disease Control and Prevention (CDC). United States National Hospital Discharge Survey 1998-2002
Ischemic heart disease	National Center for Health Statistics (NCHS), Centers for Disease Control and Prevention (CDC), United States Census Bureau. United States National Hospital Discharge Survey 1997. Hyattsville, United States: National Center for Health Statistics (NCHS), Centers for Disease Control and Prevention (CDC)
Ischemic heart disease	National Center for Health Statistics (NCHS), Centers for Disease Control and Prevention (CDC), United States Census Bureau. United States National Hospital Discharge Survey 1992. Hyattsville, United States: National Center for Health Statistics (NCHS), Centers for Disease Control and Prevention (CDC)
Ischemic heart disease	National Center for Health Statistics (NCHS), Centers for Disease Control and Prevention (CDC), United States Census Bureau. United States National Hospital Discharge Survey 2008. Hyattsville, United States: National Center for Health Statistics (NCHS), Centers for Disease Control and Prevention (CDC)
Ischemic heart disease	National Center for Health Statistics (NCHS), Centers for Disease Control and Prevention (CDC). United States National Health and Nutrition Examination Survey 2005-2006. Hyattsville, United States: National Center for Health Statistics (NCHS), Centers for Disease Control and Prevention (CDC), 2007
Ischemic heart disease	National Center for Health Statistics (NCHS), Centers for Disease Control and Prevention (CDC). United States National Health and Nutrition Examination Survey 1988-1994. Hyattsville, United States: National Center for Health Statistics (NCHS), Centers for Disease Control and Prevention (CDC)
Ischemic heart disease	National Center for Health Statistics (NCHS), Centers for Disease Control and Prevention (CDC). United States National Health and Nutrition Examination Survey 1999-2000. Hyattsville, United States: National Center for Health Statistics (NCHS), Centers for Disease Control and Prevention (CDC)
Ischemic heart disease	National Center for Health Statistics (NCHS), Centers for Disease Control and Prevention (CDC), US Census Bureau. United States National Health Interview Survey 2011. Hyattsville, United States: National Center for Health Statistics (NCHS), Centers for Disease Control and Prevention (CDC)
Ischemic heart disease	Washington State University, World Health Organization (WHO). United States WHO Multi-country Survey Study on Health and Health System Responsiveness 2000-2001. Geneva, Switzerland: World Health Organization (WHO)
Ischemic heart disease	National Center for Health Statistics (NCHS), Centers for Disease Control and Prevention (CDC), United States Census Bureau. United States National Hospital Discharge Survey 1988. Hyattsville, United States: National Center for Health Statistics (NCHS), Centers for Disease Control and Prevention (CDC)
Ischemic heart disease	National Center for Health Statistics (NCHS), Centers for Disease Control and Prevention (CDC), United States Census Bureau. United States National Hospital Discharge Survey 1983. Hyattsville, United States: National Center for Health Statistics (NCHS), Centers for Disease Control and Prevention (CDC)
Ischemic heart disease	National Center for Health Statistics (NCHS), Centers for Disease Control and Prevention (CDC), United States Census Bureau. United States National Hospital Discharge Survey 1995. Hyattsville, United States: National Center for Health Statistics (NCHS), Centers for Disease Control and Prevention (CDC)
Ischemic heart disease	National Center for Health Statistics (NCHS), Centers for Disease Control and Prevention (CDC), US Census Bureau. United States National Health Interview Survey 2001. Hyattsville, United States: National Center for Health Statistics (NCHS), Centers for Disease Control and Prevention (CDC)
Ischemic heart disease	National Center for Health Statistics (NCHS), Centers for Disease Control and Prevention (CDC), US Census Bureau. United States National Health Interview Survey 2006. Hyattsville, United States: National Center for Health Statistics (NCHS), Centers for Disease Control and Prevention (CDC)
Ischemic heart disease	National Center for Health Statistics (NCHS), Centers for Disease Control and Prevention (CDC), United States Census Bureau. United States National Hospital Discharge Survey 1982. Hyattsville, United States: National Center for Health Statistics (NCHS), Centers for Disease Control and Prevention (CDC)
Ischemic heart disease	National Center for Health Statistics (NCHS), Centers for Disease Control and Prevention (CDC), United States Census Bureau. United States National Hospital Discharge Survey 2009. Hyattsville, United States: National Center for Health Statistics (NCHS), Centers for Disease Control and Prevention (CDC)

© 2018 Global Burden of Cardiovascular Diseases Collaboration. *JAMA Cardiology*.

Ischemic heart disease	Healthcare Cost and Utilization Project (HCUP), Agency for Healthcare Research and Quality (AHRQ). United States Nationwide Inpatient Sample 2013. Rockville, United States: Healthcare Cost and Utilization Project (HCUP), Agency for Healthcare Research and Quality (AHRQ)
Ischemic heart disease	National Center for Health Statistics (NCHS), Centers for Disease Control and Prevention (CDC), United States Census Bureau. United States National Hospital Discharge Survey 1979. Hyattsville, United States: National Center for Health Statistics (NCHS), Centers for Disease Control and Prevention (CDC)
Ischemic heart disease	National Center for Health Statistics (NCHS), Centers for Disease Control and Prevention (CDC), US Census Bureau. United States National Health Interview Survey 1993. Hyattville, United States: National Center for Health Statistics (NCHS), Centers for Disease Control and Prevention (CDC)
Ischemic heart disease	National Center for Health Statistics (NCHS), Centers for Disease Control and Prevention (CDC), United States Census Bureau. United States National Hospital Discharge Survey 1998. Hyattsville, United States: National Center for Health Statistics (NCHS), Centers for Disease Control and Prevention (CDC)
Ischemic heart disease	National Center for Health Statistics (NCHS), Centers for Disease Control and Prevention (CDC), United States Census Bureau. United States National Hospital Discharge Survey 1999. Hyattsville, United States: National Center for Health Statistics (NCHS), Centers for Disease Control and Prevention (CDC)
Ischemic heart disease	National Center for Health Statistics (NCHS), Centers for Disease Control and Prevention (CDC), United States Census Bureau. United States National Hospital Discharge Survey 1986. Hyattsville, United States: National Center for Health Statistics (NCHS), Centers for Disease Control and Prevention (CDC)
Ischemic heart disease	National Center for Health Statistics (NCHS), Centers for Disease Control and Prevention (CDC). United States National Health and Nutrition Examination Survey 2001-2002. Hyattsville, United States: National Center for Health Statistics (NCHS), Centers for Disease Control and Prevention (CDC)
Ischemic heart disease	National Center for Health Statistics (NCHS), Centers for Disease Control and Prevention (CDC), US Census Bureau. United States National Health Interview Survey 1998. Hyattsville, United States: National Center for Health Statistics (NCHS), Centers for Disease Control and Prevention (CDC)
Ischemic heart disease	National Center for Health Statistics (NCHS), Centers for Disease Control and Prevention (CDC), United States Census Bureau. United States National Hospital Discharge Survey 2001. Hyattsville, United States: National Center for Health Statistics (NCHS), Centers for Disease Control and Prevention (CDC)
Ischemic heart disease	National Center for Health Statistics (NCHS), Centers for Disease Control and Prevention (CDC), US Census Bureau. United States National Health Interview Survey 1991. Hyattsville, United States: National Center for Health Statistics (NCHS), Centers for Disease Control and Prevention (CDC)
Ischemic heart disease	National Center for Health Statistics (NCHS), Centers for Disease Control and Prevention (CDC), United States Census Bureau. United States National Hospital Discharge Survey 2003. Hyattsville, United States: National Center for Health Statistics (NCHS), Centers for Disease Control and Prevention (CDC)
Ischemic heart disease	National Center for Health Statistics (NCHS), Centers for Disease Control and Prevention (CDC). United States National Health and Nutrition Examination Survey 2007-2008. Hyattsville, United States: National Center for Health Statistics (NCHS), Centers for Disease Control and Prevention (CDC), 2009
Ischemic heart disease	National Center for Health Statistics (NCHS), Centers for Disease Control and Prevention (CDC). United States National Hospital Discharge Survey 2003-2007
Ischemic heart disease	National Center for Health Statistics (NCHS), Centers for Disease Control and Prevention (CDC), United States Census Bureau. United States National Hospital Discharge Survey 1985. Hyattsville, United States: National Center for Health Statistics (NCHS), Centers for Disease Control and Prevention (CDC)
Ischemic heart disease	Healthcare Cost and Utilization Project (HCUP), Agency for Healthcare Research and Quality (AHRQ). United States Nationwide Inpatient Sample 2014. Rockville, United States: Healthcare Cost and Utilization Project (HCUP), Agency for Healthcare Research and Quality (AHRQ)
Ischemic heart disease	Harvard University Medical School. United States National Comorbidity Survey: Replication 12-Month Prevalence Estimates 2001-2003. Boston, United States: Harvard University Medical School
Ischemic heart disease	United States Department of Health and Human Services. Centers for Disease Control and Prevention. National Center for Health Statistics. National Health Interview Survey, 1994: Second Longitudinal Study on Aging, Wave 2, 1997. ICPSR03526-v2. Ann Arbor, MI: Inter-university Consortium for Political and Social Research [distributor], 2007-03-01. http://doi.org/10.3886/ICPSR03526.v2
Ischemic heart disease	Healthcare Cost and Utilization Project (HCUP), Agency for Healthcare Research and Quality (AHRQ). United States State Inpatient Databases 2008-2009
Ischemic heart disease	National Center for Health Statistics (NCHS), Centers for Disease Control and Prevention (CDC). United States National Hospital Discharge Survey 1988-1992
Ischemic heart disease	National Center for Health Statistics (NCHS), Centers for Disease Control and Prevention (CDC). United States National Hospital Discharge Survey 1993-1997
Ischemic heart disease	National Center for Health Statistics (NCHS), Centers for Disease Control and Prevention (CDC), US Census Bureau. United States National Health Interview Survey 1990. Hyattsville, United States: National Center for Health Statistics (NCHS), Centers for Disease Control and Prevention (CDC)
Ischemic heart disease	National Center for Health Statistics (NCHS), Centers for Disease Control and Prevention (CDC). United States National Hospital Discharge Survey 2008-2010
Ischemic heart disease	National Center for Health Statistics (NCHS), Centers for Disease Control and Prevention (CDC). United States National Health and Nutrition Examination Survey 2003-2004. Hyattsville, United States: National Center for Health Statistics (NCHS), Centers for Disease Control and Prevention (CDC)
Ischemic heart disease	National Center for Health Statistics (NCHS), Centers for Disease Control and Prevention (CDC), United States Census Bureau. United States National Hospital Discharge Survey 1980. Hyattsville, United States: National Center for Health Statistics (NCHS), Centers for Disease Control and Prevention (CDC)

© 2018 Global Burden of Cardiovascular Diseases Collaboration. *JAMA Cardiology*.

Ischemic heart disease	National Center for Health Statistics (NCHS), Centers for Disease Control and Prevention (CDC), US Census Bureau. United States National Health Interview Survey 2009. Hyattsville, United States: National Center for Health Statistics (NCHS), Centers for Disease Control and Prevention (CDC)
Ischemic heart disease	National Center for Health Statistics (NCHS), Centers for Disease Control and Prevention (CDC), United States Census Bureau. United States National Hospital Discharge Survey 1991. Hyattsville, United States: National Center for Health Statistics (NCHS), Centers for Disease Control and Prevention (CDC)
Ischemic heart disease	Healthcare Cost and Utilization Project (HCUP), Agency for Healthcare Research and Quality (AHRQ). United States Nationwide Inpatient Sample 2015. Rockville, United States: Healthcare Cost and Utilization Project (HCUP), Agency for Healthcare Research and Quality (AHRQ)
Ischemic heart disease	National Center for Health Statistics (NCHS), Centers for Disease Control and Prevention (CDC), US Census Bureau. United States National Health Interview Survey 2007. Hyattsville, United States: National Center for Health Statistics (NCHS), Centers for Disease Control and Prevention (CDC)
Ischemic heart disease	Agency for Healthcare Research and Quality. United States Medical Expenditure Panel Survey 2002-2009. Rockville, United States: Agency for Healthcare Research and Quality
Ischemic heart disease	Ford ES, Giles WH, Croft JB. Prevalence of nonfatal coronary heart disease among American adults. Am Heart J. 2000; 139(3): 371-7
Ischemic heart disease	Mozaffarian D, Bryson CL, Spertus JA, McDonell MB, Fihn SD. Anginal symptoms consistently predict total mortality among outpatients with coronary artery disease. Am Heart J. 2003; 146(6): 1015-22
Ischemic heart disease	Shao Y-H, Croitor SK, Moreyra AE, Wilson AC, Kostis WJ, Cosgrove NM, Kostis JB, MIDAS Study Group (MIDAS II). Comparison of hospital versus out of hospital coronary death rates in women and men. Am J Cardiol. 2010; 106(1): 26Â–30
Ischemic heart disease	OÂ’Brien EC, Rose KM, Suchindran CM, StÃ¼rmer T, Chang PP, Chambless L, Guild CS, Rosamond WD. Medication, reperfusion therapy and survival in a community-based setting of hospitalised myocardial infarction. Heart. 2013; 99(11): 767Â–73
Ischemic heart disease	Nguyen HL, Saczynski JS, Gore JM, Waring ME, Lessard D, Yarzebski J, Reed G, Spencer FA, Li S, Goldberg RJ. Long-term trends in short-term outcomes in acute myocardial infarction. Am J Med. 2011; 124(10): 939Â–46
Ischemic heart disease	Ergin A, Muntner P, Sherwin R, He J. Secular trends in cardiovascular disease mortality, incidence, and case fatality rates in adults in the United States. Am J Med. 2004; 117(4): 219-27
Ischemic heart disease	McGovern PG, Jacobs DR, Shahar E, Arnett DK, Folsom AR, Blackburn H, Luepker RV. Trends in Acute Coronary Heart Disease Mortality, Morbidity, and Medical Care From 1985 Through 1997?: The Minnesota Heart Survey. Circulation. 2001; 104(1): 19-24
Ischemic heart disease	National Center for Health Statistics (NCHS), Centers for Disease Control and Prevention (CDC), US Census Bureau. United States National Health Interview Survey 2003. Hyattsville, United States: National Center for Health Statistics (NCHS), Centers for Disease Control and Prevention (CDC)
Ischemic heart disease	National Center for Health Statistics (NCHS), Centers for Disease Control and Prevention (CDC), United States Census Bureau. United States National Hospital Discharge Survey 2010. Hyattsville, United States: National Center for Health Statistics (NCHS), Centers for Disease Control and Prevention (CDC)
Ischemic heart disease	National Center for Health Statistics (NCHS), Centers for Disease Control and Prevention (CDC), United States Census Bureau. United States National Hospital Discharge Survey 2005. Hyattsville, United States: National Center for Health Statistics (NCHS), Centers for Disease Control and Prevention (CDC)
Ischemic heart disease	National Center for Health Statistics (NCHS), Centers for Disease Control and Prevention (CDC). United States National Health and Nutrition Examination Survey 2013-2014. Hyattsville, United States: National Center for Health Statistics (NCHS), Centers for Disease Control and Prevention (CDC)
Ischemic heart disease	National Center for Health Statistics (NCHS), Centers for Disease Control and Prevention (CDC), United States Census Bureau. United States National Hospital Discharge Survey 1984. Hyattsville, United States: National Center for Health Statistics (NCHS), Centers for Disease Control and Prevention (CDC)
Ischemic heart disease	National Center for Health Statistics (NCHS), Centers for Disease Control and Prevention (CDC), United States Census Bureau. United States National Health Interview Survey 2013. Hyattsville, United States: National Center for Health Statistics (NCHS), Centers for Disease Control and Prevention (CDC), 2014
Ischemic heart disease	United States Department of Health and Human Services. Centers for Disease Control and Prevention. National Center for Health Statistics. National Health Interview Survey, 1994: Second Supplement on Aging. ICPSR02563-v3. Ann Arbor, MI: Inter-university Consortium for Political and Social Research [distributor], 2007-02-12. http://doi.org/10.3886/ICPSR02563.v3
Ischemic heart disease	National Center for Health Statistics (NCHS), Centers for Disease Control and Prevention (CDC), US Census Bureau. United States National Health Interview Survey 1997. Hyattsville, United States: National Center for Health Statistics (NCHS), Centers for Disease Control and Prevention (CDC)
Ischemic heart disease	Tunstall-Pedoe H, Kuulasmaa K, MÃ¤hÃ¶nen M, Tolonen H, Ruokokoski E. Contribution of trends in survival and coronary-event rates to changes in coronary heart disease mortality: 10-year results from 37 WHO MONICA Project populations. Lancet. 1999; 353(9164): 1547-57
Ischemic heart disease	Wellenius GA, Mittleman MA. Disparities in myocardial infarction case fatality rates among the elderly: the 20-year Medicare experience. Am Heart J. 2008; 156(3): 483Â–490
Ischemic heart disease	Hurt RD, Weston SA, Ebbert JO, McNallan SM, Croghan IT, Schroeder DR, Roger VL. Myocardial infarction and sudden cardiac death in Olmsted County, Minnesota, before and after smoke-free workplace laws. Arch Intern Med. 2012; 172(21): 1635-41
Ischemic heart disease	Goldberg RJ, Yarzebski J, Lessard D, Gore JM. A two-decades (1975 to 1995) long experience in the incidence, in-hospital and long-term case-fatality rates of acute myocardial infarction: a community-wide perspective. J Am Coll Cardiol. 1999; 33(6): 1533Â–9

© 2018 Global Burden of Cardiovascular Diseases Collaboration. *JAMA Cardiology*.

Ischemic heart disease	McManus DD, Piacentine SM, Lessard D, Gore JM, Yarzebski J, Spencer FA, Goldberg RJ. Thirty-Year (1975 to 2005) Trends in the Incidence Rates, Clinical Features, Treatment Practices, and Short-Term Outcomes of Patients Am J Cardiol. 2011; 108(4): 477-82
Ischemic heart disease	Ho PM, Eng MH, Rumsfeld JS, Spertus JA, Peterson PN, Jones PG, Peterson ED, Alexander KP, Havranek EP, Krumholz HM, Masoudi FA. The influence of age on health status outcomes after acute myocardial infarction. Am Heart J. 2008; 155(5): 855-61
Ischemic heart disease	McManus DD, Gore J, Yarzebski J, Spencer F, Lessard D, Goldberg RJ. Recent trends in the incidence, treatment, and outcomes of patients with STEMI and NSTEMI. Am J Med. 2011; 124(1): 40-47
Ischemic heart disease	Arciero TJ, Jacobsen SJ, Reeder GS, Frye RL, Weston SA, Killian JM, Roger Vr VÃ©. Temporal trends in the incidence of coronary disease. Am J Med. 2004; 117(4): 228-33
Ischemic stroke	Healthcare Cost and Utilization Project (HCUP), Agency for Healthcare Research and Quality (AHRQ). United States State Inpatient Databases 2009. Rockville, United States: Healthcare Cost and Utilization Project (HCUP), Agency for Healthcare Research and Quality (AHRQ)
Ischemic stroke	Healthcare Cost and Utilization Project (HCUP), Agency for Healthcare Research and Quality (AHRQ). United States State Inpatient Databases 2011. Rockville, United States: Healthcare Cost and Utilization Project (HCUP), Agency for Healthcare Research and Quality (AHRQ)
Ischemic stroke	Healthcare Cost and Utilization Project (HCUP), Agency for Healthcare Research and Quality (AHRQ). United States State Inpatient Databases 2003-2007
Ischemic stroke	Healthcare Cost and Utilization Project (HCUP), Agency for Healthcare Research and Quality (AHRQ). United States State Inpatient Databases 2008. Rockville, United States: Healthcare Cost and Utilization Project (HCUP), Agency for Healthcare Research and Quality (AHRQ)
Ischemic stroke	Healthcare Cost and Utilization Project (HCUP), Agency for Healthcare Research and Quality (AHRQ). United States State Inpatient Databases 2012. Rockville, United States: Healthcare Cost and Utilization Project (HCUP), Agency for Healthcare Research and Quality (AHRQ)
Ischemic stroke	Healthcare Cost and Utilization Project (HCUP), Agency for Healthcare Research and Quality (AHRQ). United States State Inpatient Databases 2013. Rockville, United States: Healthcare Cost and Utilization Project (HCUP), Agency for Healthcare Research and Quality (AHRQ)
Ischemic stroke	Healthcare Cost and Utilization Project (HCUP), Agency for Healthcare Research and Quality (AHRQ). United States State Inpatient Databases 2014. Rockville, United States: Healthcare Cost and Utilization Project (HCUP), Agency for Healthcare Research and Quality (AHRQ)
Ischemic stroke	Healthcare Cost and Utilization Project (HCUP), Agency for Healthcare Research and Quality (AHRQ). United States State Inpatient Databases 2006. Rockville, United States: Healthcare Cost and Utilization Project (HCUP), Agency for Healthcare Research and Quality (AHRQ)
Ischemic stroke	Healthcare Cost and Utilization Project (HCUP), Agency for Healthcare Research and Quality (AHRQ). United States State Inpatient Databases 2007. Rockville, United States: Healthcare Cost and Utilization Project (HCUP), Agency for Healthcare Research and Quality (AHRQ)
Ischemic stroke	Healthcare Cost and Utilization Project (HCUP), Agency for Healthcare Research and Quality (AHRQ). United States State Inpatient Databases 2005. Rockville, United States: Healthcare Cost and Utilization Project (HCUP), Agency for Healthcare Research and Quality (AHRQ)
Ischemic stroke	National Center for Health Statistics (NCHS), Centers for Disease Control and Prevention (CDC), US Census Bureau. United States National Health Interview Survey 2000. Hyattsville, United States: National Center for Health Statistics (NCHS), Centers for Disease Control and Prevention (CDC)
Ischemic stroke	Healthcare Cost and Utilization Project (HCUP), Agency for Healthcare Research and Quality (AHRQ). United States State Inpatient Databases 2003. Rockville, United States: Healthcare Cost and Utilization Project (HCUP), Agency for Healthcare Research and Quality (AHRQ)
Ischemic stroke	Healthcare Cost and Utilization Project (HCUP), Agency for Healthcare Research and Quality (AHRQ). United States Nationwide Inpatient Sample 2015. Rockville, United States: Healthcare Cost and Utilization Project (HCUP), Agency for Healthcare Research and Quality (AHRQ)
Ischemic stroke	National Center for Health Statistics (NCHS), Centers for Disease Control and Prevention (CDC), United States Census Bureau. United States National Hospital Discharge Survey 1999. Hyattsville, United States: National Center for Health Statistics (NCHS), Centers for Disease Control and Prevention (CDC)
Ischemic stroke	National Center for Health Statistics (NCHS), Centers for Disease Control and Prevention (CDC), United States Census Bureau. United States National Hospital Discharge Survey 1990. Hyattsville, United States: National Center for Health Statistics (NCHS), Centers for Disease Control and Prevention (CDC)
Ischemic stroke	National Center for Health Statistics (NCHS), Centers for Disease Control and Prevention (CDC), United States Census Bureau. United States National Hospital Discharge Survey 1981. Hyattsville, United States: National Center for Health Statistics (NCHS), Centers for Disease Control and Prevention (CDC)
Ischemic stroke	National Center for Health Statistics (NCHS), Centers for Disease Control and Prevention (CDC), United States Census Bureau. United States National Hospital Discharge Survey 1993. Hyattsville, United States: National Center for Health Statistics (NCHS), Centers for Disease Control and Prevention (CDC)
Ischemic stroke	Healthcare Cost and Utilization Project (HCUP), Agency for Healthcare Research and Quality (AHRQ). United States State Inpatient Databases 2004. Rockville, United States: Healthcare Cost and Utilization Project (HCUP), Agency for Healthcare Research and Quality (AHRQ)
Ischemic stroke	National Center for Health Statistics (NCHS), Centers for Disease Control and Prevention (CDC). United States National Health and Nutrition Examination Survey 2001-2002. Hyattsville, United States: National Center for Health Statistics (NCHS), Centers for Disease Control and Prevention (CDC)
Ischemic stroke	National Center for Health Statistics (NCHS), Centers for Disease Control and Prevention (CDC), United States Census Bureau. United States National Hospital Discharge Survey 1994. Hyattsville, United States: National Center for Health Statistics (NCHS), Centers for Disease Control and Prevention (CDC)

© 2018 Global Burden of Cardiovascular Diseases Collaboration. *JAMA Cardiology*.

Ischemic stroke	National Center for Health Statistics (NCHS), Centers for Disease Control and Prevention (CDC), United States Census Bureau. United States National Hospital Discharge Survey 1996. Hyattsville, United States: National Center for Health Statistics (NCHS), Centers for Disease Control and Prevention (CDC)
Ischemic stroke	National Center for Health Statistics (NCHS), Centers for Disease Control and Prevention (CDC), United States Census Bureau. United States National Hospital Discharge Survey 1987. Hyattsville, United States: National Center for Health Statistics (NCHS), Centers for Disease Control and Prevention (CDC)
Ischemic stroke	National Center for Health Statistics (NCHS), Centers for Disease Control and Prevention (CDC), United States Census Bureau. United States National Hospital Discharge Survey 1984. Hyattsville, United States: National Center for Health Statistics (NCHS), Centers for Disease Control and Prevention (CDC)
Ischemic stroke	National Center for Health Statistics (NCHS), Centers for Disease Control and Prevention (CDC), US Census Bureau. United States National Health Interview Survey 2009. Hyattsville, United States: National Center for Health Statistics (NCHS), Centers for Disease Control and Prevention (CDC)
Ischemic stroke	National Center for Health Statistics (NCHS), Centers for Disease Control and Prevention (CDC). United States National Health and Nutrition Examination Survey 2009-2010. Hyattsville, United States: National Center for Health Statistics (NCHS), Centers for Disease Control and Prevention (CDC), 2011
Ischemic stroke	National Center for Health Statistics (NCHS), Centers for Disease Control and Prevention (CDC). United States National Hospital Discharge Survey 1988-1992
Ischemic stroke	Health and Retirement Study, (Biennial 2012) public use dataset. Produced and distributed by the University of Michigan with funding from the National Institute on Aging (grant number NIA U01AG009740). Ann Arbor, MI, (2015)
Ischemic stroke	National Center for Health Statistics (NCHS), Centers for Disease Control and Prevention (CDC), United States Census Bureau. United States National Hospital Discharge Survey 2009. Hyattsville, United States: National Center for Health Statistics (NCHS), Centers for Disease Control and Prevention (CDC)
Ischemic stroke	National Center for Health Statistics (NCHS), Centers for Disease Control and Prevention (CDC). United States National Health and Nutrition Examination Survey 1988-1994. Hyattsville, United States: National Center for Health Statistics (NCHS), Centers for Disease Control and Prevention (CDC)
Ischemic stroke	National Center for Health Statistics (NCHS) Centers for Disease Control and Prevention (CDC). United States National Health and Nutrition Examination Survey 2011-2012. Hyattsville, United States: National Center for Health Statistics (NCHS) Centers for Disease Control and Prevention (CDC), 2013
Ischemic stroke	National Center for Health Statistics (NCHS), Centers for Disease Control and Prevention (CDC), US Census Bureau. United States National Health Interview Survey 2008. Hyattsville, United States: National Center for Health Statistics (NCHS), Centers for Disease Control and Prevention (CDC)
Ischemic stroke	Healthcare Cost and Utilization Project (HCUP), Agency for Healthcare Research and Quality (AHRQ). United States State Inpatient Databases 2008-2009
Ischemic stroke	Health and Retirement Study, (Biennial 1998) public use dataset. Produced and distributed by the University of Michigan with funding from the National Institute on Aging (grant number NIA U01AG009740). Ann Arbor, MI, (2014)
Ischemic stroke	National Center for Health Statistics (NCHS), Centers for Disease Control and Prevention (CDC), US Census Bureau. United States National Health Interview Survey 2001. Hyattsville, United States: National Center for Health Statistics (NCHS), Centers for Disease Control and Prevention (CDC)
Ischemic stroke	National Center for Health Statistics (NCHS), Centers for Disease Control and Prevention (CDC), United States Census Bureau. United States National Hospital Discharge Survey 1992. Hyattsville, United States: National Center for Health Statistics (NCHS), Centers for Disease Control and Prevention (CDC)
Ischemic stroke	National Center for Health Statistics (NCHS), Centers for Disease Control and Prevention (CDC). United States National Health and Nutrition Examination Survey 1999-2000. Hyattsville, United States: National Center for Health Statistics (NCHS), Centers for Disease Control and Prevention (CDC)
Ischemic stroke	National Center for Health Statistics (NCHS), Centers for Disease Control and Prevention (CDC), US Census Bureau. United States National Health Interview Survey 2011. Hyattsville, United States: National Center for Health Statistics (NCHS), Centers for Disease Control and Prevention (CDC)
Ischemic stroke	National Center for Health Statistics (NCHS), Centers for Disease Control and Prevention (CDC), US Census Bureau. United States National Health Interview Survey 2004. Hyattsville, United States: National Center for Health Statistics (NCHS), Centers for Disease Control and Prevention (CDC)
Ischemic stroke	National Center for Health Statistics (NCHS), Centers for Disease Control and Prevention (CDC), United States Census Bureau. United States National Hospital Discharge Survey 1983. Hyattsville, United States: National Center for Health Statistics (NCHS), Centers for Disease Control and Prevention (CDC)
Ischemic stroke	National Center for Health Statistics (NCHS), Centers for Disease Control and Prevention (CDC), US Census Bureau. United States National Health Interview Survey 2002. Hyattsville, United States: National Center for Health Statistics (NCHS), Centers for Disease Control and Prevention (CDC)
Ischemic stroke	National Center for Health Statistics (NCHS), Centers for Disease Control and Prevention (CDC), United States Census Bureau. United States National Health Interview Survey 2012. Hyattsville, United States: National Center for Health Statistics (NCHS), Centers for Disease Control and Prevention (CDC), 2013
Ischemic stroke	National Center for Health Statistics (NCHS), Centers for Disease Control and Prevention (CDC), United States Census Bureau. United States National Hospital Discharge Survey 2003. Hyattsville, United States: National Center for Health Statistics (NCHS), Centers for Disease Control and Prevention (CDC)
Ischemic stroke	National Center for Health Statistics (NCHS), Centers for Disease Control and Prevention (CDC), United States Census Bureau. United States National Hospital Discharge Survey 2000. Hyattsville, United States: National Center for Health Statistics (NCHS), Centers for Disease Control and Prevention (CDC)

© 2018 Global Burden of Cardiovascular Diseases Collaboration. *JAMA Cardiology*.

Ischemic stroke	National Center for Health Statistics (NCHS), Centers for Disease Control and Prevention (CDC). United States National Health and Nutrition Examination Survey 2005-2006. Hyattsville, United States: National Center for Health Statistics (NCHS), Centers for Disease Control and Prevention (CDC), 2007
Ischemic stroke	National Center for Health Statistics (NCHS), Centers for Disease Control and Prevention (CDC), United States Census Bureau. United States National Hospital Discharge Survey 2007. Hyattsville, United States: National Center for Health Statistics (NCHS), Centers for Disease Control and Prevention (CDC)
Ischemic stroke	National Center for Health Statistics (NCHS), Centers for Disease Control and Prevention (CDC), United States Census Bureau. United States National Hospital Discharge Survey 2006. Hyattsville, United States: National Center for Health Statistics (NCHS), Centers for Disease Control and Prevention (CDC)
Ischemic stroke	Washington State University, World Health Organization (WHO). United States WHO Multi-country Survey Study on Health and Health System Responsiveness 2000-2001. Geneva, Switzerland: World Health Organization (WHO)
Ischemic stroke	National Center for Health Statistics (NCHS), Centers for Disease Control and Prevention (CDC). United States National Health and Nutrition Examination Survey 2003-2004. Hyattsville, United States: National Center for Health Statistics (NCHS), Centers for Disease Control and Prevention (CDC)
Ischemic stroke	National Center for Health Statistics (NCHS), Centers for Disease Control and Prevention (CDC), US Census Bureau. United States National Health Interview Survey 1997. Hyattsville, United States: National Center for Health Statistics (NCHS), Centers for Disease Control and Prevention (CDC)
Ischemic stroke	National Center for Health Statistics (NCHS), Centers for Disease Control and Prevention (CDC), United States Census Bureau. United States National Hospital Discharge Survey 1995. Hyattsville, United States: National Center for Health Statistics (NCHS), Centers for Disease Control and Prevention (CDC)
Ischemic stroke	National Center for Health Statistics (NCHS), Centers for Disease Control and Prevention (CDC), United States Census Bureau. United States National Hospital Discharge Survey 1997. Hyattsville, United States: National Center for Health Statistics (NCHS), Centers for Disease Control and Prevention (CDC)
Ischemic stroke	National Center for Health Statistics (NCHS), Centers for Disease Control and Prevention (CDC), United States Census Bureau. United States National Hospital Discharge Survey 1988. Hyattsville, United States: National Center for Health Statistics (NCHS), Centers for Disease Control and Prevention (CDC)
Ischemic stroke	National Center for Health Statistics (NCHS), Centers for Disease Control and Prevention (CDC), US Census Bureau. United States National Health Interview Survey 2006. Hyattsville, United States: National Center for Health Statistics (NCHS), Centers for Disease Control and Prevention (CDC)
Ischemic stroke	National Center for Health Statistics (NCHS), Centers for Disease Control and Prevention (CDC), United States Census Bureau. United States National Hospital Discharge Survey 1982. Hyattsville, United States: National Center for Health Statistics (NCHS), Centers for Disease Control and Prevention (CDC)
Ischemic stroke	Health and Retirement Study, (Biennial 2002) public use dataset. Produced and distributed by the University of Michigan with funding from the National Institute on Aging (grant number NIA U01AG009740). Ann Arbor, MI, (2011)
Ischemic stroke	National Center for Health Statistics (NCHS), Centers for Disease Control and Prevention (CDC), US Census Bureau. United States National Health Interview Survey 1998. Hyattsville, United States: National Center for Health Statistics (NCHS), Centers for Disease Control and Prevention (CDC)
Ischemic stroke	National Center for Health Statistics (NCHS), Centers for Disease Control and Prevention (CDC), United States Census Bureau. United States National Hospital Discharge Survey 1991. Hyattsville, United States: National Center for Health Statistics (NCHS), Centers for Disease Control and Prevention (CDC)
Ischemic stroke	National Center for Health Statistics (NCHS), Centers for Disease Control and Prevention (CDC), United States Census Bureau. United States National Hospital Discharge Survey 1979. Hyattsville, United States: National Center for Health Statistics (NCHS), Centers for Disease Control and Prevention (CDC)
Ischemic stroke	National Center for Health Statistics (NCHS), Centers for Disease Control and Prevention (CDC). United States National Health Interview Survey 2015. Hyattsville, United States: National Center for Health Statistics (NCHS), Centers for Disease Control and Prevention (CDC), 2016
Ischemic stroke	National Center for Health Statistics (NCHS), Centers for Disease Control and Prevention (CDC). United States National Health Interview Survey 2014. Hyattsville, United States: National Center for Health Statistics (NCHS), Centers for Disease Control and Prevention (CDC), 2015
Ischemic stroke	National Center for Health Statistics (NCHS), Centers for Disease Control and Prevention (CDC), United States Census Bureau. United States National Hospital Discharge Survey 1985. Hyattsville, United States: National Center for Health Statistics (NCHS), Centers for Disease Control and Prevention (CDC)
Ischemic stroke	National Center for Health Statistics (NCHS), Centers for Disease Control and Prevention (CDC). United States National Health and Nutrition Examination Survey 2013-2014. Hyattsville, United States: National Center for Health Statistics (NCHS), Centers for Disease Control and Prevention (CDC)
Ischemic stroke	Healthcare Cost and Utilization Project (HCUP), Agency for Healthcare Research and Quality (AHRQ). United States Nationwide Inpatient Sample 2013. Rockville, United States: Healthcare Cost and Utilization Project (HCUP), Agency for Healthcare Research and Quality (AHRQ)
Ischemic stroke	Agency for Healthcare Research and Quality. United States Medical Expenditure Panel Survey 2002-2009. Rockville, United States: Agency for Healthcare Research and Quality
Ischemic stroke	National Center for Health Statistics (NCHS), Centers for Disease Control and Prevention (CDC), United States Census Bureau. United States National Hospital Discharge Survey 1989. Hyattsville, United States: National Center for Health Statistics (NCHS), Centers for Disease Control and Prevention (CDC)
Ischemic stroke	National Center for Health Statistics (NCHS), Centers for Disease Control and Prevention (CDC). United States National Hospital Discharge Survey 1998-2002
Ischemic stroke	National Center for Health Statistics (NCHS), Centers for Disease Control and Prevention (CDC), United States Census Bureau. United States National Hospital Discharge Survey 2002. Hyattsville, United States: National Center for Health Statistics (NCHS), Centers for Disease Control and Prevention (CDC)

© 2018 Global Burden of Cardiovascular Diseases Collaboration. *JAMA Cardiology*.

Ischemic stroke	Health Measurement Research Group, University of Wisconsin. United States National Health Measurement Study 2005-2006. Data and Information Sciences Center, University of Wisconsin-Madison [distributor]
Ischemic stroke	National Center for Health Statistics (NCHS), Centers for Disease Control and Prevention (CDC), US Census Bureau. United States National Health Interview Survey 1999. Hyattsville, United States: National Center for Health Statistics (NCHS), Centers for Disease Control and Prevention (CDC)
Ischemic stroke	National Center for Health Statistics (NCHS), Centers for Disease Control and Prevention (CDC), United States Census Bureau. United States National Health Interview Survey 2013. Hyattsville, United States: National Center for Health Statistics (NCHS), Centers for Disease Control and Prevention (CDC), 2014
Ischemic stroke	National Center for Health Statistics (NCHS), Centers for Disease Control and Prevention (CDC), US Census Bureau. United States National Health Interview Survey 1993. Hyattville, United States: National Center for Health Statistics (NCHS), Centers for Disease Control and Prevention (CDC)
Ischemic stroke	Healthcare Cost and Utilization Project (HCUP), Agency for Healthcare Research and Quality (AHRQ). United States Nationwide Inpatient Sample 2014. Rockville, United States: Healthcare Cost and Utilization Project (HCUP), Agency for Healthcare Research and Quality (AHRQ)
Ischemic stroke	United States Department of Health and Human Services. Centers for Disease Control and Prevention. National Center for Health Statistics. National Health Interview Survey, 1994: Second Longitudinal Study on Aging, Wave 2, 1997. ICPSR03526-v2. Ann Arbor, MI: Inter-university Consortium for Political and Social Research [distributor], 2007-03-01. http://doi.org/10.3886/ICPSR03526.v2
Ischemic stroke	Health and Retirement Study, (Biennial 2010) public use dataset. Produced and distributed by the University of Michigan with funding from the National Institute on Aging (grant number NIA U01AG009740). Ann Arbor, MI, (2014)
Ischemic stroke	Truven Health Analytics. United States MarketScan Commercial Claims and Encounters Database 2010. Ann Arbor, United States: Truven Health Analytics
Ischemic stroke	National Center for Health Statistics (NCHS), Centers for Disease Control and Prevention (CDC), US Census Bureau. United States National Health Interview Survey 2003. Hyattsville, United States: National Center for Health Statistics (NCHS), Centers for Disease Control and Prevention (CDC)
Ischemic stroke	National Center for Health Statistics (NCHS), Centers for Disease Control and Prevention (CDC). United States National Health and Nutrition Examination Survey 2007-2008. Hyattsville, United States: National Center for Health Statistics (NCHS), Centers for Disease Control and Prevention (CDC), 2009
Ischemic stroke	National Center for Health Statistics (NCHS), Centers for Disease Control and Prevention (CDC), United States Census Bureau. United States National Hospital Discharge Survey 1998. Hyattsville, United States: National Center for Health Statistics (NCHS), Centers for Disease Control and Prevention (CDC)
Ischemic stroke	National Center for Health Statistics (NCHS), Centers for Disease Control and Prevention (CDC), United States Census Bureau. United States National Hospital Discharge Survey 1980. Hyattsville, United States: National Center for Health Statistics (NCHS), Centers for Disease Control and Prevention (CDC)
Ischemic stroke	National Center for Health Statistics (NCHS), Centers for Disease Control and Prevention (CDC). United States National Hospital Discharge Survey 2008-2010
Ischemic stroke	National Center for Health Statistics (NCHS), Centers for Disease Control and Prevention (CDC), United States Census Bureau. United States National Hospital Discharge Survey 2004. Hyattsville, United States: National Center for Health Statistics (NCHS), Centers for Disease Control and Prevention (CDC)
Ischemic stroke	National Center for Health Statistics (NCHS), Centers for Disease Control and Prevention (CDC), US Census Bureau. United States National Health Interview Survey 2007. Hyattsville, United States: National Center for Health Statistics (NCHS), Centers for Disease Control and Prevention (CDC)
Ischemic stroke	Health and Retirement Study, (Biennial 2000) public use dataset. Produced and distributed by the University of Michigan with funding from the National Institute on Aging (grant number NIA U01AG009740). Ann Arbor, MI, (2011)
Ischemic stroke	Health and Retirement Study, (Biennial 2004) public use dataset. Produced and distributed by the University of Michigan with funding from the National Institute on Aging (grant number NIA U01AG009740). Ann Arbor, MI, (2013)
Ischemic stroke	Health and Retirement Study, (Biennial 2006) public use dataset. Produced and distributed by the University of Michigan with funding from the National Institute on Aging (grant number NIA U01AG009740). Ann Arbor, MI, (2014)
Ischemic stroke	Panel Study of Income Dynamics, 2007 public use dataset. Produced and distributed by the University of Michigan with primary funding from the National Science Foundation, the National Institute of Aging, and the National Institute of Child Health and Human Development. Ann Arbor, MI, (2011)
Ischemic stroke	Panel Study of Income Dynamics, 2005 public use dataset. Produced and distributed by the University of Michigan with primary funding from the National Science Foundation, the National Institute of Aging, and the National Institute of Child Health and Human Development. Ann Arbor, MI, (2011)
Ischemic stroke	National Center for Health Statistics (NCHS), Centers for Disease Control and Prevention (CDC), US Census Bureau. United States National Health Interview Survey 2010. Hyattsville, United States: National Center for Health Statistics (NCHS), Centers for Disease Control and Prevention (CDC)
Ischemic stroke	National Center for Health Statistics (NCHS), Centers for Disease Control and Prevention (CDC), United States Census Bureau. United States National Hospital Discharge Survey 2010. Hyattsville, United States: National Center for Health Statistics (NCHS), Centers for Disease Control and Prevention (CDC)
Ischemic stroke	National Center for Health Statistics (NCHS), Centers for Disease Control and Prevention (CDC), United States Census Bureau. United States National Hospital Discharge Survey 2008. Hyattsville, United States: National Center for Health Statistics (NCHS), Centers for Disease Control and Prevention (CDC)

© 2018 Global Burden of Cardiovascular Diseases Collaboration. *JAMA Cardiology*.

Ischemic stroke	National Center for Health Statistics (NCHS), Centers for Disease Control and Prevention (CDC), US Census Bureau. United States National Health Interview Survey 2005. Hyattsville, United States: National Center for Health Statistics (NCHS), Centers for Disease Control and Prevention (CDC)
Ischemic stroke	United States Department of Health and Human Services. Centers for Disease Control and Prevention. National Center for Health Statistics. National Health Interview Survey, 1994: Second Supplement on Aging. ICPSR02563-v3. Ann Arbor, MI: Inter-university Consortium for Political and Social Research [distributor], 2007-02-12. http://doi.org/10.3886/ICPSR02563.v3
Ischemic stroke	National Center for Health Statistics (NCHS), Centers for Disease Control and Prevention (CDC), United States Census Bureau. United States National Hospital Discharge Survey 2001. Hyattsville, United States: National Center for Health Statistics (NCHS), Centers for Disease Control and Prevention (CDC)
Ischemic stroke	National Center for Health Statistics (NCHS), Centers for Disease Control and Prevention (CDC), United States Census Bureau. United States National Hospital Discharge Survey 1986. Hyattsville, United States: National Center for Health Statistics (NCHS), Centers for Disease Control and Prevention (CDC)
Ischemic stroke	National Center for Health Statistics (NCHS), Centers for Disease Control and Prevention (CDC), US Census Bureau. United States National Health Interview Survey 1990. Hyattsville, United States: National Center for Health Statistics (NCHS), Centers for Disease Control and Prevention (CDC)
Ischemic stroke	National Center for Health Statistics (NCHS), Centers for Disease Control and Prevention (CDC). United States National Hospital Discharge Survey 1993-1997
Ischemic stroke	National Center for Health Statistics (NCHS), Centers for Disease Control and Prevention (CDC). United States National Hospital Discharge Survey 2003-2007
Ischemic stroke	National Center for Health Statistics (NCHS), Centers for Disease Control and Prevention (CDC), US Census Bureau. United States National Health Interview Survey 1991. Hyattsville, United States: National Center for Health Statistics (NCHS), Centers for Disease Control and Prevention (CDC)
Ischemic stroke	National Center for Health Statistics (NCHS), Centers for Disease Control and Prevention (CDC), United States Census Bureau. United States National Hospital Discharge Survey 2005. Hyattsville, United States: National Center for Health Statistics (NCHS), Centers for Disease Control and Prevention (CDC)
Ischemic stroke	National Center for Health Statistics (NCHS), Centers for Disease Control and Prevention (CDC), US Census Bureau. United States National Health Interview Survey 1992. Hyattsville, United States: National Center for Health Statistics (NCHS), Centers for Disease Control and Prevention (CDC)
Ischemic stroke	Zahuranec DB, Brown DL, Lisabeth LD, Morgenstern LB. Is it time for a large, collaborative study of pediatric stroke?. Stroke. 2005; 36(9): 1825-9
Ischemic stroke	Earley CJ, Kittner SJ, Feeser BR, Gardner J, Epstein A, Wozniak MA, Wityk R, Stern BJ, Price TR, Macko RF, Johnson C, Sloan MA, Buchholz D. Stroke in children and sickle-cell disease: Baltimore-Washington Cooperative Young Stroke Study. Neurology. 1998; 51(1): 169-76
Ischemic stroke	Health and Retirement Study, (Biennial 1992) public use dataset. Produced and distributed by the University of Michigan with funding from the National Institute on Aging (grant number NIA U01AG009740). Ann Arbor, MI, (2011)
Ischemic stroke	Health and Retirement Study, (Biennial 1996) public use dataset. Produced and distributed by the University of Michigan with funding from the National Institute on Aging (grant number NIA U01AG009740). Ann Arbor, MI, (2011)
Myocarditis	Healthcare Cost and Utilization Project (HCUP), Agency for Healthcare Research and Quality (AHRQ). United States State Inpatient Databases 2011. Rockville, United States: Healthcare Cost and Utilization Project (HCUP), Agency for Healthcare Research and Quality (AHRQ)
Myocarditis	Healthcare Cost and Utilization Project (HCUP), Agency for Healthcare Research and Quality (AHRQ). United States State Inpatient Databases 2014. Rockville, United States: Healthcare Cost and Utilization Project (HCUP), Agency for Healthcare Research and Quality (AHRQ)
Myocarditis	Healthcare Cost and Utilization Project (HCUP), Agency for Healthcare Research and Quality (AHRQ). United States State Inpatient Databases 2013. Rockville, United States: Healthcare Cost and Utilization Project (HCUP), Agency for Healthcare Research and Quality (AHRQ)
Myocarditis	Healthcare Cost and Utilization Project (HCUP), Agency for Healthcare Research and Quality (AHRQ). United States State Inpatient Databases 2012. Rockville, United States: Healthcare Cost and Utilization Project (HCUP), Agency for Healthcare Research and Quality (AHRQ)
Myocarditis	Truven Health Analytics. United States MarketScan Claims and Medicare Data - 2010. Ann Arbor, United States: Truven Health Analytics
Myocarditis	Healthcare Cost and Utilization Project (HCUP), Agency for Healthcare Research and Quality (AHRQ). United States State Inpatient Databases 2009. Rockville, United States: Healthcare Cost and Utilization Project (HCUP), Agency for Healthcare Research and Quality (AHRQ)
Myocarditis	Healthcare Cost and Utilization Project (HCUP), Agency for Healthcare Research and Quality (AHRQ). United States State Inpatient Databases 2007. Rockville, United States: Healthcare Cost and Utilization Project (HCUP), Agency for Healthcare Research and Quality (AHRQ)
Myocarditis	Healthcare Cost and Utilization Project (HCUP), Agency for Healthcare Research and Quality (AHRQ). United States State Inpatient Databases 2005. Rockville, United States: Healthcare Cost and Utilization Project (HCUP), Agency for Healthcare Research and Quality (AHRQ)
Myocarditis	Healthcare Cost and Utilization Project (HCUP), Agency for Healthcare Research and Quality (AHRQ). United States State Inpatient Databases 2004. Rockville, United States: Healthcare Cost and Utilization Project (HCUP), Agency for Healthcare Research and Quality (AHRQ)
Myocarditis	National Center for Health Statistics (NCHS), Centers for Disease Control and Prevention (CDC), United States Census Bureau. United States National Hospital Discharge Survey 2007. Hyattsville, United States: National Center for Health Statistics (NCHS), Centers for Disease Control and Prevention (CDC)

© 2018 Global Burden of Cardiovascular Diseases Collaboration. *JAMA Cardiology*.

Myocarditis	National Center for Health Statistics (NCHS), Centers for Disease Control and Prevention (CDC), United States Census Bureau. United States National Hospital Discharge Survey 2002. Hyattsville, United States: National Center for Health Statistics (NCHS), Centers for Disease Control and Prevention (CDC)
Myocarditis	National Center for Health Statistics (NCHS), Centers for Disease Control and Prevention (CDC), US Census Bureau. United States National Health Interview Survey 1999. Hyattsville, United States: National Center for Health Statistics (NCHS), Centers for Disease Control and Prevention (CDC)
Myocarditis	National Center for Health Statistics (NCHS), Centers for Disease Control and Prevention (CDC), United States Census Bureau. United States National Hospital Discharge Survey 1999. Hyattsville, United States: National Center for Health Statistics (NCHS), Centers for Disease Control and Prevention (CDC)
Myocarditis	National Center for Health Statistics (NCHS), Centers for Disease Control and Prevention (CDC), United States Census Bureau. United States National Hospital Discharge Survey 1981. Hyattsville, United States: National Center for Health Statistics (NCHS), Centers for Disease Control and Prevention (CDC)
Myocarditis	National Center for Health Statistics (NCHS), Centers for Disease Control and Prevention (CDC), US Census Bureau. United States National Health Interview Survey 1992. Hyattsville, United States: National Center for Health Statistics (NCHS), Centers for Disease Control and Prevention (CDC)
Myocarditis	Healthcare Cost and Utilization Project (HCUP), Agency for Healthcare Research and Quality (AHRQ). United States State Inpatient Databases 2006. Rockville, United States: Healthcare Cost and Utilization Project (HCUP), Agency for Healthcare Research and Quality (AHRQ)
Myocarditis	Healthcare Cost and Utilization Project (HCUP), Agency for Healthcare Research and Quality (AHRQ). United States State Inpatient Databases 2008. Rockville, United States: Healthcare Cost and Utilization Project (HCUP), Agency for Healthcare Research and Quality (AHRQ)
Myocarditis	Healthcare Cost and Utilization Project (HCUP), Agency for Healthcare Research and Quality (AHRQ). United States State Inpatient Databases 2003. Rockville, United States: Healthcare Cost and Utilization Project (HCUP), Agency for Healthcare Research and Quality (AHRQ)
Myocarditis	National Center for Health Statistics (NCHS), Centers for Disease Control and Prevention (CDC), United States Census Bureau. United States National Hospital Discharge Survey 2004. Hyattsville, United States: National Center for Health Statistics (NCHS), Centers for Disease Control and Prevention (CDC)
Myocarditis	National Center for Health Statistics (NCHS), Centers for Disease Control and Prevention (CDC), United States Census Bureau. United States National Hospital Discharge Survey 1993. Hyattsville, United States: National Center for Health Statistics (NCHS), Centers for Disease Control and Prevention (CDC)
Myocarditis	National Center for Health Statistics (NCHS), Centers for Disease Control and Prevention (CDC), United States Census Bureau. United States National Hospital Discharge Survey 2005. Hyattsville, United States: National Center for Health Statistics (NCHS), Centers for Disease Control and Prevention (CDC)
Myocarditis	National Center for Health Statistics (NCHS), Centers for Disease Control and Prevention (CDC), United States Census Bureau. United States National Hospital Discharge Survey 2010. Hyattsville, United States: National Center for Health Statistics (NCHS), Centers for Disease Control and Prevention (CDC)
Myocarditis	National Center for Health Statistics (NCHS) Centers for Disease Control and Prevention (CDC). United States National Health and Nutrition Examination Survey 2011-2012. Hyattsville, United States: National Center for Health Statistics (NCHS) Centers for Disease Control and Prevention (CDC), 2013
Myocarditis	National Center for Health Statistics (NCHS), Centers for Disease Control and Prevention (CDC), United States Census Bureau. United States National Hospital Discharge Survey 1992. Hyattsville, United States: National Center for Health Statistics (NCHS), Centers for Disease Control and Prevention (CDC)
Myocarditis	Truven Health Analytics. United States MarketScan Claims and Medicare Data - 2012. Ann Arbor, United States: Truven Health Analytics
Myocarditis	National Center for Health Statistics (NCHS), Centers for Disease Control and Prevention (CDC), US Census Bureau. United States National Health Interview Survey 1991. Hyattsville, United States: National Center for Health Statistics (NCHS), Centers for Disease Control and Prevention (CDC)
Myocarditis	National Center for Health Statistics (NCHS), Centers for Disease Control and Prevention (CDC), United States Census Bureau. United States National Hospital Discharge Survey 1987. Hyattsville, United States: National Center for Health Statistics (NCHS), Centers for Disease Control and Prevention (CDC)
Myocarditis	National Center for Health Statistics (NCHS), Centers for Disease Control and Prevention (CDC), US Census Bureau. United States National Health Interview Survey 2004. Hyattsville, United States: National Center for Health Statistics (NCHS), Centers for Disease Control and Prevention (CDC)
Myocarditis	National Center for Health Statistics (NCHS), Centers for Disease Control and Prevention (CDC), United States Census Bureau. United States National Hospital Discharge Survey 2003. Hyattsville, United States: National Center for Health Statistics (NCHS), Centers for Disease Control and Prevention (CDC)
Myocarditis	National Center for Health Statistics (NCHS), Centers for Disease Control and Prevention (CDC), United States Census Bureau. United States National Hospital Discharge Survey 2000. Hyattsville, United States: National Center for Health Statistics (NCHS), Centers for Disease Control and Prevention (CDC)
Myocarditis	National Center for Health Statistics (NCHS), Centers for Disease Control and Prevention (CDC), United States Census Bureau. United States National Hospital Discharge Survey 2006. Hyattsville, United States: National Center for Health Statistics (NCHS), Centers for Disease Control and Prevention (CDC)
Myocarditis	National Center for Health Statistics (NCHS), Centers for Disease Control and Prevention (CDC), United States Census Bureau. United States National Health Interview Survey 2012. Hyattsville, United States: National Center for Health Statistics (NCHS), Centers for Disease Control and Prevention (CDC), 2013
Myocarditis	National Center for Health Statistics (NCHS), Centers for Disease Control and Prevention (CDC). United States National Health and Nutrition Examination Survey 2005-2006. Hyattsville, United States: National Center for Health Statistics (NCHS), Centers for Disease Control and Prevention (CDC), 2007

© 2018 Global Burden of Cardiovascular Diseases Collaboration. *JAMA Cardiology*.

Myocarditis	National Center for Health Statistics (NCHS), Centers for Disease Control and Prevention (CDC), United States Census Bureau. United States National Hospital Discharge Survey 1994. Hyattsville, United States: National Center for Health Statistics (NCHS), Centers for Disease Control and Prevention (CDC)
Myocarditis	Washington State University, World Health Organization (WHO). United States WHO Multi-country Survey Study on Health and Health System Responsiveness 2000-2001. Geneva, Switzerland: World Health Organization (WHO)
Myocarditis	National Center for Health Statistics (NCHS), Centers for Disease Control and Prevention (CDC), United States Census Bureau. United States National Hospital Discharge Survey 1990. Hyattsville, United States: National Center for Health Statistics (NCHS), Centers for Disease Control and Prevention (CDC)
Myocarditis	National Center for Health Statistics (NCHS), Centers for Disease Control and Prevention (CDC), US Census Bureau. United States National Health Interview Survey 1990. Hyattsville, United States: National Center for Health Statistics (NCHS), Centers for Disease Control and Prevention (CDC)
Myocarditis	Panel Study of Income Dynamics, 2005 public use dataset. Produced and distributed by the University of Michigan with primary funding from the National Science Foundation, the National Institute of Aging, and the National Institute of Child Health and Human Development. Ann Arbor, MI, (2011)
Myocarditis	National Center for Health Statistics (NCHS), Centers for Disease Control and Prevention (CDC), United States Census Bureau. United States National Hospital Discharge Survey 1988. Hyattsville, United States: National Center for Health Statistics (NCHS), Centers for Disease Control and Prevention (CDC)
Myocarditis	National Center for Health Statistics (NCHS), Centers for Disease Control and Prevention (CDC), United States Census Bureau. United States National Hospital Discharge Survey 2009. Hyattsville, United States: National Center for Health Statistics (NCHS), Centers for Disease Control and Prevention (CDC)
Myocarditis	National Center for Health Statistics (NCHS), Centers for Disease Control and Prevention (CDC), United States Census Bureau. United States National Hospital Discharge Survey 1982. Hyattsville, United States: National Center for Health Statistics (NCHS), Centers for Disease Control and Prevention (CDC)
Myocarditis	Truven Health Analytics. United States MarketScan Claims and Medicare Data - 2000. Ann Arbor, United States: Truven Health Analytics
Myocarditis	National Center for Health Statistics (NCHS), Centers for Disease Control and Prevention (CDC), United States Census Bureau. United States National Hospital Discharge Survey 1989. Hyattsville, United States: National Center for Health Statistics (NCHS), Centers for Disease Control and Prevention (CDC)
Myocarditis	National Center for Health Statistics (NCHS), Centers for Disease Control and Prevention (CDC), United States Census Bureau. United States National Hospital Discharge Survey 1980. Hyattsville, United States: National Center for Health Statistics (NCHS), Centers for Disease Control and Prevention (CDC)
Myocarditis	Healthcare Cost and Utilization Project (HCUP), Agency for Healthcare Research and Quality (AHRQ). United States Nationwide Inpatient Sample 2013. Rockville, United States: Healthcare Cost and Utilization Project (HCUP), Agency for Healthcare Research and Quality (AHRQ)
Myocarditis	National Center for Health Statistics (NCHS), Centers for Disease Control and Prevention (CDC). United States National Health Interview Survey 2014. Hyattsville, United States: National Center for Health Statistics (NCHS), Centers for Disease Control and Prevention (CDC), 2015
Myocarditis	National Center for Health Statistics (NCHS), Centers for Disease Control and Prevention (CDC), US Census Bureau. United States National Health Interview Survey 2001. Hyattsville, United States: National Center for Health Statistics (NCHS), Centers for Disease Control and Prevention (CDC)
Myocarditis	National Center for Health Statistics (NCHS), Centers for Disease Control and Prevention (CDC), United States Census Bureau. United States National Hospital Discharge Survey 1979. Hyattsville, United States: National Center for Health Statistics (NCHS), Centers for Disease Control and Prevention (CDC)
Myocarditis	Panel Study of Income Dynamics, 2007 public use dataset. Produced and distributed by the University of Michigan with primary funding from the National Science Foundation, the National Institute of Aging, and the National Institute of Child Health and Human Development. Ann Arbor, MI, (2011)
Myocarditis	Healthcare Cost and Utilization Project (HCUP), Agency for Healthcare Research and Quality (AHRQ). United States Nationwide Inpatient Sample 2014. Rockville, United States: Healthcare Cost and Utilization Project (HCUP), Agency for Healthcare Research and Quality (AHRQ)
Myocarditis	National Center for Health Statistics (NCHS), Centers for Disease Control and Prevention (CDC), US Census Bureau. United States National Health Interview Survey 1993. Hyattville, United States: National Center for Health Statistics (NCHS), Centers for Disease Control and Prevention (CDC)
Myocarditis	Healthcare Cost and Utilization Project (HCUP), Agency for Healthcare Research and Quality (AHRQ). United States Nationwide Inpatient Sample 2015. Rockville, United States: Healthcare Cost and Utilization Project (HCUP), Agency for Healthcare Research and Quality (AHRQ)
Myocarditis	National Center for Health Statistics (NCHS), Centers for Disease Control and Prevention (CDC), US Census Bureau. United States National Health Interview Survey 2011. Hyattsville, United States: National Center for Health Statistics (NCHS), Centers for Disease Control and Prevention (CDC)
Myocarditis	National Center for Health Statistics (NCHS), Centers for Disease Control and Prevention (CDC), United States Census Bureau. United States National Hospital Discharge Survey 1986. Hyattsville, United States: National Center for Health Statistics (NCHS), Centers for Disease Control and Prevention (CDC)
Myocarditis	National Center for Health Statistics (NCHS), Centers for Disease Control and Prevention (CDC), United States Census Bureau. United States National Hospital Discharge Survey 2008. Hyattsville, United States: National Center for Health Statistics (NCHS), Centers for Disease Control and Prevention (CDC)
Myocarditis	National Center for Health Statistics (NCHS), Centers for Disease Control and Prevention (CDC), United States Census Bureau. United States National Hospital Discharge Survey 2001. Hyattsville, United States: National Center for Health Statistics (NCHS), Centers for Disease Control and Prevention (CDC)

© 2018 Global Burden of Cardiovascular Diseases Collaboration. *JAMA Cardiology*.

Myocarditis	National Center for Health Statistics (NCHS), Centers for Disease Control and Prevention (CDC), United States Census Bureau. United States National Hospital Discharge Survey 1995. Hyattsville, United States: National Center for Health Statistics (NCHS), Centers for Disease Control and Prevention (CDC)
Myocarditis	National Center for Health Statistics (NCHS), Centers for Disease Control and Prevention (CDC), United States Census Bureau. United States National Hospital Discharge Survey 1998. Hyattsville, United States: National Center for Health Statistics (NCHS), Centers for Disease Control and Prevention (CDC)
Myocarditis	National Center for Health Statistics (NCHS), Centers for Disease Control and Prevention (CDC), US Census Bureau. United States National Health Interview Survey 2010. Hyattsville, United States: National Center for Health Statistics (NCHS), Centers for Disease Control and Prevention (CDC)
Myocarditis	National Center for Health Statistics (NCHS), Centers for Disease Control and Prevention (CDC), United States Census Bureau. United States National Hospital Discharge Survey 1983. Hyattsville, United States: National Center for Health Statistics (NCHS), Centers for Disease Control and Prevention (CDC)
Myocarditis	National Center for Health Statistics (NCHS), Centers for Disease Control and Prevention (CDC). United States National Health and Nutrition Examination Survey 2013-2014. Hyattsville, United States: National Center for Health Statistics (NCHS), Centers for Disease Control and Prevention (CDC)
Myocarditis	National Center for Health Statistics (NCHS), Centers for Disease Control and Prevention (CDC). United States National Hospital Discharge Survey 1988-1992
Myocarditis	National Center for Health Statistics (NCHS), Centers for Disease Control and Prevention (CDC). United States National Hospital Discharge Survey 1993-1997
Myocarditis	National Center for Health Statistics (NCHS), Centers for Disease Control and Prevention (CDC). United States National Hospital Discharge Survey 1998-2002
Myocarditis	National Center for Health Statistics (NCHS), Centers for Disease Control and Prevention (CDC), United States Census Bureau. United States National Hospital Discharge Survey 1984. Hyattsville, United States: National Center for Health Statistics (NCHS), Centers for Disease Control and Prevention (CDC)
Myocarditis	National Center for Health Statistics (NCHS), Centers for Disease Control and Prevention (CDC). United States National Hospital Discharge Survey 2003-2007
Myocarditis	National Center for Health Statistics (NCHS), Centers for Disease Control and Prevention (CDC). United States National Hospital Discharge Survey 2008-2010
Myocarditis	National Center for Health Statistics (NCHS), Centers for Disease Control and Prevention (CDC). United States National Health and Nutrition Examination Survey 2003-2004. Hyattsville, United States: National Center for Health Statistics (NCHS), Centers for Disease Control and Prevention (CDC)
Myocarditis	National Center for Health Statistics (NCHS), Centers for Disease Control and Prevention (CDC), United States Census Bureau. United States National Hospital Discharge Survey 1985. Hyattsville, United States: National Center for Health Statistics (NCHS), Centers for Disease Control and Prevention (CDC)
Myocarditis	National Center for Health Statistics (NCHS), Centers for Disease Control and Prevention (CDC), US Census Bureau. United States National Health Interview Survey 2003. Hyattsville, United States: National Center for Health Statistics (NCHS), Centers for Disease Control and Prevention (CDC)
Myocarditis	National Center for Health Statistics (NCHS), Centers for Disease Control and Prevention (CDC). United States National Health and Nutrition Examination Survey 2007-2008. Hyattsville, United States: National Center for Health Statistics (NCHS), Centers for Disease Control and Prevention (CDC), 2009
Myocarditis	National Center for Health Statistics (NCHS), Centers for Disease Control and Prevention (CDC), United States Census Bureau. United States National Health Interview Survey 2013. Hyattsville, United States: National Center for Health Statistics (NCHS), Centers for Disease Control and Prevention (CDC), 2014
Myocarditis	National Center for Health Statistics (NCHS), Centers for Disease Control and Prevention (CDC), US Census Bureau. United States National Health Interview Survey 2006. Hyattsville, United States: National Center for Health Statistics (NCHS), Centers for Disease Control and Prevention (CDC)
Myocarditis	National Center for Health Statistics (NCHS), Centers for Disease Control and Prevention (CDC). United States National Health and Nutrition Examination Survey 2001-2002. Hyattsville, United States: National Center for Health Statistics (NCHS), Centers for Disease Control and Prevention (CDC)
Myocarditis	United States Department of Health and Human Services. Centers for Disease Control and Prevention. National Center for Health Statistics. National Health Interview Survey, 1994: Second Supplement on Aging. ICPSR02563-v3. Ann Arbor, MI: Inter-university Consortium for Political and Social Research [distributor], 2007-02-12. http://doi.org/10.3886/ICPSR02563.v3
Myocarditis	National Center for Health Statistics (NCHS), Centers for Disease Control and Prevention (CDC), US Census Bureau. United States National Health Interview Survey 2007. Hyattsville, United States: National Center for Health Statistics (NCHS), Centers for Disease Control and Prevention (CDC)
Myocarditis	National Center for Health Statistics (NCHS), Centers for Disease Control and Prevention (CDC), United States Census Bureau. United States National Hospital Discharge Survey 1996. Hyattsville, United States: National Center for Health Statistics (NCHS), Centers for Disease Control and Prevention (CDC)
Myocarditis	United States Department of Health and Human Services. Centers for Disease Control and Prevention. National Center for Health Statistics. National Health Interview Survey, 1994: Second Longitudinal Study on Aging, Wave 2, 1997. ICPSR03526-v2. Ann Arbor, MI: Inter-university Consortium for Political and Social Research [distributor], 2007-03-01. http://doi.org/10.3886/ICPSR03526.v2
Myocarditis	National Center for Health Statistics (NCHS), Centers for Disease Control and Prevention (CDC), US Census Bureau. United States National Health Interview Survey 2008. Hyattsville, United States: National Center for Health Statistics (NCHS), Centers for Disease Control and Prevention (CDC)
Myocarditis	National Center for Health Statistics (NCHS), Centers for Disease Control and Prevention (CDC). United States National Health and Nutrition Examination Survey 1999-2000. Hyattsville, United States: National Center for Health Statistics (NCHS), Centers for Disease Control and Prevention (CDC)

© 2018 Global Burden of Cardiovascular Diseases Collaboration. *JAMA Cardiology*.

Myocarditis	National Center for Health Statistics (NCHS), Centers for Disease Control and Prevention (CDC), United States Census Bureau. United States National Hospital Discharge Survey 1991. Hyattsville, United States: National Center for Health Statistics (NCHS), Centers for Disease Control and Prevention (CDC)
Myocarditis	National Center for Health Statistics (NCHS), Centers for Disease Control and Prevention (CDC), US Census Bureau. United States National Health Interview Survey 2002. Hyattsville, United States: National Center for Health Statistics (NCHS), Centers for Disease Control and Prevention (CDC)
Myocarditis	National Center for Health Statistics (NCHS), Centers for Disease Control and Prevention (CDC). United States National Health and Nutrition Examination Survey 1988-1994. Hyattsville, United States: National Center for Health Statistics (NCHS), Centers for Disease Control and Prevention (CDC)
Myocarditis	National Center for Health Statistics (NCHS), Centers for Disease Control and Prevention (CDC), US Census Bureau. United States National Health Interview Survey 2000. Hyattsville, United States: National Center for Health Statistics (NCHS), Centers for Disease Control and Prevention (CDC)
Myocarditis	National Center for Health Statistics (NCHS), Centers for Disease Control and Prevention (CDC), US Census Bureau. United States National Health Interview Survey 1997. Hyattsville, United States: National Center for Health Statistics (NCHS), Centers for Disease Control and Prevention (CDC)
Myocarditis	National Center for Health Statistics (NCHS), Centers for Disease Control and Prevention (CDC), United States Census Bureau. United States National Hospital Discharge Survey 1997. Hyattsville, United States: National Center for Health Statistics (NCHS), Centers for Disease Control and Prevention (CDC)
Myocarditis	National Center for Health Statistics (NCHS), Centers for Disease Control and Prevention (CDC). United States National Health and Nutrition Examination Survey 2009-2010. Hyattsville, United States: National Center for Health Statistics (NCHS), Centers for Disease Control and Prevention (CDC), 2011
Myocarditis	National Center for Health Statistics (NCHS), Centers for Disease Control and Prevention (CDC), US Census Bureau. United States National Health Interview Survey 1998. Hyattsville, United States: National Center for Health Statistics (NCHS), Centers for Disease Control and Prevention (CDC)
Myocarditis	National Center for Health Statistics (NCHS), Centers for Disease Control and Prevention (CDC), US Census Bureau. United States National Health Interview Survey 2009. Hyattsville, United States: National Center for Health Statistics (NCHS), Centers for Disease Control and Prevention (CDC)
Peripheral artery disease	National Center for Health Statistics (NCHS), Centers for Disease Control and Prevention (CDC). United States National Health and Nutrition Examination Survey 1976-1980. Hyattsville, United States: National Center for Health Statistics (NCHS), Centers for Disease Control and Prevention (CDC)
Peripheral artery disease	Truven Health Analytics. United States MarketScan Claims and Medicare Data - 2000. Ann Arbor, United States: Truven Health Analytics
Peripheral artery disease	Lamar Welch VL, Casper M, Greenlund K, Zheng Z-J, Giles W, Rith-Najarian S. Prevalence of lower extremity arterial disease defined by the ankle-brachial index among American Indians: the Inter-Tribal Heart Project. Ethn Dis. 2002; 12(1): S1-63-7
Peripheral artery disease	Truven Health Analytics. United States MarketScan Claims and Medicare Data - 2010. Ann Arbor, United States: Truven Health Analytics
Peripheral artery disease	Jones WS, Patel MR, Rockman CB, Guo Y, Adelman M, Riles T, Berger JS. Association of the ankle-brachial index with history of myocardial infarction and stroke. Am Heart J. 2014; 167(4): 499Â–505
Peripheral artery disease	National Center for Health Statistics (NCHS), Centers for Disease Control and Prevention (CDC). United States National Health and Nutrition Examination Survey 2001-2002. Hyattsville, United States: National Center for Health Statistics (NCHS), Centers for Disease Control and Prevention (CDC)
Peripheral artery disease	National Center for Health Statistics (NCHS), Centers for Disease Control and Prevention (CDC). United States National Health and Nutrition Examination Survey 2003-2004. Hyattsville, United States: National Center for Health Statistics (NCHS), Centers for Disease Control and Prevention (CDC)
Peripheral artery disease	Truven Health Analytics. United States MarketScan Claims and Medicare Data - 2012. Ann Arbor, United States: Truven Health Analytics
Peripheral artery disease	Wattanakit K, Williams JE, Schreiner PJ, Hirsch AT, Folsom AR. Association of anger proneness, depression and low social support with peripheral arterial disease: the Atherosclerosis Risk in Communities Study. Vasc Med. 2005; 10(3): 199-206
Peripheral artery disease	National Center for Health Statistics (NCHS), Centers for Disease Control and Prevention (CDC). United States National Health and Nutrition Examination Survey 1999-2004. Hyattsville, United States: National Center for Health Statistics (NCHS), Centers for Disease Control and Prevention (CDC)
Peripheral artery disease	Murabito JM, Evans JC, Larson MG, Nieto K, Levy D, Wilson PWF. The Ankle-Brachial Index in the Elderly and Risk of Stroke, Coronary Disease, and Death: The Framingham Study. Arch Intern Med. 2003; 163(16): 1939-42
Peripheral artery disease	Ostchega Y, Paulose-Ram R, Dillon CF, Gu Q, Hughes JP. Prevalence of peripheral arterial disease and risk factors in persons aged 60 and older: data from the National Health and Nutrition Examination Survey 1999-2004. J Am Geriatr Soc. 2007; 55(4): 583-9
Peripheral artery disease	Murabito JM, Evans JC, Nieto K, Larson MG, Levy D, Wilson PWF. Prevalence and clinical correlates of peripheral arterial disease in the Framingham Offspring Study. Am Heart J. 2002; 143(6): 961-5
Peripheral artery disease	Joosten MM, Pai JK, Bertoia ML, Rimm EB, Spiegelman D, Mittleman MA, Mukamal KJ. Associations between conventional cardiovascular risk factors and risk of peripheral artery disease in men. JAMA. 2012; 308(16): 1660Â–7
Rheumatic heart disease	Healthcare Cost and Utilization Project (HCUP), Agency for Healthcare Research and Quality (AHRQ). United States State Inpatient Databases 2009. Rockville, United States: Healthcare Cost and Utilization Project (HCUP), Agency for Healthcare Research and Quality (AHRQ)
Rheumatic heart disease	Healthcare Cost and Utilization Project (HCUP), Agency for Healthcare Research and Quality (AHRQ). United States State Inpatient Databases 2006. Rockville, United States: Healthcare Cost and Utilization Project (HCUP), Agency for Healthcare Research and Quality (AHRQ)

© 2018 Global Burden of Cardiovascular Diseases Collaboration. *JAMA Cardiology*.

Rheumatic heart disease	Healthcare Cost and Utilization Project (HCUP), Agency for Healthcare Research and Quality (AHRQ). United States State Inpatient Databases 2011. Rockville, United States: Healthcare Cost and Utilization Project (HCUP), Agency for Healthcare Research and Quality (AHRQ)
Rheumatic heart disease	Healthcare Cost and Utilization Project (HCUP), Agency for Healthcare Research and Quality (AHRQ). United States State Inpatient Databases 2012. Rockville, United States: Healthcare Cost and Utilization Project (HCUP), Agency for Healthcare Research and Quality (AHRQ)
Rheumatic heart disease	Healthcare Cost and Utilization Project (HCUP), Agency for Healthcare Research and Quality (AHRQ). United States State Inpatient Databases 2013. Rockville, United States: Healthcare Cost and Utilization Project (HCUP), Agency for Healthcare Research and Quality (AHRQ)
Rheumatic heart disease	Healthcare Cost and Utilization Project (HCUP), Agency for Healthcare Research and Quality (AHRQ). United States State Inpatient Databases 2014. Rockville, United States: Healthcare Cost and Utilization Project (HCUP), Agency for Healthcare Research and Quality (AHRQ)
Rheumatic heart disease	Truven Health Analytics. United States MarketScan Claims and Medicare Data - 2012. Ann Arbor, United States: Truven Health Analytics
Rheumatic heart disease	Healthcare Cost and Utilization Project (HCUP), Agency for Healthcare Research and Quality (AHRQ). United States State Inpatient Databases 2004. Rockville, United States: Healthcare Cost and Utilization Project (HCUP), Agency for Healthcare Research and Quality (AHRQ)
Rheumatic heart disease	Healthcare Cost and Utilization Project (HCUP), Agency for Healthcare Research and Quality (AHRQ). United States State Inpatient Databases 2008. Rockville, United States: Healthcare Cost and Utilization Project (HCUP), Agency for Healthcare Research and Quality (AHRQ)
Rheumatic heart disease	Healthcare Cost and Utilization Project (HCUP), Agency for Healthcare Research and Quality (AHRQ). United States State Inpatient Databases 2003. Rockville, United States: Healthcare Cost and Utilization Project (HCUP), Agency for Healthcare Research and Quality (AHRQ)
Rheumatic heart disease	Healthcare Cost and Utilization Project (HCUP), Agency for Healthcare Research and Quality (AHRQ). United States State Inpatient Databases 2005. Rockville, United States: Healthcare Cost and Utilization Project (HCUP), Agency for Healthcare Research and Quality (AHRQ)
Rheumatic heart disease	Truven Health Analytics. United States MarketScan Claims and Medicare Data - 2000. Ann Arbor, United States: Truven Health Analytics
Rheumatic heart disease	Healthcare Cost and Utilization Project (HCUP), Agency for Healthcare Research and Quality (AHRQ). United States State Inpatient Databases 2007. Rockville, United States: Healthcare Cost and Utilization Project (HCUP), Agency for Healthcare Research and Quality (AHRQ)
Rheumatic heart disease	National Center for Health Statistics (NCHS), Centers for Disease Control and Prevention (CDC), US Census Bureau. United States National Health Interview Survey 1999. Hyattsville, United States: National Center for Health Statistics (NCHS), Centers for Disease Control and Prevention (CDC)
Rheumatic heart disease	National Center for Health Statistics (NCHS), Centers for Disease Control and Prevention (CDC). United States National Health and Nutrition Examination Survey 2009-2010. Hyattsville, United States: National Center for Health Statistics (NCHS), Centers for Disease Control and Prevention (CDC), 2011
Rheumatic heart disease	National Center for Health Statistics (NCHS), Centers for Disease Control and Prevention (CDC), United States Census Bureau. United States National Hospital Discharge Survey 1996. Hyattsville, United States: National Center for Health Statistics (NCHS), Centers for Disease Control and Prevention (CDC)
Rheumatic heart disease	National Center for Health Statistics (NCHS), Centers for Disease Control and Prevention (CDC), United States Census Bureau. United States National Hospital Discharge Survey 1993. Hyattsville, United States: National Center for Health Statistics (NCHS), Centers for Disease Control and Prevention (CDC)
Rheumatic heart disease	National Center for Health Statistics (NCHS), Centers for Disease Control and Prevention (CDC), US Census Bureau. United States National Health Interview Survey 2000. Hyattsville, United States: National Center for Health Statistics (NCHS), Centers for Disease Control and Prevention (CDC)
Rheumatic heart disease	National Center for Health Statistics (NCHS), Centers for Disease Control and Prevention (CDC), US Census Bureau. United States National Health Interview Survey 1992. Hyattsville, United States: National Center for Health Statistics (NCHS), Centers for Disease Control and Prevention (CDC)
Rheumatic heart disease	Truven Health Analytics. United States MarketScan Claims and Medicare Data - 2010. Ann Arbor, United States: Truven Health Analytics
Rheumatic heart disease	National Center for Health Statistics (NCHS), Centers for Disease Control and Prevention (CDC), United States Census Bureau. United States National Hospital Discharge Survey 2000. Hyattsville, United States: National Center for Health Statistics (NCHS), Centers for Disease Control and Prevention (CDC)
Rheumatic heart disease	National Center for Health Statistics (NCHS), Centers for Disease Control and Prevention (CDC), United States Census Bureau. United States National Hospital Discharge Survey 2004. Hyattsville, United States: National Center for Health Statistics (NCHS), Centers for Disease Control and Prevention (CDC)
Rheumatic heart disease	National Center for Health Statistics (NCHS), Centers for Disease Control and Prevention (CDC), United States Census Bureau. United States National Hospital Discharge Survey 2005. Hyattsville, United States: National Center for Health Statistics (NCHS), Centers for Disease Control and Prevention (CDC)
Rheumatic heart disease	National Center for Health Statistics (NCHS), Centers for Disease Control and Prevention (CDC), United States Census Bureau. United States National Hospital Discharge Survey 1999. Hyattsville, United States: National Center for Health Statistics (NCHS), Centers for Disease Control and Prevention (CDC)
Rheumatic heart disease	National Center for Health Statistics (NCHS), Centers for Disease Control and Prevention (CDC), United States Census Bureau. United States National Hospital Discharge Survey 1981. Hyattsville, United States: National Center for Health Statistics (NCHS), Centers for Disease Control and Prevention (CDC)
Rheumatic heart disease	National Center for Health Statistics (NCHS), Centers for Disease Control and Prevention (CDC), US Census Bureau. United States National Health Interview Survey 2002. Hyattsville, United States: National Center for Health Statistics (NCHS), Centers for Disease Control and Prevention (CDC)

© 2018 Global Burden of Cardiovascular Diseases Collaboration. *JAMA Cardiology*.

Rheumatic heart disease	National Center for Health Statistics (NCHS), Centers for Disease Control and Prevention (CDC), United States Census Bureau. United States National Health Interview Survey 2012. Hyattsville, United States: National Center for Health Statistics (NCHS), Centers for Disease Control and Prevention (CDC), 2013
Rheumatic heart disease	National Center for Health Statistics (NCHS), Centers for Disease Control and Prevention (CDC). United States National Health and Nutrition Examination Survey 2001-2002. Hyattsville, United States: National Center for Health Statistics (NCHS), Centers for Disease Control and Prevention (CDC)
Rheumatic heart disease	National Center for Health Statistics (NCHS), Centers for Disease Control and Prevention (CDC), United States Census Bureau. United States National Hospital Discharge Survey 1994. Hyattsville, United States: National Center for Health Statistics (NCHS), Centers for Disease Control and Prevention (CDC)
Rheumatic heart disease	National Center for Health Statistics (NCHS), Centers for Disease Control and Prevention (CDC), United States Census Bureau. United States National Hospital Discharge Survey 1986. Hyattsville, United States: National Center for Health Statistics (NCHS), Centers for Disease Control and Prevention (CDC)
Rheumatic heart disease	National Center for Health Statistics (NCHS), Centers for Disease Control and Prevention (CDC), US Census Bureau. United States National Health Interview Survey 2009. Hyattsville, United States: National Center for Health Statistics (NCHS), Centers for Disease Control and Prevention (CDC)
Rheumatic heart disease	National Center for Health Statistics (NCHS), Centers for Disease Control and Prevention (CDC), United States Census Bureau. United States National Hospital Discharge Survey 2006. Hyattsville, United States: National Center for Health Statistics (NCHS), Centers for Disease Control and Prevention (CDC)
Rheumatic heart disease	National Center for Health Statistics (NCHS), Centers for Disease Control and Prevention (CDC), US Census Bureau. United States National Health Interview Survey 2004. Hyattsville, United States: National Center for Health Statistics (NCHS), Centers for Disease Control and Prevention (CDC)
Rheumatic heart disease	Panel Study of Income Dynamics, 2005 public use dataset. Produced and distributed by the University of Michigan with primary funding from the National Science Foundation, the National Institute of Aging, and the National Institute of Child Health and Human Development. Ann Arbor, MI, (2011)
Rheumatic heart disease	National Center for Health Statistics (NCHS), Centers for Disease Control and Prevention (CDC). United States National Health and Nutrition Examination Survey 2005-2006. Hyattsville, United States: National Center for Health Statistics (NCHS), Centers for Disease Control and Prevention (CDC), 2007
Rheumatic heart disease	National Center for Health Statistics (NCHS), Centers for Disease Control and Prevention (CDC), United States Census Bureau. United States National Hospital Discharge Survey 1987. Hyattsville, United States: National Center for Health Statistics (NCHS), Centers for Disease Control and Prevention (CDC)
Rheumatic heart disease	Washington State University, World Health Organization (WHO). United States WHO Multi-country Survey Study on Health and Health System Responsiveness 2000-2001. Geneva, Switzerland: World Health Organization (WHO)
Rheumatic heart disease	National Center for Health Statistics (NCHS), Centers for Disease Control and Prevention (CDC), US Census Bureau. United States National Health Interview Survey 1991. Hyattsville, United States: National Center for Health Statistics (NCHS), Centers for Disease Control and Prevention (CDC)
Rheumatic heart disease	National Center for Health Statistics (NCHS), Centers for Disease Control and Prevention (CDC), United States Census Bureau. United States National Hospital Discharge Survey 1988. Hyattsville, United States: National Center for Health Statistics (NCHS), Centers for Disease Control and Prevention (CDC)
Rheumatic heart disease	National Center for Health Statistics (NCHS), Centers for Disease Control and Prevention (CDC), United States Census Bureau. United States National Hospital Discharge Survey 2007. Hyattsville, United States: National Center for Health Statistics (NCHS), Centers for Disease Control and Prevention (CDC)
Rheumatic heart disease	National Center for Health Statistics (NCHS), Centers for Disease Control and Prevention (CDC), United States Census Bureau. United States National Hospital Discharge Survey 2009. Hyattsville, United States: National Center for Health Statistics (NCHS), Centers for Disease Control and Prevention (CDC)
Rheumatic heart disease	National Center for Health Statistics (NCHS), Centers for Disease Control and Prevention (CDC), US Census Bureau. United States National Health Interview Survey 2006. Hyattsville, United States: National Center for Health Statistics (NCHS), Centers for Disease Control and Prevention (CDC)
Rheumatic heart disease	National Center for Health Statistics (NCHS), Centers for Disease Control and Prevention (CDC), United States Census Bureau. United States National Hospital Discharge Survey 1997. Hyattsville, United States: National Center for Health Statistics (NCHS), Centers for Disease Control and Prevention (CDC)
Rheumatic heart disease	National Center for Health Statistics (NCHS), Centers for Disease Control and Prevention (CDC), United States Census Bureau. United States National Hospital Discharge Survey 1995. Hyattsville, United States: National Center for Health Statistics (NCHS), Centers for Disease Control and Prevention (CDC)
Rheumatic heart disease	National Center for Health Statistics (NCHS), Centers for Disease Control and Prevention (CDC), United States Census Bureau. United States National Hospital Discharge Survey 1979. Hyattsville, United States: National Center for Health Statistics (NCHS), Centers for Disease Control and Prevention (CDC)
Rheumatic heart disease	National Center for Health Statistics (NCHS) Centers for Disease Control and Prevention (CDC). United States National Health and Nutrition Examination Survey 2011-2012. Hyattsville, United States: National Center for Health Statistics (NCHS) Centers for Disease Control and Prevention (CDC), 2013
Rheumatic heart disease	Healthcare Cost and Utilization Project (HCUP), Agency for Healthcare Research and Quality (AHRQ). United States Nationwide Inpatient Sample 2013. Rockville, United States: Healthcare Cost and Utilization Project (HCUP), Agency for Healthcare Research and Quality (AHRQ)
Rheumatic heart disease	National Center for Health Statistics (NCHS), Centers for Disease Control and Prevention (CDC), US Census Bureau. United States National Health Interview Survey 2003. Hyattsville, United States: National Center for Health Statistics (NCHS), Centers for Disease Control and Prevention (CDC)
Rheumatic heart disease	National Center for Health Statistics (NCHS), Centers for Disease Control and Prevention (CDC), United States Census Bureau. United States National Hospital Discharge Survey 2010. Hyattsville, United States: National Center for Health Statistics (NCHS), Centers for Disease Control and Prevention (CDC)

© 2018 Global Burden of Cardiovascular Diseases Collaboration. *JAMA Cardiology*.

Rheumatic heart disease	National Center for Health Statistics (NCHS), Centers for Disease Control and Prevention (CDC), United States Census Bureau. United States National Hospital Discharge Survey 1982. Hyattsville, United States: National Center for Health Statistics (NCHS), Centers for Disease Control and Prevention (CDC)
Rheumatic heart disease	National Center for Health Statistics (NCHS), Centers for Disease Control and Prevention (CDC), US Census Bureau. United States National Health Interview Survey 2011. Hyattsville, United States: National Center for Health Statistics (NCHS), Centers for Disease Control and Prevention (CDC)
Rheumatic heart disease	National Center for Health Statistics (NCHS), Centers for Disease Control and Prevention (CDC), US Census Bureau. United States National Health Interview Survey 1998. Hyattsville, United States: National Center for Health Statistics (NCHS), Centers for Disease Control and Prevention (CDC)
Rheumatic heart disease	National Center for Health Statistics (NCHS), Centers for Disease Control and Prevention (CDC). United States National Health and Nutrition Examination Survey 1999-2000. Hyattsville, United States: National Center for Health Statistics (NCHS), Centers for Disease Control and Prevention (CDC)
Rheumatic heart disease	Healthcare Cost and Utilization Project (HCUP), Agency for Healthcare Research and Quality (AHRQ). United States Nationwide Inpatient Sample 2014. Rockville, United States: Healthcare Cost and Utilization Project (HCUP), Agency for Healthcare Research and Quality (AHRQ)
Rheumatic heart disease	United States Department of Health and Human Services. Centers for Disease Control and Prevention. National Center for Health Statistics. National Health Interview Survey, 1994: Second Longitudinal Study on Aging, Wave 2, 1997. ICPSR03526-v2. Ann Arbor, MI: Inter-university Consortium for Political and Social Research [distributor], 2007-03-01. http://doi.org/10.3886/ICPSR03526.v2
Rheumatic heart disease	National Center for Health Statistics (NCHS), Centers for Disease Control and Prevention (CDC), US Census Bureau. United States National Health Interview Survey 2001. Hyattsville, United States: National Center for Health Statistics (NCHS), Centers for Disease Control and Prevention (CDC)
Rheumatic heart disease	National Center for Health Statistics (NCHS), Centers for Disease Control and Prevention (CDC), United States Census Bureau. United States National Hospital Discharge Survey 2008. Hyattsville, United States: National Center for Health Statistics (NCHS), Centers for Disease Control and Prevention (CDC)
Rheumatic heart disease	National Center for Health Statistics (NCHS), Centers for Disease Control and Prevention (CDC), United States Census Bureau. United States National Hospital Discharge Survey 1998. Hyattsville, United States: National Center for Health Statistics (NCHS), Centers for Disease Control and Prevention (CDC)
Rheumatic heart disease	National Center for Health Statistics (NCHS), Centers for Disease Control and Prevention (CDC), United States Census Bureau. United States National Hospital Discharge Survey 1985. Hyattsville, United States: National Center for Health Statistics (NCHS), Centers for Disease Control and Prevention (CDC)
Rheumatic heart disease	National Center for Health Statistics (NCHS), Centers for Disease Control and Prevention (CDC). United States National Health and Nutrition Examination Survey 2003-2004. Hyattsville, United States: National Center for Health Statistics (NCHS), Centers for Disease Control and Prevention (CDC)
Rheumatic heart disease	Healthcare Cost and Utilization Project (HCUP), Agency for Healthcare Research and Quality (AHRQ). United States Nationwide Inpatient Sample 2015. Rockville, United States: Healthcare Cost and Utilization Project (HCUP), Agency for Healthcare Research and Quality (AHRQ)
Rheumatic heart disease	National Center for Health Statistics (NCHS), Centers for Disease Control and Prevention (CDC), United States Census Bureau. United States National Hospital Discharge Survey 2001. Hyattsville, United States: National Center for Health Statistics (NCHS), Centers for Disease Control and Prevention (CDC)
Rheumatic heart disease	National Center for Health Statistics (NCHS), Centers for Disease Control and Prevention (CDC), United States Census Bureau. United States National Hospital Discharge Survey 1980. Hyattsville, United States: National Center for Health Statistics (NCHS), Centers for Disease Control and Prevention (CDC)
Rheumatic heart disease	National Center for Health Statistics (NCHS), Centers for Disease Control and Prevention (CDC). United States National Health Interview Survey 2014. Hyattsville, United States: National Center for Health Statistics (NCHS), Centers for Disease Control and Prevention (CDC), 2015
Rheumatic heart disease	National Center for Health Statistics (NCHS), Centers for Disease Control and Prevention (CDC), United States Census Bureau. United States National Hospital Discharge Survey 2003. Hyattsville, United States: National Center for Health Statistics (NCHS), Centers for Disease Control and Prevention (CDC)
Rheumatic heart disease	National Center for Health Statistics (NCHS), Centers for Disease Control and Prevention (CDC), US Census Bureau. United States National Health Interview Survey 2007. Hyattsville, United States: National Center for Health Statistics (NCHS), Centers for Disease Control and Prevention (CDC)
Rheumatic heart disease	National Center for Health Statistics (NCHS), Centers for Disease Control and Prevention (CDC), United States Census Bureau. United States National Hospital Discharge Survey 1983. Hyattsville, United States: National Center for Health Statistics (NCHS), Centers for Disease Control and Prevention (CDC)
Rheumatic heart disease	National Center for Health Statistics (NCHS), Centers for Disease Control and Prevention (CDC), United States Census Bureau. United States National Hospital Discharge Survey 1989. Hyattsville, United States: National Center for Health Statistics (NCHS), Centers for Disease Control and Prevention (CDC)
Rheumatic heart disease	National Center for Health Statistics (NCHS), Centers for Disease Control and Prevention (CDC), US Census Bureau. United States National Health Interview Survey 1993. Hyattville, United States: National Center for Health Statistics (NCHS), Centers for Disease Control and Prevention (CDC)
Rheumatic heart disease	National Center for Health Statistics (NCHS), Centers for Disease Control and Prevention (CDC). United States National Health and Nutrition Examination Survey 2013-2014. Hyattsville, United States: National Center for Health Statistics (NCHS), Centers for Disease Control and Prevention (CDC)
Rheumatic heart disease	National Center for Health Statistics (NCHS), Centers for Disease Control and Prevention (CDC), United States Census Bureau. United States National Hospital Discharge Survey 1984. Hyattsville, United States: National Center for Health Statistics (NCHS), Centers for Disease Control and Prevention (CDC)

© 2018 Global Burden of Cardiovascular Diseases Collaboration. *JAMA Cardiology*.

Rheumatic heart disease	National Center for Health Statistics (NCHS), Centers for Disease Control and Prevention (CDC), United States Census Bureau. United States National Hospital Discharge Survey 2002. Hyattsville, United States: National Center for Health Statistics (NCHS), Centers for Disease Control and Prevention (CDC)
Rheumatic heart disease	National Center for Health Statistics (NCHS), Centers for Disease Control and Prevention (CDC). United States National Health and Nutrition Examination Survey 2007-2008. Hyattsville, United States: National Center for Health Statistics (NCHS), Centers for Disease Control and Prevention (CDC), 2009
Rheumatic heart disease	National Center for Health Statistics (NCHS), Centers for Disease Control and Prevention (CDC), United States Census Bureau. United States National Health Interview Survey 2013. Hyattsville, United States: National Center for Health Statistics (NCHS), Centers for Disease Control and Prevention (CDC), 2014
Rheumatic heart disease	National Center for Health Statistics (NCHS), Centers for Disease Control and Prevention (CDC), US Census Bureau. United States National Health Interview Survey 1990. Hyattsville, United States: National Center for Health Statistics (NCHS), Centers for Disease Control and Prevention (CDC)
Rheumatic heart disease	Panel Study of Income Dynamics, 2007 public use dataset. Produced and distributed by the University of Michigan with primary funding from the National Science Foundation, the National Institute of Aging, and the National Institute of Child Health and Human Development. Ann Arbor, MI, (2011)
Rheumatic heart disease	National Center for Health Statistics (NCHS), Centers for Disease Control and Prevention (CDC). United States National Health and Nutrition Examination Survey 1988-1994. Hyattsville, United States: National Center for Health Statistics (NCHS), Centers for Disease Control and Prevention (CDC)
Rheumatic heart disease	United States Department of Health and Human Services. Centers for Disease Control and Prevention. National Center for Health Statistics. National Health Interview Survey, 1994: Second Supplement on Aging. ICPSR02563-v3. Ann Arbor, MI: Inter-university Consortium for Political and Social Research [distributor], 2007-02-12. http://doi.org/10.3886/ICPSR02563.v3
Rheumatic heart disease	National Center for Health Statistics (NCHS), Centers for Disease Control and Prevention (CDC), US Census Bureau. United States National Health Interview Survey 2008. Hyattsville, United States: National Center for Health Statistics (NCHS), Centers for Disease Control and Prevention (CDC)
Rheumatic heart disease	National Center for Health Statistics (NCHS), Centers for Disease Control and Prevention (CDC), United States Census Bureau. United States National Hospital Discharge Survey 1990. Hyattsville, United States: National Center for Health Statistics (NCHS), Centers for Disease Control and Prevention (CDC)
Rheumatic heart disease	National Center for Health Statistics (NCHS), Centers for Disease Control and Prevention (CDC), US Census Bureau. United States National Health Interview Survey 2010. Hyattsville, United States: National Center for Health Statistics (NCHS), Centers for Disease Control and Prevention (CDC)
Rheumatic heart disease	National Center for Health Statistics (NCHS), Centers for Disease Control and Prevention (CDC), US Census Bureau. United States National Health Interview Survey 1997. Hyattsville, United States: National Center for Health Statistics (NCHS), Centers for Disease Control and Prevention (CDC)
Rheumatic heart disease	National Center for Health Statistics (NCHS), Centers for Disease Control and Prevention (CDC), United States Census Bureau. United States National Hospital Discharge Survey 1991. Hyattsville, United States: National Center for Health Statistics (NCHS), Centers for Disease Control and Prevention (CDC)
Rheumatic heart disease	National Center for Health Statistics (NCHS), Centers for Disease Control and Prevention (CDC), United States Census Bureau. United States National Hospital Discharge Survey 1992. Hyattsville, United States: National Center for Health Statistics (NCHS), Centers for Disease Control and Prevention (CDC)

© 2018 Global Burden of Cardiovascular Diseases Collaboration. *JAMA Cardiology*.

eTable 1. Disability-adjusted life-years (DALYs) and percentage change of DALYs for all cardiovascular causes by US state, total number and age-standardized rate for 1990, 2006, and 2016 for both sexes

	Number of DALYS (95% UI)			Percentage change in DALYs (95% UI)		Age-standardized DALY rates per 100,000 persons (95% UI)			Percentage change in DALY rates (95% UI)	
	1990	2006	2016	1990 to 2016	2006 to 2016	1990	2006	2016	1990 to 2016	2006 to 2016
**Alcoholic cardiomyopathy**										
**Alabama**	1,567 (1,098 to 1,985)	2,054 (1,717 to 3,050)	2,418 (1,917 to 3,636)	.56 (.17 to 1.48)	.18 (-.02 to .42)	36 (25 to 45)	36 (30 to 54)	38 (30 to 57)	.07 (-.2 to .67)	.04 (-.13 to .25)
**Alaska**	193 (122 to 230)	281 (206 to 374)	370 (266 to 488)	.95 (.49 to 1.71)	.32 (.08 to .62)	48 (31 to 58)	40 (29 to 52)	44 (31 to 58)	-.08 (-.3 to .26)	.11 (-.1 to .35)
**Arizona**	1,113 (893 to 1,420)	2,234 (1,835 to 3,287)	2,641 (2,118 to 4,230)	1.39 (.85 to 2.81)	.18 (-.02 to .4)	28 (23 to 36)	32 (26 to 46)	30 (24 to 46)	.06 (-.17 to .61)	-.07 (-.22 to .1)
**Arkansas**	844 (702 to 1,128)	1,121 (925 to 1,747)	1,300 (1,009 to 2,241)	.54 (.18 to 1.3)	.15 (-.03 to .39)	32 (26 to 42)	32 (27 to 51)	34 (26 to 59)	.06 (-.19 to .59)	.03 (-.14 to .25)
**California**	13,915 (6,351 to 18,037)	15,691 (9,658 to 18,450)	18,886 (11,218 to 23,783)	.42 (.04 to 1.52)	.21 (-.01 to .45)	48 (22 to 62)	39 (24 to 46)	38 (23 to 48)	-.17 (-.4 to .49)	-.03 (-.2 to .16)
**Colorado**	944 (738 to 1,225)	1,483 (1,200 to 2,399)	1,933 (1,502 to 3,420)	1.05 (.58 to 2.06)	.3 (.08 to .53)	29 (23 to 37)	27 (22 to 44)	28 (22 to 48)	-.03 (-.25 to .42)	.02 (-.14 to .19)
**Connecticut**	1,233 (861 to 1,464)	1,390 (1,052 to 1,738)	1,430 (1,124 to 2,072)	.18 (-.12 to .92)	.03 (-.16 to .27)	33 (23 to 39)	31 (23 to 39)	29 (22 to 41)	-.11 (-.33 to .41)	-.06 (-.23 to .13)
**Delaware**	330 (185 to 401)	433 (295 to 510)	496 (366 to 598)	.54 (.2 to 1.24)	.15 (-.04 to .38)	46 (25 to 56)	40 (28 to 48)	37 (28 to 45)	-.16 (-.34 to .19)	-.06 (-.22 to .11)
**District of Columbia**	612 (201 to 883)	441 (234 to 582)	428 (259 to 550)	-.25 (-.49 to .45)	-.02 (-.21 to .24)	94 (30 to 134)	67 (36 to 89)	57 (34 to 73)	-.35 (-.56 to .27)	-.15 (-.31 to .07)

© 2018 Global Burden of Cardiovascular Diseases Collaboration. *JAMA Cardiology*.

**Florida**	6,955 (3,459 to 8,650)	9,864 (6,314 to 11,596)	12,041 (7,937 to 14,778)	.78 (.37 to 1.72)	.23 (.03 to .45)	43 (22 to 53)	41 (27 to 48)	40 (27 to 48)	-.05 (-.27 to .49)	-.03 (-.18 to .16)
**Georgia**	2,331 (1,408 to 2,961)	4,833 (3,203 to 5,806)	6,067 (4,095 to 7,591)	1.67 (.7 to 3.15)	.26 (.02 to .55)	37 (22 to 47)	48 (31 to 58)	46 (32 to 58)	.29 (-.14 to 1.04)	-.03 (-.23 to .2)
**Hawaii**	615 (260 to 796)	755 (393 to 937)	885 (475 to 1,117)	.49 (.16 to 1.07)	.18 (-.01 to .37)	53 (22 to 68)	46 (24 to 57)	47 (25 to 59)	-.08 (-.29 to .26)	.03 (-.14 to .19)
**Idaho**	279 (222 to 374)	409 (325 to 687)	553 (417 to 953)	.98 (.49 to 1.86)	.35 (.13 to .6)	27 (22 to 37)	24 (19 to 41)	26 (20 to 44)	-.04 (-.27 to .38)	.07 (-.11 to .26)
**Illinois**	4,672 (2,574 to 5,780)	4,782 (3,889 to 6,133)	5,653 (4,476 to 7,559)	.26 (-.08 to 1.48)	.18 (0 to .43)	39 (21 to 48)	32 (26 to 41)	34 (27 to 45)	-.09 (-.33 to .8)	.05 (-.11 to .26)
**Indiana**	2,329 (1,395 to 2,820)	2,821 (2,106 to 3,384)	2,985 (2,375 to 4,147)	.32 (-.03 to 1.2)	.06 (-.14 to .33)	39 (23 to 47)	38 (28 to 45)	35 (28 to 49)	-.09 (-.34 to .5)	-.08 (-.25 to .16)
**Iowa**	975 (730 to 1,161)	1,080 (848 to 1,676)	1,259 (975 to 2,023)	.3 (-.01 to 1.05)	.17 (-.01 to .37)	31 (23 to 37)	29 (23 to 45)	30 (23 to 48)	-.01 (-.23 to .55)	.06 (-.1 to .24)
**Kansas**	902 (614 to 1,076)	1,115 (842 to 1,436)	1,225 (966 to 1,805)	.38 (.02 to 1.16)	.1 (-.1 to .34)	33 (23 to 40)	34 (26 to 44)	33 (26 to 49)	0 (-.26 to .56)	-.02 (-.21 to .19)
**Kentucky**	1,204 (963 to 1,570)	1,663 (1,374 to 2,576)	1,910 (1,487 to 3,180)	.59 (.21 to 1.44)	.14 (-.04 to .34)	30 (24 to 40)	32 (26 to 50)	33 (25 to 54)	.09 (-.17 to .65)	.01 (-.15 to .19)
**Louisiana**	1,598 (1,053 to 1,980)	1,743 (1,423 to 2,741)	2,251 (1,821 to 3,373)	.43 (.08 to 1.13)	.3 (.12 to .51)	38 (25 to 47)	34 (28 to 54)	38 (30 to 57)	.02 (-.23 to .52)	.1 (-.05 to .28)
**Maine**	501 (327 to 590)	573 (448 to 728)	686 (519 to 917)	.39 (.08 to 1.14)	.2 (.02 to .4)	36 (24 to 43)	32 (25 to 40)	33 (25 to 45)	-.07 (-.28 to .38)	.04 (-.11 to .21)
**Maryland**	2,063 (1,093 to 2,600)	2,675 (2,169 to 3,321)	3,250 (2,483 to 3,997)	.64 (.2 to 2.09)	.22 (.04 to .42)	41 (22 to 52)	40 (32 to 49)	40 (31 to 50)	.03 (-.24 to .94)	0 (-.13 to .16)
**Massachusetts**	1,731 (1,394 to 2,501)	2,086 (1,689 to 3,434)	2,409 (1,839 to 4,341)	.4 (.05 to 1.5)	.15 (-.03 to .38)	26 (21 to 38)	27 (21 to 43)	26 (20 to 46)	0 (-.25 to .72)	-.03 (-.19 to .16)

© 2018 Global Burden of Cardiovascular Diseases Collaboration. *JAMA Cardiology*.

**Michigan**	3,556 (2,223 to 4,345)	5,287 (3,571 to 6,206)	5,589 (4,117 to 7,022)	.6 (.23 to 1.37)	.06 (-.13 to .31)	37 (23 to 45)	43 (29 to 50)	41 (30 to 50)	.13 (-.12 to .66)	-.05 (-.22 to .15)
**Minnesota**	1,184 (957 to 1,563)	1,684 (1,191 to 2,794)	2,144 (1,493 to 3,616)	.81 (.34 to 1.74)	.27 (.08 to .5)	26 (21 to 34)	28 (19 to 45)	29 (20 to 48)	.11 (-.18 to .66)	.05 (-.11 to .22)
**Mississippi**	1,124 (694 to 1,366)	1,502 (1,245 to 2,056)	1,699 (1,342 to 2,415)	.55 (.14 to 1.45)	.13 (-.07 to .36)	42 (26 to 51)	44 (36 to 61)	45 (35 to 65)	.09 (-.19 to .71)	.02 (-.16 to .23)
**Missouri**	2,002 (1,346 to 2,394)	2,266 (1,910 to 3,191)	2,272 (1,783 to 3,780)	.16 (-.17 to 1.03)	0 (-.19 to .26)	36 (24 to 43)	32 (27 to 45)	28 (22 to 47)	-.18 (-.41 to .42)	-.1 (-.27 to .13)
**Montana**	258 (201 to 319)	313 (251 to 511)	379 (285 to 677)	.47 (.09 to 1.38)	.21 (-.02 to .46)	29 (23 to 36)	25 (20 to 41)	26 (19 to 45)	-.11 (-.35 to .39)	.03 (-.17 to .23)
**Nebraska**	483 (397 to 629)	538 (435 to 903)	623 (482 to 1,071)	.29 (-.01 to 1.01)	.15 (-.02 to .34)	28 (23 to 36)	25 (21 to 43)	26 (20 to 44)	-.08 (-.3 to .42)	.01 (-.15 to .17)
**Nevada**	518 (333 to 666)	1,175 (910 to 1,436)	1,779 (1,263 to 2,166)	2.5 (1.41 to 4.21)	.52 (.19 to .86)	40 (26 to 51)	40 (31 to 48)	47 (32 to 56)	.19 (-.18 to .74)	.18 (-.08 to .42)
**New Hampshire**	379 (291 to 475)	463 (383 to 701)	570 (455 to 891)	.51 (.16 to 1.25)	.23 (.05 to .43)	33 (25 to 41)	28 (23 to 42)	29 (23 to 44)	-.12 (-.32 to .29)	.05 (-.11 to .21)
**New Jersey**	4,058 (2,216 to 4,979)	4,786 (2,840 to 5,654)	4,450 (3,087 to 5,571)	.13 (-.14 to 1.03)	-.06 (-.24 to .17)	47 (25 to 57)	45 (27 to 53)	36 (25 to 45)	-.19 (-.39 to .45)	-.18 (-.34 to .03)
**New Mexico**	526 (370 to 628)	792 (652 to 1,161)	877 (683 to 1,411)	.69 (.24 to 1.75)	.11 (-.1 to .37)	35 (25 to 42)	34 (28 to 48)	33 (26 to 51)	-.04 (-.3 to .48)	-.02 (-.2 to .2)
**New York**	6,511 (4,907 to 8,600)	6,689 (5,408 to 10,422)	7,080 (5,325 to 12,409)	.1 (-.22 to 1.06)	.05 (-.14 to .28)	33 (25 to 43)	29 (23 to 45)	27 (20 to 47)	-.17 (-.42 to .53)	-.07 (-.25 to .12)
**North Carolina**	3,256 (1,636 to 4,031)	4,743 (2,925 to 5,572)	5,895 (3,893 to 7,178)	.87 (.44 to 2.22)	.25 (.05 to .58)	46 (23 to 57)	44 (27 to 51)	43 (29 to 52)	-.03 (-.26 to .7)	-.02 (-.18 to .25)
**North Dakota**	195 (154 to 266)	190 (152 to 328)	242 (182 to 421)	.24 (-.08 to .83)	.27 (.03 to .53)	28 (22 to 38)	24 (19 to 41)	26 (20 to 45)	-.07 (-.31 to .37)	.12 (-.1 to .34)

© 2018 Global Burden of Cardiovascular Diseases Collaboration. *JAMA Cardiology*.

**Ohio**	2,958 (2,181 to 4,457)	4,361 (3,682 to 6,044)	5,781 (4,579 to 7,565)	.99 (.38 to 1.68)	.33 (.11 to .58)	25 (18 to 37)	31 (26 to 43)	37 (29 to 48)	.51 (.07 to 1.03)	.21 (.01 to .43)
**Oklahoma**	1,033 (843 to 1,359)	1,273 (1,018 to 2,149)	1,707 (1,304 to 2,996)	.65 (.25 to 1.69)	.34 (.13 to .6)	30 (24 to 40)	29 (24 to 50)	35 (26 to 61)	.15 (-.13 to .84)	.17 (0 to .39)
**Oregon**	1,028 (785 to 1,229)	1,483 (1,128 to 2,195)	2,043 (1,355 to 3,184)	1 (.48 to 2.02)	.38 (.15 to .62)	32 (25 to 38)	32 (24 to 47)	35 (24 to 55)	.11 (-.17 to .65)	.1 (-.07 to .28)
**Pennsylvania**	3,840 (2,567 to 5,435)	4,185 (3,416 to 6,266)	5,218 (4,204 to 7,441)	.38 (.07 to .99)	.25 (.06 to .52)	27 (19 to 38)	26 (21 to 39)	28 (23 to 42)	.06 (-.18 to .51)	.09 (-.08 to .3)
**Rhode Island**	350 (278 to 468)	340 (271 to 566)	375 (282 to 650)	.07 (-.22 to .66)	.1 (-.08 to .32)	30 (24 to 41)	25 (20 to 42)	26 (19 to 44)	-.16 (-.38 to .27)	.01 (-.15 to .21)
**South Carolina**	1,472 (871 to 1,793)	2,237 (1,665 to 2,654)	2,620 (2,075 to 3,304)	.82 (.38 to 1.83)	.18 (-.04 to .42)	41 (24 to 49)	41 (31 to 49)	39 (31 to 50)	-.02 (-.25 to .5)	-.06 (-.23 to .14)
**South Dakota**	193 (157 to 287)	211 (161 to 393)	267 (193 to 517)	.37 (.02 to 1.06)	.26 (.05 to .51)	25 (21 to 38)	22 (17 to 41)	24 (17 to 46)	-.07 (-.3 to .38)	.1 (-.09 to .3)
**Tennessee**	2,157 (1,370 to 2,558)	3,290 (2,575 to 4,269)	4,787 (3,326 to 6,388)	1.27 (.72 to 2.76)	.46 (.19 to .71)	40 (26 to 48)	44 (34 to 56)	54 (38 to 71)	.36 (.05 to 1.25)	.23 (.02 to .43)
**Texas**	4,453 (3,305 to 5,968)	6,377 (5,237 to 10,016)	7,350 (5,498 to 13,596)	.66 (.19 to 1.97)	.14 (-.09 to .45)	27 (20 to 36)	26 (21 to 40)	22 (17 to 42)	-.18 (-.42 to .5)	-.15 (-.32 to .11)
**Utah**	356 (285 to 466)	502 (397 to 845)	703 (546 to 1,232)	.97 (.5 to 1.9)	.4 (.2 to .64)	25 (20 to 33)	21 (17 to 36)	23 (18 to 40)	-.09 (-.31 to .34)	.06 (-.09 to .24)
**Vermont**	205 (141 to 245)	222 (178 to 318)	260 (206 to 388)	.29 (-.02 to .97)	.17 (-.01 to .38)	35 (24 to 42)	27 (22 to 38)	28 (22 to 41)	-.19 (-.38 to .22)	.03 (-.13 to .21)
**Virginia**	2,334 (1,506 to 2,813)	3,188 (2,496 to 3,868)	4,307 (2,914 to 5,450)	.88 (.36 to 1.89)	.35 (.1 to .62)	37 (23 to 44)	34 (27 to 42)	38 (26 to 48)	.06 (-.22 to .68)	.11 (-.08 to .32)
**Washington**	1,882 (1,227 to 2,248)	2,757 (1,911 to 3,564)	3,559 (2,369 to 4,614)	.91 (.48 to 1.76)	.29 (.11 to .51)	38 (24 to 45)	36 (25 to 47)	37 (25 to 48)	-.01 (-.23 to .46)	.02 (-.12 to .2)

© 2018 Global Burden of Cardiovascular Diseases Collaboration. *JAMA Cardiology*.

**West Virginia**	827 (569 to 979)	996 (803 to 1,315)	1,106 (895 to 1,532)	.36 (.04 to 1.09)	.11 (-.07 to .3)	39 (27 to 46)	40 (33 to 53)	42 (34 to 60)	.11 (-.17 to .66)	.05 (-.12 to .23)
**Wisconsin**	1,713 (1,191 to 2,019)	2,679 (1,658 to 3,346)	2,990 (1,936 to 4,194)	.76 (.32 to 1.74)	.12 (-.06 to .34)	33 (23 to 38)	39 (24 to 49)	38 (25 to 52)	.16 (-.12 to .79)	-.03 (-.18 to .16)
**Wyoming**	118 (97 to 169)	153 (115 to 272)	190 (135 to 356)	.59 (.18 to 1.37)	.24 (.03 to .49)	26 (22 to 38)	24 (18 to 42)	25 (18 to 47)	-.06 (-.3 to .38)	.06 (-.12 to .27)
**Aortic aneurysm**										
**Alabama**	4,799 (4,416 to 5,212)	4,011 (3,721 to 4,344)	4,045 (3,502 to 4,598)	-.16 (-.28 to -.02)	.01 (-.13 to .16)	100 (92 to 109)	69 (64 to 75)	60 (52 to 68)	-.4 (-.49 to -.31)	-.13 (-.25 to 0)
**Alaska**	297 (272 to 325)	368 (336 to 402)	427 (365 to 494)	.44 (.21 to .71)	.16 (-.02 to .36)	102 (93 to 112)	62 (57 to 68)	54 (47 to 62)	-.47 (-.55 to -.37)	-.13 (-.26 to .02)
**Arizona**	3,676 (3,382 to 3,963)	3,922 (3,637 to 4,234)	4,192 (3,712 to 4,732)	.14 (0 to .31)	.07 (-.06 to .21)	86 (79 to 92)	53 (50 to 58)	43 (38 to 49)	-.49 (-.56 to -.42)	-.19 (-.29 to -.08)
**Arkansas**	2,978 (2,742 to 3,237)	2,637 (2,440 to 2,848)	2,622 (2,331 to 2,948)	-.12 (-.23 to .01)	0 (-.12 to .13)	98 (90 to 106)	73 (67 to 78)	63 (56 to 71)	-.35 (-.43 to -.25)	-.13 (-.23 to -.01)
**California**	27,291 (25,331 to 29,624)	21,726 (20,201 to 23,448)	21,530 (19,000 to 24,416)	-.21 (-.32 to -.09)	-.01 (-.13 to .13)	92 (85 to 99)	54 (50 to 58)	43 (38 to 49)	-.53 (-.6 to -.46)	-.21 (-.31 to -.1)
**Colorado**	3,271 (3,022 to 3,533)	3,254 (3,023 to 3,474)	3,693 (3,291 to 4,132)	.13 (-.01 to .28)	.14 (0 to .28)	101 (94 to 110)	63 (59 to 67)	52 (47 to 59)	-.48 (-.55 to -.41)	-.17 (-.27 to -.06)
**Connecticut**	3,623 (3,344 to 3,905)	2,500 (2,308 to 2,699)	2,223 (1,969 to 2,539)	-.39 (-.47 to -.28)	-.11 (-.23 to .02)	88 (82 to 95)	53 (49 to 57)	42 (37 to 48)	-.52 (-.59 to -.44)	-.2 (-.31 to -.07)
**Delaware**	836 (770 to 907)	727 (674 to 782)	761 (683 to 844)	-.09 (-.2 to .04)	.05 (-.07 to .17)	111 (102 to 120)	65 (61 to 70)	54 (48 to 59)	-.51 (-.57 to -.45)	-.18 (-.27 to -.09)
**District of Columbia**	741 (648 to 878)	421 (372 to 495)	355 (300 to 426)	-.52 (-.59 to -.44)	-.16 (-.28 to -.04)	107 (94 to 127)	64 (56 to 75)	47 (39 to 56)	-.56 (-.63 to -.49)	-.27 (-.37 to -.17)

© 2018 Global Burden of Cardiovascular Diseases Collaboration. *JAMA Cardiology*.

**Florida**	18,486 (17,030 to 19,982)	15,746 (14,608 to 16,905)	15,633 (13,889 to 17,588)	-.15 (-.26 to -.03)	-.01 (-.13 to .12)	94 (86 to 102)	60 (56 to 64)	48 (42 to 55)	-.49 (-.55 to -.41)	-.19 (-.3 to -.09)
**Georgia**	6,012 (5,532 to 6,565)	6,072 (5,588 to 6,647)	6,442 (5,588 to 7,450)	.07 (-.08 to .25)	.06 (-.08 to .23)	94 (87 to 103)	62 (58 to 68)	50 (44 to 58)	-.47 (-.54 to -.38)	-.19 (-.3 to -.07)
**Hawaii**	1,124 (1,038 to 1,216)	1,077 (1,002 to 1,156)	1,125 (1,017 to 1,248)	0 (-.11 to .14)	.05 (-.06 to .17)	95 (88 to 103)	62 (58 to 67)	54 (49 to 60)	-.43 (-.5 to -.36)	-.14 (-.23 to -.03)
**Idaho**	1,163 (1,069 to 1,263)	1,110 (1,015 to 1,200)	1,230 (1,079 to 1,408)	.06 (-.1 to .23)	.11 (-.05 to .29)	104 (95 to 113)	65 (59 to 70)	55 (48 to 63)	-.47 (-.55 to -.38)	-.16 (-.28 to -.02)
**Illinois**	12,406 (11,506 to 13,353)	9,098 (8,460 to 9,785)	8,658 (7,804 to 9,647)	-.3 (-.38 to -.21)	-.05 (-.15 to .07)	94 (87 to 101)	60 (56 to 65)	50 (45 to 56)	-.47 (-.53 to -.4)	-.17 (-.26 to -.07)
**Indiana**	6,732 (6,237 to 7,288)	5,498 (5,101 to 5,937)	5,547 (4,828 to 6,341)	-.17 (-.3 to -.05)	.01 (-.14 to .16)	104 (97 to 113)	71 (66 to 77)	62 (54 to 71)	-.41 (-.49 to -.31)	-.13 (-.26 to 0)
**Iowa**	3,536 (3,266 to 3,811)	2,520 (2,330 to 2,716)	2,437 (2,173 to 2,706)	-.31 (-.4 to -.21)	-.03 (-.14 to .09)	93 (86 to 101)	61 (56 to 66)	53 (47 to 60)	-.43 (-.5 to -.35)	-.13 (-.23 to -.01)
**Kansas**	2,910 (2,676 to 3,152)	2,217 (2,044 to 2,389)	2,156 (1,871 to 2,445)	-.26 (-.37 to -.13)	-.03 (-.16 to .12)	94 (87 to 102)	64 (59 to 69)	55 (47 to 62)	-.42 (-.51 to -.32)	-.14 (-.27 to 0)
**Kentucky**	4,515 (4,170 to 4,840)	3,731 (3,478 to 4,024)	3,804 (3,405 to 4,214)	-.16 (-.26 to -.05)	.02 (-.1 to .15)	105 (97 to 112)	71 (66 to 77)	62 (55 to 69)	-.41 (-.48 to -.33)	-.13 (-.23 to -.02)
**Louisiana**	4,236 (3,895 to 4,610)	3,310 (3,051 to 3,608)	3,373 (3,043 to 3,818)	-.2 (-.29 to -.1)	.02 (-.09 to .14)	96 (88 to 105)	65 (60 to 71)	55 (50 to 63)	-.42 (-.49 to -.35)	-.15 (-.24 to -.05)
**Maine**	1,757 (1,627 to 1,904)	1,293 (1,191 to 1,397)	1,258 (1,136 to 1,400)	-.28 (-.37 to -.18)	-.03 (-.14 to .1)	116 (108 to 126)	68 (63 to 74)	57 (51 to 63)	-.51 (-.57 to -.45)	-.16 (-.26 to -.06)
**Maryland**	4,778 (4,390 to 5,215)	3,638 (3,381 to 3,974)	3,529 (3,151 to 3,976)	-.26 (-.35 to -.17)	-.03 (-.13 to .08)	95 (88 to 104)	54 (50 to 60)	44 (39 to 49)	-.54 (-.6 to -.49)	-.2 (-.28 to -.1)
**Massachusetts**	6,894 (6,358 to 7,421)	4,426 (4,107 to 4,764)	4,041 (3,647 to 4,462)	-.41 (-.49 to -.33)	-.09 (-.18 to .02)	92 (85 to 99)	52 (48 to 56)	41 (37 to 46)	-.55 (-.61 to -.49)	-.2 (-.29 to -.11)

© 2018 Global Burden of Cardiovascular Diseases Collaboration. *JAMA Cardiology*.

**Michigan**	10,544 (9,776 to 11,356)	8,731 (8,187 to 9,364)	8,266 (7,511 to 9,143)	-.22 (-.3 to -.12)	-.05 (-.15 to .05)	102 (94 to 109)	69 (65 to 74)	58 (53 to 64)	-.43 (-.49 to -.36)	-.16 (-.25 to -.07)
**Minnesota**	5,224 (4,805 to 5,673)	4,017 (3,688 to 4,347)	4,058 (3,610 to 4,515)	-.22 (-.32 to -.11)	.01 (-.11 to .14)	103 (95 to 112)	63 (58 to 68)	52 (46 to 58)	-.49 (-.56 to -.42)	-.17 (-.27 to -.06)
**Mississippi**	2,641 (2,437 to 2,873)	2,414 (2,217 to 2,635)	2,431 (2,121 to 2,808)	-.08 (-.22 to .07)	.01 (-.14 to .16)	91 (84 to 99)	70 (64 to 76)	61 (53 to 71)	-.33 (-.43 to -.22)	-.13 (-.26 to 0)
**Missouri**	6,313 (5,841 to 6,775)	4,711 (4,418 to 5,040)	4,661 (4,248 to 5,134)	-.26 (-.34 to -.18)	-.01 (-.11 to .1)	98 (91 to 105)	63 (59 to 68)	54 (50 to 60)	-.44 (-.5 to -.38)	-.13 (-.22 to -.03)
**Montana**	976 (894 to 1,071)	831 (767 to 901)	854 (740 to 974)	-.12 (-.26 to .03)	.03 (-.11 to .19)	100 (92 to 109)	64 (59 to 70)	54 (47 to 62)	-.46 (-.54 to -.37)	-.16 (-.27 to -.02)
**Nebraska**	1,858 (1,708 to 2,017)	1,340 (1,239 to 1,443)	1,341 (1,205 to 1,478)	-.28 (-.36 to -.18)	0 (-.11 to .12)	93 (86 to 101)	59 (55 to 64)	52 (46 to 57)	-.44 (-.51 to -.37)	-.13 (-.23 to -.02)
**Nevada**	1,339 (1,221 to 1,492)	1,903 (1,750 to 2,109)	2,129 (1,869 to 2,426)	.59 (.39 to .8)	.12 (-.01 to .26)	106 (97 to 117)	66 (61 to 73)	54 (47 to 61)	-.49 (-.56 to -.43)	-.19 (-.28 to -.09)
**New Hampshire**	1,224 (1,130 to 1,331)	952 (880 to 1,023)	974 (876 to 1,080)	-.2 (-.3 to -.09)	.02 (-.09 to .15)	103 (95 to 112)	57 (53 to 61)	47 (42 to 53)	-.54 (-.59 to -.48)	-.17 (-.26 to -.07)
**New Jersey**	8,894 (8,218 to 9,582)	5,930 (5,531 to 6,381)	5,309 (4,768 to 5,921)	-.4 (-.48 to -.32)	-.1 (-.2 to .01)	93 (86 to 100)	53 (50 to 57)	42 (38 to 47)	-.55 (-.61 to -.49)	-.21 (-.3 to -.11)
**New Mexico**	1,323 (1,213 to 1,452)	1,334 (1,233 to 1,441)	1,419 (1,225 to 1,614)	.07 (-.09 to .25)	.06 (-.08 to .23)	85 (78 to 93)	56 (52 to 60)	49 (43 to 56)	-.42 (-.51 to -.32)	-.11 (-.24 to .02)
**New York**	19,121 (17,790 to 20,489)	12,640 (11,807 to 13,600)	11,313 (9,995 to 12,858)	-.41 (-.49 to -.32)	-.1 (-.22 to .03)	89 (82 to 95)	52 (49 to 56)	41 (36 to 47)	-.54 (-.6 to -.46)	-.21 (-.31 to -.08)
**North Carolina**	7,266 (6,763 to 7,836)	6,664 (6,208 to 7,131)	6,911 (6,278 to 7,639)	-.05 (-.15 to .07)	.04 (-.07 to .16)	96 (90 to 104)	61 (57 to 66)	50 (45 to 55)	-.48 (-.54 to -.42)	-.19 (-.28 to -.1)
**North Dakota**	727 (666 to 792)	518 (473 to 563)	510 (445 to 577)	-.3 (-.39 to -.19)	-.01 (-.15 to .12)	90 (82 to 98)	59 (54 to 64)	51 (45 to 59)	-.43 (-.51 to -.34)	-.12 (-.25 to 0)

© 2018 Global Burden of Cardiovascular Diseases Collaboration. *JAMA Cardiology*.

**Ohio**	13,687 (12,719 to 14,669)	10,188 (9,529 to 10,869)	9,727 (8,736 to 10,780)	-.29 (-.37 to -.19)	-.04 (-.14 to .07)	105 (98 to 113)	68 (64 to 73)	59 (52 to 65)	-.44 (-.51 to -.36)	-.14 (-.23 to -.04)
**Oklahoma**	3,448 (3,201 to 3,713)	3,065 (2,872 to 3,280)	3,091 (2,796 to 3,407)	-.1 (-.21 to .02)	.01 (-.1 to .13)	90 (83 to 97)	68 (64 to 73)	60 (54 to 66)	-.33 (-.41 to -.24)	-.12 (-.22 to -.02)
**Oregon**	3,534 (3,261 to 3,815)	2,968 (2,754 to 3,189)	3,063 (2,761 to 3,369)	-.13 (-.24 to -.02)	.03 (-.08 to .15)	100 (92 to 108)	62 (57 to 66)	51 (46 to 56)	-.49 (-.55 to -.43)	-.18 (-.27 to -.08)
**Pennsylvania**	16,340 (15,184 to 17,526)	11,270 (10,573 to 12,031)	10,481 (9,496 to 11,539)	-.36 (-.43 to -.28)	-.07 (-.16 to .04)	100 (93 to 107)	63 (59 to 67)	54 (49 to 59)	-.46 (-.52 to -.39)	-.15 (-.24 to -.04)
**Rhode Island**	1,179 (1,088 to 1,276)	779 (713 to 846)	714 (629 to 814)	-.39 (-.48 to -.3)	-.08 (-.21 to .05)	89 (82 to 97)	54 (50 to 59)	46 (40 to 52)	-.49 (-.56 to -.4)	-.16 (-.28 to -.02)
**South Carolina**	3,990 (3,672 to 4,326)	3,795 (3,508 to 4,099)	4,187 (3,693 to 4,752)	.05 (-.1 to .21)	.1 (-.04 to .26)	106 (98 to 115)	70 (65 to 76)	60 (53 to 67)	-.44 (-.52 to -.35)	-.15 (-.26 to -.03)
**South Dakota**	908 (830 to 987)	693 (633 to 752)	706 (621 to 794)	-.22 (-.34 to -.09)	.02 (-.11 to .17)	102 (93 to 111)	67 (61 to 72)	58 (51 to 66)	-.43 (-.51 to -.33)	-.12 (-.24 to .01)
**Tennessee**	5,109 (4,699 to 5,538)	4,731 (4,404 to 5,071)	4,995 (4,487 to 5,537)	-.02 (-.15 to .11)	.06 (-.06 to .17)	89 (82 to 96)	63 (58 to 67)	54 (49 to 61)	-.39 (-.47 to -.3)	-.13 (-.23 to -.03)
**Texas**	13,271 (12,306 to 14,297)	12,867 (11,960 to 13,752)	14,518 (13,073 to 16,131)	.1 (-.03 to .23)	.13 (.01 to .26)	80 (74 to 86)	53 (49 to 56)	45 (40 to 50)	-.44 (-.51 to -.37)	-.15 (-.24 to -.05)
**Utah**	1,109 (1,017 to 1,201)	1,201 (1,111 to 1,294)	1,341 (1,205 to 1,491)	.21 (.06 to .38)	.12 (0 to .26)	77 (70 to 83)	52 (48 to 56)	43 (39 to 48)	-.44 (-.51 to -.36)	-.17 (-.26 to -.06)
**Vermont**	724 (668 to 783)	553 (512 to 595)	539 (482 to 600)	-.25 (-.34 to -.15)	-.02 (-.14 to .1)	116 (107 to 126)	66 (61 to 71)	54 (48 to 60)	-.54 (-.59 to -.47)	-.18 (-.28 to -.08)
**Virginia**	6,076 (5,654 to 6,538)	4,878 (4,548 to 5,226)	4,943 (4,468 to 5,425)	-.19 (-.28 to -.08)	.01 (-.1 to .13)	95 (88 to 102)	54 (50 to 58)	44 (39 to 48)	-.54 (-.59 to -.48)	-.19 (-.28 to -.1)
**Washington**	5,213 (4,822 to 5,627)	4,625 (4,334 to 4,941)	4,879 (4,378 to 5,358)	-.06 (-.18 to .06)	.06 (-.06 to .18)	97 (90 to 105)	61 (57 to 65)	50 (44 to 55)	-.49 (-.55 to -.43)	-.18 (-.27 to -.09)

© 2018 Global Burden of Cardiovascular Diseases Collaboration. *JAMA Cardiology*.

**West Virginia**	2,551 (2,350 to 2,763)	1,951 (1,797 to 2,097)	1,851 (1,668 to 2,060)	-.27 (-.36 to -.18)	-.05 (-.16 to .07)	106 (98 to 114)	75 (69 to 80)	65 (59 to 73)	-.38 (-.46 to -.3)	-.12 (-.22 to -.01)
**Wisconsin**	5,860 (5,431 to 6,295)	4,516 (4,234 to 4,828)	4,487 (4,072 to 4,932)	-.23 (-.32 to -.13)	-.01 (-.11 to .11)	98 (91 to 106)	63 (59 to 67)	53 (48 to 59)	-.46 (-.52 to -.39)	-.15 (-.24 to -.05)
**Wyoming**	489 (448 to 534)	482 (441 to 522)	492 (427 to 560)	.01 (-.14 to .18)	.02 (-.12 to .18)	107 (99 to 117)	75 (69 to 82)	63 (55 to 72)	-.42 (-.5 to -.31)	-.17 (-.29 to -.04)
**Atrial fibrillation and flutter**										
**Alabama**	5,967 (4,550 to 7,672)	8,038 (6,222 to 10,099)	9,757 (7,582 to 12,309)	.64 (.45 to .84)	.21 (.16 to .28)	121 (92 to 155)	130 (101 to 164)	131 (101 to 165)	.09 (-.04 to .21)	.01 (-.04 to .06)
**Alaska**	325 (242 to 424)	753 (571 to 959)	1,069 (809 to 1,368)	2.3 (2.01 to 2.6)	.42 (.34 to .51)	130 (98 to 169)	144 (111 to 183)	146 (111 to 186)	.12 (.03 to .23)	.01 (-.05 to .07)
**Arizona**	5,313 (4,009 to 6,868)	10,824 (8,418 to 13,651)	15,193 (11,786 to 19,082)	1.87 (1.53 to 2.2)	.4 (.34 to .47)	122 (92 to 158)	138 (107 to 175)	137 (106 to 173)	.13 (0 to .25)	0 (-.05 to .04)
**Arkansas**	3,785 (2,853 to 4,911)	4,767 (3,671 to 6,012)	5,752 (4,440 to 7,216)	.53 (.36 to .71)	.21 (.15 to .26)	115 (86 to 149)	121 (92 to 153)	124 (95 to 156)	.08 (-.03 to .2)	.03 (-.02 to .08)
**California**	27,867 (20,719 to 35,905)	53,886 (41,736 to 67,851)	69,897 (54,711 to 88,461)	1.52 (1.21 to 1.84)	.3 (.24 to .37)	93 (69 to 119)	130 (100 to 164)	129 (101 to 164)	.4 (.23 to .57)	0 (-.05 to .05)
**Colorado**	3,620 (2,706 to 4,704)	6,449 (5,007 to 8,091)	9,248 (7,137 to 11,587)	1.57 (1.23 to 1.92)	.43 (.36 to .51)	112 (83 to 145)	125 (97 to 156)	128 (98 to 160)	.15 (0 to .3)	.02 (-.03 to .07)
**Connecticut**	5,629 (4,283 to 7,247)	8,121 (6,301 to 10,201)	9,136 (7,149 to 11,461)	.63 (.44 to .83)	.13 (.07 to .19)	133 (101 to 171)	156 (121 to 198)	155 (121 to 198)	.17 (.04 to .31)	0 (-.05 to .05)
**Delaware**	1,003 (752 to 1,300)	1,777 (1,385 to 2,255)	2,363 (1,843 to 2,982)	1.36 (1.11 to 1.64)	.33 (.27 to .39)	133 (100 to 172)	152 (118 to 193)	151 (117 to 191)	.14 (.01 to .27)	0 (-.05 to .04)
**District of Columbia**	781 (590 to 1,026)	779 (594 to 983)	870 (659 to 1,096)	.12 (.01 to .23)	.12 (.06 to .18)	107 (80 to 140)	111 (84 to 140)	110 (83 to 139)	.03 (-.06 to .13)	-.01 (-.06 to .05)

© 2018 Global Burden of Cardiovascular Diseases Collaboration. *JAMA Cardiology*.

**Florida**	28,301 (21,458 to 36,459)	46,224 (35,712 to 57,999)	59,546 (46,377 to 74,289)	1.11 (.89 to 1.35)	.29 (.23 to .35)	133 (101 to 171)	153 (118 to 193)	152 (118 to 191)	.14 (.03 to .26)	-.01 (-.05 to .03)
**Georgia**	7,607 (5,731 to 9,849)	12,616 (9,701 to 15,900)	17,097 (13,286 to 21,375)	1.25 (1.03 to 1.49)	.36 (.3 to .42)	120 (91 to 155)	133 (102 to 167)	131 (102 to 164)	.1 (-.01 to .21)	-.01 (-.05 to .04)
**Hawaii**	1,359 (1,021 to 1,778)	2,280 (1,755 to 2,886)	2,960 (2,290 to 3,727)	1.19 (.94 to 1.43)	.3 (.23 to .37)	117 (89 to 153)	122 (93 to 154)	122 (94 to 155)	.05 (-.06 to .16)	.01 (-.05 to .06)
**Idaho**	1,400 (1,048 to 1,815)	2,472 (1,909 to 3,113)	3,504 (2,705 to 4,430)	1.51 (1.24 to 1.79)	.42 (.34 to .5)	122 (91 to 158)	139 (107 to 176)	145 (112 to 183)	.19 (.06 to .33)	.04 (-.02 to .1)
**Illinois**	16,365 (12,276 to 21,279)	20,799 (16,096 to 26,010)	24,747 (19,286 to 31,268)	.52 (.35 to .7)	.19 (.14 to .24)	119 (89 to 155)	128 (98 to 160)	131 (102 to 166)	.1 (-.01 to .23)	.03 (-.02 to .07)
**Indiana**	8,284 (6,267 to 10,730)	11,366 (8,818 to 14,259)	13,807 (10,805 to 17,312)	.67 (.48 to .86)	.22 (.16 to .27)	124 (94 to 161)	138 (106 to 174)	141 (110 to 177)	.14 (.01 to .26)	.02 (-.02 to .08)
**Iowa**	4,907 (3,715 to 6,332)	5,920 (4,594 to 7,429)	6,451 (5,023 to 8,101)	.32 (.16 to .48)	.09 (.03 to .15)	119 (89 to 152)	128 (97 to 162)	125 (96 to 158)	.06 (-.06 to .18)	-.02 (-.07 to .02)
**Kansas**	3,915 (2,956 to 5,045)	4,910 (3,832 to 6,186)	5,826 (4,509 to 7,308)	.49 (.32 to .68)	.19 (.12 to .25)	118 (88 to 153)	127 (99 to 162)	132 (102 to 167)	.13 (0 to .27)	.04 (-.02 to .09)
**Kentucky**	5,862 (4,431 to 7,501)	8,358 (6,496 to 10,462)	10,131 (7,947 to 12,643)	.73 (.54 to .94)	.21 (.16 to .27)	132 (100 to 169)	152 (118 to 191)	153 (120 to 191)	.16 (.04 to .3)	.01 (-.04 to .05)
**Louisiana**	5,543 (4,190 to 7,222)	7,182 (5,544 to 8,994)	8,825 (6,822 to 11,058)	.6 (.43 to .77)	.23 (.17 to .29)	124 (93 to 161)	135 (104 to 170)	136 (105 to 171)	.1 (-.02 to .22)	0 (-.04 to .05)
**Maine**	2,126 (1,620 to 2,723)	3,241 (2,512 to 4,078)	3,921 (3,040 to 4,922)	.85 (.66 to 1.06)	.21 (.16 to .26)	135 (102 to 173)	159 (123 to 202)	158 (122 to 199)	.17 (.05 to .31)	-.01 (-.05 to .03)
**Maryland**	6,228 (4,691 to 8,027)	9,704 (7,527 to 12,177)	12,245 (9,469 to 15,305)	.97 (.76 to 1.19)	.26 (.21 to .32)	126 (95 to 162)	140 (108 to 175)	141 (109 to 177)	.13 (0 to .25)	.01 (-.04 to .05)
**Massachusetts**	10,670 (8,094 to 13,615)	15,439 (11,927 to 19,375)	17,263 (13,596 to 21,744)	.62 (.46 to .79)	.12 (.07 to .17)	137 (104 to 176)	168 (130 to 213)	160 (125 to 202)	.17 (.06 to .29)	-.05 (-.09 to 0)

© 2018 Global Burden of Cardiovascular Diseases Collaboration. *JAMA Cardiology*.

**Michigan**	13,067 (9,912 to 16,655)	17,720 (13,681 to 22,109)	21,926 (17,036 to 27,512)	.68 (.54 to .83)	.24 (.17 to .31)	124 (94 to 158)	132 (101 to 165)	139 (107 to 174)	.12 (.03 to .22)	.05 (0 to .11)
**Minnesota**	6,264 (4,750 to 8,128)	8,642 (6,667 to 10,921)	10,679 (8,252 to 13,443)	.71 (.5 to .92)	.24 (.17 to .3)	116 (87 to 151)	127 (98 to 161)	127 (98 to 161)	.1 (-.03 to .24)	.01 (-.04 to .06)
**Mississippi**	3,577 (2,691 to 4,632)	4,675 (3,640 to 5,902)	5,428 (4,227 to 6,795)	.52 (.36 to .69)	.16 (.1 to .22)	117 (87 to 151)	129 (100 to 163)	125 (97 to 157)	.08 (-.04 to .19)	-.03 (-.08 to .03)
**Missouri**	8,482 (6,420 to 10,811)	11,557 (8,980 to 14,535)	14,012 (10,916 to 17,730)	.66 (.46 to .86)	.21 (.16 to .27)	123 (93 to 158)	142 (110 to 179)	145 (112 to 185)	.19 (.05 to .32)	.03 (-.02 to .07)
**Montana**	1,254 (943 to 1,617)	2,096 (1,622 to 2,623)	2,607 (2,025 to 3,275)	1.09 (.82 to 1.36)	.24 (.18 to .31)	124 (93 to 160)	150 (116 to 190)	147 (114 to 186)	.19 (.04 to .34)	-.02 (-.07 to .03)
**Nebraska**	2,687 (2,041 to 3,441)	3,390 (2,628 to 4,220)	3,923 (2,996 to 4,914)	.46 (.32 to .61)	.16 (.1 to .22)	123 (93 to 158)	135 (103 to 169)	135 (102 to 171)	.1 (0 to .21)	0 (-.05 to .05)
**Nevada**	1,397 (1,044 to 1,828)	3,623 (2,799 to 4,587)	5,176 (4,027 to 6,523)	2.72 (2.26 to 3.19)	.43 (.37 to .5)	119 (90 to 154)	129 (100 to 163)	127 (98 to 159)	.07 (-.06 to .2)	-.02 (-.06 to .03)
**New Hampshire**	1,631 (1,231 to 2,095)	2,799 (2,173 to 3,507)	3,549 (2,763 to 4,480)	1.18 (.94 to 1.43)	.27 (.22 to .33)	133 (100 to 172)	161 (125 to 203)	158 (122 to 201)	.19 (.06 to .32)	-.02 (-.06 to .03)
**New Jersey**	12,518 (9,544 to 16,032)	17,694 (13,834 to 22,170)	20,760 (16,168 to 25,820)	.66 (.48 to .85)	.17 (.12 to .23)	129 (99 to 166)	147 (114 to 185)	149 (115 to 187)	.16 (.04 to .28)	.01 (-.04 to .06)
**New Mexico**	1,664 (1,245 to 2,172)	2,623 (2,036 to 3,288)	3,350 (2,618 to 4,193)	1.02 (.76 to 1.29)	.28 (.21 to .35)	108 (81 to 140)	105 (82 to 132)	103 (80 to 129)	-.04 (-.16 to .09)	-.02 (-.07 to .03)
**New York**	29,543 (22,289 to 37,949)	39,194 (30,478 to 49,441)	45,115 (35,316 to 56,581)	.53 (.36 to .71)	.15 (.1 to .21)	131 (99 to 169)	149 (115 to 189)	149 (116 to 189)	.14 (.02 to .27)	0 (-.04 to .05)
**North Carolina**	9,236 (6,995 to 11,859)	15,430 (11,974 to 19,383)	20,190 (15,676 to 25,417)	1.19 (.94 to 1.46)	.31 (.26 to .36)	122 (92 to 157)	137 (106 to 172)	134 (104 to 170)	.1 (-.02 to .23)	-.02 (-.06 to .02)
**North Dakota**	1,020 (764 to 1,329)	1,265 (979 to 1,582)	1,434 (1,105 to 1,824)	.41 (.28 to .54)	.13 (.07 to .2)	115 (85 to 150)	125 (96 to 157)	126 (96 to 159)	.1 (0 to .21)	.01 (-.05 to .07)

© 2018 Global Burden of Cardiovascular Diseases Collaboration. *JAMA Cardiology*.

**Ohio**	17,111 (12,952 to 22,154)	24,036 (18,628 to 29,952)	28,063 (21,803 to 35,023)	.65 (.46 to .84)	.17 (.12 to .22)	129 (97 to 166)	149 (116 to 186)	151 (117 to 189)	.18 (.05 to .31)	.01 (-.03 to .05)
**Oklahoma**	4,893 (3,701 to 6,207)	6,419 (4,984 to 8,092)	7,983 (6,238 to 10,001)	.64 (.45 to .83)	.24 (.19 to .3)	120 (91 to 153)	132 (103 to 168)	140 (109 to 175)	.17 (.04 to .3)	.05 (0 to .1)
**Oregon**	4,453 (3,350 to 5,816)	7,251 (5,643 to 9,074)	9,212 (7,208 to 11,544)	1.08 (.85 to 1.31)	.27 (.21 to .33)	121 (91 to 158)	141 (109 to 177)	140 (110 to 177)	.16 (.03 to .29)	0 (-.05 to .05)
**Pennsylvania**	22,207 (16,778 to 28,670)	29,416 (22,822 to 36,829)	32,953 (25,784 to 41,231)	.49 (.33 to .66)	.12 (.08 to .17)	132 (100 to 171)	149 (115 to 188)	149 (116 to 188)	.14 (.02 to .27)	0 (-.03 to .04)
**Rhode Island**	1,735 (1,312 to 2,233)	2,344 (1,816 to 2,942)	2,599 (2,009 to 3,266)	.5 (.34 to .66)	.11 (.05 to .17)	124 (94 to 160)	145 (112 to 184)	148 (114 to 188)	.2 (.07 to .32)	.02 (-.04 to .07)
**South Carolina**	4,475 (3,366 to 5,760)	7,660 (5,905 to 9,658)	10,554 (8,226 to 13,186)	1.37 (1.09 to 1.66)	.38 (.31 to .45)	122 (92 to 156)	136 (105 to 172)	138 (108 to 173)	.14 (.01 to .27)	.01 (-.03 to .07)
**South Dakota**	1,188 (898 to 1,524)	1,566 (1,209 to 1,984)	1,933 (1,487 to 2,453)	.63 (.48 to .81)	.24 (.17 to .31)	120 (90 to 154)	133 (102 to 169)	138 (106 to 176)	.15 (.05 to .28)	.04 (-.02 to .09)
**Tennessee**	7,213 (5,466 to 9,317)	10,318 (7,976 to 13,049)	13,478 (10,347 to 16,897)	.87 (.69 to 1.07)	.31 (.24 to .37)	122 (93 to 159)	131 (101 to 166)	136 (104 to 170)	.11 (.01 to .22)	.04 (-.01 to .08)
**Texas**	20,236 (15,254 to 26,172)	34,109 (26,381 to 42,904)	46,258 (36,216 to 58,253)	1.29 (1.05 to 1.56)	.36 (.3 to .42)	121 (91 to 156)	140 (108 to 176)	140 (109 to 176)	.16 (.04 to .28)	0 (-.04 to .05)
**Utah**	1,705 (1,294 to 2,209)	3,039 (2,342 to 3,822)	4,159 (3,203 to 5,213)	1.45 (1.17 to 1.73)	.37 (.3 to .43)	118 (89 to 153)	131 (101 to 166)	132 (102 to 166)	.12 (0 to .25)	.01 (-.04 to .05)
**Vermont**	822 (620 to 1,060)	1,294 (996 to 1,629)	1,620 (1,241 to 2,045)	.98 (.79 to 1.17)	.25 (.19 to .32)	126 (95 to 163)	146 (111 to 185)	148 (112 to 187)	.17 (.06 to .29)	.01 (-.04 to .06)
**Virginia**	7,736 (5,839 to 9,955)	12,852 (9,957 to 16,115)	16,281 (12,644 to 20,348)	1.11 (.87 to 1.37)	.27 (.22 to .32)	121 (91 to 156)	139 (107 to 174)	136 (106 to 171)	.13 (0 to .27)	-.02 (-.06 to .03)
**Washington**	6,724 (5,048 to 8,655)	11,179 (8,679 to 14,103)	14,763 (11,411 to 18,563)	1.2 (.95 to 1.48)	.32 (.27 to .38)	123 (92 to 158)	140 (108 to 177)	141 (109 to 178)	.15 (.02 to .29)	.01 (-.04 to .05)

© 2018 Global Burden of Cardiovascular Diseases Collaboration. *JAMA Cardiology*.

**West Virginia**	3,174 (2,383 to 4,054)	4,078 (3,137 to 5,113)	4,727 (3,678 to 5,941)	.49 (.33 to .66)	.16 (.11 to .22)	127 (95 to 162)	143 (109 to 180)	147 (114 to 186)	.17 (.04 to .29)	.03 (-.02 to .08)
**Wisconsin**	7,676 (5,787 to 9,872)	10,566 (8,241 to 13,292)	12,571 (9,713 to 15,737)	.64 (.45 to .83)	.19 (.14 to .24)	122 (92 to 157)	136 (105 to 172)	136 (104 to 171)	.12 (-.01 to .24)	0 (-.04 to .04)
**Wyoming**	511 (385 to 668)	769 (591 to 978)	984 (754 to 1,241)	.93 (.74 to 1.14)	.28 (.21 to .35)	111 (84 to 146)	116 (89 to 147)	117 (90 to 148)	.05 (-.05 to .16)	.01 (-.04 to .07)
**Endocarditis**										
**Alabama**	1,569 (1,225 to 2,092)	2,287 (1,552 to 2,703)	2,705 (1,853 to 3,330)	.74 (.33 to 1.13)	.19 (.03 to .35)	34 (27 to 46)	41 (28 to 48)	43 (29 to 53)	.25 (-.05 to .54)	.05 (-.09 to .2)
**Alaska**	166 (101 to 203)	303 (176 to 378)	387 (219 to 504)	1.33 (.96 to 1.72)	.28 (.1 to .48)	44 (27 to 53)	48 (27 to 59)	49 (28 to 65)	.12 (-.06 to .31)	.04 (-.1 to .2)
**Arizona**	1,381 (1,065 to 1,812)	2,631 (1,943 to 3,311)	3,265 (2,530 to 4,405)	1.37 (1.1 to 1.68)	.24 (.07 to .44)	34 (26 to 45)	37 (27 to 46)	36 (28 to 48)	.06 (-.07 to .19)	-.03 (-.16 to .12)
**Arkansas**	944 (751 to 1,249)	1,406 (958 to 1,692)	1,649 (1,123 to 1,999)	.76 (.39 to 1.12)	.17 (.05 to .31)	34 (26 to 44)	41 (28 to 48)	43 (29 to 52)	.28 (.01 to .55)	.06 (-.06 to .18)
**California**	9,283 (7,705 to 13,968)	12,147 (9,813 to 18,482)	14,727 (11,102 to 23,411)	.58 (.31 to .88)	.21 (.05 to .38)	31 (26 to 46)	30 (24 to 46)	30 (23 to 49)	-.03 (-.2 to .18)	-.01 (-.14 to .14)
**Colorado**	1,138 (854 to 1,470)	1,928 (1,421 to 2,374)	2,601 (1,898 to 3,268)	1.29 (1 to 1.62)	.35 (.21 to .5)	34 (26 to 44)	37 (27 to 45)	38 (28 to 48)	.11 (-.02 to .27)	.04 (-.07 to .16)
**Connecticut**	1,299 (1,063 to 1,828)	1,548 (1,226 to 2,117)	1,688 (1,349 to 2,361)	.3 (.13 to .48)	.09 (-.05 to .24)	33 (28 to 47)	34 (27 to 47)	34 (27 to 48)	.01 (-.12 to .16)	0 (-.13 to .13)
**Delaware**	288 (208 to 352)	448 (301 to 530)	568 (393 to 678)	.97 (.74 to 1.23)	.27 (.13 to .41)	39 (29 to 48)	42 (28 to 50)	43 (29 to 51)	.09 (-.05 to .24)	.03 (-.08 to .14)
**District of Columbia**	416 (232 to 521)	364 (219 to 439)	393 (262 to 489)	-.03 (-.24 to .28)	.09 (-.1 to .32)	61 (33 to 76)	55 (33 to 66)	51 (34 to 64)	-.13 (-.32 to .15)	-.06 (-.22 to .14)

© 2018 Global Burden of Cardiovascular Diseases Collaboration. *JAMA Cardiology*.

**Florida**	5,953 (4,742 to 8,176)	9,200 (6,944 to 11,827)	11,498 (8,801 to 15,265)	.94 (.7 to 1.22)	.25 (.1 to .41)	35 (28 to 47)	37 (28 to 48)	38 (29 to 50)	.1 (-.03 to .26)	.03 (-.09 to .15)
**Georgia**	2,329 (1,770 to 3,043)	3,899 (2,801 to 4,774)	5,003 (3,635 to 6,224)	1.15 (.85 to 1.47)	.29 (.12 to .48)	36 (27 to 47)	40 (29 to 49)	41 (30 to 51)	.14 (-.02 to .31)	.03 (-.1 to .18)
**Hawaii**	385 (318 to 556)	582 (453 to 777)	711 (566 to 959)	.86 (.59 to 1.12)	.22 (.1 to .34)	33 (27 to 47)	35 (27 to 47)	36 (28 to 49)	.1 (-.07 to .25)	.03 (-.07 to .13)
**Idaho**	363 (271 to 449)	630 (426 to 742)	823 (563 to 1,003)	1.27 (.9 to 1.68)	.31 (.14 to .49)	34 (25 to 42)	37 (25 to 44)	39 (26 to 47)	.14 (-.05 to .35)	.03 (-.1 to .18)
**Illinois**	4,464 (3,493 to 5,993)	5,431 (4,225 to 7,250)	6,064 (4,955 to 8,495)	.36 (.2 to .54)	.12 (-.01 to .25)	35 (27 to 47)	37 (29 to 49)	37 (30 to 52)	.05 (-.08 to .2)	0 (-.11 to .12)
**Indiana**	2,076 (1,602 to 2,772)	2,957 (2,087 to 3,562)	3,460 (2,335 to 4,207)	.67 (.34 to 1.04)	.17 (.03 to .31)	33 (26 to 45)	39 (28 to 47)	41 (28 to 49)	.22 (-.03 to .49)	.04 (-.08 to .17)
**Iowa**	959 (752 to 1,512)	1,119 (898 to 1,733)	1,293 (1,032 to 1,976)	.36 (.18 to .56)	.16 (.04 to .3)	27 (21 to 44)	29 (23 to 44)	30 (24 to 46)	.12 (-.04 to .29)	.06 (-.06 to .19)
**Kansas**	890 (729 to 1,278)	1,209 (897 to 1,503)	1,394 (1,021 to 1,788)	.58 (.23 to .96)	.15 (0 to .32)	31 (25 to 44)	36 (26 to 45)	37 (27 to 48)	.22 (-.05 to .51)	.04 (-.1 to .2)
**Kentucky**	1,341 (1,073 to 1,842)	1,964 (1,384 to 2,396)	2,357 (1,633 to 2,839)	.77 (.42 to 1.17)	.2 (.08 to .34)	32 (26 to 44)	38 (27 to 47)	41 (28 to 49)	.27 (.01 to .56)	.06 (-.04 to .19)
**Louisiana**	1,611 (1,197 to 2,040)	2,150 (1,430 to 2,516)	2,587 (1,733 to 3,139)	.61 (.33 to .88)	.21 (.09 to .34)	37 (28 to 47)	43 (29 to 50)	45 (30 to 54)	.21 (0 to .41)	.04 (-.07 to .15)
**Maine**	471 (365 to 634)	640 (471 to 810)	743 (554 to 949)	.58 (.36 to .81)	.16 (.05 to .29)	33 (26 to 44)	35 (26 to 45)	36 (27 to 46)	.12 (-.04 to .29)	.03 (-.07 to .13)
**Maryland**	1,875 (1,456 to 2,518)	2,544 (1,982 to 3,362)	2,999 (2,381 to 4,120)	.6 (.42 to .8)	.18 (.04 to .32)	37 (29 to 50)	38 (30 to 51)	39 (31 to 54)	.05 (-.06 to .18)	.01 (-.1 to .13)
**Massachusetts**	2,213 (1,824 to 3,220)	2,645 (2,204 to 3,777)	3,004 (2,413 to 4,374)	.36 (.21 to .52)	.13 (.01 to .25)	31 (26 to 45)	32 (27 to 46)	32 (26 to 47)	.03 (-.08 to .15)	0 (-.1 to .11)

© 2018 Global Burden of Cardiovascular Diseases Collaboration. *JAMA Cardiology*.

**Michigan**	3,525 (2,695 to 4,681)	4,733 (3,465 to 5,879)	5,445 (4,021 to 6,773)	.55 (.36 to .76)	.15 (.05 to .26)	35 (27 to 46)	39 (28 to 49)	41 (30 to 51)	.16 (.02 to .31)	.05 (-.05 to .15)
**Minnesota**	1,448 (1,195 to 2,113)	1,917 (1,574 to 2,702)	2,316 (1,874 to 3,279)	.61 (.41 to .85)	.21 (.08 to .34)	29 (24 to 43)	31 (25 to 44)	31 (25 to 45)	.07 (-.06 to .23)	.03 (-.08 to .15)
**Mississippi**	923 (737 to 1,291)	1,310 (947 to 1,622)	1,531 (1,127 to 1,938)	.67 (.35 to 1.01)	.17 (.02 to .33)	33 (26 to 46)	39 (28 to 48)	41 (30 to 51)	.25 (0 to .5)	.05 (-.09 to .2)
**Missouri**	1,917 (1,557 to 2,737)	2,655 (1,977 to 3,343)	3,082 (2,332 to 3,962)	.62 (.33 to .93)	.16 (.05 to .28)	32 (26 to 45)	37 (27 to 47)	38 (29 to 49)	.21 (-.01 to .43)	.04 (-.06 to .14)
**Montana**	368 (240 to 437)	552 (325 to 671)	668 (395 to 846)	.81 (.52 to 1.15)	.21 (.05 to .39)	40 (26 to 47)	44 (26 to 54)	45 (27 to 58)	.13 (-.07 to .35)	.03 (-.1 to .17)
**Nebraska**	657 (496 to 826)	875 (594 to 1,025)	1,012 (693 to 1,212)	.54 (.29 to .81)	.16 (.04 to .28)	35 (27 to 44)	39 (27 to 46)	41 (28 to 49)	.16 (-.02 to .36)	.03 (-.08 to .14)
**Nevada**	480 (362 to 601)	1,173 (821 to 1,411)	1,534 (1,150 to 1,941)	2.2 (1.82 to 2.58)	.31 (.13 to .49)	38 (29 to 48)	41 (29 to 50)	41 (31 to 52)	.07 (-.06 to .19)	0 (-.14 to .13)
**New Hampshire**	404 (316 to 537)	572 (434 to 723)	703 (535 to 914)	.75 (.52 to .98)	.23 (.11 to .36)	34 (26 to 45)	35 (27 to 44)	36 (28 to 47)	.07 (-.06 to .21)	.04 (-.06 to .15)
**New Jersey**	3,458 (2,552 to 4,271)	4,299 (3,046 to 5,235)	4,830 (3,514 to 5,958)	.4 (.23 to .58)	.13 (-.01 to .26)	38 (28 to 47)	40 (28 to 49)	40 (29 to 50)	.05 (-.08 to .18)	.01 (-.11 to .13)
**New Mexico**	532 (400 to 678)	873 (617 to 1,057)	1,081 (746 to 1,335)	1.03 (.68 to 1.4)	.24 (.08 to .41)	35 (26 to 44)	38 (26 to 46)	40 (27 to 49)	.15 (-.08 to .38)	.06 (-.09 to .21)
**New York**	7,571 (5,813 to 9,823)	8,593 (6,685 to 11,332)	9,700 (7,616 to 13,312)	.28 (.1 to .5)	.13 (-.01 to .29)	37 (28 to 48)	36 (28 to 48)	37 (29 to 51)	.01 (-.15 to .2)	.01 (-.11 to .16)
**North Carolina**	2,502 (1,944 to 3,340)	3,928 (2,839 to 4,850)	5,043 (3,753 to 6,368)	1.02 (.76 to 1.29)	.29 (.15 to .42)	34 (27 to 46)	37 (27 to 46)	38 (28 to 48)	.12 (-.02 to .27)	.03 (-.07 to .14)
**North Dakota**	250 (197 to 334)	307 (227 to 377)	371 (266 to 454)	.49 (.23 to .77)	.21 (.07 to .35)	33 (26 to 44)	36 (27 to 44)	38 (28 to 47)	.17 (-.04 to .4)	.06 (-.06 to .21)

© 2018 Global Burden of Cardiovascular Diseases Collaboration. *JAMA Cardiology*.

**Ohio**	4,247 (3,287 to 5,622)	5,747 (4,079 to 6,867)	6,602 (4,574 to 7,891)	.56 (.27 to .83)	.15 (.04 to .27)	34 (27 to 46)	40 (29 to 48)	42 (29 to 50)	.23 (0 to .44)	.05 (-.04 to .16)
**Oklahoma**	1,166 (949 to 1,636)	1,727 (1,193 to 2,070)	2,087 (1,452 to 2,533)	.81 (.4 to 1.22)	.21 (.1 to .34)	32 (26 to 45)	40 (28 to 48)	43 (30 to 51)	.34 (.03 to .64)	.07 (-.03 to .19)
**Oregon**	1,216 (860 to 1,472)	1,767 (1,230 to 2,074)	2,181 (1,535 to 2,613)	.79 (.59 to 1.02)	.24 (.11 to .36)	36 (26 to 44)	38 (26 to 44)	38 (27 to 46)	.06 (-.06 to .19)	.02 (-.08 to .13)
**Pennsylvania**	5,816 (4,004 to 6,904)	7,154 (4,614 to 8,432)	7,981 (5,284 to 9,576)	.37 (.21 to .54)	.12 (.01 to .23)	39 (27 to 46)	42 (28 to 50)	43 (29 to 52)	.11 (-.01 to .25)	.03 (-.07 to .13)
**Rhode Island**	387 (319 to 555)	460 (372 to 629)	502 (403 to 703)	.31 (.11 to .54)	.09 (-.05 to .24)	32 (26 to 45)	33 (27 to 46)	34 (27 to 47)	.07 (-.08 to .26)	.03 (-.11 to .17)
**South Carolina**	1,296 (984 to 1,678)	2,068 (1,427 to 2,489)	2,666 (1,828 to 3,265)	1.06 (.73 to 1.41)	.29 (.14 to .47)	35 (27 to 46)	39 (27 to 48)	41 (28 to 50)	.16 (-.04 to .36)	.03 (-.09 to .17)
**South Dakota**	281 (214 to 358)	371 (264 to 445)	451 (323 to 550)	.61 (.34 to .9)	.22 (.08 to .37)	34 (26 to 43)	37 (26 to 44)	39 (28 to 48)	.16 (-.04 to .39)	.06 (-.06 to .2)
**Tennessee**	1,840 (1,447 to 2,520)	2,867 (2,045 to 3,508)	3,560 (2,548 to 4,333)	.95 (.58 to 1.31)	.24 (.12 to .37)	33 (26 to 46)	39 (28 to 48)	41 (30 to 50)	.25 (.01 to .48)	.06 (-.05 to .17)
**Texas**	5,373 (4,384 to 7,570)	8,586 (6,739 to 11,246)	11,337 (8,906 to 15,481)	1.12 (.85 to 1.42)	.32 (.19 to .46)	32 (26 to 45)	35 (27 to 46)	36 (28 to 49)	.13 (-.02 to .28)	.03 (-.08 to .13)
**Utah**	590 (395 to 692)	1,045 (637 to 1,263)	1,379 (848 to 1,705)	1.33 (1 to 1.67)	.32 (.19 to .47)	40 (27 to 47)	44 (27 to 53)	45 (27 to 55)	.12 (-.04 to .29)	.02 (-.09 to .13)
**Vermont**	207 (161 to 273)	275 (209 to 360)	321 (244 to 424)	.56 (.39 to .76)	.17 (.05 to .3)	34 (26 to 44)	34 (26 to 45)	34 (26 to 46)	.03 (-.09 to .15)	.02 (-.09 to .14)
**Virginia**	2,233 (1,733 to 2,986)	3,288 (2,472 to 4,189)	3,962 (3,066 to 5,127)	.78 (.6 to .99)	.21 (.08 to .33)	35 (27 to 46)	37 (28 to 47)	37 (29 to 48)	.07 (-.04 to .19)	0 (-.1 to .11)
**Washington**	1,951 (1,421 to 2,376)	2,880 (2,059 to 3,436)	3,613 (2,573 to 4,454)	.85 (.63 to 1.08)	.26 (.13 to .38)	37 (27 to 45)	38 (27 to 45)	38 (28 to 47)	.04 (-.09 to .16)	.01 (-.09 to .12)

© 2018 Global Burden of Cardiovascular Diseases Collaboration. *JAMA Cardiology*.

**West Virginia**	727 (578 to 996)	944 (661 to 1,130)	1,071 (738 to 1,293)	.48 (.17 to .78)	.14 (.03 to .26)	33 (26 to 45)	39 (27 to 46)	41 (28 to 50)	.26 (-.03 to .53)	.07 (-.03 to .2)
**Wisconsin**	1,828 (1,432 to 2,484)	2,503 (1,893 to 3,170)	2,926 (2,246 to 3,767)	.61 (.37 to .84)	.17 (.06 to .28)	32 (26 to 44)	36 (27 to 46)	37 (28 to 47)	.14 (-.02 to .3)	.03 (-.07 to .13)
**Wyoming**	184 (119 to 220)	293 (170 to 368)	359 (215 to 462)	.94 (.62 to 1.29)	.23 (.07 to .41)	40 (26 to 48)	47 (27 to 58)	48 (29 to 62)	.18 (-.02 to .4)	.04 (-.09 to .18)
**Hypertensive heart disease**										
**Alabama**	8,493 (5,951 to 10,286)	11,127 (8,069 to 14,657)	12,776 (9,358 to 16,765)	.51 (.29 to .79)	.15 (.02 to .29)	184 (129 to 223)	190 (137 to 248)	193 (138 to 248)	.05 (-.1 to .22)	.02 (-.1 to .15)
**Alaska**	438 (359 to 656)	863 (682 to 1,159)	1,078 (808 to 1,454)	1.51 (.83 to 2.09)	.25 (.07 to .47)	131 (108 to 193)	131 (105 to 178)	134 (101 to 180)	.04 (-.22 to .27)	.02 (-.13 to .2)
**Arizona**	4,498 (3,612 to 7,057)	10,539 (7,277 to 12,666)	14,316 (9,098 to 17,128)	2.32 (1 to 3.33)	.35 (.17 to .55)	112 (89 to 176)	144 (99 to 174)	150 (94 to 181)	.41 (-.17 to .86)	.04 (-.11 to .19)
**Arkansas**	3,884 (3,176 to 5,815)	6,291 (4,689 to 8,167)	8,110 (5,336 to 9,649)	1.15 (.35 to 1.68)	.29 (.07 to .49)	133 (109 to 202)	174 (128 to 223)	201 (129 to 239)	.56 (-.06 to .98)	.16 (-.05 to .35)
**California**	49,366 (32,767 to 57,421)	69,158 (40,261 to 82,860)	79,378 (45,079 to 99,125)	.6 (.23 to .89)	.15 (.02 to .29)	169 (112 to 197)	168 (98 to 201)	155 (88 to 195)	-.09 (-.3 to .08)	-.08 (-.18 to .04)
**Colorado**	3,393 (2,729 to 5,351)	5,907 (4,811 to 8,242)	7,982 (6,124 to 10,676)	1.42 (.75 to 1.96)	.36 (.17 to .55)	105 (84 to 165)	109 (89 to 152)	112 (86 to 150)	.1 (-.2 to .34)	.03 (-.11 to .17)
**Connecticut**	4,928 (3,954 to 6,879)	5,125 (4,193 to 7,170)	5,748 (4,499 to 7,941)	.17 (.01 to .35)	.12 (0 to .28)	126 (101 to 176)	106 (87 to 150)	108 (84 to 148)	-.14 (-.28 to .01)	.02 (-.11 to .17)
**Delaware**	1,466 (946 to 1,704)	1,945 (1,324 to 2,269)	2,353 (1,566 to 2,769)	.61 (.42 to .84)	.21 (.1 to .34)	201 (130 to 234)	176 (118 to 205)	174 (111 to 206)	-.14 (-.24 to -.03)	-.01 (-.11 to .1)
**District of Columbia**	4,192 (1,433 to 5,546)	3,432 (1,142 to 4,617)	3,225 (1,105 to 4,602)	-.24 (-.42 to -.08)	-.06 (-.2 to .1)	629 (210 to 837)	512 (168 to 689)	419 (143 to 598)	-.34 (-.5 to -.2)	-.18 (-.3 to -.04)

© 2018 Global Burden of Cardiovascular Diseases Collaboration. *JAMA Cardiology*.

**Florida**	29,840 (19,872 to 35,266)	44,646 (27,989 to 52,823)	55,149 (32,358 to 69,041)	.84 (.51 to 1.16)	.23 (.11 to .38)	170 (114 to 201)	175 (106 to 209)	177 (99 to 224)	.04 (-.19 to .25)	.01 (-.11 to .14)
**Georgia**	12,874 (8,438 to 15,090)	24,599 (13,233 to 30,229)	33,795 (16,117 to 44,300)	1.59 (.69 to 2.33)	.36 (.14 to .59)	204 (133 to 239)	244 (132 to 298)	262 (125 to 344)	.27 (-.17 to .63)	.06 (-.11 to .25)
**Hawaii**	1,285 (1,078 to 1,920)	1,761 (1,460 to 2,617)	2,157 (1,749 to 3,148)	.68 (.48 to .88)	.23 (.12 to .35)	112 (94 to 167)	102 (84 to 151)	105 (84 to 153)	-.06 (-.19 to .06)	.03 (-.07 to .15)
**Idaho**	1,084 (852 to 1,755)	1,868 (1,530 to 2,751)	2,495 (2,017 to 3,584)	1.35 (.81 to 1.85)	.34 (.17 to .54)	101 (79 to 166)	107 (88 to 158)	112 (91 to 160)	.14 (-.14 to .39)	.05 (-.09 to .21)
**Illinois**	23,231 (15,774 to 27,127)	29,784 (17,705 to 35,530)	35,513 (18,720 to 45,011)	.52 (.06 to .87)	.18 (-.01 to .36)	184 (125 to 215)	194 (114 to 232)	205 (105 to 262)	.11 (-.24 to .38)	.05 (-.12 to .22)
**Indiana**	7,609 (6,134 to 11,865)	10,603 (8,814 to 15,281)	12,880 (10,101 to 17,895)	.72 (.35 to 1.05)	.22 (.07 to .39)	120 (96 to 191)	134 (112 to 193)	143 (111 to 197)	.21 (-.08 to .47)	.07 (-.08 to .23)
**Iowa**	4,184 (3,425 to 5,943)	4,563 (3,741 to 6,493)	5,243 (4,115 to 7,205)	.26 (.09 to .42)	.15 (.03 to .29)	115 (95 to 167)	109 (89 to 156)	114 (89 to 158)	-.01 (-.15 to .14)	.05 (-.07 to .18)
**Kansas**	3,006 (2,344 to 4,892)	3,591 (2,798 to 6,008)	3,982 (2,981 to 6,568)	.32 (.14 to .53)	.11 (-.02 to .25)	100 (77 to 171)	100 (77 to 169)	100 (74 to 165)	0 (-.15 to .15)	.01 (-.12 to .15)
**Kentucky**	5,750 (4,726 to 8,399)	9,145 (6,893 to 11,728)	11,859 (7,889 to 14,135)	1.1 (.41 to 1.56)	.3 (.11 to .48)	137 (113 to 203)	172 (129 to 219)	197 (129 to 233)	.47 (-.05 to .82)	.14 (-.04 to .32)
**Louisiana**	11,694 (6,227 to 14,185)	15,362 (7,611 to 18,913)	18,907 (8,853 to 24,263)	.6 (.31 to .84)	.22 (.08 to .38)	271 (145 to 329)	296 (147 to 365)	313 (144 to 404)	.14 (-.08 to .32)	.05 (-.08 to .19)
**Maine**	1,504 (1,186 to 2,447)	1,840 (1,459 to 3,004)	2,105 (1,644 to 3,442)	.41 (.26 to .57)	.15 (.04 to .27)	103 (81 to 170)	96 (76 to 157)	97 (76 to 155)	-.05 (-.18 to .08)	.01 (-.1 to .13)
**Maryland**	11,488 (6,630 to 13,628)	16,377 (8,566 to 20,248)	19,517 (9,535 to 25,082)	.67 (.26 to .99)	.18 (.05 to .34)	232 (133 to 276)	237 (125 to 291)	240 (116 to 310)	.02 (-.24 to .22)	.01 (-.12 to .14)
**Massachusetts**	7,727 (6,404 to 12,080)	9,125 (7,554 to 12,761)	10,323 (8,110 to 14,306)	.36 (.05 to .59)	.13 (0 to .27)	109 (90 to 170)	106 (88 to 148)	105 (80 to 143)	-.02 (-.26 to .17)	-.01 (-.13 to .13)

© 2018 Global Burden of Cardiovascular Diseases Collaboration. *JAMA Cardiology*.

**Michigan**	15,735 (12,073 to 20,486)	23,734 (15,318 to 27,473)	29,039 (17,358 to 35,280)	.85 (.3 to 1.28)	.22 (.06 to .39)	157 (120 to 204)	185 (119 to 214)	205 (119 to 250)	.32 (-.1 to .63)	.1 (-.06 to .26)
**Minnesota**	4,086 (2,992 to 7,343)	4,908 (3,647 to 8,901)	5,723 (4,177 to 10,405)	.41 (.25 to .58)	.17 (.06 to .3)	82 (59 to 150)	74 (55 to 136)	73 (53 to 132)	-.1 (-.22 to .02)	-.01 (-.1 to .11)
**Mississippi**	6,718 (4,159 to 7,917)	11,422 (5,375 to 14,263)	13,507 (6,100 to 17,747)	.98 (.38 to 1.44)	.18 (.02 to .34)	239 (149 to 282)	327 (154 to 410)	344 (152 to 454)	.41 (-.04 to .76)	.04 (-.1 to .2)
**Missouri**	8,978 (6,926 to 11,810)	11,629 (8,757 to 14,760)	13,664 (9,793 to 16,994)	.53 (.29 to .72)	.18 (.06 to .3)	146 (113 to 193)	155 (114 to 195)	163 (114 to 197)	.12 (-.09 to .29)	.05 (-.06 to .17)
**Montana**	946 (733 to 1,568)	1,381 (1,123 to 2,147)	1,644 (1,302 to 2,470)	.77 (.42 to 1.1)	.2 (.05 to .36)	101 (78 to 170)	105 (85 to 163)	106 (84 to 157)	.06 (-.17 to .28)	.01 (-.12 to .16)
**Nebraska**	1,971 (1,556 to 3,227)	2,365 (1,941 to 3,638)	2,614 (2,096 to 4,159)	.34 (.17 to .5)	.11 (.01 to .2)	102 (80 to 167)	102 (84 to 159)	100 (80 to 158)	-.01 (-.16 to .12)	-.02 (-.1 to .08)
**Nevada**	3,651 (1,669 to 4,676)	8,253 (3,663 to 10,598)	10,813 (4,736 to 14,384)	1.94 (1.53 to 2.33)	.31 (.17 to .46)	294 (135 to 375)	282 (126 to 361)	277 (121 to 369)	-.07 (-.2 to .06)	-.02 (-.13 to .09)
**New Hampshire**	1,402 (1,159 to 2,058)	1,926 (1,492 to 2,552)	2,370 (1,773 to 3,133)	.7 (.37 to 1)	.23 (.11 to .38)	119 (99 to 173)	111 (87 to 148)	114 (84 to 149)	-.03 (-.24 to .15)	.03 (-.08 to .16)
**New Jersey**	14,727 (10,632 to 18,257)	15,855 (11,545 to 19,244)	17,803 (11,990 to 21,985)	.21 (-.02 to .41)	.12 (-.01 to .27)	161 (117 to 201)	140 (102 to 170)	139 (93 to 172)	-.14 (-.31 to .02)	-.01 (-.13 to .13)
**New Mexico**	2,013 (1,653 to 2,862)	3,473 (2,673 to 4,463)	4,397 (3,173 to 5,369)	1.21 (.65 to 1.67)	.27 (.08 to .46)	133 (109 to 189)	144 (111 to 184)	156 (108 to 191)	.18 (-.15 to .45)	.08 (-.1 to .26)
**New York**	39,888 (26,021 to 46,622)	47,026 (26,274 to 57,468)	53,275 (27,763 to 68,942)	.32 (-.09 to .64)	.13 (-.03 to .28)	192 (126 to 225)	192 (106 to 235)	194 (99 to 253)	0 (-.33 to .25)	0 (-.14 to .15)
**North Carolina**	12,238 (8,904 to 15,004)	16,933 (12,771 to 21,451)	22,155 (15,565 to 27,246)	.81 (.59 to 1.05)	.31 (.15 to .48)	168 (122 to 207)	154 (116 to 195)	162 (111 to 196)	-.03 (-.17 to .1)	.05 (-.09 to .19)
**North Dakota**	778 (614 to 1,252)	900 (750 to 1,364)	1,067 (866 to 1,580)	.39 (.16 to .63)	.19 (.04 to .34)	101 (80 to 165)	99 (83 to 152)	105 (85 to 155)	.06 (-.15 to .26)	.07 (-.08 to .21)

© 2018 Global Burden of Cardiovascular Diseases Collaboration. *JAMA Cardiology*.

**Ohio**	20,682 (14,644 to 24,618)	28,831 (18,441 to 33,468)	33,422 (20,229 to 40,281)	.61 (.26 to .92)	.16 (.04 to .29)	165 (118 to 200)	192 (122 to 224)	204 (120 to 249)	.23 (-.08 to .49)	.05 (-.07 to .19)
**Oklahoma**	4,586 (3,750 to 7,101)	7,548 (5,777 to 10,135)	11,561 (6,956 to 13,718)	1.62 (.41 to 2.41)	.54 (.12 to .88)	124 (101 to 193)	167 (127 to 223)	226 (134 to 269)	.91 (-.01 to 1.5)	.36 (-.02 to .66)
**Oregon**	3,629 (2,922 to 5,810)	4,701 (3,744 to 7,606)	5,291 (4,092 to 8,839)	.45 (.28 to .67)	.12 (.02 to .23)	106 (85 to 173)	95 (75 to 155)	88 (67 to 147)	-.18 (-.27 to -.06)	-.07 (-.16 to .01)
**Pennsylvania**	20,198 (16,562 to 28,638)	22,146 (18,660 to 31,405)	26,530 (20,233 to 33,933)	.33 (.08 to .53)	.21 (.01 to .39)	132 (109 to 191)	126 (105 to 177)	138 (102 to 175)	.06 (-.17 to .25)	.11 (-.08 to .28)
**Rhode Island**	1,442 (1,182 to 2,241)	1,608 (1,346 to 2,339)	1,868 (1,458 to 2,574)	.31 (.03 to .57)	.17 (-.03 to .38)	117 (96 to 182)	110 (92 to 160)	119 (91 to 161)	.04 (-.21 to .26)	.09 (-.1 to .29)
**South Carolina**	6,498 (4,706 to 8,240)	9,176 (6,837 to 12,136)	11,765 (8,653 to 15,408)	.81 (.59 to 1.1)	.28 (.14 to .45)	179 (129 to 229)	168 (126 to 222)	172 (124 to 220)	-.04 (-.17 to .11)	.02 (-.1 to .16)
**South Dakota**	787 (567 to 1,407)	992 (756 to 1,680)	1,228 (947 to 1,987)	.6 (.29 to .9)	.25 (.09 to .42)	92 (66 to 168)	93 (70 to 159)	100 (77 to 161)	.12 (-.13 to .35)	.09 (-.06 to .25)
**Tennessee**	10,634 (6,994 to 12,369)	16,960 (10,150 to 19,848)	21,725 (12,083 to 26,391)	1.03 (.54 to 1.35)	.28 (.14 to .42)	191 (126 to 223)	222 (132 to 260)	242 (131 to 296)	.26 (-.08 to .48)	.08 (-.05 to .22)
**Texas**	29,013 (19,810 to 34,046)	47,573 (29,605 to 55,604)	62,925 (36,392 to 76,477)	1.16 (.74 to 1.5)	.32 (.17 to .47)	178 (121 to 209)	189 (118 to 220)	192 (111 to 234)	.07 (-.14 to .24)	.01 (-.1 to .13)
**Utah**	1,705 (1,359 to 2,364)	2,703 (2,196 to 3,760)	3,588 (2,867 to 4,950)	1.11 (.89 to 1.34)	.33 (.22 to .46)	121 (96 to 167)	114 (93 to 160)	115 (92 to 157)	-.05 (-.15 to .06)	.01 (-.08 to .11)
**Vermont**	831 (643 to 1,118)	1,017 (748 to 1,268)	1,225 (850 to 1,483)	.48 (.22 to .71)	.21 (.07 to .35)	136 (105 to 183)	118 (86 to 147)	124 (84 to 149)	-.09 (-.26 to .07)	.04 (-.08 to .18)
**Virginia**	8,629 (6,854 to 12,275)	11,444 (9,459 to 16,302)	13,787 (10,993 to 18,846)	.61 (.38 to .81)	.21 (.08 to .34)	136 (108 to 193)	123 (102 to 175)	123 (97 to 165)	-.09 (-.23 to .03)	0 (-.12 to .13)
**Washington**	6,144 (5,065 to 8,880)	10,043 (7,187 to 12,358)	11,858 (8,541 to 14,892)	.95 (.5 to 1.33)	.18 (.07 to .31)	117 (96 to 170)	126 (90 to 157)	119 (86 to 150)	.03 (-.22 to .23)	-.06 (-.15 to .04)

© 2018 Global Burden of Cardiovascular Diseases Collaboration. *JAMA Cardiology*.

**West Virginia**	3,316 (2,655 to 4,640)	3,738 (3,029 to 5,760)	4,257 (3,432 to 6,479)	.28 (.12 to .52)	.14 (.04 to .26)	145 (117 to 208)	144 (118 to 218)	158 (127 to 230)	.08 (-.07 to .24)	.1 (-.03 to .23)
**Wisconsin**	6,730 (5,543 to 9,909)	8,759 (6,966 to 11,757)	10,481 (8,112 to 13,772)	.57 (.26 to .83)	.2 (.08 to .32)	117 (97 to 175)	119 (94 to 161)	124 (94 to 162)	.08 (-.16 to .27)	.05 (-.07 to .17)
**Wyoming**	466 (360 to 788)	738 (597 to 1,147)	881 (699 to 1,337)	.93 (.55 to 1.26)	.2 (.05 to .37)	104 (80 to 174)	112 (91 to 174)	113 (90 to 169)	.12 (-.12 to .32)	.02 (-.11 to .17)
**Intracerebral Hemorrhage**										
**Alabama**	22,664 (21,226 to 24,234)	30,315 (28,317 to 32,329)	33,326 (29,597 to 37,421)	.47 (.29 to .67)	.1 (-.03 to .25)	510 (478 to 546)	531 (496 to 567)	513 (456 to 577)	.01 (-.12 to .15)	-.03 (-.15 to .1)
**Alaska**	1,279 (1,189 to 1,380)	1,991 (1,848 to 2,148)	2,382 (2,097 to 2,690)	.86 (.62 to 1.14)	.2 (.05 to .36)	335 (311 to 364)	311 (288 to 334)	301 (266 to 340)	-.1 (-.21 to .03)	-.03 (-.14 to .09)
**Arizona**	11,835 (10,973 to 12,733)	19,714 (18,413 to 21,122)	23,187 (20,848 to 25,585)	.96 (.75 to 1.19)	.18 (.06 to .31)	297 (276 to 319)	275 (257 to 294)	249 (224 to 275)	-.16 (-.25 to -.06)	-.09 (-.18 to 0)
**Arkansas**	12,551 (11,734 to 13,370)	15,950 (14,993 to 16,935)	17,784 (16,149 to 19,637)	.42 (.28 to .57)	.12 (.01 to .22)	461 (431 to 491)	458 (430 to 485)	452 (410 to 498)	-.02 (-.11 to .09)	-.01 (-.11 to .08)
**California**	106,395 (98,500 to 114,773)	122,217 (113,875 to 130,687)	131,266 (117,461 to 145,864)	.24 (.09 to .39)	.07 (-.03 to .2)	361 (335 to 388)	304 (283 to 325)	265 (237 to 295)	-.27 (-.35 to -.17)	-.13 (-.22 to -.03)
**Colorado**	9,064 (8,447 to 9,708)	13,431 (12,443 to 14,393)	16,224 (14,731 to 17,864)	.79 (.61 to 1.01)	.21 (.1 to .34)	279 (260 to 299)	255 (237 to 274)	235 (213 to 257)	-.16 (-.24 to -.06)	-.08 (-.16 to .02)
**Connecticut**	11,166 (10,324 to 11,972)	10,824 (9,954 to 11,695)	11,363 (10,010 to 12,774)	.02 (-.1 to .16)	.05 (-.07 to .18)	292 (271 to 313)	235 (216 to 255)	221 (194 to 248)	-.24 (-.33 to -.14)	-.06 (-.17 to .05)
**Delaware**	2,495 (2,312 to 2,685)	3,291 (3,067 to 3,538)	3,880 (3,533 to 4,210)	.56 (.41 to .73)	.18 (.07 to .3)	345 (320 to 371)	302 (282 to 324)	284 (259 to 308)	-.18 (-.25 to -.09)	-.06 (-.15 to .03)
**District of Columbia**	4,715 (4,139 to 5,674)	2,813 (2,493 to 3,292)	2,381 (2,042 to 2,837)	-.49 (-.56 to -.42)	-.15 (-.25 to -.04)	716 (631 to 863)	430 (381 to 503)	314 (269 to 375)	-.56 (-.62 to -.5)	-.27 (-.35 to -.17)

© 2018 Global Burden of Cardiovascular Diseases Collaboration. *JAMA Cardiology*.

**Florida**	55,379 (51,522 to 59,821)	73,508 (68,510 to 79,060)	84,401 (75,626 to 93,411)	.53 (.36 to .7)	.15 (.04 to .27)	342 (319 to 370)	299 (279 to 322)	277 (247 to 307)	-.19 (-.28 to -.1)	-.07 (-.17 to .02)
**Georgia**	32,925 (30,611 to 35,562)	45,489 (42,356 to 49,163)	54,258 (47,916 to 61,249)	.65 (.45 to .88)	.19 (.06 to .35)	514 (478 to 555)	456 (425 to 491)	430 (380 to 485)	-.16 (-.26 to -.05)	-.06 (-.16 to .06)
**Hawaii**	4,557 (4,237 to 4,931)	5,640 (5,278 to 6,035)	6,166 (5,623 to 6,729)	.35 (.22 to .5)	.09 (0 to .19)	396 (368 to 429)	337 (315 to 360)	312 (284 to 341)	-.21 (-.29 to -.13)	-.07 (-.16 to .01)
**Idaho**	2,960 (2,731 to 3,182)	4,575 (4,239 to 4,905)	5,856 (5,179 to 6,597)	.98 (.73 to 1.26)	.28 (.14 to .45)	280 (258 to 301)	270 (250 to 289)	271 (240 to 307)	-.03 (-.15 to .11)	0 (-.11 to .14)
**Illinois**	48,513 (45,250 to 51,792)	47,135 (43,951 to 50,188)	49,287 (44,948 to 53,541)	.02 (-.08 to .12)	.05 (-.04 to .14)	390 (363 to 417)	314 (293 to 335)	291 (266 to 316)	-.25 (-.32 to -.18)	-.07 (-.15 to .01)
**Indiana**	22,082 (20,555 to 23,625)	25,919 (24,203 to 27,710)	29,946 (26,710 to 33,522)	.36 (.19 to .53)	.16 (.03 to .29)	362 (337 to 387)	342 (319 to 365)	346 (309 to 387)	-.04 (-.16 to .08)	.01 (-.1 to .13)
**Iowa**	9,389 (8,759 to 10,070)	10,672 (9,919 to 11,396)	11,720 (10,607 to 12,920)	.25 (.11 to .39)	.1 (0 to .22)	275 (257 to 293)	268 (249 to 287)	266 (240 to 293)	-.03 (-.14 to .07)	-.01 (-.1 to .1)
**Kansas**	8,508 (7,909 to 9,116)	10,678 (9,899 to 11,399)	11,764 (10,422 to 13,284)	.38 (.22 to .57)	.1 (-.04 to .25)	304 (283 to 325)	313 (291 to 335)	308 (273 to 350)	.01 (-.11 to .15)	-.02 (-.14 to .12)
**Kentucky**	15,158 (14,199 to 16,233)	18,870 (17,641 to 20,143)	22,048 (19,917 to 24,114)	.46 (.32 to .61)	.17 (.06 to .28)	373 (349 to 399)	365 (341 to 389)	373 (337 to 408)	0 (-.09 to .1)	.02 (-.07 to .12)
**Louisiana**	21,097 (19,558 to 22,751)	23,949 (22,250 to 25,842)	26,722 (24,383 to 29,585)	.27 (.15 to .4)	.12 (.02 to .22)	498 (462 to 536)	472 (438 to 510)	450 (412 to 501)	-.09 (-.18 to 0)	-.04 (-.13 to .04)
**Maine**	4,015 (3,715 to 4,304)	4,830 (4,441 to 5,205)	5,473 (4,929 to 6,016)	.36 (.22 to .51)	.13 (.03 to .25)	283 (263 to 303)	263 (241 to 283)	257 (232 to 282)	-.09 (-.18 to .01)	-.02 (-.11 to .07)
**Maryland**	19,073 (17,651 to 20,979)	21,083 (19,488 to 23,014)	22,586 (20,478 to 24,951)	.19 (.07 to .31)	.07 (-.02 to .18)	380 (352 to 418)	313 (289 to 342)	283 (257 to 315)	-.25 (-.32 to -.18)	-.09 (-.17 to -.01)
**Massachusetts**	20,282 (18,927 to 21,720)	19,842 (18,233 to 21,361)	21,801 (19,524 to 24,183)	.08 (-.04 to .19)	.1 (0 to .21)	293 (274 to 314)	238 (220 to 257)	227 (203 to 252)	-.23 (-.31 to -.14)	-.05 (-.13 to .05)

© 2018 Global Burden of Cardiovascular Diseases Collaboration. *JAMA Cardiology*.

**Michigan**	35,575 (33,428 to 38,055)	37,519 (35,029 to 40,217)	40,482 (36,866 to 44,345)	.14 (.03 to .26)	.08 (-.01 to .18)	359 (338 to 384)	303 (284 to 325)	296 (270 to 324)	-.18 (-.25 to -.09)	-.03 (-.11 to .07)
**Minnesota**	13,800 (12,839 to 14,740)	16,298 (14,944 to 17,556)	18,801 (16,732 to 20,747)	.36 (.22 to .52)	.15 (.05 to .27)	288 (268 to 307)	257 (235 to 278)	249 (220 to 275)	-.14 (-.23 to -.03)	-.03 (-.12 to .06)
**Mississippi**	14,181 (13,248 to 15,316)	17,959 (16,645 to 19,576)	19,902 (17,546 to 22,454)	.4 (.23 to .59)	.11 (-.02 to .25)	529 (493 to 570)	529 (489 to 577)	522 (460 to 591)	-.01 (-.14 to .12)	-.01 (-.13 to .11)
**Missouri**	20,970 (19,524 to 22,379)	24,704 (23,092 to 26,283)	27,732 (25,261 to 30,074)	.32 (.19 to .45)	.12 (.02 to .23)	358 (334 to 381)	340 (318 to 362)	335 (305 to 363)	-.06 (-.16 to .02)	-.01 (-.1 to .08)
**Montana**	2,639 (2,437 to 2,860)	3,346 (3,093 to 3,610)	3,808 (3,350 to 4,278)	.44 (.26 to .65)	.14 (.01 to .29)	293 (271 to 316)	267 (246 to 288)	253 (222 to 285)	-.13 (-.25 to -.01)	-.05 (-.16 to .07)
**Nebraska**	5,444 (5,068 to 5,830)	6,406 (5,917 to 6,889)	7,215 (6,550 to 7,912)	.33 (.2 to .47)	.13 (.03 to .23)	301 (281 to 323)	290 (267 to 311)	286 (259 to 313)	-.05 (-.14 to .05)	-.01 (-.1 to .08)
**Nevada**	4,849 (4,416 to 5,339)	10,682 (9,871 to 11,704)	12,544 (11,251 to 14,116)	1.59 (1.32 to 1.88)	.18 (.06 to .3)	386 (352 to 425)	370 (342 to 405)	327 (294 to 367)	-.15 (-.24 to -.06)	-.11 (-.2 to -.02)
**New Hampshire**	3,256 (3,018 to 3,494)	3,585 (3,320 to 3,859)	4,347 (3,908 to 4,842)	.34 (.2 to .49)	.21 (.09 to .34)	279 (258 to 298)	217 (200 to 234)	219 (196 to 243)	-.21 (-.3 to -.12)	.01 (-.09 to .12)
**New Jersey**	32,043 (30,029 to 34,208)	29,952 (28,035 to 31,992)	31,500 (28,422 to 34,721)	-.02 (-.12 to .1)	.05 (-.05 to .17)	361 (339 to 385)	274 (257 to 293)	255 (230 to 281)	-.29 (-.37 to -.21)	-.07 (-.16 to .03)
**New Mexico**	4,438 (4,133 to 4,814)	6,196 (5,741 to 6,670)	7,255 (6,450 to 8,193)	.64 (.43 to .86)	.17 (.04 to .33)	293 (273 to 317)	263 (244 to 283)	263 (233 to 296)	-.1 (-.22 to .03)	0 (-.12 to .13)
**New York**	72,410 (67,628 to 77,245)	54,802 (51,028 to 58,654)	56,952 (50,669 to 64,203)	-.21 (-.3 to -.11)	.04 (-.07 to .16)	360 (336 to 384)	232 (217 to 248)	216 (192 to 242)	-.4 (-.47 to -.33)	-.07 (-.17 to .04)
**North Carolina**	32,795 (30,638 to 34,989)	42,546 (40,012 to 45,233)	49,851 (45,781 to 54,182)	.52 (.38 to .66)	.17 (.07 to .28)	456 (426 to 486)	396 (372 to 420)	369 (340 to 400)	-.19 (-.26 to -.11)	-.07 (-.15 to .02)
**North Dakota**	2,101 (1,940 to 2,265)	2,196 (2,012 to 2,370)	2,627 (2,351 to 2,944)	.25 (.11 to .41)	.2 (.07 to .33)	291 (269 to 314)	259 (238 to 279)	272 (242 to 306)	-.06 (-.17 to .05)	.05 (-.06 to .18)

© 2018 Global Burden of Cardiovascular Diseases Collaboration. *JAMA Cardiology*.

**Ohio**	41,531 (38,927 to 44,214)	48,704 (45,609 to 51,906)	53,556 (49,035 to 58,377)	.29 (.17 to .42)	.1 (.01 to .2)	342 (321 to 363)	335 (314 to 357)	333 (305 to 361)	-.03 (-.11 to .06)	-.01 (-.09 to .08)
**Oklahoma**	13,238 (12,367 to 14,052)	16,912 (15,847 to 17,990)	19,322 (17,631 to 21,059)	.46 (.32 to .61)	.14 (.04 to .25)	377 (353 to 401)	388 (364 to 412)	389 (356 to 423)	.03 (-.07 to .13)	0 (-.08 to .09)
**Oregon**	10,517 (9,805 to 11,209)	14,546 (13,540 to 15,528)	16,471 (14,876 to 18,095)	.57 (.41 to .73)	.13 (.03 to .24)	319 (298 to 340)	306 (284 to 326)	283 (255 to 311)	-.11 (-.2 to -.02)	-.07 (-.15 to .02)
**Pennsylvania**	49,683 (46,436 to 52,913)	52,841 (49,577 to 56,453)	54,498 (49,657 to 59,726)	.1 (0 to .2)	.03 (-.06 to .12)	344 (322 to 365)	311 (293 to 333)	290 (265 to 317)	-.15 (-.23 to -.08)	-.07 (-.15 to .02)
**Rhode Island**	3,428 (3,151 to 3,714)	3,377 (3,083 to 3,667)	3,568 (3,110 to 4,022)	.04 (-.08 to .18)	.06 (-.07 to .18)	288 (265 to 312)	240 (219 to 261)	235 (204 to 267)	-.18 (-.28 to -.07)	-.02 (-.14 to .09)
**South Carolina**	21,018 (19,614 to 22,508)	25,484 (23,815 to 27,253)	30,316 (27,132 to 33,814)	.44 (.27 to .63)	.19 (.06 to .34)	580 (541 to 622)	478 (447 to 510)	451 (404 to 504)	-.22 (-.32 to -.12)	-.06 (-.16 to .06)
**South Dakota**	2,377 (2,184 to 2,560)	2,718 (2,503 to 2,931)	3,191 (2,830 to 3,561)	.34 (.19 to .52)	.17 (.06 to .3)	298 (274 to 322)	272 (250 to 294)	278 (246 to 310)	-.07 (-.18 to .06)	.02 (-.09 to .13)
**Tennessee**	23,890 (22,413 to 25,527)	31,559 (29,565 to 33,749)	37,088 (33,874 to 40,375)	.55 (.4 to .7)	.18 (.07 to .28)	440 (413 to 470)	424 (397 to 453)	420 (384 to 458)	-.05 (-.14 to .05)	-.01 (-.1 to .08)
**Texas**	63,820 (59,443 to 68,760)	87,980 (82,322 to 94,069)	108,374 (98,187 to 118,890)	.7 (.53 to .88)	.23 (.12 to .35)	387 (360 to 417)	353 (331 to 377)	336 (305 to 368)	-.13 (-.22 to -.04)	-.05 (-.13 to .04)
**Utah**	4,093 (3,820 to 4,390)	6,339 (5,946 to 6,806)	8,420 (7,651 to 9,195)	1.06 (.84 to 1.29)	.33 (.21 to .45)	280 (261 to 301)	268 (252 to 288)	271 (246 to 295)	-.03 (-.13 to .08)	.01 (-.09 to .1)
**Vermont**	1,621 (1,504 to 1,742)	1,675 (1,546 to 1,812)	1,940 (1,745 to 2,137)	.2 (.08 to .33)	.16 (.04 to .27)	269 (250 to 288)	204 (188 to 220)	204 (184 to 225)	-.24 (-.31 to -.16)	0 (-.09 to .1)
**Virginia**	24,565 (23,079 to 26,199)	29,784 (27,769 to 31,577)	33,732 (30,647 to 36,732)	.37 (.24 to .51)	.13 (.03 to .25)	385 (362 to 410)	327 (306 to 347)	305 (277 to 331)	-.21 (-.28 to -.13)	-.07 (-.15 to .02)
**Washington**	15,850 (14,783 to 16,941)	21,008 (19,606 to 22,376)	24,142 (22,055 to 26,337)	.52 (.38 to .69)	.15 (.05 to .26)	306 (286 to 327)	275 (256 to 292)	250 (229 to 272)	-.18 (-.26 to -.1)	-.09 (-.17 to 0)

© 2018 Global Burden of Cardiovascular Diseases Collaboration. *JAMA Cardiology*.

**West Virginia**	7,854 (7,310 to 8,395)	9,123 (8,497 to 9,747)	10,269 (9,366 to 11,183)	.31 (.18 to .45)	.13 (.03 to .24)	362 (337 to 387)	365 (340 to 389)	384 (349 to 418)	.06 (-.04 to .17)	.05 (-.04 to .16)
**Wisconsin**	16,976 (15,812 to 18,083)	19,474 (18,241 to 20,714)	22,102 (20,356 to 24,070)	.3 (.19 to .44)	.14 (.04 to .24)	309 (288 to 329)	276 (258 to 293)	272 (250 to 295)	-.12 (-.2 to -.03)	-.01 (-.1 to .07)
**Wyoming**	1,305 (1,207 to 1,405)	1,787 (1,650 to 1,920)	1,989 (1,758 to 2,233)	.53 (.35 to .73)	.11 (-.01 to .25)	290 (269 to 313)	282 (261 to 303)	262 (232 to 295)	-.1 (-.2 to .03)	-.07 (-.17 to .04)
**Ischemic heart disease**										
**Alabama**	178,117 (169,726 to 186,437)	158,680 (151,439 to 166,070)	165,656 (146,862 to 184,186)	-.07 (-.18 to .04)	.04 (-.08 to .17)	3,778 (3,596 to 3,959)	2,661 (2,539 to 2,790)	2,386 (2,112 to 2,660)	-.37 (-.45 to -.29)	-.1 (-.21 to .01)
**Alaska**	8,339 (7,859 to 8,854)	9,746 (9,118 to 10,371)	11,985 (10,457 to 13,562)	.44 (.24 to .65)	.23 (.06 to .43)	2,936 (2,780 to 3,109)	1,620 (1,524 to 1,718)	1,487 (1,308 to 1,673)	-.49 (-.56 to -.42)	-.08 (-.2 to .06)
**Arizona**	124,129 (118,866 to 129,460)	135,582 (129,702 to 141,915)	148,189 (135,035 to 161,538)	.19 (.09 to .31)	.09 (-.01 to .19)	2,990 (2,861 to 3,123)	1,806 (1,729 to 1,892)	1,458 (1,323 to 1,594)	-.51 (-.56 to -.46)	-.19 (-.27 to -.12)
**Arkansas**	114,818 (110,185 to 119,875)	103,688 (99,087 to 108,223)	108,871 (99,684 to 118,038)	-.05 (-.14 to .04)	.05 (-.04 to .15)	3,817 (3,659 to 3,988)	2,795 (2,666 to 2,917)	2,571 (2,345 to 2,796)	-.33 (-.39 to -.26)	-.08 (-.17 to .01)
**California**	877,887 (833,450 to 921,783)	738,985 (705,015 to 775,654)	740,401 (668,292 to 814,383)	-.16 (-.25 to -.06)	0 (-.1 to .12)	2,994 (2,840 to 3,147)	1,803 (1,720 to 1,892)	1,402 (1,262 to 1,545)	-.53 (-.58 to -.48)	-.22 (-.3 to -.13)
**Colorado**	83,199 (79,402 to 86,706)	77,099 (73,357 to 80,602)	85,799 (78,873 to 93,152)	.03 (-.06 to .13)	.11 (.02 to .22)	2,607 (2,490 to 2,715)	1,461 (1,390 to 1,527)	1,185 (1,090 to 1,287)	-.55 (-.58 to -.5)	-.19 (-.26 to -.11)
**Connecticut**	118,689 (112,874 to 124,225)	78,712 (74,542 to 82,543)	72,934 (65,692 to 81,332)	-.39 (-.45 to -.31)	-.07 (-.17 to .03)	2,920 (2,772 to 3,057)	1,578 (1,492 to 1,656)	1,291 (1,156 to 1,447)	-.56 (-.61 to -.5)	-.18 (-.27 to -.08)
**Delaware**	26,392 (25,300 to 27,630)	23,856 (22,800 to 24,889)	24,479 (22,664 to 26,261)	-.07 (-.15 to .01)	.03 (-.05 to .11)	3,559 (3,410 to 3,725)	2,099 (2,007 to 2,193)	1,660 (1,537 to 1,783)	-.53 (-.57 to -.49)	-.21 (-.27 to -.14)
**District of Columbia**	25,769 (23,416 to 29,471)	19,106 (17,624 to 21,516)	15,684 (13,602 to 18,277)	-.39 (-.46 to -.32)	-.18 (-.27 to -.08)	3,771 (3,423 to 4,326)	2,851 (2,628 to 3,218)	2,030 (1,757 to 2,380)	-.46 (-.52 to -.39)	-.29 (-.37 to -.2)

© 2018 Global Burden of Cardiovascular Diseases Collaboration. *JAMA Cardiology*.

**Florida**	587,490 (561,954 to 615,767)	524,421 (500,102 to 549,612)	546,526 (496,864 to 596,261)	-.07 (-.15 to .02)	.04 (-.05 to .14)	3,049 (2,904 to 3,210)	1,899 (1,806 to 1,998)	1,565 (1,418 to 1,718)	-.49 (-.53 to -.43)	-.18 (-.25 to -.1)
**Georgia**	235,844 (223,766 to 250,165)	222,498 (210,683 to 237,545)	249,265 (224,858 to 279,140)	.06 (-.06 to .2)	.12 (.01 to .27)	3,754 (3,564 to 3,981)	2,259 (2,141 to 2,409)	1,912 (1,722 to 2,140)	-.49 (-.55 to -.42)	-.15 (-.24 to -.04)
**Hawaii**	26,920 (25,606 to 28,337)	26,199 (24,993 to 27,499)	28,310 (26,195 to 30,399)	.05 (-.03 to .15)	.08 (0 to .16)	2,342 (2,229 to 2,464)	1,488 (1,419 to 1,562)	1,318 (1,215 to 1,421)	-.44 (-.49 to -.38)	-.11 (-.19 to -.04)
**Idaho**	29,662 (28,099 to 31,178)	28,703 (27,036 to 30,277)	32,677 (29,092 to 36,605)	.1 (-.02 to .24)	.14 (0 to .28)	2,697 (2,553 to 2,836)	1,639 (1,543 to 1,730)	1,412 (1,254 to 1,588)	-.48 (-.54 to -.41)	-.14 (-.24 to -.03)
**Illinois**	475,688 (456,586 to 495,729)	329,657 (315,799 to 344,753)	309,473 (286,906 to 331,921)	-.35 (-.4 to -.29)	-.06 (-.13 to .02)	3,631 (3,479 to 3,788)	2,108 (2,018 to 2,205)	1,708 (1,581 to 1,837)	-.53 (-.56 to -.49)	-.19 (-.25 to -.12)
**Indiana**	228,397 (218,118 to 238,142)	182,839 (173,614 to 191,862)	189,237 (170,395 to 212,492)	-.17 (-.26 to -.07)	.04 (-.07 to .16)	3,570 (3,409 to 3,726)	2,305 (2,186 to 2,421)	2,043 (1,840 to 2,301)	-.43 (-.49 to -.36)	-.11 (-.21 to 0)
**Iowa**	117,393 (112,489 to 122,100)	80,939 (77,145 to 84,860)	80,516 (73,007 to 87,946)	-.31 (-.37 to -.24)	0 (-.1 to .09)	3,095 (2,957 to 3,223)	1,886 (1,792 to 1,977)	1,680 (1,517 to 1,845)	-.46 (-.51 to -.4)	-.11 (-.19 to -.02)
**Kansas**	92,426 (88,020 to 96,399)	69,343 (65,633 to 72,827)	69,326 (61,990 to 77,503)	-.25 (-.33 to -.15)	0 (-.12 to .12)	3,011 (2,863 to 3,145)	1,912 (1,806 to 2,012)	1,680 (1,496 to 1,887)	-.44 (-.51 to -.36)	-.12 (-.23 to -.01)
**Kentucky**	168,778 (162,368 to 174,937)	144,253 (137,839 to 150,398)	150,671 (138,890 to 162,501)	-.11 (-.18 to -.03)	.04 (-.04 to .14)	3,977 (3,822 to 4,123)	2,688 (2,567 to 2,805)	2,404 (2,212 to 2,594)	-.4 (-.44 to -.34)	-.11 (-.18 to -.03)
**Louisiana**	177,792 (169,663 to 187,956)	140,938 (133,663 to 149,893)	150,091 (138,801 to 163,596)	-.16 (-.22 to -.08)	.07 (-.02 to .15)	4,102 (3,914 to 4,338)	2,701 (2,559 to 2,879)	2,401 (2,216 to 2,627)	-.41 (-.46 to -.36)	-.11 (-.18 to -.04)
**Maine**	48,837 (46,818 to 50,755)	32,743 (31,022 to 34,292)	32,416 (29,940 to 35,138)	-.34 (-.39 to -.27)	-.01 (-.09 to .08)	3,265 (3,126 to 3,397)	1,671 (1,581 to 1,751)	1,398 (1,287 to 1,524)	-.57 (-.61 to -.53)	-.16 (-.23 to -.08)
**Maryland**	160,880 (153,073 to 171,760)	139,370 (132,564 to 148,978)	137,841 (126,505 to 151,114)	-.14 (-.21 to -.07)	-.01 (-.09 to .07)	3,260 (3,102 to 3,479)	2,031 (1,932 to 2,170)	1,625 (1,488 to 1,789)	-.5 (-.54 to -.46)	-.2 (-.26 to -.13)
**Massachusetts**	227,062 (217,190 to 236,416)	144,097 (136,144 to 151,273)	137,774 (126,094 to 149,513)	-.39 (-.45 to -.34)	-.04 (-.11 to .04)	3,102 (2,965 to 3,235)	1,637 (1,541 to 1,724)	1,337 (1,217 to 1,456)	-.57 (-.61 to -.53)	-.18 (-.25 to -.11)

© 2018 Global Burden of Cardiovascular Diseases Collaboration. *JAMA Cardiology*.

**Michigan**	384,426 (370,127 to 399,443)	301,216 (289,102 to 313,950)	311,109 (290,111 to 333,134)	-.19 (-.25 to -.13)	.03 (-.04 to .11)	3,753 (3,613 to 3,900)	2,317 (2,223 to 2,416)	2,071 (1,924 to 2,225)	-.45 (-.49 to -.41)	-.11 (-.17 to -.04)
**Minnesota**	135,828 (129,705 to 141,666)	85,302 (80,459 to 89,394)	87,718 (78,790 to 96,537)	-.35 (-.42 to -.28)	.03 (-.06 to .14)	2,692 (2,567 to 2,810)	1,285 (1,211 to 1,349)	1,089 (970 to 1,206)	-.6 (-.64 to -.55)	-.15 (-.23 to -.06)
**Mississippi**	127,162 (121,007 to 134,133)	109,571 (104,005 to 116,586)	110,972 (98,920 to 123,820)	-.13 (-.23 to -.02)	.01 (-.1 to .13)	4,450 (4,227 to 4,701)	3,107 (2,946 to 3,308)	2,725 (2,421 to 3,046)	-.39 (-.46 to -.31)	-.12 (-.22 to -.02)
**Missouri**	225,935 (217,679 to 234,896)	183,327 (175,972 to 190,648)	190,606 (176,801 to 203,473)	-.16 (-.22 to -.09)	.04 (-.05 to .13)	3,524 (3,390 to 3,669)	2,373 (2,275 to 2,469)	2,137 (1,978 to 2,286)	-.39 (-.44 to -.35)	-.1 (-.17 to -.02)
**Montana**	25,749 (24,388 to 27,040)	22,575 (21,305 to 23,851)	23,477 (20,784 to 26,280)	-.09 (-.2 to .03)	.04 (-.08 to .17)	2,677 (2,535 to 2,814)	1,683 (1,586 to 1,783)	1,424 (1,255 to 1,599)	-.47 (-.54 to -.39)	-.15 (-.25 to -.04)
**Nebraska**	60,287 (57,839 to 62,835)	37,944 (36,092 to 39,618)	37,457 (34,391 to 40,372)	-.38 (-.42 to -.33)	-.01 (-.09 to .07)	2,994 (2,868 to 3,123)	1,596 (1,514 to 1,670)	1,372 (1,255 to 1,485)	-.54 (-.58 to -.5)	-.14 (-.21 to -.07)
**Nevada**	41,564 (39,098 to 45,002)	62,422 (58,739 to 67,372)	71,617 (64,956 to 79,271)	.72 (.56 to .89)	.15 (.04 to .26)	3,413 (3,214 to 3,677)	2,161 (2,036 to 2,329)	1,771 (1,603 to 1,964)	-.48 (-.53 to -.43)	-.18 (-.25 to -.1)
**New Hampshire**	37,536 (36,032 to 39,060)	28,208 (26,849 to 29,582)	28,720 (26,256 to 31,227)	-.23 (-.3 to -.17)	.02 (-.07 to .11)	3,175 (3,043 to 3,305)	1,628 (1,547 to 1,712)	1,322 (1,199 to 1,445)	-.58 (-.62 to -.55)	-.19 (-.26 to -.11)
**New Jersey**	321,171 (309,084 to 334,159)	218,105 (208,689 to 227,481)	208,825 (192,591 to 226,300)	-.35 (-.41 to -.29)	-.04 (-.12 to .04)	3,417 (3,287 to 3,559)	1,877 (1,792 to 1,960)	1,550 (1,426 to 1,684)	-.55 (-.59 to -.5)	-.17 (-.24 to -.1)
**New Mexico**	40,589 (38,670 to 42,729)	42,285 (40,299 to 44,425)	46,258 (41,096 to 51,576)	.14 (0 to .28)	.09 (-.03 to .23)	2,662 (2,534 to 2,804)	1,724 (1,644 to 1,812)	1,520 (1,342 to 1,701)	-.43 (-.5 to -.35)	-.12 (-.22 to -.01)
**New York**	845,708 (808,689 to 881,262)	564,976 (540,049 to 591,719)	522,794 (470,533 to 578,739)	-.38 (-.44 to -.31)	-.07 (-.17 to .03)	3,916 (3,743 to 4,084)	2,219 (2,117 to 2,327)	1,783 (1,597 to 1,986)	-.54 (-.59 to -.49)	-.2 (-.28 to -.1)
**North Carolina**	269,864 (258,737 to 282,086)	231,158 (220,791 to 241,844)	249,501 (232,554 to 268,821)	-.08 (-.15 to 0)	.08 (0 to .17)	3,647 (3,494 to 3,813)	2,090 (1,995 to 2,186)	1,745 (1,623 to 1,881)	-.52 (-.56 to -.48)	-.16 (-.23 to -.09)
**North Dakota**	23,683 (22,452 to 24,830)	15,976 (15,103 to 16,769)	15,931 (14,277 to 17,725)	-.33 (-.4 to -.24)	0 (-.11 to .11)	2,934 (2,769 to 3,088)	1,717 (1,618 to 1,804)	1,524 (1,354 to 1,708)	-.48 (-.54 to -.41)	-.11 (-.21 to 0)

© 2018 Global Burden of Cardiovascular Diseases Collaboration. *JAMA Cardiology*.

**Ohio**	472,793 (455,276 to 491,140)	346,104 (332,919 to 361,078)	338,677 (315,308 to 363,269)	-.28 (-.34 to -.23)	-.02 (-.09 to .06)	3,694 (3,556 to 3,839)	2,255 (2,165 to 2,354)	1,951 (1,814 to 2,095)	-.47 (-.51 to -.43)	-.13 (-.2 to -.06)
**Oklahoma**	149,288 (144,208 to 154,813)	135,785 (129,840 to 141,399)	141,349 (131,851 to 151,291)	-.05 (-.12 to .02)	.04 (-.04 to .12)	3,938 (3,798 to 4,089)	2,940 (2,811 to 3,063)	2,656 (2,472 to 2,847)	-.33 (-.38 to -.27)	-.1 (-.17 to -.03)
**Oregon**	101,173 (97,027 to 105,183)	75,072 (71,204 to 78,429)	75,215 (69,441 to 80,684)	-.26 (-.32 to -.19)	0 (-.08 to .08)	2,908 (2,791 to 3,027)	1,509 (1,429 to 1,579)	1,206 (1,108 to 1,297)	-.59 (-.62 to -.55)	-.2 (-.27 to -.13)
**Pennsylvania**	570,975 (550,352 to 591,700)	385,413 (369,935 to 400,927)	357,853 (334,678 to 382,671)	-.37 (-.42 to -.32)	-.07 (-.14 to 0)	3,583 (3,450 to 3,719)	2,088 (2,002 to 2,176)	1,736 (1,618 to 1,865)	-.52 (-.55 to -.48)	-.17 (-.23 to -.1)
**Rhode Island**	44,503 (42,397 to 46,440)	30,926 (29,192 to 32,459)	26,979 (24,257 to 30,129)	-.39 (-.46 to -.32)	-.13 (-.22 to -.03)	3,400 (3,236 to 3,553)	2,025 (1,905 to 2,128)	1,611 (1,438 to 1,808)	-.53 (-.58 to -.47)	-.2 (-.29 to -.1)
**South Carolina**	143,998 (136,915 to 151,080)	127,900 (121,605 to 133,934)	144,368 (130,562 to 158,830)	0 (-.11 to .12)	.13 (.01 to .26)	3,938 (3,745 to 4,127)	2,320 (2,207 to 2,432)	2,024 (1,827 to 2,231)	-.49 (-.54 to -.42)	-.13 (-.22 to -.03)
**South Dakota**	27,883 (26,448 to 29,245)	20,275 (19,101 to 21,392)	20,846 (18,734 to 23,058)	-.25 (-.33 to -.17)	.03 (-.08 to .14)	3,095 (2,931 to 3,255)	1,862 (1,745 to 1,970)	1,628 (1,449 to 1,813)	-.47 (-.53 to -.41)	-.13 (-.22 to -.03)
**Tennessee**	223,098 (214,471 to 232,238)	204,634 (196,152 to 214,869)	224,821 (207,122 to 240,756)	.01 (-.07 to .09)	.1 (.01 to .18)	3,947 (3,790 to 4,112)	2,648 (2,539 to 2,779)	2,393 (2,198 to 2,563)	-.39 (-.44 to -.34)	-.1 (-.17 to -.03)
**Texas**	556,778 (533,401 to 584,304)	530,870 (508,128 to 555,611)	601,120 (550,131 to 645,417)	.08 (-.02 to .18)	.13 (.03 to .23)	3,412 (3,270 to 3,585)	2,150 (2,058 to 2,251)	1,815 (1,663 to 1,950)	-.47 (-.52 to -.42)	-.16 (-.23 to -.09)
**Utah**	35,458 (34,027 to 37,165)	34,305 (32,714 to 36,084)	40,333 (37,404 to 43,549)	.14 (.05 to .24)	.18 (.09 to .28)	2,494 (2,392 to 2,616)	1,468 (1,400 to 1,546)	1,280 (1,186 to 1,384)	-.49 (-.53 to -.44)	-.13 (-.19 to -.05)
**Vermont**	18,928 (18,135 to 19,776)	13,211 (12,579 to 13,843)	13,601 (12,507 to 14,759)	-.28 (-.34 to -.22)	.03 (-.06 to .12)	3,055 (2,927 to 3,191)	1,516 (1,440 to 1,589)	1,297 (1,189 to 1,412)	-.58 (-.61 to -.54)	-.14 (-.22 to -.06)
**Virginia**	212,763 (205,179 to 221,519)	177,391 (170,168 to 184,515)	182,416 (169,337 to 196,993)	-.14 (-.21 to -.07)	.03 (-.05 to .11)	3,359 (3,241 to 3,499)	1,914 (1,835 to 1,990)	1,569 (1,454 to 1,698)	-.53 (-.57 to -.49)	-.18 (-.24 to -.11)
**Washington**	145,587 (140,049 to 151,748)	121,015 (115,770 to 125,984)	126,659 (117,008 to 136,558)	-.13 (-.2 to -.05)	.05 (-.03 to .14)	2,751 (2,642 to 2,867)	1,540 (1,470 to 1,604)	1,240 (1,144 to 1,340)	-.55 (-.59 to -.51)	-.19 (-.26 to -.12)

© 2018 Global Burden of Cardiovascular Diseases Collaboration. *JAMA Cardiology*.

**West Virginia**	98,907 (94,895 to 102,945)	71,439 (68,048 to 74,676)	69,955 (64,497 to 75,515)	-.29 (-.35 to -.23)	-.02 (-.1 to .06)	4,188 (4,017 to 4,367)	2,658 (2,530 to 2,782)	2,396 (2,210 to 2,591)	-.43 (-.48 to -.37)	-.1 (-.17 to -.02)
**Wisconsin**	182,701 (176,082 to 189,395)	127,594 (122,502 to 132,757)	131,371 (122,115 to 140,317)	-.28 (-.33 to -.22)	.03 (-.05 to .11)	3,083 (2,967 to 3,200)	1,710 (1,640 to 1,783)	1,491 (1,381 to 1,598)	-.52 (-.55 to -.48)	-.13 (-.2 to -.06)
**Wyoming**	12,581 (11,963 to 13,248)	12,252 (11,582 to 12,911)	12,679 (11,275 to 14,189)	.01 (-.11 to .14)	.04 (-.08 to .17)	2,798 (2,659 to 2,946)	1,854 (1,753 to 1,957)	1,563 (1,386 to 1,754)	-.44 (-.51 to -.37)	-.16 (-.26 to -.05)
**Ischemic stroke**										
**Alabama**	30,848 (27,801 to 34,170)	36,632 (32,794 to 40,346)	40,364 (35,622 to 45,412)	.31 (.18 to .45)	.1 (0 to .21)	626 (565 to 692)	594 (531 to 655)	546 (481 to 615)	-.13 (-.22 to -.04)	-.08 (-.16 to .01)
**Alaska**	1,304 (1,154 to 1,452)	2,403 (2,085 to 2,691)	3,011 (2,607 to 3,410)	1.31 (1.09 to 1.55)	.25 (.13 to .38)	533 (474 to 593)	477 (418 to 530)	419 (365 to 473)	-.21 (-.29 to -.14)	-.12 (-.2 to -.03)
**Arizona**	18,595 (16,406 to 20,816)	30,431 (26,738 to 34,034)	36,255 (31,017 to 41,301)	.95 (.79 to 1.11)	.19 (.1 to .29)	430 (380 to 480)	390 (343 to 437)	335 (286 to 382)	-.22 (-.28 to -.16)	-.14 (-.21 to -.07)
**Arkansas**	21,520 (19,284 to 23,723)	22,430 (19,986 to 24,910)	24,823 (21,983 to 27,873)	.15 (.06 to .26)	.11 (.02 to .2)	660 (592 to 728)	570 (508 to 634)	542 (480 to 607)	-.18 (-.25 to -.11)	-.05 (-.12 to .03)
**California**	152,209 (135,607 to 168,527)	164,208 (143,021 to 184,023)	184,248 (160,092 to 212,519)	.21 (.1 to .32)	.12 (.04 to .22)	508 (453 to 562)	394 (343 to 442)	342 (296 to 396)	-.33 (-.39 to -.27)	-.13 (-.2 to -.05)
**Colorado**	13,882 (12,265 to 15,490)	20,456 (18,145 to 22,695)	24,458 (21,371 to 27,451)	.76 (.62 to .91)	.2 (.12 to .28)	429 (379 to 479)	393 (348 to 436)	337 (294 to 379)	-.21 (-.28 to -.15)	-.14 (-.2 to -.08)
**Connecticut**	16,904 (14,747 to 19,065)	17,818 (15,321 to 20,119)	18,157 (15,683 to 20,872)	.07 (-.03 to .18)	.02 (-.06 to .11)	399 (347 to 448)	339 (290 to 383)	306 (262 to 354)	-.23 (-.31 to -.16)	-.1 (-.17 to -.01)
**Delaware**	3,442 (3,039 to 3,854)	4,678 (4,085 to 5,248)	5,477 (4,752 to 6,181)	.59 (.46 to .74)	.17 (.09 to .27)	457 (403 to 511)	398 (348 to 448)	352 (304 to 399)	-.23 (-.29 to -.16)	-.12 (-.18 to -.04)
**District of Columbia**	4,079 (3,619 to 4,682)	2,532 (2,181 to 2,893)	2,329 (1,956 to 2,698)	-.43 (-.49 to -.37)	-.08 (-.16 to 0)	570 (507 to 655)	367 (315 to 419)	295 (247 to 343)	-.48 (-.54 to -.43)	-.2 (-.27 to -.12)

© 2018 Global Burden of Cardiovascular Diseases Collaboration. *JAMA Cardiology*.

**Florida**	88,804 (78,144 to 99,954)	109,177 (94,538 to 123,084)	129,062 (111,393 to 148,010)	.45 (.33 to .58)	.18 (.08 to .28)	428 (377 to 482)	369 (321 to 417)	341 (292 to 391)	-.2 (-.27 to -.14)	-.08 (-.15 to 0)
**Georgia**	42,004 (37,880 to 46,556)	52,335 (46,380 to 57,731)	63,822 (56,029 to 72,167)	.52 (.38 to .66)	.22 (.12 to .33)	664 (599 to 736)	550 (488 to 608)	492 (433 to 557)	-.26 (-.33 to -.19)	-.1 (-.18 to -.02)
**Hawaii**	5,101 (4,499 to 5,706)	7,023 (6,102 to 7,886)	8,007 (7,012 to 9,015)	.57 (.45 to .7)	.14 (.06 to .22)	442 (390 to 493)	373 (325 to 419)	332 (288 to 375)	-.25 (-.31 to -.18)	-.11 (-.18 to -.05)
**Idaho**	5,649 (4,996 to 6,340)	8,184 (7,251 to 9,104)	9,711 (8,562 to 10,977)	.72 (.55 to .9)	.19 (.08 to .3)	491 (434 to 550)	454 (400 to 506)	404 (353 to 457)	-.18 (-.26 to -.09)	-.11 (-.19 to -.03)
**Illinois**	68,366 (60,936 to 76,325)	70,038 (60,906 to 78,298)	72,341 (63,343 to 81,340)	.06 (-.02 to .15)	.03 (-.04 to .11)	500 (445 to 557)	430 (374 to 480)	384 (336 to 432)	-.23 (-.29 to -.17)	-.11 (-.17 to -.04)
**Indiana**	35,997 (32,157 to 40,117)	37,690 (33,035 to 42,153)	41,713 (36,570 to 47,165)	.16 (.06 to .28)	.11 (.01 to .22)	539 (482 to 600)	457 (401 to 512)	430 (376 to 487)	-.2 (-.27 to -.12)	-.06 (-.14 to .03)
**Iowa**	19,237 (17,114 to 21,350)	19,894 (17,528 to 22,177)	20,274 (17,733 to 22,775)	.05 (-.03 to .15)	.02 (-.05 to .11)	458 (407 to 509)	417 (367 to 468)	387 (337 to 436)	-.16 (-.23 to -.08)	-.07 (-.14 to 0)
**Kansas**	15,590 (13,829 to 17,461)	17,553 (15,573 to 19,650)	18,419 (16,150 to 20,780)	.18 (.06 to .3)	.05 (-.05 to .15)	467 (413 to 523)	448 (396 to 501)	415 (363 to 470)	-.11 (-.2 to -.02)	-.07 (-.16 to .02)
**Kentucky**	24,330 (21,760 to 26,938)	26,415 (23,304 to 29,311)	29,333 (25,820 to 32,766)	.21 (.11 to .3)	.11 (.03 to .2)	548 (491 to 606)	480 (423 to 533)	447 (392 to 500)	-.18 (-.25 to -.12)	-.07 (-.14 to 0)
**Louisiana**	27,082 (24,212 to 30,094)	29,529 (26,255 to 32,625)	33,271 (29,667 to 37,056)	.23 (.13 to .34)	.13 (.05 to .22)	607 (544 to 674)	559 (498 to 618)	515 (459 to 573)	-.15 (-.22 to -.08)	-.08 (-.14 to -.01)
**Maine**	6,980 (6,177 to 7,857)	8,092 (6,995 to 9,083)	8,902 (7,727 to 10,002)	.28 (.16 to .39)	.1 (.02 to .19)	442 (391 to 496)	393 (340 to 443)	358 (309 to 403)	-.19 (-.26 to -.12)	-.09 (-.16 to -.02)
**Maryland**	24,648 (21,729 to 27,460)	28,513 (24,994 to 31,794)	31,622 (27,439 to 35,996)	.28 (.17 to .39)	.11 (.03 to .19)	498 (440 to 554)	412 (360 to 460)	365 (316 to 418)	-.27 (-.33 to -.2)	-.11 (-.18 to -.04)
**Massachusetts**	32,762 (28,867 to 36,407)	33,603 (29,147 to 37,816)	35,375 (30,394 to 39,972)	.08 (-.01 to .17)	.05 (-.02 to .13)	417 (367 to 465)	358 (309 to 404)	325 (278 to 368)	-.22 (-.29 to -.15)	-.09 (-.16 to -.02)

© 2018 Global Burden of Cardiovascular Diseases Collaboration. *JAMA Cardiology*.

**Michigan**	52,685 (46,849 to 58,483)	56,228 (49,046 to 62,602)	61,023 (52,952 to 68,741)	.16 (.07 to .25)	.09 (.01 to .16)	501 (445 to 556)	418 (364 to 466)	389 (337 to 439)	-.22 (-.28 to -.16)	-.07 (-.13 to 0)
**Minnesota**	26,280 (23,382 to 29,329)	26,843 (23,487 to 30,124)	30,336 (26,087 to 34,200)	.15 (.05 to .26)	.13 (.05 to .22)	477 (424 to 533)	382 (332 to 429)	355 (305 to 402)	-.25 (-.32 to -.19)	-.07 (-.14 to .01)
**Mississippi**	19,048 (17,280 to 20,998)	20,499 (18,309 to 22,695)	23,050 (20,173 to 25,704)	.21 (.09 to .34)	.13 (.02 to .23)	631 (572 to 695)	569 (508 to 630)	539 (472 to 603)	-.14 (-.23 to -.05)	-.05 (-.14 to .04)
**Missouri**	33,628 (29,748 to 37,433)	36,312 (31,790 to 40,504)	39,665 (34,897 to 44,343)	.18 (.09 to .28)	.09 (.01 to .18)	490 (435 to 545)	446 (390 to 499)	416 (366 to 468)	-.15 (-.22 to -.08)	-.07 (-.14 to 0)
**Montana**	4,786 (4,223 to 5,384)	5,702 (5,041 to 6,412)	6,437 (5,582 to 7,268)	.35 (.22 to .48)	.13 (.03 to .23)	474 (418 to 533)	405 (357 to 457)	369 (319 to 418)	-.22 (-.3 to -.15)	-.09 (-.17 to -.01)
**Nebraska**	10,378 (9,186 to 11,534)	10,841 (9,569 to 12,132)	11,502 (10,116 to 12,820)	.11 (.03 to .2)	.06 (-.01 to .14)	472 (419 to 525)	424 (373 to 474)	395 (344 to 441)	-.16 (-.23 to -.09)	-.07 (-.13 to 0)
**Nevada**	5,847 (5,147 to 6,517)	12,711 (11,162 to 14,244)	16,023 (13,902 to 18,150)	1.74 (1.52 to 1.99)	.26 (.17 to .36)	498 (440 to 555)	458 (403 to 512)	396 (343 to 449)	-.21 (-.27 to -.14)	-.14 (-.2 to -.07)
**New Hampshire**	5,421 (4,775 to 6,087)	6,105 (5,231 to 6,924)	7,102 (6,128 to 8,103)	.31 (.19 to .43)	.16 (.08 to .27)	442 (389 to 497)	347 (298 to 394)	317 (272 to 362)	-.28 (-.35 to -.22)	-.09 (-.16 to 0)
**New Jersey**	42,018 (37,034 to 47,087)	40,497 (34,807 to 46,033)	43,057 (36,859 to 49,549)	.02 (-.06 to .11)	.06 (-.01 to .16)	436 (385 to 488)	338 (290 to 385)	311 (265 to 358)	-.29 (-.35 to -.23)	-.08 (-.15 to 0)
**New Mexico**	6,257 (5,537 to 6,987)	8,857 (7,728 to 10,028)	10,721 (9,243 to 12,268)	.71 (.55 to .88)	.21 (.1 to .33)	405 (359 to 452)	357 (312 to 405)	336 (289 to 385)	-.17 (-.25 to -.09)	-.06 (-.14 to .03)
**New York**	83,853 (73,466 to 94,339)	66,434 (56,759 to 76,417)	73,107 (61,516 to 84,995)	-.13 (-.22 to -.04)	.1 (0 to .2)	377 (331 to 423)	255 (219 to 295)	245 (205 to 285)	-.35 (-.42 to -.28)	-.04 (-.13 to .05)
**North Carolina**	50,173 (45,092 to 55,050)	62,646 (55,572 to 69,157)	74,515 (66,125 to 83,306)	.49 (.38 to .6)	.19 (.11 to .27)	663 (596 to 727)	557 (494 to 615)	500 (444 to 560)	-.24 (-.3 to -.19)	-.1 (-.16 to -.04)
**North Dakota**	3,877 (3,442 to 4,306)	3,969 (3,452 to 4,443)	4,104 (3,514 to 4,650)	.06 (-.03 to .15)	.03 (-.05 to .12)	435 (387 to 485)	384 (333 to 431)	360 (307 to 410)	-.17 (-.24 to -.09)	-.06 (-.14 to .02)

© 2018 Global Burden of Cardiovascular Diseases Collaboration. *JAMA Cardiology*.

**Ohio**	63,208 (56,034 to 70,895)	72,500 (63,423 to 81,050)	77,738 (68,454 to 86,318)	.23 (.12 to .33)	.07 (0 to .15)	476 (421 to 534)	449 (391 to 503)	420 (369 to 468)	-.12 (-.19 to -.05)	-.06 (-.13 to 0)
**Oklahoma**	22,205 (19,861 to 24,756)	25,713 (22,916 to 28,412)	27,615 (24,418 to 30,686)	.24 (.14 to .35)	.07 (-.01 to .15)	548 (490 to 610)	535 (475 to 590)	492 (433 to 548)	-.1 (-.17 to -.03)	-.08 (-.15 to -.01)
**Oregon**	20,747 (18,656 to 22,860)	24,913 (22,283 to 27,737)	26,551 (23,382 to 29,603)	.28 (.17 to .39)	.07 (-.01 to .15)	559 (502 to 615)	474 (422 to 528)	405 (357 to 454)	-.27 (-.34 to -.21)	-.14 (-.21 to -.08)
**Pennsylvania**	77,600 (68,788 to 86,500)	82,364 (72,346 to 92,124)	82,655 (72,523 to 93,152)	.07 (-.02 to .16)	0 (-.07 to .08)	462 (410 to 517)	411 (360 to 461)	372 (326 to 420)	-.19 (-.26 to -.13)	-.09 (-.16 to -.03)
**Rhode Island**	5,304 (4,674 to 5,992)	5,328 (4,617 to 6,052)	5,285 (4,459 to 6,032)	0 (-.1 to .09)	-.01 (-.09 to .08)	381 (335 to 429)	327 (281 to 372)	300 (251 to 345)	-.21 (-.29 to -.14)	-.08 (-.16 to 0)
**South Carolina**	27,187 (24,695 to 29,786)	31,732 (28,371 to 35,081)	39,045 (34,581 to 43,498)	.44 (.3 to .58)	.23 (.12 to .34)	736 (670 to 805)	567 (506 to 628)	518 (458 to 577)	-.3 (-.36 to -.23)	-.09 (-.16 to -.01)
**South Dakota**	4,275 (3,781 to 4,782)	4,713 (4,097 to 5,268)	5,175 (4,490 to 5,858)	.21 (.09 to .33)	.1 (.01 to .19)	432 (382 to 483)	390 (336 to 437)	367 (317 to 419)	-.15 (-.23 to -.07)	-.06 (-.13 to .02)
**Tennessee**	38,465 (34,511 to 42,349)	43,935 (38,928 to 48,705)	50,282 (44,567 to 55,622)	.31 (.2 to .41)	.15 (.07 to .23)	652 (585 to 719)	557 (494 to 617)	511 (452 to 566)	-.22 (-.28 to -.16)	-.08 (-.15 to -.02)
**Texas**	88,707 (79,300 to 98,473)	114,563 (100,533 to 127,749)	142,559 (124,877 to 159,088)	.61 (.48 to .75)	.24 (.16 to .34)	531 (476 to 590)	469 (412 to 522)	431 (376 to 480)	-.19 (-.26 to -.12)	-.08 (-.15 to -.01)
**Utah**	6,912 (6,161 to 7,682)	10,558 (9,411 to 11,783)	13,333 (11,825 to 14,840)	.93 (.77 to 1.1)	.26 (.17 to .36)	474 (422 to 526)	447 (398 to 501)	421 (373 to 470)	-.11 (-.18 to -.03)	-.06 (-.13 to .02)
**Vermont**	2,816 (2,492 to 3,155)	3,153 (2,747 to 3,584)	3,613 (3,128 to 4,078)	.28 (.17 to .4)	.15 (.06 to .24)	433 (383 to 486)	351 (304 to 400)	328 (283 to 372)	-.24 (-.31 to -.18)	-.06 (-.14 to .01)
**Virginia**	36,459 (32,643 to 40,032)	43,015 (37,903 to 48,026)	48,587 (42,815 to 54,571)	.33 (.23 to .44)	.13 (.06 to .21)	572 (512 to 629)	465 (410 to 519)	409 (359 to 460)	-.29 (-.34 to -.23)	-.12 (-.18 to -.06)
**Washington**	28,069 (24,924 to 30,965)	36,785 (32,376 to 41,005)	40,442 (35,471 to 45,341)	.44 (.33 to .56)	.1 (.02 to .19)	510 (453 to 563)	456 (400 to 510)	386 (338 to 434)	-.24 (-.3 to -.18)	-.15 (-.21 to -.09)

© 2018 Global Burden of Cardiovascular Diseases Collaboration. *JAMA Cardiology*.

**West Virginia**	12,359 (11,038 to 13,722)	13,280 (11,654 to 14,768)	14,147 (12,513 to 15,759)	.15 (.05 to .23)	.07 (-.01 to .15)	497 (445 to 552)	467 (410 to 520)	447 (394 to 499)	-.1 (-.17 to -.03)	-.04 (-.11 to .03)
**Wisconsin**	30,224 (26,998 to 33,482)	33,285 (29,159 to 37,320)	35,088 (30,598 to 39,446)	.16 (.07 to .25)	.06 (-.03 to .14)	475 (424 to 528)	418 (364 to 470)	376 (326 to 422)	-.21 (-.27 to -.15)	-.1 (-.17 to -.03)
**Wyoming**	2,071 (1,836 to 2,316)	2,703 (2,363 to 3,025)	2,944 (2,528 to 3,323)	.42 (.28 to .56)	.09 (0 to .19)	453 (402 to 507)	406 (354 to 453)	353 (303 to 399)	-.22 (-.3 to -.15)	-.13 (-.2 to -.05)
**Myocarditis**										
**Alabama**	520 (401 to 848)	917 (580 to 1,101)	915 (606 to 1,141)	.84 (.12 to 1.5)	0 (-.14 to .17)	13 (10 to 21)	21 (13 to 25)	20 (13 to 25)	.61 (-.02 to 1.19)	-.03 (-.17 to .13)
**Alaska**	79 (63 to 113)	126 (74 to 161)	131 (80 to 172)	.68 (-.03 to 1.32)	.05 (-.12 to .22)	14 (11 to 20)	19 (11 to 24)	18 (11 to 24)	.34 (-.22 to .83)	-.03 (-.18 to .12)
**Arizona**	409 (321 to 615)	981 (713 to 1,148)	974 (733 to 1,148)	1.48 (.4 to 2.31)	0 (-.14 to .17)	11 (9 to 17)	17 (12 to 20)	15 (11 to 18)	.41 (-.21 to .88)	-.11 (-.23 to .04)
**Arkansas**	297 (236 to 453)	570 (373 to 688)	567 (390 to 689)	.98 (.24 to 1.64)	0 (-.14 to .15)	13 (10 to 20)	21 (14 to 26)	20 (14 to 25)	.62 (0 to 1.2)	-.04 (-.19 to .1)
**California**	3,445 (2,716 to 5,046)	4,613 (3,193 to 5,403)	4,412 (3,012 to 5,557)	.32 (-.26 to .7)	-.04 (-.18 to .13)	11 (9 to 16)	13 (9 to 15)	12 (8 to 15)	.08 (-.42 to .4)	-.11 (-.23 to .05)
**Colorado**	377 (313 to 511)	738 (443 to 893)	813 (513 to 999)	1.2 (.31 to 1.96)	.11 (-.03 to .26)	12 (10 to 15)	16 (9 to 19)	15 (9 to 18)	.33 (-.22 to .78)	-.04 (-.16 to .08)
**Connecticut**	341 (262 to 492)	435 (294 to 511)	415 (292 to 511)	.27 (-.26 to .69)	-.04 (-.18 to .11)	10 (8 to 15)	13 (9 to 15)	12 (8 to 15)	.21 (-.32 to .62)	-.06 (-.19 to .08)
**Delaware**	87 (72 to 118)	151 (85 to 192)	156 (94 to 197)	.82 (.11 to 1.45)	.04 (-.1 to .18)	13 (11 to 18)	18 (10 to 23)	17 (10 to 22)	.33 (-.21 to .79)	-.03 (-.17 to .1)
**District of Columbia**	138 (74 to 395)	115 (66 to 172)	93 (75 to 117)	-.22 (-.73 to .16)	-.17 (-.38 to .23)	21 (11 to 61)	20 (12 to 29)	14 (11 to 17)	-.25 (-.74 to .13)	-.29 (-.47 to .03)

© 2018 Global Burden of Cardiovascular Diseases Collaboration. *JAMA Cardiology*.

**Florida**	1,540 (1,242 to 2,294)	3,024 (2,016 to 3,586)	2,942 (2,167 to 3,483)	.98 (.19 to 1.58)	-.02 (-.18 to .2)	12 (10 to 19)	18 (12 to 22)	16 (12 to 19)	.34 (-.22 to .77)	-.12 (-.26 to .09)
**Georgia**	927 (753 to 1,327)	1,861 (1,139 to 2,299)	1,945 (1,288 to 2,435)	1.15 (.17 to 1.88)	.05 (-.1 to .25)	14 (12 to 20)	21 (13 to 25)	20 (13 to 24)	.42 (-.24 to .9)	-.04 (-.18 to .14)
**Hawaii**	109 (79 to 142)	187 (86 to 244)	190 (92 to 251)	.75 (.03 to 1.43)	.03 (-.11 to .17)	10 (7 to 13)	14 (7 to 19)	13 (6 to 17)	.38 (-.19 to .9)	-.06 (-.18 to .07)
**Idaho**	100 (79 to 140)	186 (126 to 220)	204 (143 to 245)	1.11 (.22 to 1.82)	.1 (-.05 to .26)	10 (8 to 14)	13 (9 to 15)	13 (9 to 15)	.29 (-.26 to .73)	-.03 (-.16 to .12)
**Illinois**	1,504 (1,217 to 2,205)	2,090 (1,462 to 2,458)	2,049 (1,438 to 2,420)	.41 (-.26 to .87)	-.02 (-.14 to .14)	13 (11 to 19)	17 (12 to 20)	17 (12 to 20)	.33 (-.32 to .75)	-.02 (-.15 to .14)
**Indiana**	620 (490 to 890)	1,113 (735 to 1,344)	1,144 (783 to 1,413)	.92 (.13 to 1.64)	.03 (-.11 to .17)	11 (9 to 16)	18 (12 to 22)	18 (13 to 22)	.66 (-.01 to 1.28)	-.01 (-.15 to .13)
**Iowa**	293 (240 to 410)	420 (256 to 508)	437 (272 to 531)	.53 (-.11 to 1.05)	.05 (-.09 to .18)	11 (9 to 15)	15 (9 to 18)	14 (9 to 18)	.37 (-.23 to .84)	0 (-.13 to .13)
**Kansas**	278 (231 to 362)	471 (295 to 585)	468 (308 to 588)	.73 (.04 to 1.37)	0 (-.14 to .16)	11 (9 to 15)	17 (11 to 22)	17 (11 to 21)	.5 (-.11 to 1.05)	-.03 (-.18 to .11)
**Kentucky**	422 (320 to 634)	725 (549 to 841)	751 (577 to 910)	.87 (.15 to 1.49)	.04 (-.09 to .19)	12 (9 to 18)	18 (14 to 21)	18 (14 to 21)	.6 (-.01 to 1.14)	0 (-.13 to .14)
**Louisiana**	557 (429 to 938)	882 (515 to 1,084)	882 (577 to 1,106)	.66 (.04 to 1.22)	.01 (-.14 to .17)	13 (10 to 22)	21 (12 to 26)	20 (13 to 25)	.59 (-.01 to 1.14)	-.06 (-.19 to .11)
**Maine**	124 (101 to 171)	176 (118 to 213)	179 (126 to 219)	.49 (-.08 to 1.01)	.02 (-.11 to .16)	10 (8 to 14)	14 (9 to 17)	15 (10 to 18)	.47 (-.1 to 1.02)	.04 (-.1 to .19)
**Maryland**	724 (514 to 946)	1,247 (648 to 1,621)	1,234 (685 to 1,621)	.71 (-.08 to 1.3)	0 (-.13 to .16)	15 (11 to 20)	22 (12 to 29)	21 (12 to 27)	.39 (-.25 to .85)	-.07 (-.19 to .08)
**Massachusetts**	706 (584 to 1,017)	936 (551 to 1,143)	1,019 (619 to 1,285)	.48 (-.1 to .97)	.09 (-.04 to .23)	12 (10 to 17)	15 (8 to 18)	15 (9 to 19)	.31 (-.22 to .74)	.02 (-.1 to .15)

© 2018 Global Burden of Cardiovascular Diseases Collaboration. *JAMA Cardiology*.

**Michigan**	1,183 (954 to 1,733)	1,658 (1,118 to 1,968)	1,620 (1,164 to 1,914)	.42 (-.11 to .89)	-.02 (-.14 to .1)	13 (10 to 19)	17 (12 to 21)	17 (12 to 21)	.41 (-.13 to .89)	.01 (-.12 to .13)
**Minnesota**	430 (350 to 623)	696 (378 to 850)	732 (414 to 910)	.75 (-.04 to 1.37)	.05 (-.07 to .18)	10 (8 to 14)	14 (7 to 17)	14 (8 to 17)	.4 (-.25 to .9)	0 (-.12 to .12)
**Mississippi**	326 (245 to 593)	566 (340 to 757)	533 (355 to 748)	.72 (.09 to 1.33)	-.05 (-.2 to .12)	13 (10 to 23)	20 (12 to 27)	19 (13 to 27)	.58 (0 to 1.16)	-.05 (-.2 to .12)
**Missouri**	599 (465 to 901)	968 (710 to 1,131)	961 (726 to 1,133)	.68 (.05 to 1.27)	0 (-.13 to .12)	12 (9 to 18)	17 (13 to 20)	17 (13 to 20)	.47 (-.09 to .99)	-.04 (-.17 to .09)
**Montana**	113 (90 to 130)	186 (101 to 242)	186 (102 to 245)	.65 (.01 to 1.18)	.01 (-.13 to .17)	15 (12 to 17)	20 (11 to 26)	18 (11 to 24)	.25 (-.22 to .64)	-.07 (-.2 to .06)
**Nebraska**	182 (153 to 235)	271 (162 to 329)	278 (172 to 344)	.56 (-.1 to 1.09)	.03 (-.1 to .15)	12 (10 to 15)	15 (9 to 19)	15 (9 to 18)	.29 (-.27 to .74)	-.03 (-.15 to .09)
**Nevada**	122 (85 to 235)	338 (263 to 424)	352 (298 to 446)	2.1 (.65 to 3.08)	.05 (-.11 to .3)	10 (7 to 20)	14 (11 to 17)	12 (10 to 15)	.3 (-.32 to .71)	-.09 (-.23 to .12)
**New Hampshire**	106 (78 to 178)	139 (100 to 185)	151 (120 to 196)	.49 (-.03 to .97)	.09 (-.05 to .28)	10 (7 to 16)	11 (8 to 15)	12 (9 to 15)	.32 (-.16 to .78)	.08 (-.08 to .27)
**New Jersey**	966 (786 to 1,333)	1,371 (861 to 1,628)	1,311 (858 to 1,580)	.4 (-.23 to .87)	-.04 (-.17 to .09)	13 (10 to 17)	16 (10 to 19)	15 (10 to 18)	.26 (-.34 to .69)	-.06 (-.18 to .07)
**New Mexico**	178 (141 to 271)	301 (223 to 355)	307 (227 to 369)	.79 (.14 to 1.39)	.02 (-.13 to .2)	12 (9 to 18)	16 (12 to 19)	16 (12 to 19)	.39 (-.11 to .88)	-.01 (-.15 to .16)
**New York**	2,692 (2,127 to 3,885)	3,091 (2,053 to 3,946)	3,014 (2,022 to 4,201)	.16 (-.4 to .5)	-.02 (-.15 to .11)	15 (12 to 22)	17 (11 to 22)	16 (11 to 22)	.11 (-.44 to .44)	-.06 (-.17 to .07)
**North Carolina**	879 (735 to 1,178)	1,700 (1,009 to 2,113)	1,740 (1,140 to 2,162)	1.02 (.16 to 1.64)	.03 (-.12 to .22)	13 (11 to 18)	20 (12 to 25)	18 (12 to 23)	.39 (-.22 to .83)	-.07 (-.21 to .09)
**North Dakota**	78 (65 to 106)	104 (60 to 128)	126 (79 to 158)	.65 (.04 to 1.21)	.22 (.04 to .42)	12 (11 to 17)	16 (9 to 20)	16 (10 to 21)	.34 (-.16 to .79)	.01 (-.14 to .17)

© 2018 Global Burden of Cardiovascular Diseases Collaboration. *JAMA Cardiology*.

**Ohio**	1,362 (1,138 to 1,788)	2,086 (1,371 to 2,528)	2,098 (1,457 to 2,543)	.58 (0 to 1.12)	.01 (-.12 to .15)	13 (11 to 17)	19 (13 to 23)	19 (13 to 23)	.53 (-.02 to 1.07)	.01 (-.13 to .14)
**Oklahoma**	349 (256 to 562)	628 (476 to 755)	680 (529 to 887)	1.05 (.25 to 1.72)	.09 (-.07 to .26)	11 (8 to 18)	18 (14 to 22)	18 (14 to 24)	.72 (.05 to 1.3)	0 (-.15 to .17)
**Oregon**	276 (212 to 410)	453 (315 to 537)	470 (331 to 613)	.77 (.01 to 1.33)	.04 (-.09 to .18)	10 (8 to 15)	13 (9 to 15)	12 (8 to 15)	.23 (-.32 to .64)	-.06 (-.19 to .06)
**Pennsylvania**	1,257 (970 to 1,888)	1,763 (1,301 to 2,033)	1,715 (1,343 to 1,990)	.42 (-.08 to .88)	-.02 (-.15 to .12)	11 (8 to 16)	15 (11 to 17)	14 (11 to 16)	.37 (-.14 to .83)	-.06 (-.18 to .09)
**Rhode Island**	134 (110 to 209)	162 (96 to 199)	156 (96 to 201)	.19 (-.23 to .59)	-.03 (-.17 to .12)	14 (11 to 21)	16 (9 to 19)	15 (9 to 20)	.15 (-.28 to .56)	-.02 (-.15 to .13)
**South Carolina**	480 (384 to 688)	900 (494 to 1,140)	929 (555 to 1,187)	.98 (.15 to 1.67)	.04 (-.11 to .2)	14 (11 to 20)	21 (12 to 27)	20 (12 to 25)	.46 (-.16 to .97)	-.07 (-.2 to .07)
**South Dakota**	90 (74 to 122)	126 (86 to 150)	141 (97 to 173)	.6 (0 to 1.12)	.12 (-.04 to .27)	13 (11 to 18)	17 (12 to 20)	17 (12 to 21)	.34 (-.17 to .79)	.03 (-.12 to .18)
**Tennessee**	593 (471 to 886)	1,230 (824 to 1,465)	1,317 (919 to 1,569)	1.32 (.37 to 2.11)	.08 (-.07 to .25)	12 (10 to 19)	21 (14 to 25)	21 (15 to 25)	.75 (.03 to 1.34)	-.01 (-.15 to .14)
**Texas**	2,120 (1,661 to 3,033)	3,952 (2,747 to 4,605)	4,243 (3,130 to 4,954)	1.08 (.22 to 1.76)	.08 (-.07 to .22)	12 (10 to 17)	17 (12 to 20)	16 (12 to 19)	.35 (-.22 to .81)	-.07 (-.2 to .06)
**Utah**	208 (169 to 245)	427 (221 to 547)	487 (264 to 632)	1.34 (.46 to 2.12)	.15 (.01 to .28)	12 (9 to 14)	17 (9 to 21)	16 (9 to 21)	.39 (-.13 to .85)	-.02 (-.13 to .1)
**Vermont**	60 (51 to 82)	81 (51 to 100)	79 (52 to 99)	.34 (-.16 to .81)	-.02 (-.15 to .11)	11 (9 to 15)	14 (9 to 17)	13 (9 to 17)	.24 (-.24 to .67)	-.04 (-.16 to .09)
**Virginia**	675 (526 to 974)	1,116 (781 to 1,302)	1,141 (829 to 1,334)	.77 (-.02 to 1.37)	.03 (-.1 to .19)	11 (9 to 16)	15 (10 to 17)	14 (10 to 16)	.32 (-.29 to .77)	-.06 (-.18 to .08)
**Washington**	465 (354 to 679)	756 (528 to 892)	815 (571 to 1,014)	.83 (.04 to 1.4)	.08 (-.06 to .22)	10 (7 to 14)	12 (8 to 14)	12 (8 to 14)	.25 (-.32 to .66)	-.05 (-.16 to .08)

© 2018 Global Burden of Cardiovascular Diseases Collaboration. *JAMA Cardiology*.

**West Virginia**	235 (199 to 314)	376 (231 to 463)	388 (234 to 484)	.69 (-.06 to 1.28)	.03 (-.1 to .19)	14 (12 to 18)	22 (14 to 27)	23 (14 to 29)	.71 (-.05 to 1.33)	.05 (-.09 to .21)
**Wisconsin**	487 (401 to 661)	808 (484 to 985)	817 (516 to 1,004)	.73 (-.01 to 1.35)	.02 (-.11 to .16)	10 (8 to 14)	15 (9 to 18)	15 (9 to 18)	.47 (-.17 to 1)	-.01 (-.13 to .12)
**Wyoming**	60 (52 to 73)	100 (60 to 126)	104 (64 to 135)	.75 (.13 to 1.34)	.05 (-.1 to .21)	14 (12 to 17)	20 (12 to 25)	18 (11 to 24)	.36 (-.12 to .82)	-.06 (-.2 to .09)
**Other cardiomyopathy**										
**Alabama**	8,193 (6,217 to 9,255)	8,531 (7,625 to 10,243)	8,799 (7,464 to 11,697)	.09 (-.14 to .63)	.03 (-.11 to .23)	185 (140 to 208)	159 (143 to 190)	148 (125 to 194)	-.19 (-.36 to .2)	-.07 (-.2 to .09)
**Alaska**	565 (504 to 660)	693 (585 to 955)	822 (651 to 1,222)	.45 (.14 to .99)	.18 (.01 to .39)	138 (123 to 160)	108 (92 to 146)	106 (84 to 155)	-.24 (-.4 to .05)	-.03 (-.17 to .15)
**Arizona**	6,188 (4,667 to 6,935)	7,714 (6,810 to 9,753)	8,746 (7,374 to 11,721)	.44 (.1 to 1.18)	.13 (-.01 to .3)	157 (118 to 176)	114 (101 to 143)	104 (88 to 137)	-.32 (-.48 to .01)	-.09 (-.2 to .04)
**Arkansas**	3,891 (3,453 to 4,379)	4,525 (4,019 to 5,862)	4,733 (3,923 to 6,925)	.22 (-.01 to .72)	.04 (-.09 to .25)	144 (128 to 162)	137 (121 to 177)	130 (108 to 190)	-.1 (-.27 to .26)	-.06 (-.18 to .13)
**California**	59,360 (37,616 to 69,634)	53,219 (43,401 to 58,715)	54,330 (46,662 to 63,391)	-.05 (-.27 to .4)	.02 (-.12 to .19)	198 (125 to 233)	135 (111 to 149)	117 (99 to 136)	-.39 (-.53 to -.11)	-.13 (-.25 to 0)
**Colorado**	3,617 (3,253 to 4,343)	4,536 (3,862 to 6,468)	5,339 (4,302 to 8,220)	.47 (.19 to 1.02)	.17 (.03 to .35)	112 (101 to 133)	88 (75 to 126)	81 (65 to 125)	-.28 (-.42 to 0)	-.09 (-.19 to .05)
**Connecticut**	6,013 (4,334 to 6,781)	5,009 (4,377 to 5,757)	4,980 (4,281 to 6,162)	-.16 (-.33 to .17)	-.01 (-.14 to .16)	160 (115 to 181)	115 (102 to 133)	105 (90 to 129)	-.33 (-.47 to -.09)	-.09 (-.21 to .05)
**Delaware**	1,355 (980 to 1,540)	1,602 (1,325 to 1,773)	1,767 (1,553 to 1,996)	.32 (.11 to .71)	.11 (-.03 to .26)	189 (137 to 214)	155 (129 to 172)	142 (126 to 161)	-.24 (-.36 to -.01)	-.08 (-.19 to .04)
**District of Columbia**	2,400 (1,325 to 3,083)	1,260 (1,054 to 1,576)	1,158 (974 to 1,482)	-.49 (-.64 to -.13)	-.08 (-.22 to .09)	358 (196 to 460)	196 (164 to 245)	155 (131 to 197)	-.54 (-.67 to -.22)	-.21 (-.33 to -.06)

© 2018 Global Burden of Cardiovascular Diseases Collaboration. *JAMA Cardiology*.

**Florida**	31,786 (21,066 to 36,860)	35,391 (29,006 to 39,096)	39,360 (33,614 to 45,288)	.27 (.02 to .72)	.11 (-.02 to .26)	197 (132 to 229)	153 (128 to 169)	139 (120 to 160)	-.27 (-.42 to 0)	-.08 (-.2 to .04)
**Georgia**	14,274 (9,114 to 16,683)	15,170 (12,885 to 17,039)	16,222 (14,019 to 20,066)	.19 (-.12 to .91)	.07 (-.09 to .3)	222 (141 to 261)	157 (133 to 176)	138 (120 to 167)	-.35 (-.52 to .03)	-.12 (-.25 to .06)
**Hawaii**	2,588 (1,243 to 3,165)	2,643 (1,602 to 3,085)	2,914 (1,921 to 3,420)	.17 (-.05 to .63)	.11 (-.02 to .27)	223 (107 to 272)	165 (100 to 193)	156 (102 to 184)	-.27 (-.41 to .01)	-.05 (-.17 to .08)
**Idaho**	1,134 (1,018 to 1,358)	1,473 (1,259 to 2,046)	1,804 (1,468 to 2,604)	.59 (.29 to 1.09)	.22 (.07 to .39)	108 (97 to 130)	90 (77 to 124)	88 (72 to 127)	-.19 (-.34 to .07)	-.02 (-.15 to .11)
**Illinois**	25,393 (17,339 to 29,048)	21,204 (18,377 to 23,289)	20,954 (18,504 to 24,479)	-.15 (-.32 to .21)	-.01 (-.12 to .13)	206 (140 to 237)	148 (129 to 164)	134 (118 to 156)	-.33 (-.47 to -.05)	-.1 (-.2 to .02)
**Indiana**	10,495 (7,631 to 11,876)	11,237 (9,523 to 12,376)	12,677 (10,663 to 14,516)	.22 (.01 to .56)	.13 (-.01 to .27)	174 (127 to 197)	155 (132 to 171)	157 (133 to 179)	-.09 (-.25 to .17)	.01 (-.11 to .14)
**Iowa**	4,172 (3,512 to 4,616)	3,411 (2,957 to 4,555)	3,704 (3,085 to 5,236)	-.11 (-.28 to .31)	.08 (-.03 to .22)	127 (107 to 141)	92 (79 to 123)	91 (76 to 128)	-.28 (-.43 to .06)	-.01 (-.12 to .11)
**Kansas**	3,901 (3,083 to 4,334)	3,601 (3,215 to 4,520)	3,700 (3,031 to 5,170)	-.04 (-.27 to .46)	.02 (-.12 to .21)	141 (111 to 156)	111 (98 to 140)	103 (85 to 144)	-.26 (-.43 to .12)	-.07 (-.2 to .09)
**Kentucky**	6,038 (5,134 to 6,577)	6,402 (5,702 to 8,335)	6,922 (5,744 to 10,002)	.15 (-.07 to .68)	.08 (-.05 to .25)	150 (128 to 164)	130 (116 to 170)	126 (104 to 181)	-.16 (-.32 to .22)	-.04 (-.16 to .12)
**Louisiana**	9,069 (6,393 to 10,373)	9,059 (7,447 to 10,256)	9,700 (8,517 to 11,385)	.09 (-.11 to .5)	.07 (-.05 to .24)	211 (149 to 241)	188 (155 to 215)	175 (154 to 206)	-.15 (-.31 to .17)	-.07 (-.18 to .07)
**Maine**	1,933 (1,556 to 2,148)	1,820 (1,637 to 2,234)	1,903 (1,633 to 2,516)	0 (-.19 to .39)	.04 (-.08 to .2)	140 (113 to 155)	107 (96 to 131)	101 (88 to 132)	-.27 (-.4 to .02)	-.05 (-.16 to .08)
**Maryland**	13,424 (7,286 to 16,150)	9,879 (8,516 to 11,381)	10,829 (9,469 to 12,461)	-.13 (-.36 to .42)	.1 (-.01 to .21)	270 (147 to 324)	155 (134 to 179)	146 (128 to 169)	-.42 (-.58 to -.05)	-.06 (-.15 to .04)
**Massachusetts**	9,334 (7,664 to 10,282)	7,443 (6,476 to 9,978)	7,713 (6,416 to 11,309)	-.17 (-.34 to .22)	.03 (-.08 to .17)	138 (112 to 152)	93 (80 to 126)	86 (72 to 126)	-.37 (-.5 to -.07)	-.07 (-.18 to .04)

© 2018 Global Burden of Cardiovascular Diseases Collaboration. *JAMA Cardiology*.

**Michigan**	20,215 (13,477 to 23,316)	18,700 (15,473 to 20,644)	18,713 (16,674 to 21,273)	-.05 (-.24 to .36)	0 (-.11 to .17)	206 (137 to 238)	161 (134 to 178)	153 (134 to 173)	-.24 (-.38 to .07)	-.05 (-.15 to .08)
**Minnesota**	5,498 (4,663 to 6,158)	5,111 (4,458 to 6,919)	5,604 (4,624 to 7,892)	.02 (-.17 to .43)	.09 (-.03 to .24)	117 (99 to 131)	84 (73 to 114)	80 (66 to 112)	-.32 (-.45 to -.04)	-.05 (-.16 to .07)
**Mississippi**	5,778 (3,948 to 6,648)	5,545 (4,782 to 6,689)	5,797 (4,901 to 7,530)	.04 (-.22 to .62)	.05 (-.11 to .22)	213 (145 to 245)	172 (149 to 207)	164 (139 to 212)	-.21 (-.4 to .24)	-.05 (-.18 to .11)
**Missouri**	9,765 (7,525 to 10,927)	9,797 (8,820 to 11,109)	10,824 (9,721 to 12,901)	.12 (-.06 to .49)	.11 (-.01 to .23)	169 (130 to 189)	142 (129 to 161)	140 (126 to 169)	-.16 (-.3 to .13)	-.01 (-.11 to .1)
**Montana**	1,003 (906 to 1,182)	1,071 (885 to 1,526)	1,168 (894 to 1,848)	.16 (-.1 to .69)	.08 (-.06 to .27)	113 (102 to 134)	90 (74 to 130)	84 (64 to 132)	-.26 (-.43 to .08)	-.08 (-.2 to .07)
**Nebraska**	2,149 (1,930 to 2,439)	2,017 (1,744 to 2,716)	2,198 (1,819 to 3,149)	.02 (-.16 to .39)	.09 (-.03 to .21)	120 (108 to 137)	95 (82 to 130)	92 (76 to 133)	-.23 (-.37 to .05)	-.03 (-.14 to .08)
**Nevada**	2,739 (1,819 to 3,178)	4,765 (3,875 to 5,308)	4,910 (4,336 to 5,825)	.85 (.43 to 1.75)	.04 (-.12 to .29)	219 (145 to 253)	171 (139 to 191)	139 (123 to 161)	-.35 (-.49 to -.04)	-.19 (-.3 to 0)
**New Hampshire**	1,371 (1,216 to 1,600)	1,363 (1,159 to 1,908)	1,552 (1,260 to 2,296)	.13 (-.08 to .53)	.13 (.01 to .28)	119 (105 to 138)	87 (74 to 120)	86 (70 to 124)	-.28 (-.41 to -.04)	-.02 (-.12 to .1)
**New Jersey**	15,461 (11,217 to 17,505)	13,049 (11,530 to 14,527)	13,794 (12,058 to 16,182)	-.09 (-.26 to .21)	.06 (-.06 to .19)	176 (128 to 199)	126 (112 to 141)	120 (105 to 142)	-.31 (-.44 to -.08)	-.04 (-.15 to .07)
**New Mexico**	2,039 (1,744 to 2,249)	2,286 (1,961 to 3,110)	2,499 (1,965 to 3,784)	.23 (-.07 to .9)	.09 (-.08 to .29)	133 (114 to 147)	103 (88 to 140)	99 (78 to 150)	-.26 (-.44 to .15)	-.04 (-.19 to .14)
**New York**	30,241 (26,488 to 33,538)	22,363 (19,041 to 31,134)	23,284 (18,547 to 34,318)	-.23 (-.39 to .11)	.04 (-.09 to .18)	152 (133 to 168)	99 (84 to 138)	94 (75 to 138)	-.38 (-.51 to -.11)	-.05 (-.16 to .07)
**North Carolina**	13,092 (9,506 to 14,878)	14,768 (12,710 to 16,551)	15,880 (14,128 to 19,471)	.24 (.01 to .76)	.08 (-.05 to .28)	183 (133 to 208)	145 (125 to 162)	128 (114 to 155)	-.28 (-.42 to 0)	-.11 (-.21 to .04)
**North Dakota**	733 (646 to 930)	668 (554 to 990)	784 (602 to 1,225)	.06 (-.16 to .47)	.17 (.01 to .36)	102 (90 to 129)	83 (68 to 124)	85 (65 to 134)	-.18 (-.35 to .15)	.02 (-.12 to .18)

© 2018 Global Burden of Cardiovascular Diseases Collaboration. *JAMA Cardiology*.

**Ohio**	23,333 (15,776 to 26,785)	20,761 (18,423 to 23,108)	20,570 (18,260 to 24,664)	-.09 (-.28 to .38)	-.01 (-.12 to .16)	195 (132 to 223)	152 (136 to 170)	139 (123 to 167)	-.26 (-.42 to .12)	-.08 (-.19 to .07)
**Oklahoma**	5,272 (4,400 to 5,753)	5,761 (5,173 to 7,420)	5,946 (4,872 to 9,102)	.14 (-.11 to .81)	.02 (-.12 to .29)	152 (127 to 166)	140 (126 to 179)	130 (107 to 195)	-.14 (-.32 to .35)	-.08 (-.2 to .14)
**Oregon**	4,027 (3,475 to 4,469)	3,914 (3,346 to 5,517)	4,109 (3,285 to 6,455)	.02 (-.2 to .61)	.04 (-.08 to .23)	126 (108 to 139)	88 (75 to 123)	77 (62 to 120)	-.38 (-.52 to -.05)	-.12 (-.22 to .01)
**Pennsylvania**	28,052 (18,734 to 32,491)	25,525 (20,228 to 28,225)	24,692 (21,433 to 27,626)	-.1 (-.26 to .22)	-.03 (-.14 to .14)	197 (132 to 228)	160 (129 to 176)	144 (125 to 161)	-.25 (-.39 to .01)	-.1 (-.2 to .05)
**Rhode Island**	1,289 (1,125 to 1,695)	1,084 (865 to 1,681)	1,113 (825 to 1,894)	-.15 (-.33 to .19)	.02 (-.12 to .17)	111 (97 to 144)	82 (66 to 127)	79 (59 to 133)	-.3 (-.45 to -.01)	-.04 (-.17 to .11)
**South Carolina**	7,599 (5,120 to 8,833)	8,477 (6,882 to 9,460)	9,883 (8,348 to 11,362)	.33 (.07 to .84)	.17 (.02 to .35)	209 (141 to 243)	168 (137 to 188)	160 (134 to 183)	-.22 (-.37 to .08)	-.05 (-.16 to .1)
**South Dakota**	823 (723 to 1,071)	821 (669 to 1,241)	957 (743 to 1,526)	.15 (-.07 to .56)	.16 (.03 to .32)	106 (93 to 137)	87 (71 to 132)	89 (69 to 142)	-.17 (-.33 to .14)	.01 (-.11 to .16)
**Tennessee**	9,452 (7,153 to 10,633)	9,443 (8,395 to 12,574)	9,560 (7,860 to 14,821)	.04 (-.23 to .82)	.01 (-.14 to .27)	175 (134 to 196)	136 (121 to 179)	120 (99 to 180)	-.3 (-.47 to .19)	-.12 (-.24 to .08)
**Texas**	28,999 (21,866 to 32,505)	33,609 (30,668 to 38,720)	40,071 (35,403 to 49,167)	.4 (.14 to .93)	.19 (.07 to .34)	175 (130 to 196)	137 (125 to 158)	130 (115 to 159)	-.24 (-.39 to .04)	-.05 (-.15 to .05)
**Utah**	1,713 (1,493 to 1,984)	2,268 (1,958 to 3,054)	2,805 (2,338 to 3,861)	.64 (.35 to 1.21)	.23 (.11 to .38)	114 (97 to 133)	95 (82 to 128)	91 (76 to 126)	-.2 (-.35 to .09)	-.04 (-.14 to .08)
**Vermont**	730 (640 to 826)	689 (596 to 934)	739 (601 to 1,076)	.01 (-.19 to .39)	.07 (-.07 to .21)	123 (108 to 139)	89 (78 to 120)	84 (69 to 122)	-.31 (-.45 to -.07)	-.06 (-.17 to .06)
**Virginia**	11,600 (8,166 to 13,212)	12,225 (10,317 to 13,370)	12,412 (11,018 to 14,616)	.09 (-.11 to .53)	.02 (-.1 to .2)	183 (129 to 208)	141 (119 to 154)	120 (107 to 141)	-.33 (-.45 to -.07)	-.14 (-.24 to 0)
**Washington**	7,245 (5,642 to 8,049)	7,385 (6,612 to 9,240)	8,607 (7,371 to 11,374)	.2 (-.04 to .71)	.16 (.03 to .29)	141 (110 to 157)	101 (90 to 125)	96 (82 to 125)	-.31 (-.44 to -.04)	-.05 (-.15 to .05)

© 2018 Global Burden of Cardiovascular Diseases Collaboration. *JAMA Cardiology*.

**West Virginia**	3,177 (2,702 to 3,489)	2,851 (2,483 to 3,964)	3,045 (2,523 to 4,532)	-.04 (-.24 to .42)	.06 (-.05 to .21)	151 (129 to 165)	124 (107 to 172)	126 (104 to 186)	-.16 (-.33 to .24)	.01 (-.09 to .14)
**Wisconsin**	8,416 (6,150 to 9,523)	7,495 (6,865 to 8,579)	7,831 (6,940 to 9,855)	-.05 (-.24 to .41)	.04 (-.07 to .21)	157 (113 to 177)	112 (103 to 130)	104 (92 to 131)	-.32 (-.46 to .02)	-.07 (-.17 to .07)
**Wyoming**	489 (435 to 621)	586 (478 to 869)	675 (518 to 1,055)	.37 (.11 to .85)	.15 (-.01 to .31)	109 (97 to 138)	96 (79 to 143)	93 (71 to 146)	-.15 (-.32 to .16)	-.03 (-.16 to .1)
**Other cardiovascular and circulatory diseases**										
**Alabama**	14,793 (13,217 to 16,669)	16,939 (15,186 to 19,084)	19,164 (16,667 to 21,961)	.3 (.17 to .44)	.13 (.03 to .25)	321 (287 to 363)	301 (271 to 339)	300 (260 to 342)	-.07 (-.16 to .04)	0 (-.1 to .1)
**Alaska**	1,022 (903 to 1,168)	1,388 (1,238 to 1,558)	1,738 (1,514 to 1,984)	.7 (.52 to .92)	.25 (.12 to .4)	290 (258 to 327)	226 (201 to 253)	226 (197 to 257)	-.22 (-.3 to -.13)	0 (-.1 to .11)
**Arizona**	9,527 (8,406 to 10,936)	13,618 (12,178 to 15,344)	17,045 (14,911 to 19,500)	.79 (.64 to .96)	.25 (.16 to .35)	233 (206 to 268)	191 (170 to 215)	183 (161 to 210)	-.21 (-.28 to -.14)	-.04 (-.11 to .04)
**Arkansas**	8,502 (7,571 to 9,588)	9,700 (8,740 to 10,927)	10,827 (9,580 to 12,241)	.27 (.17 to .4)	.12 (.03 to .21)	295 (263 to 334)	278 (250 to 313)	277 (246 to 313)	-.06 (-.14 to .04)	0 (-.08 to .08)
**California**	63,905 (56,903 to 72,305)	66,649 (59,385 to 74,737)	77,178 (67,382 to 88,374)	.21 (.09 to .32)	.16 (.06 to .26)	215 (191 to 243)	165 (147 to 185)	154 (135 to 176)	-.28 (-.35 to -.22)	-.06 (-.14 to .02)
**Colorado**	9,206 (8,068 to 10,631)	12,077 (10,591 to 13,735)	15,186 (13,288 to 17,441)	.65 (.51 to .8)	.26 (.17 to .35)	284 (249 to 327)	233 (204 to 266)	222 (195 to 255)	-.22 (-.28 to -.15)	-.05 (-.11 to .02)
**Connecticut**	10,601 (9,284 to 12,137)	9,840 (8,659 to 11,256)	10,414 (8,951 to 12,167)	-.02 (-.12 to .09)	.06 (-.02 to .15)	270 (237 to 310)	210 (184 to 241)	201 (172 to 237)	-.25 (-.33 to -.18)	-.04 (-.12 to .04)
**Delaware**	2,297 (2,044 to 2,604)	2,781 (2,467 to 3,135)	3,197 (2,834 to 3,634)	.39 (.29 to .52)	.15 (.07 to .25)	313 (279 to 355)	258 (229 to 291)	239 (211 to 272)	-.24 (-.3 to -.17)	-.08 (-.14 to 0)
**District of Columbia**	3,227 (2,817 to 3,827)	2,018 (1,752 to 2,366)	1,925 (1,641 to 2,241)	-.4 (-.47 to -.32)	-.04 (-.15 to .07)	471 (410 to 561)	305 (264 to 359)	253 (215 to 294)	-.46 (-.53 to -.39)	-.17 (-.26 to -.07)

© 2018 Global Burden of Cardiovascular Diseases Collaboration. *JAMA Cardiology*.

**Florida**	41,941 (37,318 to 48,038)	50,038 (44,502 to 57,054)	58,930 (51,005 to 67,700)	.41 (.29 to .54)	.18 (.09 to .27)	235 (209 to 269)	200 (177 to 228)	192 (167 to 219)	-.18 (-.25 to -.11)	-.04 (-.11 to .03)
**Georgia**	19,349 (17,299 to 21,746)	24,776 (22,137 to 27,703)	30,342 (26,447 to 34,596)	.57 (.4 to .75)	.23 (.11 to .35)	302 (270 to 339)	254 (228 to 284)	246 (214 to 280)	-.19 (-.27 to -.09)	-.03 (-.12 to .07)
**Hawaii**	2,833 (2,464 to 3,291)	3,334 (2,928 to 3,821)	3,973 (3,477 to 4,576)	.4 (.29 to .52)	.19 (.11 to .28)	244 (213 to 283)	196 (172 to 224)	193 (168 to 222)	-.21 (-.27 to -.15)	-.02 (-.08 to .05)
**Idaho**	3,017 (2,636 to 3,471)	4,031 (3,551 to 4,554)	5,087 (4,418 to 5,891)	.69 (.53 to .86)	.26 (.15 to .39)	280 (244 to 323)	238 (209 to 270)	234 (203 to 271)	-.16 (-.24 to -.07)	-.02 (-.1 to .08)
**Illinois**	35,569 (31,778 to 40,305)	34,742 (31,027 to 39,082)	35,981 (32,032 to 40,679)	.01 (-.07 to .1)	.04 (-.04 to .11)	278 (248 to 315)	232 (206 to 261)	213 (190 to 242)	-.23 (-.29 to -.16)	-.08 (-.14 to -.01)
**Indiana**	17,661 (15,513 to 20,065)	20,567 (18,168 to 23,417)	23,578 (20,419 to 27,274)	.34 (.21 to .47)	.15 (.05 to .25)	283 (249 to 322)	272 (240 to 309)	273 (236 to 316)	-.04 (-.13 to .06)	0 (-.08 to .1)
**Iowa**	9,235 (8,117 to 10,628)	8,494 (7,547 to 9,542)	9,256 (8,185 to 10,610)	0 (-.08 to .1)	.09 (.01 to .18)	262 (230 to 302)	210 (187 to 237)	207 (182 to 237)	-.21 (-.27 to -.13)	-.01 (-.09 to .06)
**Kansas**	7,852 (6,909 to 8,956)	8,025 (7,152 to 9,109)	8,877 (7,789 to 10,144)	.13 (.02 to .25)	.11 (0 to .22)	268 (234 to 308)	234 (208 to 267)	232 (203 to 265)	-.13 (-.22 to -.04)	-.01 (-.1 to .09)
**Kentucky**	11,540 (10,313 to 13,116)	13,565 (12,050 to 15,466)	15,342 (13,514 to 17,385)	.33 (.22 to .45)	.13 (.05 to .22)	278 (248 to 316)	266 (236 to 302)	263 (233 to 298)	-.05 (-.13 to .03)	-.01 (-.08 to .07)
**Louisiana**	13,585 (12,233 to 15,131)	14,086 (12,587 to 15,798)	15,854 (14,071 to 17,988)	.17 (.07 to .28)	.13 (.04 to .21)	312 (281 to 348)	282 (252 to 318)	272 (241 to 309)	-.13 (-.2 to -.04)	-.04 (-.11 to .03)
**Maine**	3,982 (3,503 to 4,562)	4,140 (3,679 to 4,689)	4,640 (4,099 to 5,300)	.17 (.07 to .27)	.12 (.04 to .22)	275 (242 to 316)	226 (201 to 257)	219 (193 to 250)	-.2 (-.27 to -.13)	-.03 (-.11 to .05)
**Maryland**	15,763 (13,960 to 17,642)	17,123 (15,072 to 19,424)	19,003 (16,740 to 21,608)	.21 (.11 to .31)	.11 (.04 to .19)	316 (280 to 353)	259 (228 to 293)	242 (213 to 276)	-.23 (-.3 to -.17)	-.06 (-.12 to 0)
**Massachusetts**	18,527 (16,307 to 21,214)	17,957 (15,696 to 20,754)	18,688 (16,219 to 21,881)	.01 (-.07 to .09)	.04 (-.03 to .12)	260 (228 to 299)	213 (186 to 248)	194 (168 to 227)	-.25 (-.31 to -.19)	-.09 (-.15 to -.02)

© 2018 Global Burden of Cardiovascular Diseases Collaboration. *JAMA Cardiology*.

**Michigan**	25,535 (22,802 to 29,048)	27,589 (24,484 to 31,180)	31,472 (27,542 to 35,943)	.23 (.13 to .34)	.14 (.07 to .22)	253 (225 to 289)	225 (199 to 254)	229 (201 to 260)	-.09 (-.17 to -.01)	.02 (-.05 to .09)
**Minnesota**	11,808 (10,443 to 13,511)	12,368 (10,908 to 14,121)	14,439 (12,556 to 16,544)	.22 (.11 to .34)	.17 (.08 to .26)	239 (211 to 274)	193 (170 to 222)	190 (164 to 218)	-.2 (-.28 to -.13)	-.02 (-.09 to .06)
**Mississippi**	8,798 (7,989 to 9,827)	9,957 (8,942 to 11,162)	11,002 (9,606 to 12,495)	.25 (.1 to .4)	.11 (-.01 to .22)	313 (284 to 349)	298 (267 to 332)	293 (254 to 333)	-.06 (-.18 to .05)	-.02 (-.12 to .09)
**Missouri**	16,736 (14,826 to 19,016)	18,391 (16,409 to 20,754)	20,889 (18,602 to 23,671)	.25 (.15 to .36)	.14 (.06 to .22)	272 (241 to 309)	252 (226 to 285)	252 (226 to 286)	-.07 (-.15 to .01)	0 (-.07 to .08)
**Montana**	2,558 (2,240 to 2,912)	2,920 (2,577 to 3,305)	3,408 (2,939 to 3,931)	.33 (.2 to .47)	.17 (.07 to .28)	277 (242 to 316)	233 (206 to 264)	227 (196 to 261)	-.18 (-.26 to -.1)	-.03 (-.11 to .07)
**Nebraska**	5,411 (4,773 to 6,191)	5,273 (4,645 to 5,989)	5,966 (5,199 to 6,876)	.1 (.01 to .2)	.13 (.05 to .21)	286 (251 to 328)	236 (207 to 268)	235 (205 to 271)	-.18 (-.24 to -.11)	0 (-.08 to .07)
**Nevada**	3,441 (3,065 to 3,870)	6,593 (5,914 to 7,366)	8,138 (7,168 to 9,287)	1.37 (1.16 to 1.6)	.23 (.14 to .33)	279 (249 to 313)	234 (210 to 261)	215 (190 to 244)	-.23 (-.3 to -.15)	-.08 (-.15 to -.01)
**New Hampshire**	3,278 (2,890 to 3,798)	3,493 (3,080 to 3,973)	4,021 (3,540 to 4,632)	.23 (.13 to .33)	.15 (.07 to .24)	277 (244 to 322)	212 (187 to 242)	203 (179 to 234)	-.27 (-.33 to -.2)	-.04 (-.11 to .04)
**New Jersey**	28,051 (24,849 to 32,000)	27,711 (24,332 to 31,698)	28,927 (25,030 to 33,274)	.03 (-.05 to .12)	.04 (-.02 to .12)	308 (273 to 351)	254 (223 to 292)	235 (203 to 271)	-.24 (-.3 to -.17)	-.08 (-.14 to 0)
**New Mexico**	3,597 (3,164 to 4,154)	4,775 (4,191 to 5,458)	5,586 (4,816 to 6,463)	.55 (.4 to .72)	.17 (.07 to .28)	235 (206 to 271)	205 (180 to 235)	203 (174 to 234)	-.14 (-.22 to -.04)	-.01 (-.1 to .08)
**New York**	53,446 (47,453 to 60,911)	47,925 (41,405 to 55,655)	52,280 (44,753 to 61,336)	-.02 (-.12 to .08)	.09 (0 to .19)	258 (228 to 295)	202 (175 to 236)	197 (168 to 231)	-.23 (-.31 to -.15)	-.02 (-.1 to .06)
**North Carolina**	21,530 (19,361 to 24,217)	25,928 (23,282 to 28,954)	31,121 (27,769 to 34,838)	.45 (.33 to .57)	.2 (.12 to .29)	294 (264 to 330)	244 (219 to 272)	233 (208 to 260)	-.21 (-.27 to -.14)	-.04 (-.11 to .03)
**North Dakota**	1,824 (1,586 to 2,127)	1,662 (1,461 to 1,898)	1,867 (1,601 to 2,163)	.02 (-.06 to .13)	.12 (.03 to .22)	238 (207 to 278)	192 (168 to 221)	190 (164 to 221)	-.2 (-.27 to -.12)	-.01 (-.09 to .08)

© 2018 Global Burden of Cardiovascular Diseases Collaboration. *JAMA Cardiology*.

**Ohio**	33,952 (30,256 to 38,328)	35,673 (31,658 to 40,707)	38,544 (34,010 to 43,814)	.14 (.04 to .23)	.08 (.01 to .16)	273 (244 to 309)	247 (219 to 281)	241 (212 to 274)	-.12 (-.19 to -.04)	-.02 (-.08 to .05)
**Oklahoma**	9,461 (8,408 to 10,712)	10,593 (9,374 to 11,917)	11,756 (10,350 to 13,287)	.24 (.14 to .36)	.11 (.03 to .19)	258 (229 to 293)	244 (217 to 274)	238 (210 to 268)	-.08 (-.15 to .01)	-.02 (-.09 to .05)
**Oregon**	9,267 (8,113 to 10,710)	11,346 (10,047 to 12,769)	13,580 (11,906 to 15,384)	.47 (.34 to .6)	.2 (.11 to .29)	275 (241 to 320)	236 (208 to 266)	230 (202 to 261)	-.16 (-.23 to -.09)	-.02 (-.09 to .04)
**Pennsylvania**	48,903 (43,769 to 55,592)	47,217 (42,123 to 53,621)	48,827 (43,151 to 55,382)	0 (-.08 to .08)	.03 (-.03 to .1)	322 (288 to 367)	276 (245 to 314)	260 (230 to 294)	-.19 (-.26 to -.13)	-.06 (-.12 to .01)
**Rhode Island**	3,079 (2,690 to 3,567)	2,719 (2,367 to 3,117)	2,837 (2,471 to 3,328)	-.08 (-.16 to .02)	.04 (-.05 to .14)	249 (217 to 290)	193 (167 to 222)	187 (162 to 221)	-.25 (-.32 to -.17)	-.03 (-.11 to .06)
**South Carolina**	11,856 (10,596 to 13,345)	14,848 (13,341 to 16,669)	18,417 (16,257 to 20,956)	.55 (.41 to .72)	.24 (.13 to .36)	323 (289 to 364)	283 (254 to 317)	277 (245 to 316)	-.14 (-.23 to -.05)	-.02 (-.11 to .07)
**South Dakota**	1,975 (1,728 to 2,298)	2,009 (1,767 to 2,312)	2,366 (2,046 to 2,721)	.2 (.09 to .32)	.18 (.09 to .29)	236 (206 to 275)	198 (173 to 228)	201 (174 to 231)	-.14 (-.23 to -.06)	.02 (-.07 to .12)
**Tennessee**	14,672 (12,987 to 16,424)	18,550 (16,689 to 20,835)	22,562 (19,993 to 25,411)	.54 (.41 to .67)	.22 (.13 to .31)	264 (235 to 297)	253 (228 to 284)	258 (228 to 290)	-.02 (-.11 to .06)	.02 (-.05 to .1)
**Texas**	46,393 (41,345 to 52,864)	60,610 (53,403 to 68,961)	75,523 (66,082 to 85,428)	.63 (.5 to .77)	.25 (.16 to .34)	279 (249 to 318)	248 (218 to 282)	237 (207 to 268)	-.15 (-.22 to -.08)	-.04 (-.11 to .03)
**Utah**	4,037 (3,487 to 4,694)	5,927 (5,103 to 6,857)	7,444 (6,498 to 8,624)	.85 (.71 to 1.02)	.26 (.18 to .34)	273 (236 to 317)	251 (215 to 290)	240 (209 to 278)	-.12 (-.19 to -.03)	-.04 (-.1 to .02)
**Vermont**	1,851 (1,635 to 2,116)	1,779 (1,578 to 2,011)	2,030 (1,798 to 2,331)	.1 (0 to .2)	.14 (.05 to .23)	301 (266 to 345)	217 (192 to 245)	213 (188 to 244)	-.29 (-.35 to -.23)	-.02 (-.09 to .06)
**Virginia**	19,206 (17,159 to 21,837)	22,427 (19,810 to 25,437)	25,476 (22,622 to 28,846)	.33 (.23 to .44)	.14 (.06 to .22)	301 (269 to 342)	250 (221 to 284)	233 (206 to 263)	-.23 (-.29 to -.16)	-.07 (-.13 to 0)
**Washington**	14,062 (12,290 to 16,266)	16,943 (15,141 to 19,033)	20,834 (18,447 to 23,455)	.48 (.36 to .62)	.23 (.14 to .32)	267 (234 to 309)	221 (197 to 249)	216 (191 to 243)	-.19 (-.26 to -.12)	-.02 (-.09 to .05)

© 2018 Global Burden of Cardiovascular Diseases Collaboration. *JAMA Cardiology*.

**West Virginia**	6,595 (5,884 to 7,452)	6,640 (5,907 to 7,541)	7,289 (6,469 to 8,275)	.11 (.02 to .21)	.1 (.02 to .18)	294 (262 to 333)	270 (241 to 306)	278 (246 to 315)	-.05 (-.13 to .03)	.03 (-.04 to .11)
**Wisconsin**	14,899 (13,122 to 16,873)	15,468 (13,826 to 17,312)	17,604 (15,609 to 19,694)	.18 (.09 to .29)	.14 (.06 to .22)	261 (230 to 297)	216 (194 to 241)	214 (191 to 240)	-.18 (-.25 to -.1)	-.01 (-.08 to .06)
**Wyoming**	1,380 (1,215 to 1,582)	1,700 (1,502 to 1,919)	1,986 (1,720 to 2,293)	.44 (.31 to .59)	.17 (.07 to .28)	305 (268 to 351)	271 (240 to 305)	264 (228 to 302)	-.14 (-.22 to -.05)	-.03 (-.11 to .07)
**Peripheral artery disease**										
**Alabama**	1,568 (1,074 to 2,054)	2,304 (1,627 to 3,612)	2,840 (2,087 to 4,601)	.84 (.42 to 2.18)	.23 (.06 to .41)	32 (22 to 42)	38 (26 to 59)	38 (28 to 62)	.2 (-.07 to 1.07)	.01 (-.13 to .16)
**Alaska**	72 (56 to 99)	162 (124 to 247)	238 (174 to 375)	2.29 (1.65 to 3.51)	.47 (.26 to .7)	30 (23 to 42)	31 (23 to 46)	31 (23 to 49)	.02 (-.18 to .4)	.02 (-.13 to .17)
**Arizona**	1,168 (851 to 1,583)	2,295 (1,729 to 3,207)	3,226 (2,411 to 4,625)	1.78 (1.3 to 2.92)	.4 (.26 to .56)	27 (20 to 37)	30 (23 to 42)	30 (22 to 42)	.1 (-.09 to .56)	-.01 (-.11 to .11)
**Arkansas**	971 (683 to 1,305)	1,392 (988 to 2,053)	1,698 (1,223 to 2,806)	.76 (.38 to 1.9)	.21 (.04 to .44)	30 (21 to 40)	36 (25 to 53)	37 (27 to 61)	.23 (-.03 to 1.04)	.03 (-.11 to .22)
**California**	8,060 (5,940 to 11,181)	11,571 (8,784 to 17,178)	14,824 (10,693 to 23,537)	.84 (.49 to 1.64)	.28 (.12 to .45)	27 (20 to 38)	28 (22 to 42)	28 (20 to 44)	.01 (-.18 to .44)	-.04 (-.15 to .1)
**Colorado**	815 (601 to 1,151)	1,341 (1,030 to 1,957)	1,916 (1,415 to 2,977)	1.36 (.95 to 2.16)	.42 (.26 to .61)	26 (19 to 36)	26 (20 to 38)	26 (19 to 40)	.02 (-.16 to .37)	-.01 (-.12 to .12)
**Connecticut**	1,266 (910 to 1,731)	1,544 (1,187 to 2,202)	1,766 (1,308 to 2,687)	.4 (.14 to .95)	.14 (-.01 to .31)	30 (22 to 41)	30 (23 to 43)	30 (22 to 44)	0 (-.2 to .38)	-.02 (-.15 to .12)
**Delaware**	272 (188 to 368)	429 (316 to 596)	575 (431 to 831)	1.13 (.74 to 2.02)	.34 (.17 to .5)	36 (25 to 49)	37 (27 to 51)	37 (27 to 53)	.02 (-.17 to .46)	-.01 (-.13 to .11)
**District of Columbia**	312 (198 to 428)	301 (205 to 442)	323 (233 to 500)	.05 (-.16 to .39)	.08 (-.08 to .27)	44 (28 to 61)	44 (30 to 65)	41 (30 to 63)	-.05 (-.24 to .26)	-.06 (-.2 to .11)

© 2018 Global Burden of Cardiovascular Diseases Collaboration. *JAMA Cardiology*.

**Florida**	6,048 (4,405 to 8,244)	9,551 (7,252 to 13,135)	12,382 (9,168 to 17,831)	1.06 (.7 to 1.85)	.29 (.15 to .45)	29 (21 to 40)	33 (25 to 45)	33 (24 to 46)	.13 (-.07 to .55)	-.01 (-.13 to .11)
**Georgia**	2,191 (1,419 to 2,881)	3,614 (2,587 to 5,082)	4,930 (3,702 to 7,316)	1.3 (.79 to 2.74)	.36 (.18 to .56)	35 (23 to 46)	38 (27 to 53)	37 (28 to 55)	.08 (-.16 to .77)	-.02 (-.16 to .12)
**Hawaii**	278 (206 to 397)	438 (324 to 681)	594 (427 to 950)	1.14 (.76 to 2.05)	.35 (.21 to .5)	24 (18 to 34)	24 (18 to 37)	25 (18 to 40)	.04 (-.14 to .48)	.04 (-.07 to .15)
**Idaho**	299 (211 to 401)	484 (365 to 703)	688 (512 to 1,033)	1.33 (.81 to 2.63)	.42 (.22 to .61)	26 (19 to 35)	27 (21 to 39)	28 (21 to 42)	.08 (-.16 to .68)	.03 (-.11 to .18)
**Illinois**	4,276 (3,025 to 5,777)	5,413 (4,021 to 7,820)	6,375 (4,752 to 9,563)	.5 (.22 to 1.17)	.18 (.03 to .31)	32 (22 to 43)	34 (25 to 48)	34 (25 to 51)	.08 (-.12 to .55)	-.01 (-.13 to .1)
**Indiana**	2,146 (1,511 to 2,897)	2,994 (2,201 to 4,319)	3,701 (2,693 to 5,668)	.74 (.41 to 1.56)	.24 (.08 to .38)	33 (23 to 44)	37 (27 to 53)	38 (27 to 57)	.17 (-.05 to .72)	.02 (-.1 to .14)
**Iowa**	1,118 (832 to 1,532)	1,339 (992 to 2,006)	1,563 (1,156 to 2,436)	.4 (.14 to 1.04)	.16 (.02 to .31)	27 (21 to 38)	29 (22 to 44)	30 (22 to 47)	.1 (-.1 to .59)	.02 (-.1 to .15)
**Kansas**	900 (669 to 1,228)	1,138 (867 to 1,701)	1,356 (986 to 2,172)	.51 (.2 to 1.37)	.19 (.03 to .36)	28 (21 to 38)	30 (23 to 45)	31 (23 to 49)	.12 (-.11 to .74)	.01 (-.12 to .17)
**Kentucky**	1,412 (982 to 1,888)	2,163 (1,519 to 3,107)	2,675 (1,937 to 4,057)	.91 (.55 to 1.99)	.23 (.09 to .39)	32 (22 to 43)	40 (28 to 57)	40 (29 to 61)	.26 (.02 to .96)	.01 (-.11 to .14)
**Louisiana**	1,541 (1,005 to 2,025)	2,102 (1,464 to 3,060)	2,612 (1,905 to 4,284)	.72 (.32 to 1.88)	.24 (.05 to .45)	35 (23 to 46)	40 (28 to 58)	40 (29 to 65)	.17 (-.1 to .95)	0 (-.15 to .17)
**Maine**	493 (332 to 649)	673 (495 to 903)	823 (630 to 1,183)	.69 (.36 to 1.45)	.22 (.07 to .36)	32 (21 to 42)	33 (24 to 45)	33 (25 to 46)	.05 (-.16 to .51)	-.02 (-.15 to .09)
**Maryland**	1,811 (1,259 to 2,487)	2,509 (1,893 to 3,565)	3,134 (2,340 to 4,750)	.75 (.4 to 1.62)	.24 (.09 to .41)	37 (26 to 50)	37 (28 to 52)	36 (27 to 54)	-.01 (-.21 to .48)	-.03 (-.15 to .1)
**Massachusetts**	2,277 (1,501 to 3,021)	2,749 (2,043 to 3,761)	3,227 (2,471 to 4,703)	.43 (.16 to 1.01)	.17 (.02 to .32)	30 (19 to 40)	30 (23 to 41)	30 (23 to 43)	.02 (-.17 to .42)	-.02 (-.14 to .1)

© 2018 Global Burden of Cardiovascular Diseases Collaboration. *JAMA Cardiology*.

**Michigan**	3,244 (2,417 to 4,457)	4,683 (3,492 to 7,227)	5,602 (4,158 to 8,896)	.73 (.41 to 1.55)	.19 (.06 to .31)	31 (23 to 43)	36 (27 to 55)	35 (26 to 56)	.14 (-.07 to .67)	-.01 (-.12 to .09)
**Minnesota**	1,504 (1,041 to 1,974)	1,864 (1,376 to 2,615)	2,341 (1,749 to 3,507)	.57 (.26 to 1.28)	.25 (.08 to .42)	28 (20 to 37)	27 (20 to 38)	27 (20 to 41)	-.02 (-.21 to .44)	0 (-.14 to .13)
**Mississippi**	901 (673 to 1,234)	1,244 (874 to 2,469)	1,568 (1,068 to 3,162)	.73 (.3 to 2.02)	.26 (.1 to .44)	30 (22 to 41)	35 (24 to 68)	36 (25 to 73)	.2 (-.09 to 1.1)	.04 (-.08 to .2)
**Missouri**	2,087 (1,474 to 2,800)	2,847 (2,089 to 4,157)	3,466 (2,560 to 5,311)	.67 (.36 to 1.58)	.22 (.08 to .35)	31 (22 to 41)	36 (26 to 52)	36 (27 to 55)	.18 (-.04 to .82)	.01 (-.1 to .14)
**Montana**	290 (197 to 379)	421 (319 to 567)	544 (404 to 767)	.9 (.51 to 1.81)	.29 (.12 to .48)	29 (20 to 38)	30 (23 to 41)	31 (23 to 43)	.07 (-.15 to .59)	.01 (-.13 to .16)
**Nebraska**	584 (430 to 798)	718 (540 to 1,081)	868 (650 to 1,360)	.49 (.21 to 1.24)	.2 (.06 to .34)	27 (20 to 37)	29 (22 to 44)	30 (22 to 47)	.1 (-.11 to .65)	.03 (-.09 to .14)
**Nevada**	390 (289 to 530)	1,008 (755 to 1,387)	1,426 (1,069 to 2,106)	2.67 (2.03 to 3.97)	.41 (.23 to .6)	33 (24 to 44)	36 (27 to 49)	34 (26 to 51)	.06 (-.13 to .45)	-.04 (-.16 to .09)
**New Hampshire**	376 (266 to 507)	557 (427 to 789)	722 (548 to 1,078)	.94 (.56 to 1.83)	.29 (.13 to .44)	31 (22 to 42)	32 (25 to 45)	32 (24 to 47)	.02 (-.18 to .49)	-.02 (-.14 to .09)
**New Jersey**	3,211 (2,282 to 4,419)	3,922 (2,980 to 5,517)	4,553 (3,388 to 6,743)	.43 (.17 to 1.04)	.16 (.02 to .3)	33 (24 to 46)	33 (25 to 46)	33 (24 to 48)	-.01 (-.19 to .39)	-.02 (-.14 to .1)
**New Mexico**	420 (295 to 564)	730 (558 to 1,034)	975 (735 to 1,442)	1.35 (.86 to 2.61)	.33 (.16 to .52)	27 (19 to 36)	29 (23 to 42)	30 (23 to 44)	.12 (-.12 to .72)	.02 (-.11 to .17)
**New York**	6,382 (4,768 to 9,191)	7,210 (5,143 to 11,425)	8,382 (5,743 to 14,081)	.31 (.06 to .91)	.16 (.03 to .31)	29 (21 to 41)	28 (20 to 44)	28 (19 to 46)	-.04 (-.22 to .41)	-.02 (-.13 to .11)
**North Carolina**	2,424 (1,617 to 3,215)	3,923 (2,750 to 5,506)	5,198 (3,834 to 7,606)	1.17 (.74 to 2.35)	.33 (.16 to .49)	32 (21 to 43)	35 (25 to 49)	34 (25 to 50)	.09 (-.13 to .68)	-.02 (-.15 to .1)
**North Dakota**	232 (168 to 311)	274 (202 to 410)	325 (236 to 513)	.41 (.11 to 1.17)	.18 (.02 to .34)	27 (19 to 36)	28 (20 to 42)	29 (21 to 45)	.08 (-.14 to .64)	.03 (-.11 to .17)

© 2018 Global Burden of Cardiovascular Diseases Collaboration. *JAMA Cardiology*.

**Ohio**	4,374 (3,029 to 5,823)	6,150 (4,290 to 8,621)	7,045 (5,136 to 10,302)	.63 (.33 to 1.45)	.14 (0 to .28)	33 (23 to 44)	39 (27 to 54)	38 (28 to 55)	.16 (-.05 to .75)	-.03 (-.15 to .09)
**Oklahoma**	1,123 (835 to 1,582)	1,667 (1,216 to 2,758)	2,027 (1,414 to 3,626)	.8 (.43 to 2.14)	.2 (.05 to .4)	28 (21 to 40)	35 (26 to 58)	36 (25 to 64)	.27 (.01 to 1.22)	.01 (-.12 to .18)
**Oregon**	1,078 (747 to 1,412)	1,551 (1,169 to 2,191)	2,003 (1,500 to 2,904)	.87 (.53 to 1.7)	.29 (.15 to .43)	30 (21 to 39)	31 (23 to 42)	30 (23 to 44)	.03 (-.16 to .49)	-.01 (-.12 to .1)
**Pennsylvania**	5,670 (3,958 to 7,486)	6,884 (5,066 to 9,841)	7,988 (5,946 to 11,557)	.42 (.18 to 1.02)	.16 (.04 to .27)	34 (24 to 45)	35 (26 to 51)	36 (27 to 52)	.08 (-.11 to .52)	.02 (-.09 to .12)
**Rhode Island**	426 (303 to 587)	507 (387 to 719)	578 (422 to 892)	.37 (.09 to 1.01)	.14 (-.02 to .3)	31 (22 to 42)	33 (25 to 45)	33 (24 to 50)	.08 (-.14 to .58)	.01 (-.13 to .16)
**South Carolina**	1,347 (837 to 1,773)	2,239 (1,490 to 3,010)	3,091 (2,143 to 4,423)	1.34 (.86 to 2.77)	.38 (.19 to .58)	37 (23 to 48)	40 (27 to 54)	40 (28 to 58)	.12 (-.11 to .81)	.01 (-.13 to .16)
**South Dakota**	267 (184 to 350)	340 (246 to 481)	427 (314 to 642)	.62 (.28 to 1.49)	.25 (.1 to .42)	28 (19 to 36)	30 (21 to 42)	31 (23 to 46)	.12 (-.12 to .73)	.03 (-.1 to .17)
**Tennessee**	1,893 (1,236 to 2,493)	3,014 (2,100 to 4,302)	3,967 (2,822 to 5,913)	1.13 (.7 to 2.41)	.32 (.15 to .47)	32 (21 to 42)	38 (27 to 55)	40 (28 to 59)	.24 (-.01 to .99)	.03 (-.1 to .15)
**Texas**	4,981 (3,472 to 6,650)	8,660 (6,009 to 11,914)	11,910 (8,672 to 17,452)	1.41 (.97 to 2.71)	.37 (.2 to .56)	30 (21 to 40)	36 (25 to 49)	36 (26 to 52)	.19 (-.03 to .82)	-.01 (-.13 to .12)
**Utah**	351 (253 to 480)	587 (446 to 859)	819 (604 to 1,257)	1.35 (.89 to 2.42)	.39 (.22 to .58)	25 (18 to 34)	26 (19 to 37)	26 (19 to 40)	.06 (-.15 to .54)	.01 (-.11 to .14)
**Vermont**	205 (134 to 271)	275 (209 to 374)	342 (265 to 488)	.69 (.35 to 1.42)	.25 (.08 to .4)	32 (21 to 42)	31 (24 to 42)	31 (24 to 43)	-.03 (-.22 to .38)	-.02 (-.14 to .1)
**Virginia**	1,959 (1,395 to 2,693)	3,011 (2,265 to 4,386)	3,845 (2,926 to 5,928)	.98 (.59 to 1.91)	.27 (.12 to .44)	31 (22 to 42)	33 (25 to 48)	32 (24 to 49)	.04 (-.16 to .54)	-.03 (-.15 to .1)
**Washington**	1,512 (1,105 to 2,068)	2,332 (1,794 to 3,436)	3,064 (2,310 to 4,725)	1.04 (.64 to 2.04)	.31 (.16 to .47)	28 (20 to 38)	30 (23 to 43)	29 (22 to 45)	.04 (-.16 to .55)	-.02 (-.14 to .09)

© 2018 Global Burden of Cardiovascular Diseases Collaboration. *JAMA Cardiology*.

**West Virginia**	823 (558 to 1,088)	1,108 (782 to 1,677)	1,303 (938 to 1,977)	.6 (.28 to 1.59)	.18 (.04 to .32)	33 (22 to 44)	39 (28 to 59)	41 (29 to 62)	.24 (-.01 to 1)	.03 (-.08 to .16)
**Wisconsin**	1,957 (1,333 to 2,573)	2,606 (1,844 to 3,392)	3,158 (2,286 to 4,459)	.63 (.34 to 1.29)	.21 (.07 to .37)	31 (22 to 41)	34 (24 to 44)	34 (25 to 47)	.08 (-.11 to .51)	0 (-.12 to .12)
**Wyoming**	128 (93 to 172)	199 (153 to 285)	261 (196 to 397)	1.05 (.63 to 1.91)	.31 (.13 to .5)	28 (21 to 38)	30 (23 to 43)	31 (23 to 47)	.09 (-.13 to .57)	.02 (-.12 to .17)
**Rheumatic heart disease**										
**Alabama**	4,060 (3,782 to 4,347)	4,006 (3,761 to 4,269)	4,471 (3,946 to 5,015)	.1 (-.04 to .26)	.12 (-.02 to .26)	89 (83 to 95)	70 (66 to 75)	68 (60 to 76)	-.24 (-.34 to -.12)	-.03 (-.15 to .09)
**Alaska**	348 (320 to 377)	386 (356 to 418)	466 (401 to 531)	.34 (.12 to .58)	.21 (.03 to .4)	102 (94 to 111)	63 (58 to 68)	60 (52 to 68)	-.41 (-.51 to -.31)	-.05 (-.19 to .09)
**Arizona**	2,463 (2,309 to 2,640)	2,560 (2,403 to 2,725)	3,003 (2,728 to 3,322)	.22 (.08 to .38)	.17 (.05 to .31)	60 (57 to 65)	36 (34 to 38)	32 (29 to 35)	-.47 (-.53 to -.4)	-.1 (-.2 to .01)
**Arkansas**	2,100 (1,975 to 2,234)	1,924 (1,804 to 2,045)	2,182 (1,967 to 2,400)	.04 (-.07 to .17)	.13 (.02 to .26)	73 (69 to 78)	55 (51 to 58)	54 (49 to 60)	-.26 (-.34 to -.17)	0 (-.1 to .11)
**California**	20,549 (18,975 to 22,322)	14,244 (13,368 to 15,182)	15,058 (13,508 to 16,733)	-.27 (-.36 to -.15)	.06 (-.07 to .2)	69 (64 to 75)	35 (33 to 38)	30 (27 to 33)	-.57 (-.63 to -.5)	-.16 (-.26 to -.04)
**Colorado**	2,691 (2,525 to 2,879)	2,520 (2,353 to 2,690)	3,014 (2,700 to 3,327)	.12 (-.01 to .27)	.2 (.07 to .33)	83 (78 to 89)	49 (46 to 52)	44 (39 to 48)	-.48 (-.53 to -.4)	-.11 (-.2 to 0)
**Connecticut**	2,636 (2,454 to 2,841)	1,770 (1,651 to 1,886)	1,761 (1,562 to 1,983)	-.33 (-.42 to -.23)	0 (-.11 to .12)	67 (62 to 72)	37 (35 to 40)	33 (29 to 38)	-.5 (-.57 to -.42)	-.11 (-.21 to .01)
**Delaware**	538 (494 to 580)	487 (454 to 519)	563 (514 to 614)	.05 (-.07 to .19)	.16 (.05 to .28)	73 (67 to 79)	45 (41 to 48)	41 (37 to 44)	-.44 (-.51 to -.37)	-.08 (-.17 to .02)
**District of Columbia**	642 (550 to 786)	319 (283 to 373)	270 (226 to 311)	-.58 (-.65 to -.48)	-.15 (-.28 to 0)	95 (81 to 116)	48 (43 to 57)	35 (30 to 41)	-.62 (-.69 to -.54)	-.27 (-.38 to -.13)

© 2018 Global Burden of Cardiovascular Diseases Collaboration. *JAMA Cardiology*.

**Florida**	10,075 (9,322 to 10,926)	7,968 (7,423 to 8,554)	9,112 (8,233 to 10,044)	-.09 (-.2 to .02)	.14 (.01 to .28)	57 (53 to 62)	31 (29 to 34)	29 (26 to 32)	-.5 (-.56 to -.43)	-.09 (-.2 to .02)
**Georgia**	4,665 (4,303 to 5,095)	5,152 (4,776 to 5,623)	6,325 (5,627 to 7,103)	.36 (.17 to .55)	.23 (.08 to .4)	73 (67 to 79)	53 (49 to 58)	50 (45 to 56)	-.31 (-.4 to -.21)	-.05 (-.16 to .08)
**Hawaii**	751 (698 to 812)	702 (660 to 751)	818 (746 to 890)	.09 (-.03 to .22)	.17 (.06 to .28)	65 (60 to 70)	41 (39 to 44)	40 (36 to 43)	-.39 (-.45 to -.31)	-.04 (-.13 to .06)
**Idaho**	895 (831 to 964)	911 (846 to 975)	1,148 (1,019 to 1,293)	.28 (.11 to .47)	.26 (.1 to .43)	83 (77 to 89)	53 (50 to 57)	52 (46 to 59)	-.37 (-.45 to -.27)	-.02 (-.15 to .11)
**Illinois**	9,254 (8,640 to 9,928)	6,404 (6,015 to 6,797)	6,714 (6,122 to 7,299)	-.27 (-.36 to -.19)	.05 (-.05 to .17)	73 (68 to 78)	42 (40 to 45)	39 (35 to 42)	-.47 (-.53 to -.41)	-.08 (-.17 to .02)
**Indiana**	4,522 (4,214 to 4,805)	3,851 (3,603 to 4,112)	4,436 (3,954 to 4,960)	-.02 (-.15 to .12)	.15 (.01 to .29)	73 (68 to 77)	50 (47 to 54)	50 (45 to 57)	-.31 (-.4 to -.2)	0 (-.12 to .13)
**Iowa**	1,952 (1,822 to 2,078)	1,432 (1,341 to 1,525)	1,536 (1,375 to 1,709)	-.21 (-.3 to -.11)	.07 (-.03 to .19)	55 (51 to 59)	35 (33 to 37)	34 (30 to 38)	-.39 (-.46 to -.31)	-.03 (-.14 to .08)
**Kansas**	1,887 (1,764 to 2,016)	1,653 (1,548 to 1,754)	1,783 (1,567 to 2,017)	-.05 (-.18 to .08)	.08 (-.05 to .22)	65 (60 to 69)	48 (44 to 51)	45 (40 to 52)	-.3 (-.39 to -.2)	-.04 (-.16 to .09)
**Kentucky**	3,235 (3,038 to 3,432)	2,948 (2,767 to 3,123)	3,339 (3,034 to 3,643)	.03 (-.08 to .16)	.13 (.03 to .25)	78 (73 to 83)	57 (54 to 61)	56 (51 to 61)	-.28 (-.36 to -.2)	-.02 (-.11 to .08)
**Louisiana**	3,125 (2,892 to 3,409)	2,738 (2,529 to 3,006)	3,076 (2,813 to 3,370)	-.01 (-.12 to .1)	.12 (.02 to .25)	72 (67 to 79)	54 (50 to 60)	51 (47 to 57)	-.29 (-.36 to -.21)	-.06 (-.14 to .05)
**Maine**	942 (882 to 1,006)	770 (715 to 823)	846 (760 to 932)	-.1 (-.2 to 0)	.1 (-.02 to .22)	65 (61 to 69)	42 (39 to 44)	39 (35 to 43)	-.4 (-.47 to -.33)	-.06 (-.16 to .04)
**Maryland**	3,518 (3,241 to 3,872)	2,562 (2,370 to 2,801)	2,664 (2,419 to 2,939)	-.24 (-.32 to -.14)	.04 (-.06 to .16)	70 (65 to 77)	39 (36 to 42)	34 (30 to 37)	-.52 (-.57 to -.46)	-.13 (-.22 to -.04)
**Massachusetts**	4,740 (4,418 to 5,081)	3,215 (3,011 to 3,427)	3,349 (3,000 to 3,694)	-.29 (-.37 to -.21)	.04 (-.06 to .15)	66 (62 to 71)	38 (35 to 40)	34 (30 to 38)	-.48 (-.55 to -.42)	-.09 (-.18 to .01)

© 2018 Global Burden of Cardiovascular Diseases Collaboration. *JAMA Cardiology*.

**Michigan**	7,187 (6,688 to 7,730)	5,258 (4,943 to 5,613)	5,700 (5,251 to 6,208)	-.21 (-.28 to -.11)	.09 (-.02 to .2)	71 (66 to 77)	42 (40 to 45)	41 (37 to 44)	-.43 (-.49 to -.36)	-.04 (-.12 to .07)
**Minnesota**	2,915 (2,727 to 3,099)	2,473 (2,295 to 2,652)	2,806 (2,500 to 3,110)	-.04 (-.15 to .08)	.14 (.02 to .26)	59 (55 to 63)	38 (35 to 41)	36 (32 to 40)	-.38 (-.46 to -.3)	-.05 (-.15 to .05)
**Mississippi**	2,386 (2,217 to 2,603)	2,384 (2,199 to 2,575)	2,629 (2,327 to 2,973)	.1 (-.04 to .26)	.1 (-.03 to .25)	86 (80 to 94)	70 (65 to 76)	68 (60 to 77)	-.21 (-.31 to -.1)	-.03 (-.16 to .09)
**Missouri**	4,056 (3,810 to 4,309)	3,383 (3,187 to 3,596)	3,770 (3,455 to 4,107)	-.07 (-.16 to .04)	.12 (.01 to .24)	67 (63 to 71)	46 (43 to 49)	45 (41 to 48)	-.33 (-.4 to -.26)	-.03 (-.12 to .08)
**Montana**	823 (764 to 886)	744 (689 to 797)	831 (728 to 947)	.01 (-.12 to .16)	.12 (-.02 to .28)	89 (82 to 95)	58 (54 to 63)	54 (47 to 62)	-.39 (-.47 to -.29)	-.07 (-.19 to .06)
**Nebraska**	1,431 (1,346 to 1,524)	1,122 (1,050 to 1,190)	1,227 (1,112 to 1,350)	-.14 (-.23 to -.04)	.09 (-.01 to .21)	76 (71 to 81)	50 (46 to 53)	48 (43 to 52)	-.37 (-.44 to -.3)	-.04 (-.14 to .06)
**Nevada**	1,124 (1,021 to 1,239)	1,548 (1,425 to 1,715)	1,800 (1,607 to 2,015)	.6 (.41 to .81)	.16 (.03 to .31)	90 (82 to 99)	55 (51 to 60)	47 (42 to 52)	-.48 (-.54 to -.41)	-.14 (-.24 to -.04)
**New Hampshire**	756 (708 to 808)	623 (578 to 667)	733 (659 to 812)	-.03 (-.14 to .09)	.18 (.06 to .31)	64 (60 to 69)	38 (35 to 40)	36 (32 to 41)	-.43 (-.5 to -.36)	-.03 (-.13 to .08)
**New Jersey**	7,250 (6,734 to 7,800)	4,373 (4,103 to 4,647)	4,420 (3,983 to 4,887)	-.39 (-.46 to -.31)	.01 (-.09 to .13)	79 (73 to 85)	40 (37 to 42)	35 (32 to 39)	-.55 (-.61 to -.49)	-.11 (-.2 to -.01)
**New Mexico**	1,312 (1,217 to 1,418)	1,233 (1,158 to 1,319)	1,409 (1,249 to 1,592)	.08 (-.07 to .24)	.14 (0 to .31)	86 (79 to 93)	53 (49 to 56)	51 (45 to 57)	-.41 (-.49 to -.32)	-.04 (-.16 to .1)
**New York**	14,535 (13,475 to 15,641)	7,787 (7,326 to 8,295)	7,908 (7,038 to 8,856)	-.46 (-.53 to -.37)	.02 (-.1 to .14)	70 (65 to 75)	32 (30 to 34)	29 (26 to 33)	-.58 (-.64 to -.52)	-.1 (-.2 to .02)
**North Carolina**	4,817 (4,460 to 5,181)	4,530 (4,267 to 4,799)	5,352 (4,918 to 5,815)	.11 (-.01 to .24)	.18 (.08 to .3)	66 (61 to 71)	42 (40 to 45)	39 (36 to 43)	-.41 (-.47 to -.34)	-.07 (-.16 to .02)
**North Dakota**	452 (421 to 486)	344 (321 to 367)	380 (336 to 428)	-.16 (-.26 to -.05)	.11 (-.02 to .24)	59 (55 to 64)	39 (36 to 42)	38 (33 to 43)	-.35 (-.43 to -.26)	-.02 (-.14 to .1)

© 2018 Global Burden of Cardiovascular Diseases Collaboration. *JAMA Cardiology*.

**Ohio**	9,130 (8,498 to 9,729)	6,289 (5,926 to 6,705)	6,725 (6,185 to 7,300)	-.26 (-.34 to -.18)	.07 (-.03 to .18)	73 (68 to 78)	43 (40 to 46)	41 (38 to 45)	-.44 (-.49 to -.37)	-.03 (-.12 to .06)
**Oklahoma**	2,403 (2,262 to 2,558)	2,367 (2,226 to 2,518)	2,631 (2,415 to 2,857)	.1 (-.01 to .21)	.11 (.02 to .22)	66 (62 to 70)	54 (50 to 57)	52 (48 to 56)	-.21 (-.28 to -.12)	-.03 (-.11 to .06)
**Oregon**	2,403 (2,250 to 2,569)	2,148 (2,015 to 2,289)	2,421 (2,184 to 2,652)	.01 (-.1 to .13)	.13 (.02 to .24)	71 (66 to 76)	45 (42 to 48)	41 (37 to 45)	-.43 (-.49 to -.35)	-.09 (-.18 to .01)
**Pennsylvania**	12,911 (12,093 to 13,750)	7,930 (7,488 to 8,377)	8,036 (7,405 to 8,736)	-.38 (-.44 to -.31)	.01 (-.07 to .1)	85 (80 to 91)	45 (43 to 48)	41 (38 to 45)	-.51 (-.56 to -.46)	-.08 (-.16 to 0)
**Rhode Island**	693 (641 to 746)	487 (449 to 525)	486 (424 to 556)	-.3 (-.39 to -.19)	0 (-.12 to .14)	56 (52 to 60)	34 (31 to 37)	31 (27 to 36)	-.44 (-.52 to -.35)	-.08 (-.2 to .06)
**South Carolina**	2,739 (2,524 to 2,970)	2,788 (2,605 to 2,988)	3,359 (3,031 to 3,755)	.23 (.06 to .42)	.21 (.06 to .37)	75 (69 to 81)	53 (49 to 56)	49 (44 to 55)	-.34 (-.43 to -.24)	-.06 (-.17 to .07)
**South Dakota**	524 (487 to 561)	419 (389 to 449)	484 (426 to 545)	-.08 (-.2 to .06)	.16 (.02 to .3)	62 (58 to 67)	41 (38 to 44)	41 (36 to 47)	-.34 (-.43 to -.24)	0 (-.12 to .14)
**Tennessee**	3,420 (3,190 to 3,655)	3,048 (2,844 to 3,241)	3,615 (3,286 to 3,898)	.06 (-.06 to .17)	.19 (.07 to .3)	62 (58 to 66)	41 (38 to 44)	40 (37 to 44)	-.34 (-.42 to -.27)	-.01 (-.11 to .08)
**Texas**	10,445 (9,741 to 11,205)	10,119 (9,521 to 10,773)	12,481 (11,455 to 13,649)	.2 (.07 to .34)	.23 (.11 to .36)	63 (59 to 68)	41 (39 to 44)	39 (36 to 42)	-.38 (-.45 to -.31)	-.06 (-.15 to .04)
**Utah**	1,720 (1,603 to 1,846)	1,699 (1,597 to 1,815)	2,109 (1,927 to 2,314)	.23 (.1 to .36)	.24 (.12 to .37)	118 (110 to 126)	72 (68 to 77)	68 (62 to 74)	-.42 (-.49 to -.36)	-.06 (-.15 to .04)
**Vermont**	418 (387 to 447)	324 (302 to 346)	359 (324 to 394)	-.14 (-.24 to -.04)	.11 (-.01 to .23)	68 (63 to 73)	39 (36 to 42)	37 (33 to 41)	-.46 (-.52 to -.39)	-.06 (-.16 to .05)
**Virginia**	4,117 (3,873 to 4,411)	3,734 (3,525 to 3,944)	4,161 (3,776 to 4,570)	.01 (-.1 to .14)	.12 (.01 to .23)	65 (61 to 69)	41 (39 to 44)	37 (34 to 41)	-.42 (-.49 to -.35)	-.1 (-.19 to 0)
**Washington**	3,504 (3,269 to 3,748)	2,812 (2,652 to 2,987)	3,252 (2,966 to 3,565)	-.07 (-.17 to .05)	.16 (.04 to .28)	67 (62 to 71)	37 (35 to 39)	33 (30 to 37)	-.5 (-.55 to -.43)	-.09 (-.18 to .01)

© 2018 Global Burden of Cardiovascular Diseases Collaboration. *JAMA Cardiology*.

**West Virginia**	1,763 (1,648 to 1,886)	1,402 (1,311 to 1,498)	1,521 (1,376 to 1,670)	-.14 (-.24 to -.03)	.09 (-.02 to .2)	78 (73 to 84)	56 (52 to 60)	57 (51 to 62)	-.28 (-.36 to -.19)	.01 (-.08 to .12)
**Wisconsin**	3,502 (3,285 to 3,722)	2,915 (2,747 to 3,085)	3,208 (2,935 to 3,487)	-.08 (-.17 to .02)	.1 (.01 to .21)	61 (58 to 65)	40 (38 to 43)	38 (35 to 42)	-.37 (-.43 to -.3)	-.04 (-.13 to .05)
**Wyoming**	418 (387 to 449)	421 (393 to 451)	463 (406 to 527)	.11 (-.04 to .28)	.1 (-.04 to .26)	93 (86 to 100)	66 (62 to 71)	60 (53 to 69)	-.35 (-.44 to -.25)	-.09 (-.21 to .04)

© 2018 Global Burden of Cardiovascular Diseases Collaboration. *JAMA Cardiology*.

eTable 2. Age-standardized heart failure prevalence per 100 000 persons for 2016

	Female	Male
**Alabama**	1001<br>(947 to 1060)	1226<br>(1163 to 1288)
**Alaska**	688<br>(608 to 783)	894<br>(792 to 1006)
**Arizona**	802<br>(757 to 854)	994<br>(947 to 1045)
**Arkansas**	951<br>(873 to 1031)	1152<br>(1090 to 1215)
**California**	826<br>(795 to 852)	1063<br>(1027 to 1097)
**Colorado**	896<br>(843 to 950)	1052<br>(997 to 1106)
**Connecticut**	896<br>(854 to 940)	1180<br>(1136 to 1224)
**Delaware**	854<br>(803 to 912)	1074<br>(1014 to 1140)
**District of Columbia**	868<br>(768 to 984)	1001<br>(881 to 1124)
**Florida**	931<br>(892 to 972)	1156<br>(1128 to 1190)
**Georgia**	914<br>(882 to 944)	1107<br>(1063 to 1160)
**Hawaii**	769<br>(697 to 853)	1164<br>(1071 to 1262)
**Idaho**	848<br>(792 to 907)	1013<br>(949 to 1082)
**Illinois**	887<br>(845 to 928)	1101<br>(1052 to 1156)
**Indiana**	1183<br>(1129 to 1230)	1458<br>(1402 to 1504)
**Iowa**	695<br>(642 to 778)	907<br>(837 to 1003)
**Kansas**	849<br>(795 to 910)	980<br>(905 to 1061)
**Kentucky**	1183<br>(1121 to 1237)	1384<br>(1318 to 1451)
**Louisiana**	998<br>(930 to 1066)	1203<br>(1134 to 1268)
**Maine**	758<br>(702 to 828)	1043<br>(981 to 1109)
**Maryland**	1005<br>(954 to 1055)	1242<br>(1179 to 1309)
**Massachusetts**	805<br>(747 to 867)	1037<br>(976 to 1125)
**Michigan**	1182<br>(1130 to 1221)	1398<br>(1359 to 1427)
**Minnesota**	672<br>(615 to 741)	854<br>(785 to 922)
**Mississippi**	963<br>(898 to 1033)	1188<br>(1111 to 1269)
**Missouri**	1021<br>(979 to 1059)	1290<br>(1233 to 1338)
**Montana**	881<br>(826 to 942)	1099<br>(1026 to 1173)

© 2018 Global Burden of Cardiovascular Diseases Collaboration. *JAMA Cardiology*.

**Nebraska**	844<br>(772 to 923)	1083<br>(1001 to 1180)
**Nevada**	946<br>(890 to 1003)	1107<br>(1043 to 1178)
**New Hampshire**	729<br>(676 to 807)	1009<br>(923 to 1111)
**New Jersey**	987<br>(947 to 1028)	1310<br>(1259 to 1357)
**New Mexico**	914<br>(870 to 961)	1102<br>(1035 to 1195)
**New York**	1151<br>(1106 to 1190)	1515<br>(1469 to 1545)
**North Carolina**	802<br>(760 to 849)	969<br>(920 to 1031)
**North Dakota**	817<br>(725 to 925)	922<br>(816 to 1051)
**Ohio**	1097<br>(1046 to 1140)	1367<br>(1319 to 1406)
**Oklahoma**	1159<br>(1102 to 1209)	1428<br>(1365 to 1477)
**Oregon**	773<br>(720 to 831)	949<br>(882 to 1027)
**Pennsylvania**	923<br>(877 to 968)	1205<br>(1141 to 1271)
**Rhode Island**	858<br>(786 to 938)	1124<br>(1026 to 1227)
**South Carolina**	957<br>(907 to 1009)	1156<br>(1080 to 1241)
**South Dakota**	858<br>(772 to 953)	946<br>(856 to 1057)
**Tennessee**	1035<br>(977 to 1086)	1272<br>(1217 to 1322)
**Texas**	1057<br>(1017 to 1090)	1306<br>(1261 to 1343)
**Utah**	965<br>(899 to 1032)	1100<br>(1030 to 1180)
**Vermont**	703<br>(624 to 800)	869<br>(777 to 988)
**Virginia**	867<br>(828 to 905)	1040<br>(983 to 1113)
**Washington**	720<br>(679 to 766)	911<br>(851 to 975)
**West Virginia**	1151<br>(1075 to 1219)	1314<br>(1250 to 1379)
**Wisconsin**	808<br>(769 to 848)	1009<br>(946 to 1086)
**Wyoming**	833<br>(755 to 925)	1042<br>(953 to 1148)

© 2018 Global Burden of Cardiovascular Diseases Collaboration. *JAMA Cardiology*.

eFigure 1. US State rankings for age-standardized cardiovascular disease disability-adjusted life-year rates per 100 000 persons for both sexes combined in 2016

	Alabama	Alaska	Arizona	Arkansas	California	Colorado	Connecticut	Delaware	District of Columbia	Florida	Georgia	Hawaii	Idaho	Illinois	Indiana	Iowa	Kansas	Kentucky	Louisiana	Maine	Maryland	Massachusetts	Michigan	Minnesota	Mississippi	Missouri
Ischemic heart disease	1	1	1	1	1	1	1	1	1	1	1	1	1	1	1	1	1	1	1	1	1	1	1	1	1	1
Ischemic stroke	2	2	2	2	2	2	2	2	4	2	2	2	2	2	2	2	2	2	2	2	2	2	2	2	2	2
Hemorrhagic stroke	3	3	3	3	3	3	3	3	3	3	3	3	3	3	3	3	3	3	3	3	3	3	3	3	3	3
Other cardiovascular and circulatory diseases	4	4	4	4	5	4	4	4	5	4	5	4	4	4	4	4	4	4	5	4	4	4	4	4	5	4
Hypertensive heart disease	5	6	5	5	4	6	6	5	2	5	4	7	6	5	6	6	7	5	4	7	5	6	5	7	4	5
Other cardiomyopathy	6	7	7	6	7	7	7	7	6	7	6	5	7	6	5	7	6	7	6	6	6	7	6	6	6	7
Atrial fibrillation and flutter	7	5	6	7	6	5	5	6	7	6	7	6	5	7	7	5	5	6	7	5	7	5	7	5	7	6
Rheumatic heart disease	8	8	10	9	11	9	10	10	12	12	9	10	9	9	9	9	9	9	9	9	12	9	9	9	8	9
Aortic aneurysm	9	9	8	8	8	8	8	8	10	8	8	8	8	8	8	8	8	8	8	8	8	8	8	8	9	8
Endocarditis	10	10	9	10	10	10	9	9	9	10	11	11	10	10	10	11	10	10	10	10	10	10	11	10	11	10
Peripheral artery disease	11	12	12	11	12	12	11	12	11	11	12	12	11	11	11	12	12	11	11	12	11	11	12	12	12	11
Alcoholic cardiomyopathy	12	11	11	12	9	11	12	11	8	9	10	9	12	12	12	10	11	12	12	11	9	12	10	11	10	12
Myocarditis	13	13	13	13	13	13	13	13	13	13	13	13	13	13	13	13	13	13	13	13	13	13	13	13	13	13

	Montana	Nebraska	Nevada	New Hampshire	New Jersey	New Mexico	New York	North Carolina	North Dakota	Ohio	Oklahoma	Oregon	Pennsylvania	Rhode Island	South Carolina	South Dakota	Tennessee	Texas	Utah	Vermont	Virginia	Washington	West Virginia	Wisconsin	Wyoming
Ischemic heart disease	1	1	1	1	1	1	1	1	1	1	1	1	1	1	1	1	1	1	1	1	1	1	1	1	1
Ischemic stroke	2	2	2	2	2	2	2	2	2	2	2	2	2	2	2	2	2	2	2	2	2	2	2	2	2
Hemorrhagic stroke	3	3	3	3	3	3	3	3	3	3	3	3	3	3	3	3	3	3	3	4	3	3	3	3	4
Other cardiovascular and circulatory diseases	4	4	5	4	4	4	4	4	4	4	4	4	4	4	4	4	4	4	4	3	4	4	4	4	3
Hypertensive heart disease	6	6	4	6	6	5	5	5	6	5	5	6	7	6	5	6	5	5	6	6	6	6	5	6	6
Other cardiomyopathy	7	7	6	7	7	7	7	7	7	7	7	7	6	7	6	7	7	7	7	7	7	7	7	7	7
Atrial fibrillation and flutter	5	5	7	5	5	6	6	6	5	6	6	5	5	5	7	5	6	6	5	5	5	5	6	5	5
Rheumatic heart disease	9	9	9	9	11	8	10	10	10	10	9	9	10	11	9	9	11	9	8	9	10	11	9	9	9
Aortic aneurysm	8	8	8	8	8	9	8	8	8	8	8	8	8	8	8	8	8	8	10	8	8	8	8	8	8
Endocarditis	10	10	11	10	9	10	9	11	9	9	10	10	9	9	10	10	10	10	9	10	11	9	11	11	10
Peripheral artery disease	11	11	12	11	12	12	11	12	11	11	11	12	11	10	11	11	12	11	11	11	12	12	12	12	11
Alcoholic cardiomyopathy	12	12	10	12	10	11	12	9	12	12	12	11	12	12	12	12	9	12	12	12	9	10	10	10	12
Myocarditis	13	13	13	13	13	13	13	13	13	13	13	13	13	13	13	13	13	13	13	13	13	13	13	13	13

© 2018 Global Burden of Cardiovascular Diseases Collaboration. *JAMA Cardiology*.

eFigure 2. Proportion of cardiovascular disease disability-adjusted life-years due to years lived with disability in 2016

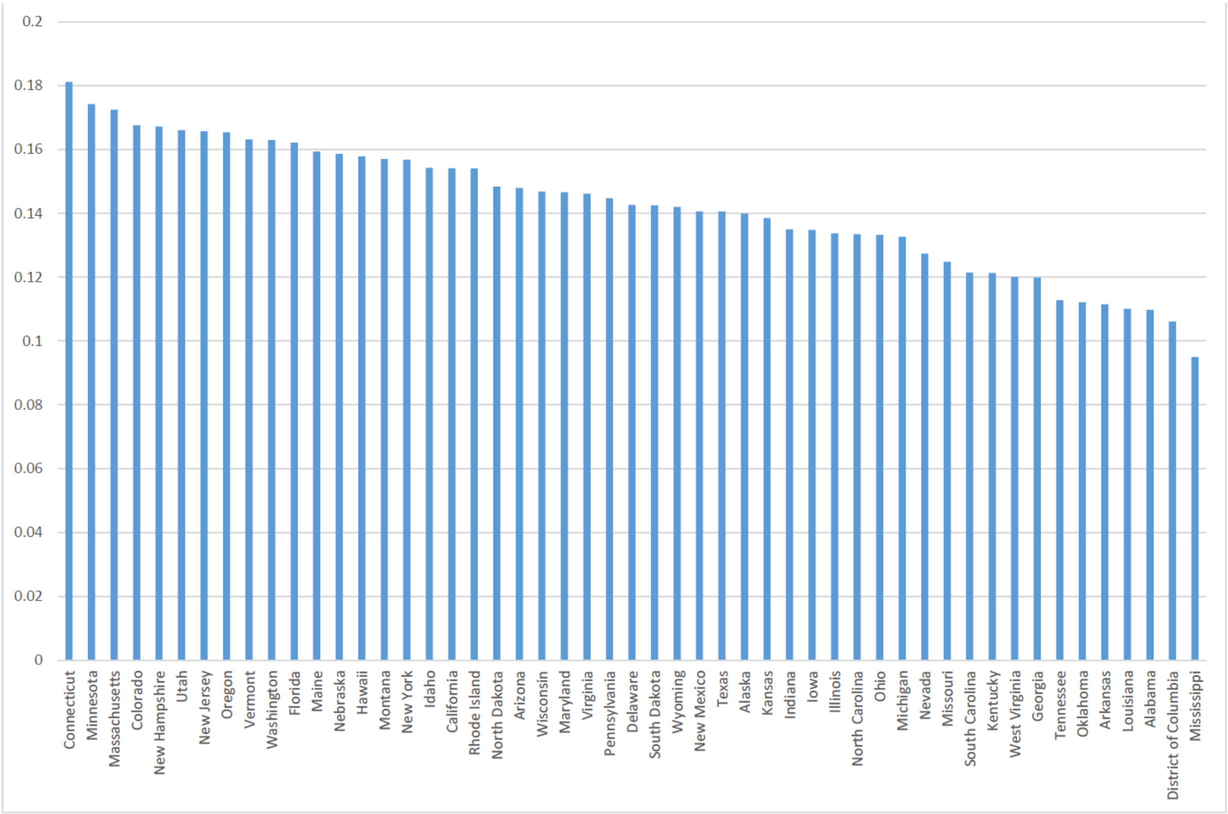

© 2018 Global Burden of Cardiovascular Diseases Collaboration. *JAMA Cardiology*.

eFigure 3. Leading level 2 cardiovascular risk factors for both sexes for Minnesota and Mississippi

A. Minnesota

Leading level 2 cardiovascular risk factors of Minnesota, both sexes

Risks are connected by lines between time periods. Behavioural risk factors are shown in red, environmental risks in blue and metabolic risks in green.

For the time period 1990 to 2006 and for 2006 to 2016, three measures of change are shown: percent change in the number of DALYs, percent change in the all-age DALY rate and percent change in the age-standardised DALY rate.

Statistically significant increases or decreases are shown in bold ($p < 0.05$). DALYs=disability-adjusted life-years.

Leading risks 1990	Leading risks 2006	Mean % change number of DALYs 1990-2006	Mean % change all-age DALY rate 1990-2006	Mean % change age-standardised DALY rate 1990-2006	Leading risks 2016	Mean % change number of DALYs 2006-2016	Mean % change all-age DALY rate 2006-2016	Mean % change age-standardised DALY rate 2006-2016
1 Dietary risks	1 Dietary risks	**-32.8%**	**-43.0%**	**-49.0%**	1 Dietary risks	7.3%	0.4%	**-11.0%**
2 High blood pressure	2 High blood pressure	**-32.8%**	**-43.0%**	**-49.2%**	2 High blood pressure	5.3%	-1.4%	**-13.7%**
3 High total cholesterol	3 High body-mass index	0.2%	-15.0%	**-24.6%**	3 High body-mass index	**12.1%**	4.9%	-7.1%
4 High body-mass index	4 High total cholesterol	**-36.1%**	**-45.8%**	**-53.2%**	4 High total cholesterol	-1.0%	-7.3%	**-16.0%**
5 Tobacco	5 Tobacco	**-32.8%**	**-43.0%**	**-49.9%**	5 High fasting plasma glucose	5.6%	-1.2%	-14.6%
6 High fasting plasma glucose	6 High fasting plasma glucose	-9.7%	-23.4%	-30.1%	6 Tobacco	**-13.8%**	**-19.3%**	**-27.5%**
7 Low physical activity	7 Low physical activity	-28.0%	-38.9%	-45.0%	7 Low physical activity	0.7%	-5.8%	-17.7%
8 Air pollution	8 Impaired kidney function	**-25.4%**	**-36.7%**	**-43.1%**	8 Impaired kidney function	7.3%	0.4%	**-11.3%**
9 Impaired kidney function	9 Air pollution	**-33.7%**	**-43.8%**	**-50.0%**	9 Air pollution	0.0%	-6.4%	**-16.3%**
10 Other environmental	10 Occupational risks	**-29.9%**	**-40.6%**	**-50.0%**	10 Occupational risks	1.7%	-4.8%	**-14.7%**
11 Occupational risks	11 Other environmental				11 Other environmental			

Legend:
Environmental
Behavioural
Metabolic

© 2018 Global Burden of Cardiovascular Diseases Collaboration. *JAMA Cardiology*.

B. Mississippi

Leading level 2 cardiovascular risk factors of Mississippi, both sexes

Risks are connected by lines between time periods. Behavioural risk factors are shown in red, environmental risks in blue and metabolic risks in green.

For the time period 1990 to 2006 and for 2006 to 2016, three measures of change are shown: percent change in the number of DALYs, percent change in the all-age DALY rate and percent change in the age-standardised DALY rate.

Statistically significant increases or decreases are shown in bold (p < 0.05). DALYs=disability-adjusted life-years.

Leading risks 1990	Leading risks 2006	Mean % change number of DALYs 1990-2006	Mean % change all-age DALY rate 1990-2006	Mean % change age-standardised DALY rate 1990-2006	Leading risks 2016	Mean % change number of DALYs 2006-2016	Mean % change all-age DALY rate 2006-2016	Mean % change age-standardised DALY rate 2006-2016
1 Dietary risks	1 Dietary risks	**-9.9%**	**-19.7%**	**-27.6%**	1 Dietary risks	3.4%	0.5%	-9.6%
2 High blood pressure	2 High blood pressure	**-11.0%**	**-20.6%**	**-28.0%**	2 High blood pressure	3.7%	0.8%	**-10.2%**
3 High total cholesterol	3 High body-mass index	**33.3%**	**18.9%**	4.6%	3 High body-mass index	5.0%	2.1%	-6.9%
4 High body-mass index	4 High total cholesterol	**-12.8%**	**-22.3%**	**-31.6%**	4 High total cholesterol	-2.6%	-5.3%	**-12.9%**
5 Tobacco	5 Tobacco	**-8.8%**	**-18.7%**	**-28.9%**	5 High fasting plasma glucose	6.7%	3.7%	-10.4%
6 High fasting plasma glucose	6 High fasting plasma glucose	37.6%	22.7%	12.9%	6 Tobacco	**-11.9%**	**-14.4%**	**-20.9%**
7 Air pollution	7 Low physical activity	-6.2%	-16.4%	-23.2%	7 Impaired kidney function	7.1%	4.1%	-8.1%
8 Low physical activity	8 Air pollution	**-12.9%**	**-22.4%**	**-30.5%**	8 Low physical activity	-3.1%	-5.8%	-17.1%
9 Impaired kidney function	9 Impaired kidney function	**-8.8%**	**-18.7%**	**-25.1%**	9 Air pollution	-8.5%	-11.1%	**-19.3%**
10 Occupational risks	10 Alcohol & drug use	57.3%	40.2%	20.3%	10 Alcohol & drug use	14.5%	11.3%	5.9%
12 Alcohol & drug use	11 Occupational risks				11 Occupational risks			

Legend:
Environmental
Behavioural
Metabolic

© 2018 Global Burden of Cardiovascular Diseases Collaboration. *JAMA Cardiology*.

eFigure 4. US State drivers of change in cardiovascular disease from 1990 to 2016

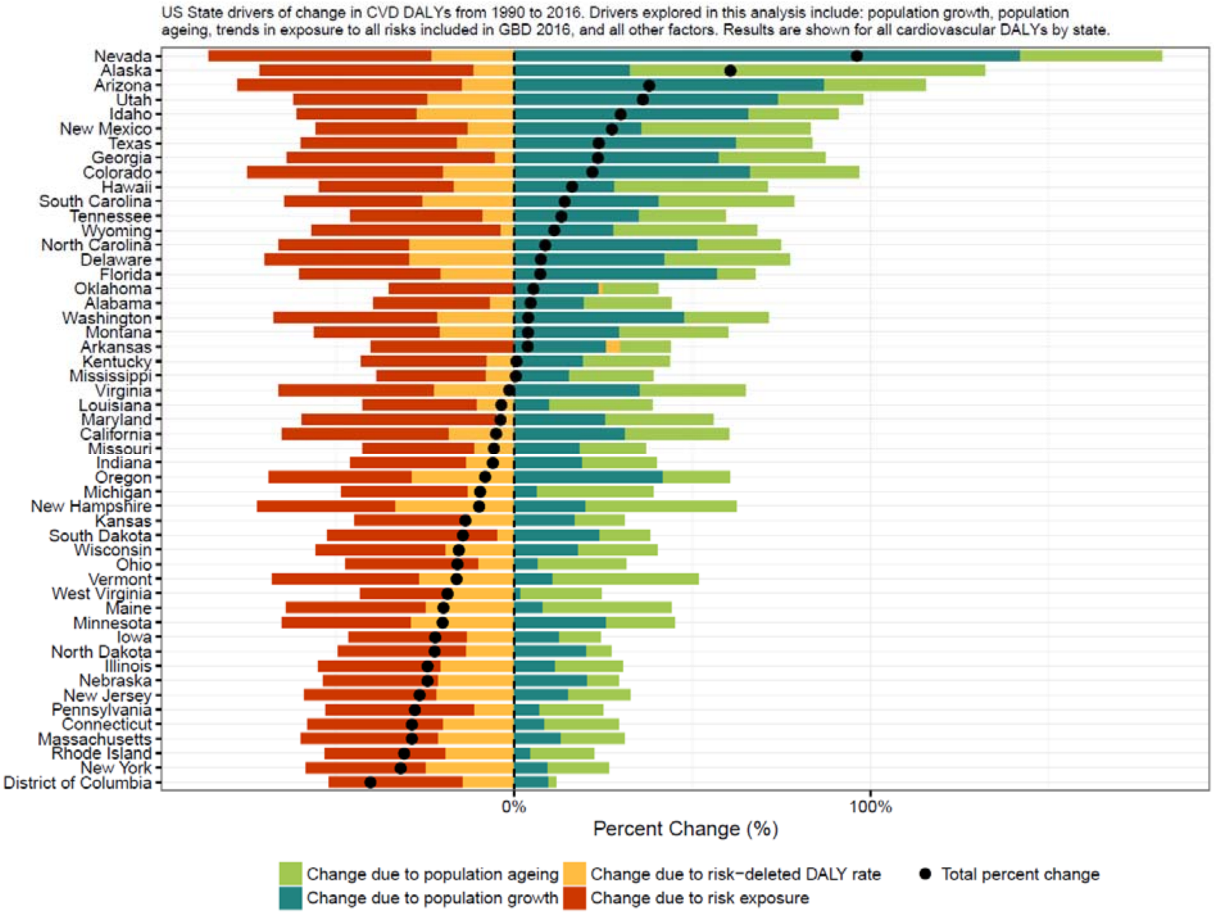

© 2018 Global Burden of Cardiovascular Diseases Collaboration. *JAMA Cardiology*.

eFigure 5. Age-standardized percentage change in disability-adjusted life-year rate between 2010 and 2016 for all cardiovascular diseases in men and women

A. Men

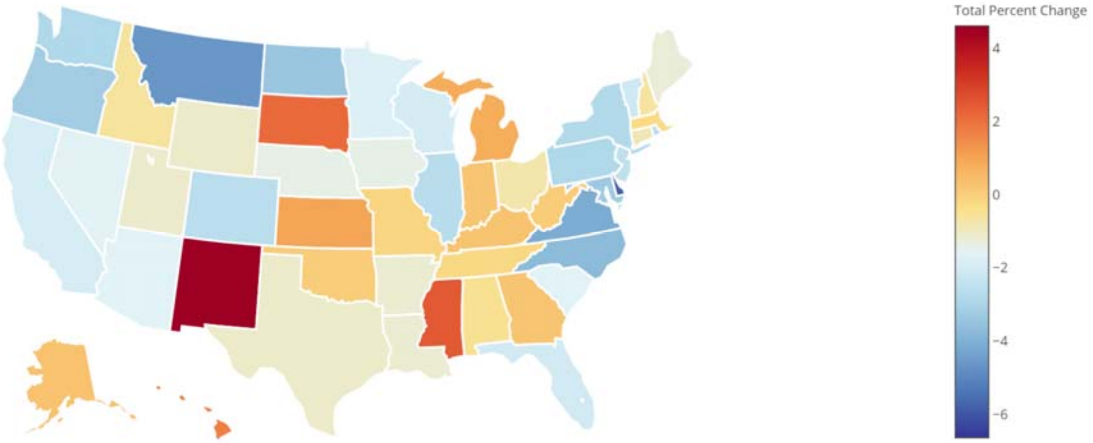

B. Women

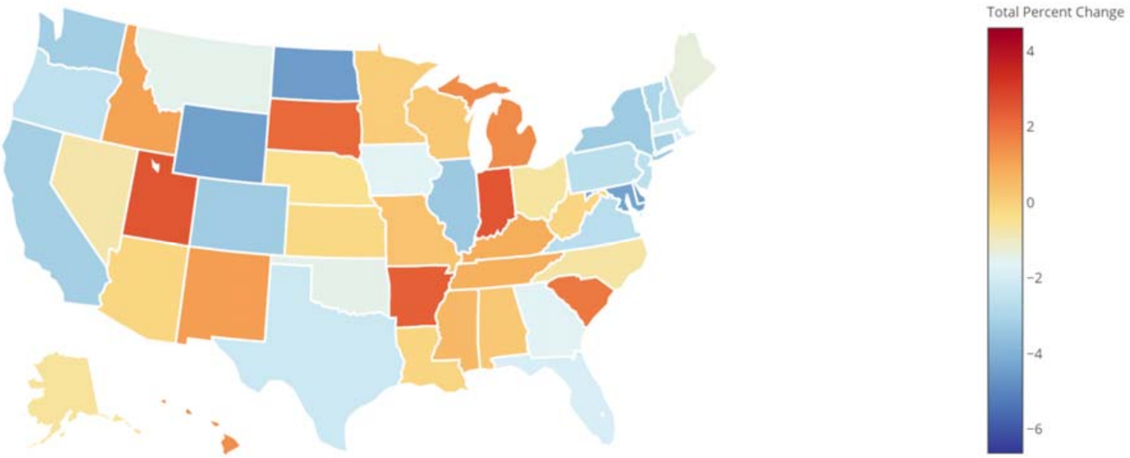

© 2018 Global Burden of Cardiovascular Diseases Collaboration. *JAMA Cardiology*.
